# A Trifunctional, Rare-Earth Theranostic Chelator Platform to Enable Diagnostic Nuclear Imaging, Surgical Resection, and Radiotherapy

**DOI:** 10.1021/jacs.5c15147

**Published:** 2025-12-01

**Authors:** M. Andrey Joaqui-Joaqui, Georgia G. Sands, Dariusz Śmiłowicz, Mallory J. Gork, Eduardo Aluicio-Sarduy, Todd E. Barnhart, Jonathan W. Engle, Eszter Boros

**Affiliations:** † Department of Chemistry, 5228 University of Wisconsin Madison, 1101 University Avenue, Madison, Wisconsin 53705, United States; ‡ Department of Medical Physics, University of Wisconsin-Madison, 1111 Highland Avenue, Madison, Wisconsin 53705, United States; § Department of Radiology, University of Wisconsin-Madison, Madison, Wisconsin 53705, United States

## Abstract

Here, we establish a single-molecule, trifunctional platform compatible with diagnostic positron emission tomography (PET), optical imaging-assisted intrasurgical resection, and chelation of radiotherapeutic rare-earth nuclides. Building on previous pyridylalkynylaryl (PEPA) antenna scaffolds, mono- and bisantenna analogs incorporating either PEPA or methoxy-substituted MEPA chromophores were prepared to assess the role of pyridylalkynylaryl number and polyethylene glycol (PEG) substitution on photophysical and biological performance. Photophysical characterization with Eu­(III) revealed that antenna truncation had minimal impact on quantum yields and Cerenkov radiation energy transfer (CRET) imaging while substantially improving water solubility. Biodistribution studies with Y-86-labeled analogs identified [^86^Y]­Y-pepa-pic_2_ as the lead scaffold, showing rapid renal clearance, minimal nontarget retention, and high in vivo stability. This platform was functionalized with the peptide sequence C-Hex-KuE targeting prostate-specific membrane antigen. PET imaging with the corresponding ^86^Y-based probe showed selective tumor uptake and rapid renal clearance, affirming that the pepa-pic_2_ chelator produced a favorable in vivo profile. Intratumoral administration of Eu-pepa-pic_2_-C-Hex-KuE, paired with the systemically injected [^68^Ga]­Ga-PSMA-617 tracer as the intermolecular CRET photon source, produces selective enhancement of the optical signal in the target tissue. Finally, in vivo studies with [^177^Lu]­Lu-pepa-pic_2_-C-Hex-KuE and [^161^Tb]­Tb-pepa-pic_2_-C-Hex-KuE evidence efficient tumor uptake of β^–^ radiotherapeutics. Together, these results establish a versatile, single chelator platform for targeted rare-earth-metal optical imaging and theranostic nuclear medicine.

## Graphical abstract

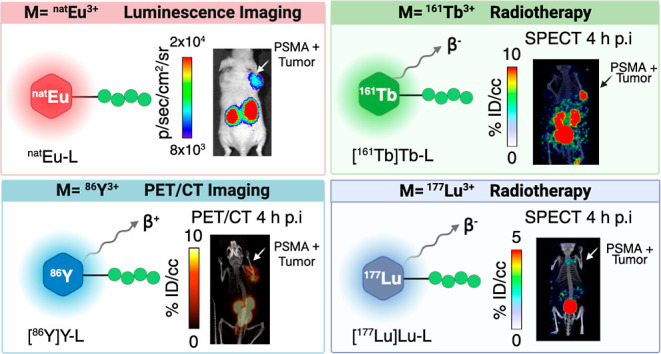

## Introduction

Theranostic agents that diagnose and stratify patients for matching therapeutic treatment are an emerging area of interest in personalized medicine.^1–3^ Single-molecule platforms designed to provide compatibility with diagnostic and therapeutic capabilities within a single construct enable sequential disease detection, treatment, and real-time monitoring of therapeutic outcomes. In nuclear medicine, this often involves pairing a diagnostic radionuclide for positron emission tomography (PET) or single photon emission computed tomography (SPECT) imaging with a chemically equivalent therapeutic radionuclide, ensuring that the pharmacokinetics and targeting properties are identical between diagnosis and therapy.^4^ In oncology, early stage disease management includes surgical intervention by tumor resection with healthy margins to improve treatment efficacy.^5^ As such, combining theranostic platforms with an optical imaging beacon, such as a luminescent probe, adds value by enabling high-resolution, real-time visualization of disease sites at the cellular or intraoperative level, complementing whole-body nuclear imaging.

However, the currently established, clinical standard theranostic pair ^68^Ga^3+^/^177^Lu^3+^ is limited by the short half-life of the diagnostic isotope (*t*_1/2_ = 1.1 h) and incompatible with predictive dosimetry to tune the therapy dose. This incompatibility arises from the differing coordination numbers for Ga^3+^ (6 coordinate) and Lu^3+^ (8 coordinate), producing chemically distinct coordination complexes with differential pharmacokinetic properties and resulting in pronounced differences in tumor and off-target tissue distribution.^6^ Furthermore, while combined bimodal nuclear/optical probes, incorporating both diagnostic radionuclides and organic chromophores,^7–11^ have shown promise in preclinical studies, their clinical implementation remains challenging; altered pharmacokinetics and lipophilicity, combined with regulatory hurdles, have slowed translation. By incorporating a diagnostic and therapeutic isotope with similar chemical properties into one ligand scaffold, improvements in dosimetry are expected, allowing for personalized treatment.

The rare-earth elements yttrium (Y), europium (Eu), terbium (Tb), and lutetium (Lu) exhibit close chemical homology. Europium has biocompatible luminescence properties, while Y, Tb, and Lu possess various clinically effective short-lived radionuclides suitable for nuclear imaging and radiotherapy applications. As such, a single chelation approach compatible with each modality and element of interest could vastly streamline development, clinical translation, and application of a trifunctional platform technology. Specifically, several regulatory agencies have accelerated translation of chemically homologous molecular agents by concerted toxicology assessment. Furthermore, the close chemical and biological homology enables prediction of the pharmacokinetics of the optical and therapeutic agent based on the diagnostic and personalized dosimetry. ^86^Y^3+^ (*t*_1/2_ = 14.7 h, *E*_β+_ = 660 keV, 31.9%) is suitable for noninvasive, diagnostic, PET imaging,^12,13^ and Eu^3+^ emits red light (620 and 670 nm, comparing well to clinically used porphyrin induced by 5-ALA) that is ideal for intraoperative optical imaging.^14–16^ For radiotherapy, ^177^Lu^3+^ (*t*_1/2_ = 160 h, *E*_β‑_ = 149 keV) clinical efficacy^17,18^ and ^161^Tb^3+^ (*t*_1/2_ = 166 h, *E*_β‑_ = 154 keV, 100%, in addition to Auger and internal conversion electrons) demonstrated compatibility with low dose β^–^ therapy.^17,18^ While ^nat^Tb has been extensively studied for its luminescent properties, its characteristic green emission does not penetrate skin as deeply as the longer wavelength, red emission of europium, making europium an ideal candidate for optical imaging.^19^

All four elements are stabilized in the trivalent state, with ionic radii of 1.075 Å (Y^3+^), 1.032 Å (Lu^3+^), 1.095 Å (Tb^3+^), and 1.12 Å (Eu^3+^) as nine-coordinate complexes, respectively, rendering them chemically closely homologous (Figure 1).^20^ In spite of the favorable properties and possible multimodality applications, a functional, singular molecular chelation platform compatible with diagnostic, optically active, or therapeutic particle-emissive rare earths remains elusive.

**Figure 1:**
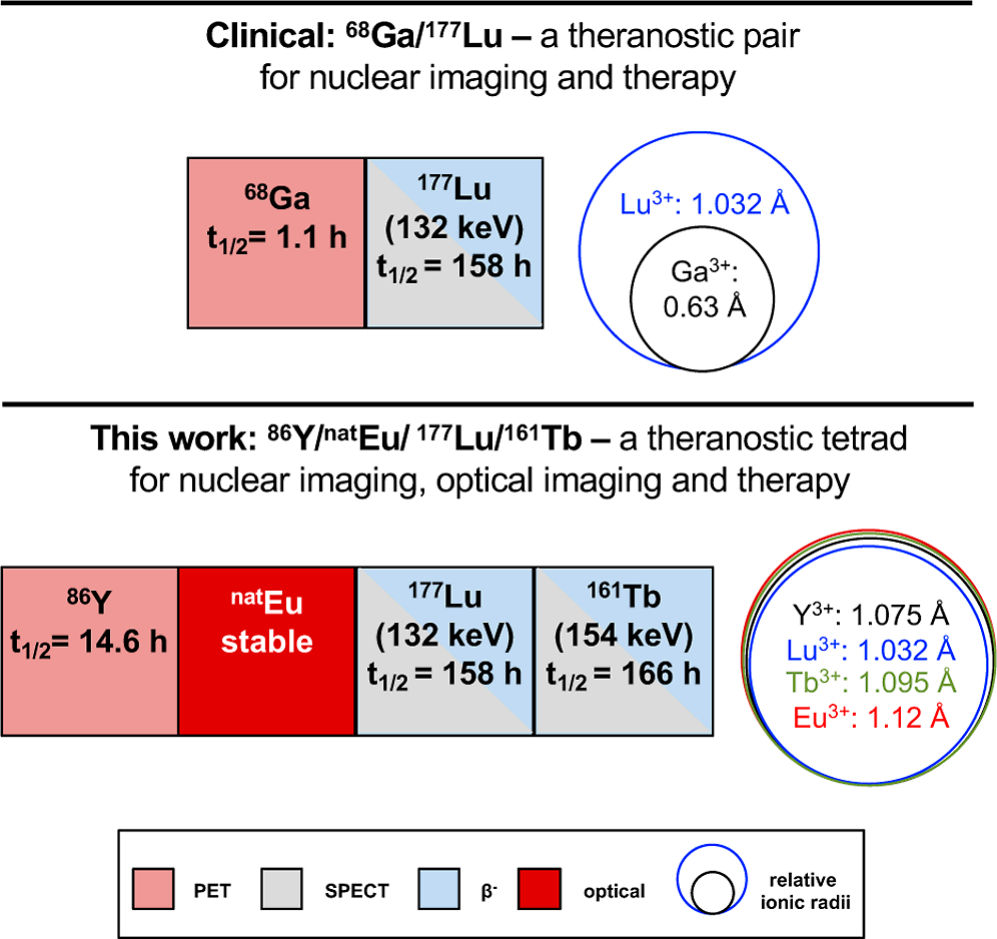
Overview of properties of clinically employed nuclear theranostic pair, compared with a potential, exclusively rare-earth element-based theranostic tetrad, which also includes optical imaging.

The greatest challenge in implementing a molecular, lanthanide-based optical imaging platform remains the requirement for short-wave excitation below 400 nm, which is inherently incompatible with imaging in higher organisms. Cellular imaging has been achieved with various Tb-, Eu-, Dy-, and Yb-chelate constructs,^21–24^ generally necessitating custom-built, confocal microscopy instruments compatible with two-photon (2P) excitation or time-resolved luminescence imaging.

Lanthanide-based nanoparticles have shown compatibility with in vivo imaging using photon upconversion strategies and taking advantage of the high localized concentration of lanthanide ions.^25^ Nanoparticles, however, remain largely incompatible with targeted imaging approaches. In vivo imaging with a molecular system was demonstrated for the first time by Zhang and co-workers using a locally administered, nontargeted, Kläuli-ligand-bound Yb complex.^26^

An alternative strategy to external excitation and upconversion was introduced by in situ sensitization of Eu-based nanoparticles with Cerenkov luminescence produced by positron-emitting radionuclides.^27^ Subsequently, our group has successfully demonstrated that a combination of Cerenkov luminescence-producing radionuclides can efficiently sensitize molecular Eu^3+^ complexes in situ. However, the corresponding conjugates had poor pharmacokinetic properties and were incompatible with systemic administration.^28^

Here, we investigate strategies to furnish Eu^3+^ chelates with improved water-solubility and pharmacokinetics but without diminished brightness. We study the in vivo compatibility with ^86^Y-surrogate complexes and demonstrate that the lead compound, functionalized with a cancer-homing peptide, is compatible with targeted nuclear imaging, in situ optical imaging, and radiotherapeutic nuclides.

## Results and Discussion

### Ligand Design

Previously, we reported the synthesis and photophysical properties of a series of Eu-based polyazamacrocylic molecular probes compatible with in vivo luminescence imaging. The first-generation probe, [Eu­(DO3Aphen)], featured phenanthroline—a planar heterocycle with an extended π-system—as the antenna for the sensitization of Eu^3+^.^29^ Evaluation of its photophysical properties revealed that [Eu (DO3Aphen)] exhibited a moderate quantum yield of (Φ_Eu_: 15%) due to the energy mismatch between the triplet state T_1_ of phenanthroline compared to the accessible, lanthanide 4f-excited states (^5^D_0_ and ^5^D_1_, Figure 2a). Previous optimization focused on the use of the polyethylene-glycol-pyridylalkynylaryl (pepa)-based antenna that demonstrated a better match of excited-state energy for Eu^3+^ and resulted in improved limits of detection in vivo (Figure 2a).^28^ Despite this improvement, the incorporation of two pendant pepa antennae increased the lipophilicity of the Eu-complex, leading to limited water-solubility and biocompatibility.

These results indicated that further optimization of the chelating platform was required to produce Eu-chelates with reduced lipophilicity while retaining favorable thermodynamics and optical properties such as high Φ_Eu_. Thus, we probed the influence of changing the nature and number of the pyridylalkynylaryl antennae in combination with chelating picolyl antennae. To this end, we designed and synthesized analogs of Eu-pepa_2_-pic in which the pepa antenna was replaced by methylethylene-glycol-pyridylalkynylaryl (mepa). This substitution allowed us to isolate and assess the specific role of the PEG moiety in modulating the solubility and pharmacokinetics. In parallel, we synthesized truncated analogs bearing only one pyridylalkynylaryl unit, namely, Eu-pepa-pic_2_ and Eu-mepa-pic_2_ (Figure 2b). This strategy was intended to further reduce the lipophilicity of the resulting constructs while retaining the same number and type of chelating ligand donors to exclude inner-sphere hydration-induced quenching. Given that the Eu-based luminescence requires efficient energy transfer from the antenna, we investigated whether removing one of the two pyridylalkynylaryl antennae would significantly diminish the luminescent output. Alternatively, we considered whether the photophysical properties would remain largely unaffected or comparable to those of the parent compounds featuring two pyridylalkynylaryl antennae.

**Figure 2:**
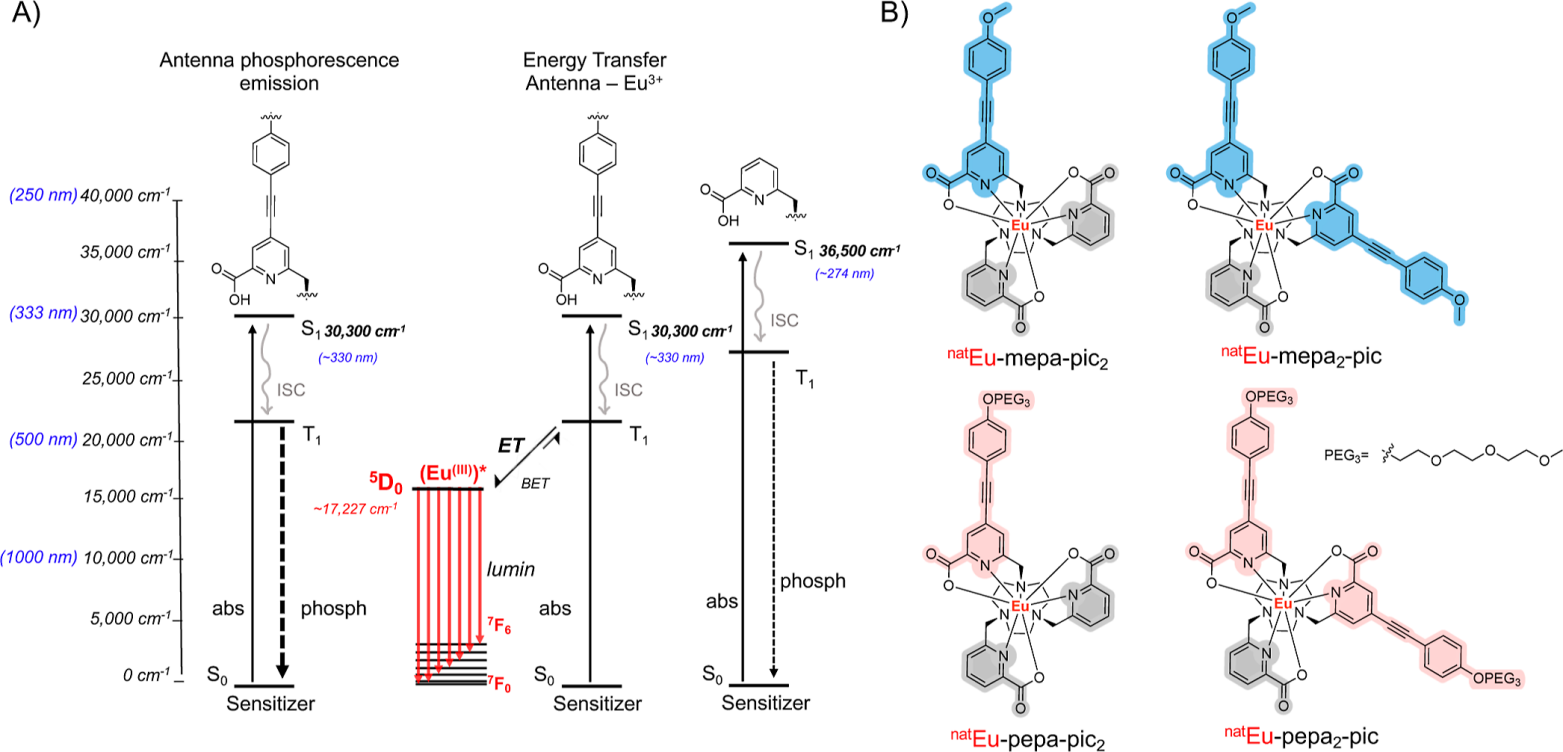
(A) Dieke diagram showing the energy levels of trivalent Eu^3+^, the singlet (S_1_), and triplet (T_1_) state energy of various antennae and the sensitization of Europium via energy transfer—dual antenna excitation can lead to ligand-centered emission (left) or energy transfer (right). (B) Chemical structures of the pyridylalkynylaryl-based Eu-complexes Eu-mepa-pic_2_, Eu-mepa_2_-pic, Eu-pepa-pic_2_, and Eu-pepa_2_-pic.

Synthesis of mepa-pic_2_ and mepa_2_-pic was achieved through a stepwise alkylation of the triazacyclononane (tacn) scaffold (Scheme S1) in accordance with literature procedures as established by Parker, Maury, and co-workers.^30–32^ Sequential alkylation steps, followed by a concerted basic hydrolysis, yielded deprotected-chelating scaffolds mepa-pic_2_ and mepa_2_-pic. An analogous strategy was initially pursued for the synthesis of ligand pepa-pic_2_; however, purification of the resulting intermediates presented significant challenges. To circumvent this, an alternative synthetic route was employed, wherein monoboc-protected tacn (Scheme S2) was first functionalized with two picolinic acid-chelating arms. Subsequent removal of the Boc-protecting group enabled the incorporation of the pepa antenna in the final step prior to global basic hydrolysis to afford the final ligands. Subsequent formation of the corresponding Y^3+^ and Eu^3+^ complexes was carried out in aqueous media at pH 5.5.

### Photophysical Characterization and In Vitro CRET Analysis

The photophysical properties of the Eu^3+^ complexes were characterized, including acquisition of absorption, excitation and emission profiles, molar extinction coefficients, and luminescence lifetimes, to approximate inner sphere hydration numbers (q) and quantum yields (Table 1).^33,34^ All chelating platforms—mepa-pic_2_, mepa_2_-pic, pepa-pic_2_, and pepa_2_-pic—showed absorption bands with a maximum centered at 318 nm attributable to the intraligand charge transfer (ILCT) transition of the pyridylalkynylaryl antenna.^35–37^ Upon coordination to Eu^3+^, a bathochromic shift occurred, moving the absorption maximum to longer wavelengths (330–336 nm). These values are consistent with results previously reported for homologous Eu complexes containing pyridylalkynylaryl antennae.^24,38^

**Table 1:** Ligand and Eu Complexes Photophysical Properties^a^

compound	λ_max_ [nm]	ε [M^–1^ cm^–1^]	Φ_Eu_ [%]	*q*
mepa-pic_2_	318	23783^b^	-	-
mepa_2_-pic	318	72734^b^	-	-
pepa-pic_2_	318	24950^b^	-	-
pepa_2_-pic	318	43958^b^	-	-
Eu-mepa-pic_2_	330	33668^b^	28^d^	0
Eu-mepa_2_-pic	334	43531^b^	25^e^	0
Eu-pepa-pic_2_	334	27516^b^	42^e^	0
Eu-pepa_2_-pic	336	65230^b^	32^f^	0
Eu·L^1^ ^c^	338	55000^c^	25^c^	-

a Experimental conditions.

b 295 K, 10 mM pH 5.5 NaOAc.

c 295 K, MeOH (ref 30).

d 295 K, H_2_O, reference Eu-pepa_2_-pic.

e 295 K, 20% EtOH in H_2_O, reference Eu-pepa_2_-pic.

f 295 K, H_2_O, reference quinine sulfate (ref 28).

All Eu^3+^ complexes show the characteristic photoluminescence profile originating from the ^5^D_0_ - ^7^F_J_ transitions (*J* = 0–6) of the metal center (Supporting Information, Figures S4, S10, and S14). In all cases, the number of inner sphere bound waters was estimated to be zero based on the Horrock’s equation. This is desirable for luminescent Eu^3+^ molecular probes to exclude nonradiative luminescence quenching by OH oscillators.^39^ Notably, complexes with two pyridylalkynylaryl chromophores, Eu-pepa_2_-pic and Eu-mepa_2_-pic, did not exhibit greater luminescence quantum yields than of Eu-pepa-pic_2_ and Eu-mepa-pic_2_ complexes (Table 1). This finding suggests that replacing a picolinate unit, a less favorable antenna for Eu, with a second pyridylalkynylaryl unit, which acts as a much better sensitizer for europium, does not lead to a substantial enhancement of the overall luminescence quantum yields. The latter remains consistent as long as quenching O–H oscillators remain excluded from the inner sphere (Figure 2). Of note, the quantum yields compare well to other pepa-containing Eu complexes such as Eu·L^1c^ by Parker and co-workers (25% in EtOH).^30^

Next, we conducted phantom imaging experiments to assess the suitability of the Eu complexes for optical imaging via in situ excitation through CRET. Samples containing increasing quantities of metal complex (0–50 nmol) were doped with 10 μCi ^68^Ga^3+^ and imaged using a small animal IVIS scanner. No external source of excitation was used, and images were acquired using the 620 nm emission filter, which selectively captures the ^5^D_0_ → ^7^F_2_ de-excitation of Eu^3+^. As observed in Figure 3a, the radiance increases as a function of the concentration of Eu complex. Region of interest (ROI) analysis revealed that no substantial difference in the limit of detection is observed between complexes featuring one or two of the same pyridylalkynylaryl units (Figure 3b). In all cases, the lowest amount of complex that produced a signal with a statistically significant difference from Cerenkov emitter-containing samples was 1 nmol of complex. The results are fully consistent with the quantum yield measurements, demonstrating that incorporation of a second pyridylalkynylaryl chromophore unit offers no significant advantage, as it does not produce any substantial enhancement in the CRET-mediated luminescence. CRET experiments were also carried out in the presence of ^90^Y ^3+^, one of the brightest Cerenkov-emitting radionuclides per activity concentration.^40^ Similarly to the results observed with ^68^Ga^3+^, results correlate well with the quantum yields measured. This further affirms that a single pyridylalkynylaryl-sensitizing antenna is sufficient to retain the favorable photophysical properties of the parent probe.

**Figure 3:**
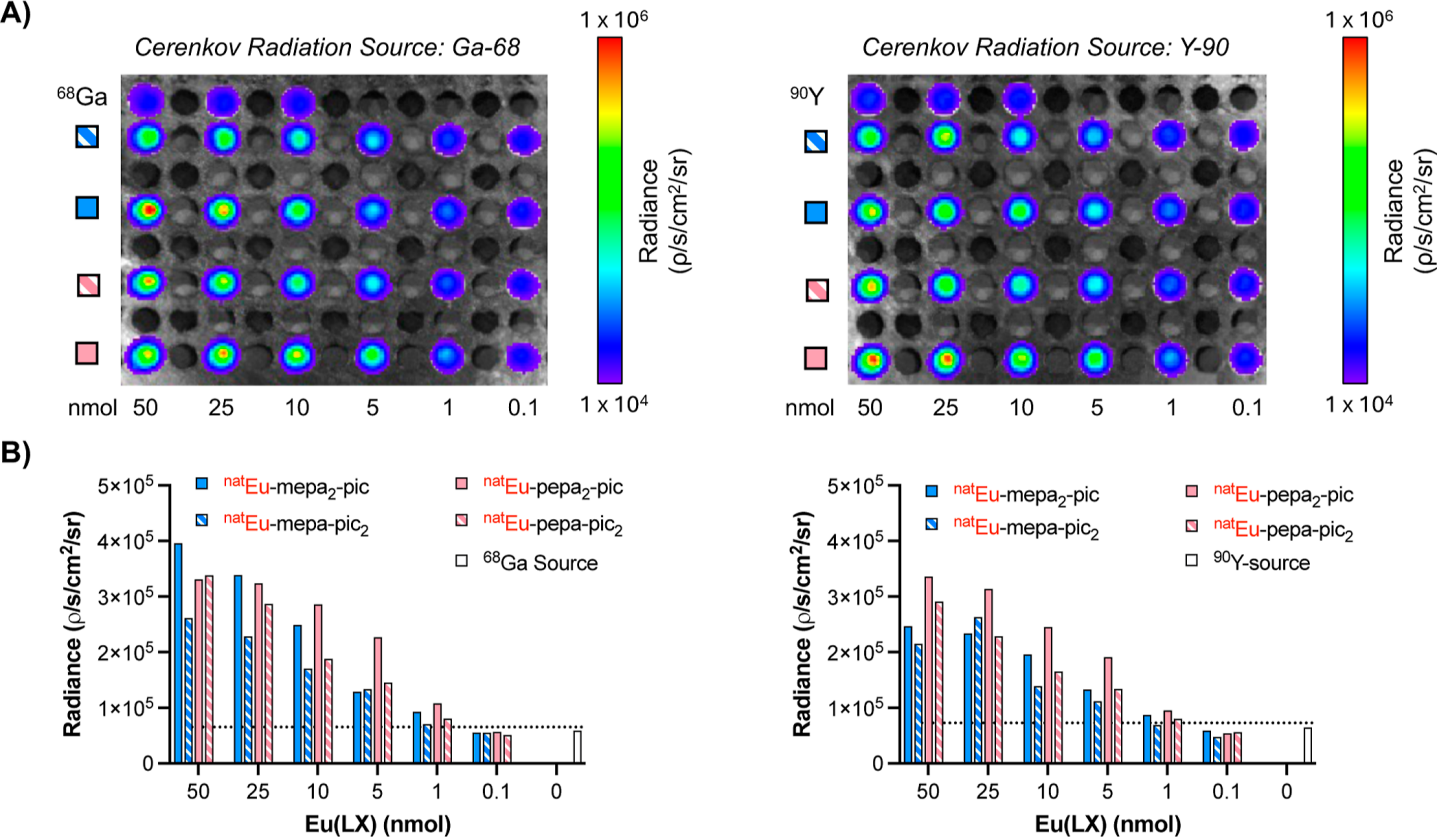
(A) CRET in vitro phantom imaging assays of various amounts of Eu-mepa-pic_2_, Eu-mepa_2_-pic, Eu-pepa-pic_2_, and Eu-pepa_2_-pic doped with ^68^Ga or ^90^Y (620 nm filter). (B) Luminescence signal intensity within the region of interest from phantom images of Eu-mepa-pic_2_, Eu-mepa_2_-pic, Eu-pepa-pic_2_, and Eu-pepa_2_-pic excited by CRET from ^68^Ga or ^90^Y. The dotted line represents the limit of detection, indicating the minimum amount of Eu complex required to produce a signal distinguishable from the background noise via CRET (620 nm filter).

### Pharmacokinetic Optimization

We next probed the pharmacokinetic properties of the four chelator constructs. For this purpose, we radiolabeled mepa-pic_2_, pepa-pic_2_, and pepa_2_-pic with ^86^Y^3+^ (*t*_1/2_ = 14.7 h), positron emitting nuclide representing a suitable surrogate for medium-small ionic radius lanthanides.^41–43^

Optimization of radiolabeling conditions was conducted using ^86^YCl_3_ at pH 5.5, to yield [^86^Y]­Y-mepa-pic_2_, [^86^Y]­Y-pepa-pic_2_, and [^86^Y]­Y-pepa_2_-pic with molar specific activities of 6.9 mCi/μmol, 12.8 mCi/μmol, and 7.2 mCi/μmol, respectively. Due to the high radiochemical purity of the resulting radiotracers, no additional purification or isolation processes were conducted prior to subsequent in vivo studies (Figure 4a–c). A lower radiochemical conversion was observed for the formation of [^86^Y]­Y-mepa_2_-pic, which we attribute to the limited solubility of the ligand in water. Indeed, determination of the logD_7.4_, revealed that [^86^Y]­Y-mepa_2_-pic exhibits the most lipophilic character, followed by [^86^Y]­Y-pepa_2_-pic and [^86^Y]­Y-mepa-pic_2_, rendering [^86^Y]­Y-pepa-pic_2_ the most hydrophilic complex (Figure 4d). Conclusively, we excluded [^86^Y]­Y-mepa_2_-pic from the in vivo pharmacokinetic experiments due to its high lipophilic character and poor radiochemical labeling properties.

**Figure 4:**
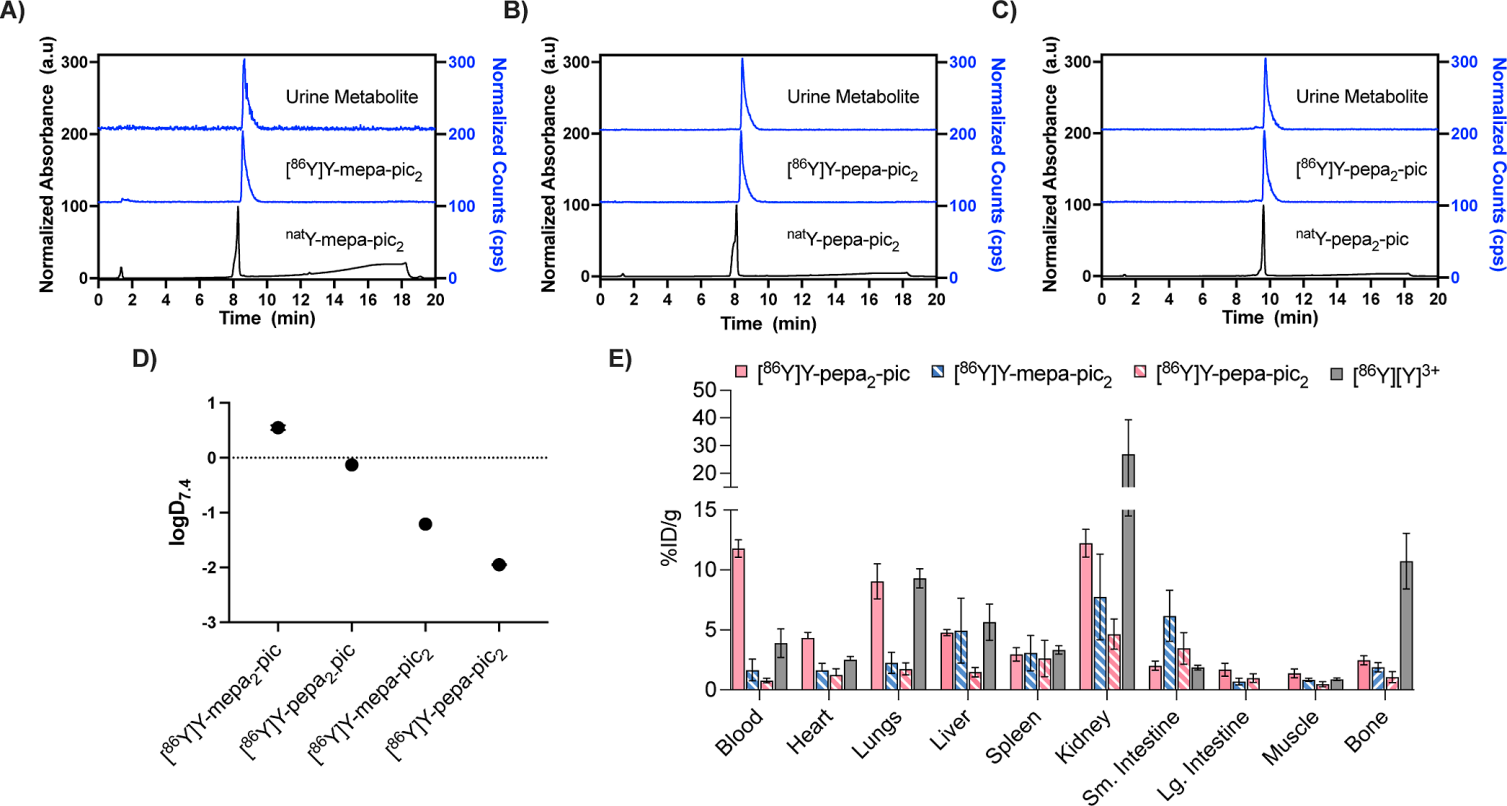
Chromatographic analysis of ^86^Y-labeled tracers, their corresponding nonradioactive complexes and urine metabolite products found ex vivo for: (a) mepa-pic_2_; (b) pepa-pic_2_; and (c) pepa_2_-pic. (d) Experimental logD_7.4_ values to assess the pH-dependent lipophilicity profiles of the ^86^Y-labeled tracers. (e) Ex vivo biodistribution pattern of [^86^Y]­Y-mepa-pic_2_, [^86^Y]­Y-pepa-pic_2_, [^86^Y]­Y-pepa_2_-pic, and [^86^Y]­[Y]^3+^ (ref 46) 1 h post injection in BALB/c mice. Error bars represent ±1 standard deviation (*n* = 4). % ID/g of organ is the percentage of injected dose per gram of organ weight.

Biodistribution and urine metabolite analysis was conducted in naïve BALB/c mice at 60 min post injection. As shown in Figure 4e, [^86^Y]­Y-pepa_2_-pic showed enhanced blood retention (>10% ID/g), which also results in retention of activity in highly perfused organs such as the heart (4.34 ± 0.46% ID/g) and lungs (9.05 ± 1.46 ID/g). Elevated levels of tracer were also observed in the liver (4.78 ± 0.25% ID/g), likely due to the higher logD_7.4_ value (−0.13). In contrast, [^86^Y]­Y-mepa-pic_2_ exhibited a 7-fold reduction in tracer levels in the blood (1.66 ± 0.90% ID/g), over 2–4-fold in heart (1.63 ± 0.59% ID/g) and lungs (2.26 ± 0.88% ID/g), compared to [^86^Y]­Y-pepa_2_-pic. This marked decrease in retention aligns well with the lower lipophilicity of [^86^Y]­Y-mepa-pic_2_ (logD_7.4_ = −1.21). Finally, [^86^Y]­Y-pepa-pic_2_, showed the lowest retained activity in the blood (0.78 ± 0.19% ID/g), lungs (1.75 ± 0.50% ID/g), and liver (1.48 ± 0.39% ID/g), highlighting the enhanced pharmacokinetic profile associated with reduced lipophilicity (logD_7.4_ = −1.95). All radiotracers exhibited minimal retention in muscle tissue (<1.5%) and similarly low accumulation in bone—[^86^Y]­Y-mepa-pic_2_ (1.89 ± 0.38% ID/g), [^86^Y]­Y-pepa-pic_2_ (1.07 ± 0.46% ID/g), and [^86^Y]­Y-pepa_2_-pic (2.47 ± 0.38% ID/g). The difference in logD_7.4_ and biodistribution profile between [^86^Y]­Y-mepa-pic_2_ and [^86^Y]­Y-pepa-pic_2_ evidence the favorable impact of PEGylation of the antenna to boost hydrophilicity. Notably, among all chelates, [^86^Y]­Y-pepa-pic_2_ consistently showed the lowest retention in all nontarget tissues. These findings indicate high in vivo stability, as nonchelated [^86^Y]Y typically accumulates in liver and bone tissues, as depicted in Figure 4e.^44,45^ This conclusion was further supported by metabolite analysis, which revealed that >98% of the injected tracers remained intact (Figure 4), with no detectable unchelated [^86^Y]­Y^3+^. In our previous work, [^86^Y]­Y^3+^ was otherwise observed to appear in urine as early as 60 min post injection.^44^ Overall, these results identify the pepa-pic_2_ scaffold as the best-suited candidate for further investigation as a systemically administered, theranostic platform.

### Conjugate Synthesis and In Vitro Evaluation

We next targeted the synthesis of a targeting-vector-functionalized analogue of pepa-pic_2_; specifically, we sought to introduce Hex-KuE, a peptide with high affinity to the prostate-specific membrane antigen (PSMA). To this end, we employed our previously validated electrophilic substitution of nitro-picolinate with a cysteine thiol.^28,46^

To obtain the cysteine-reactive chelator, we employed a stepwise introduction of the three different picolyl arms. N-alkylation of N-Boc-tacn with bromo-picolinic acid ethyl ester, affording 7 in 32% yield, followed by alkylation with nitro-substituted bromo-picolinic acid ethyl ester (NO_2_Pic), yielded 10 in 53% yield. The resulting intermediate was then treated under acidic conditions to remove the Boc-protecting group and carry out a final N-alkylation step with the protected pyridylalkynylaryl moiety to install the Eu-sensitizing antenna (Scheme S3). Basic hydrolysis of all ethyl ester-protecting groups was performed prior to peptide bioconjugation, as the targeting vector is susceptible to degradation under basic conditions. As a targeting vector, we selected the Hex-KuE peptide, which is readily synthesized using solid-phase peptide synthesis.^47^ The N-terminus was modified with a cysteine for compatibility with electrophilic aromatic substitution to the nitro-picolinate. Indeed, the formation of the thio-ether can be achieved at room temperature in a DMF/NH_4_HCO_3_ buffer mixture using a metal-free chelator (Figure 5a). Subsequently, aqueous complexation reactions with any rare-earth metal ion of choice (Eu, Lu, Y, and Tb) afford the desired M-pepa-pic_2_-C-hex-KuE product in quantitative yield.

**Figure 5:**
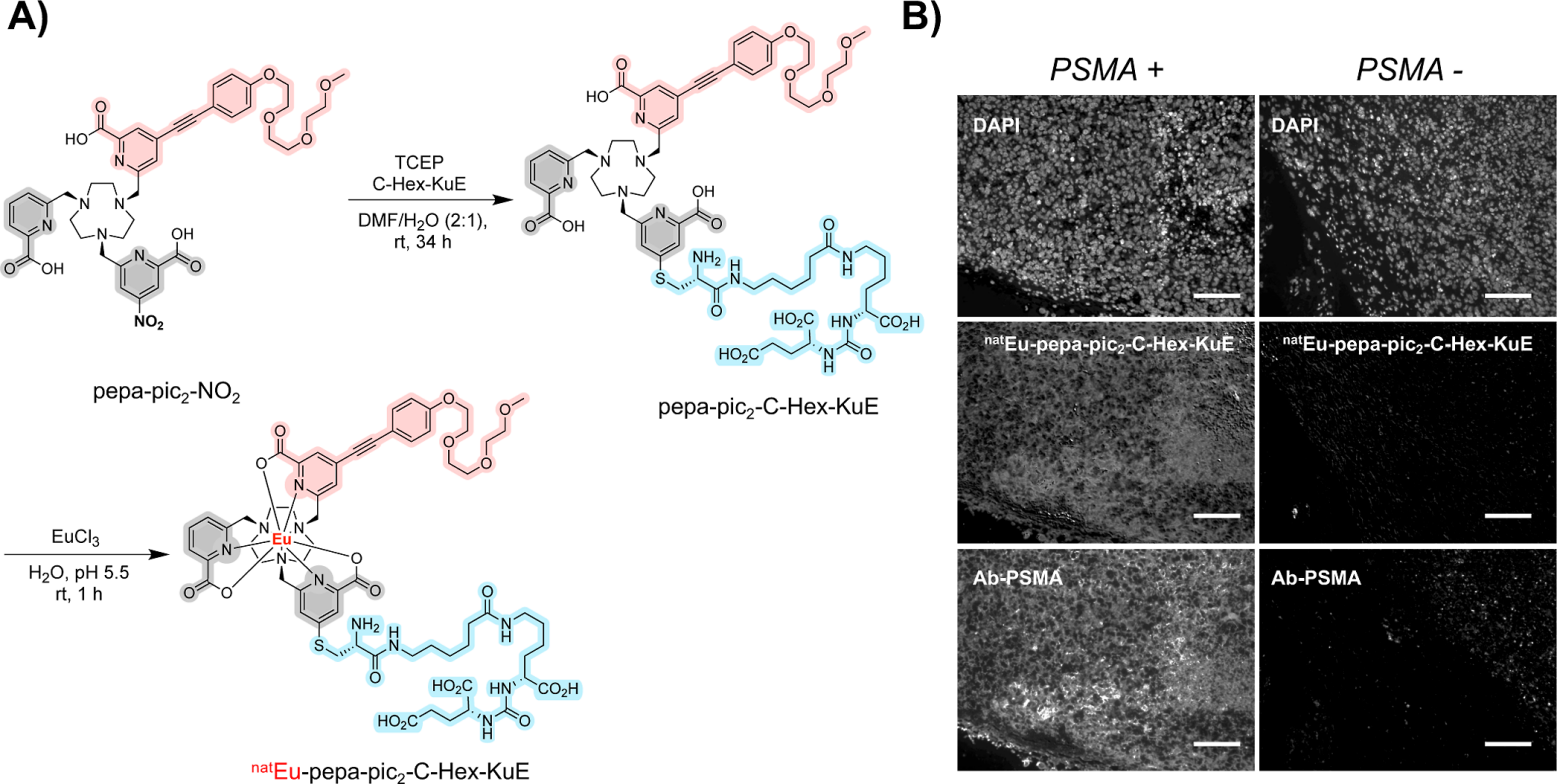
(A) Synthesis of Eu-pepa-pic_2_-C-Hex-KuE. (B) Optical imaging of tumor tissues harvested from mice-bearing xenografts from PC3-PIP (PSMA-positive) and PC3-flu (PSMA-negative). Sample tissues were stained with DAPI, Eu-pepa-pic_2_-C-Hex-KuE, or Ab-PSMA. Histological images taken using a 20× objective. Scale bar: 100 μm.

To demonstrate that the Eu-pepa-pic_2_-C-hex-KuE complex efficiently retained an affinity for the biological target, we conducted immunohistochemical experiments with PC-3 cell tissue samples grown and excised from nude mice. Specifically, we prepared sections of PC-3 PIP (PSMA-expressing) and PC-3 flu cells and incubated samples with Eu-pepa-pic_2_-C-hex-KuE or a fluorescein-linked, PSMA-binding antibody (Ab-PSMA) overnight. Nuclear staining to visualize cell localization was conducted with DAPI (ex: 365 nm, em: 450 nm). Indeed, Eu-pepa-pic_2_-C-hex-KuE (ex: 365 nm, em: 620 nm) showed an imageable signal and discernible cell features that correlated well with antibody-based immunohistochemistry staining. Conversely, the non-PSMA-expressing tissue did not show a detectable signal for Eu-pepa-pic_2_-C-Hex-KuE or the PSMA antibody (Figure 5b). This evidence shows that the functionalization of the KuE peptide with the pepa-pic_2_-chelator retained the affinity to PSMA-expressing cells and did not result in significant nonspecific binding to cells.

### Validation of the Diagnostic Modality

With the affinity to PSMA-expressing cells validated, we next sought to investigate whether the [^86^Y]­Y-pepa-pic_2_-C-hex-KuE could be readily formed and serve as a diagnostic probe following systemic administration (Figure 6a). Although Y-86 is a nonconventional positron-emitting radionuclide, it has garnered considerable interest in nuclear medicine due to its relatively long half-life (*t*_1/2_ = 14.7 h), especially when compared to commonly used PET isotopes such as ^68^Ga (*t*_1/2_ = 68 min) and ^18^F (*t*_1/2_ = 109.8 min).^48^ This extended half-life enables imaging at later time points and shipping to remote locations without direct access to short-lived PET nuclides.

**Figure 6:**
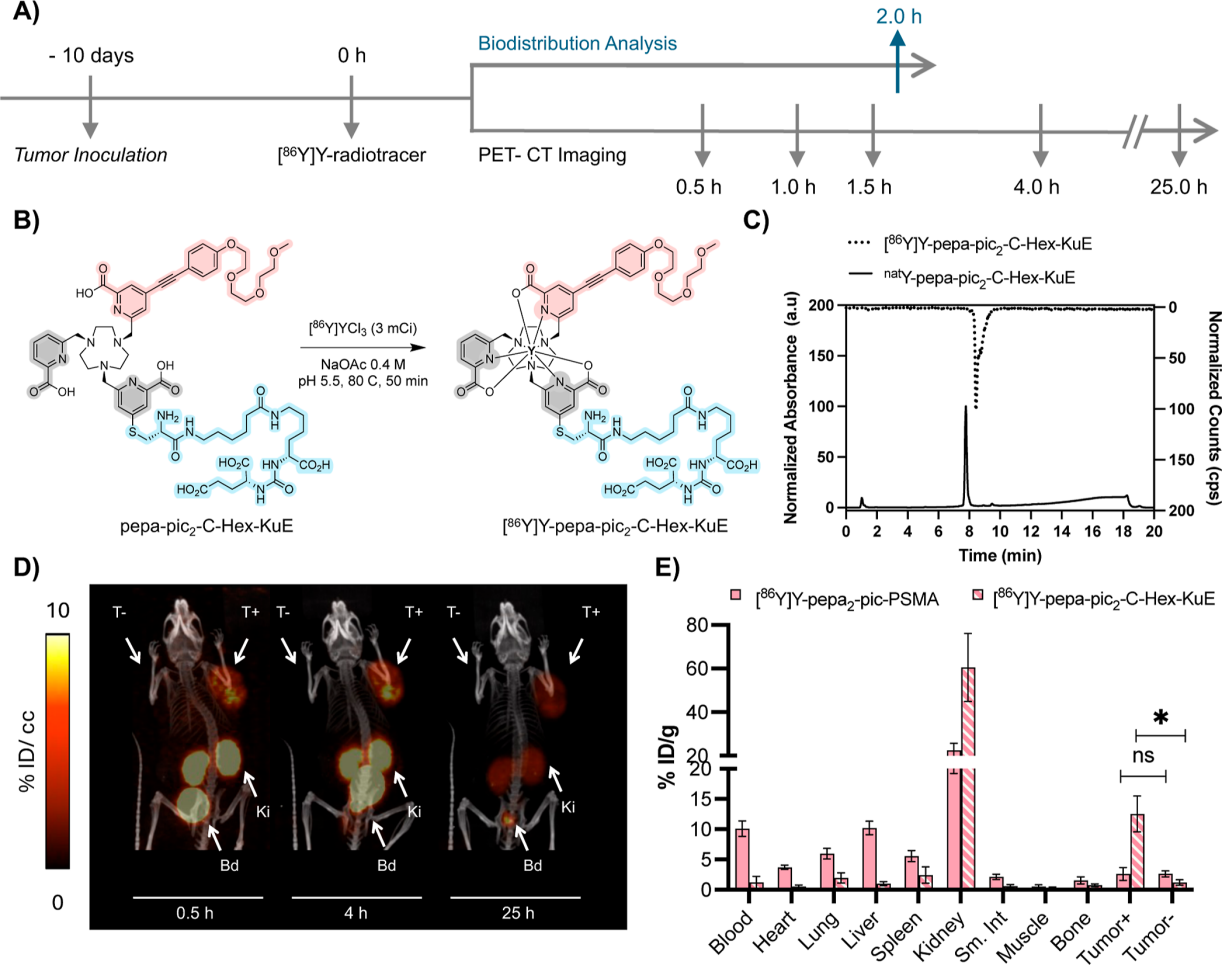
(A) Descriptive timeline of the animal study. (B) Scheme for the optimized radiosynthesis of [^86^Y]­Y-pepa-pic_2_-C-Hex-KuE. (C) Chromatographic analysis of [^86^Y]­Y-pepa-pic_2_-C-Hex-KuE and its corresponding nonradioactive complex. (D) Longitudinal in vivo PET/CT imaging of [^86^Y]­Y-pepa-pic_2_-C-Hex-KuE 0.5, 4, and 25 h post administration of radiotracer to nude mice-bearing PSMA ± tumors. Images are normalized to units of % ID/cc and presented as maximum intensity projection scans (MIPS). Activity is primarily shown in the bladder (Bd), kidneys (Ki), and PSMA-positive tumor (T+). % ID/cc is the percentage of the injected dose per cubic centimeter. (E) Comparison of the ex vivo biodistribution profile of [^86^Y]­Y-pepa-pic_2_-C-Hex-KuE and [^86^Y]­Y-pepa_2_-pic-PSMA 2 h post injection. Error bars represent ±1 standard deviation (*n* = 4). % ID/g of organ is the percentage of injected dose per gram of organ weight. Statistical differences were calculated using two-tailed Student’s *t* tests assuming unequal variances; *p = 0.004.

Radiolabeling to afford [^86^Y]­Y-pepa-pic_2_-C-hex-KuE was performed under conditions similar to those used for the nontargeted chelators evaluated in the naïve biodistribution studies (vide supra): the resulting complex was obtained with a high radiochemical yield and acceptable molar activity (96 mCi/μmol), requiring no additional purification due to >98% radiochemical purity (Figure 6b,c).

For in vivo evaluation, athymic nude mice-bearing bilateral PC-3 PIP/flu (PSMA^+^/PSMA^–^) tumor xenografts were used (Figure 6). This model is well established for studying radiopharmaceuticals for prostate cancer in a preclinical setting and has been validated previously for intratumoral CRET imaging by our lab. [^86^Y]­Y-pepa-pic_2_-C-hex-KuE was administered to mice intravenously, and PET/CT imaging was conducted at 0.5 h, 1 h, 1.5 h, 4 h, and up to 25 h (Figure 6d, Supporting Information, Figure S51). At 0.5 h post-injection, elevated levels of the radiotracer in the PSMA+ tumor are observed, whereas no significant accumulation was observed in the PSMA- tumor. A significant fraction of the tracer is localized in the kidneys and bladder, consistent with the rapid renal clearance of the tracer. Region-of-interest (ROI) analysis of the PET/CT images (Figure S51 and Table S3) revealed that there is a sustained uptake of the tracer in the PSMA+ site of the tumor (5.2 ± 1.7% ID/cc) in the first 4 h, after which tumor uptake decreases as observed at the 25 h post injection time point (3.3 ± 1.0% ID/cc). Similarly, activity in the kidneys, muscle, heart, bone, and liver decreases as the tracer is excreted (Table S3).

Ex vivo biodistribution analysis of the radiotracer was conducted 2 h post injection and compared to that of [^86^Y]­Y-pepa_2_-pic-PSMA, as previously reported by our group (Figure 6e).^28^ Imaging and biodistribution data show good correlation, with high levels of activity retained in kidneys (60.48 ± 15.62% ID/g) revealing renal clearance. Throughout, <2% ID/g was observed in the liver, blood, heart, and lungs, indicating a low lipophilic character, in good correlation with the logD_7.4_ (−2.98). Compared to [^86^Y]­Y-pepa_2_-pic-PSMA,^28^ these results represent a substantial improvement of pharmacokinetic properties (Figure 6e). Urine metabolite analysis showed no significant degradation of the probe, with >93% remaining intact. The in vivo compatibility and complex inertness are further evidenced by low bone and liver uptake.

Overall, the results demonstrate the significant pharmacokinetic improvement presented by the pepa-pic_2_-C-Hex-KuE platform, rendering it ideal for both intratumoral and systemic administration.

### Validation of the Optical Imaging Modality

With the favorable pharmacokinetics of [^86^Y]­Y-pepa-pic_2_-C-hex-KuE established, we evaluated the potential of Eu-pepa-pic_2_-C-Hex-KuE as a luminescent probe administered in combination with a positron-emitting radiopharmaceutical as an in situ Cerenkov radiation source. Based on our in vitro CRET experiments with ^nat^Eu-pepa-pic_2_, we had determined that colocalization of 10 μCi ^68^Ga with 1 nmol of ^nat^Eu-pepa-pic_2_ provided signal intensity distinguishable from the background Cerenkov luminescence (Figure 3).

We first validated intratumoral injection of 20 nmol of Eu-pepa-pic_2_-C-Hex-KuE combined with systemic, intravenous administration of 290 μCi of the clinically validated radiopharmaceutical [^68^Ga]­Ga-PSMA-617. Under these conditions, we observed significant signal enhancement in the tumor receiving Eu-pepa-pic_2_-C-Hex-KuE, with the signal remaining statistically significantly enhanced even after two half-lives of the Cerenkov-emitting radioisotope. This affirmed that intrasurigcal, in situ administration and reimaging of resected tissue was readily feasible (Figure 7).

**Figure 7:**
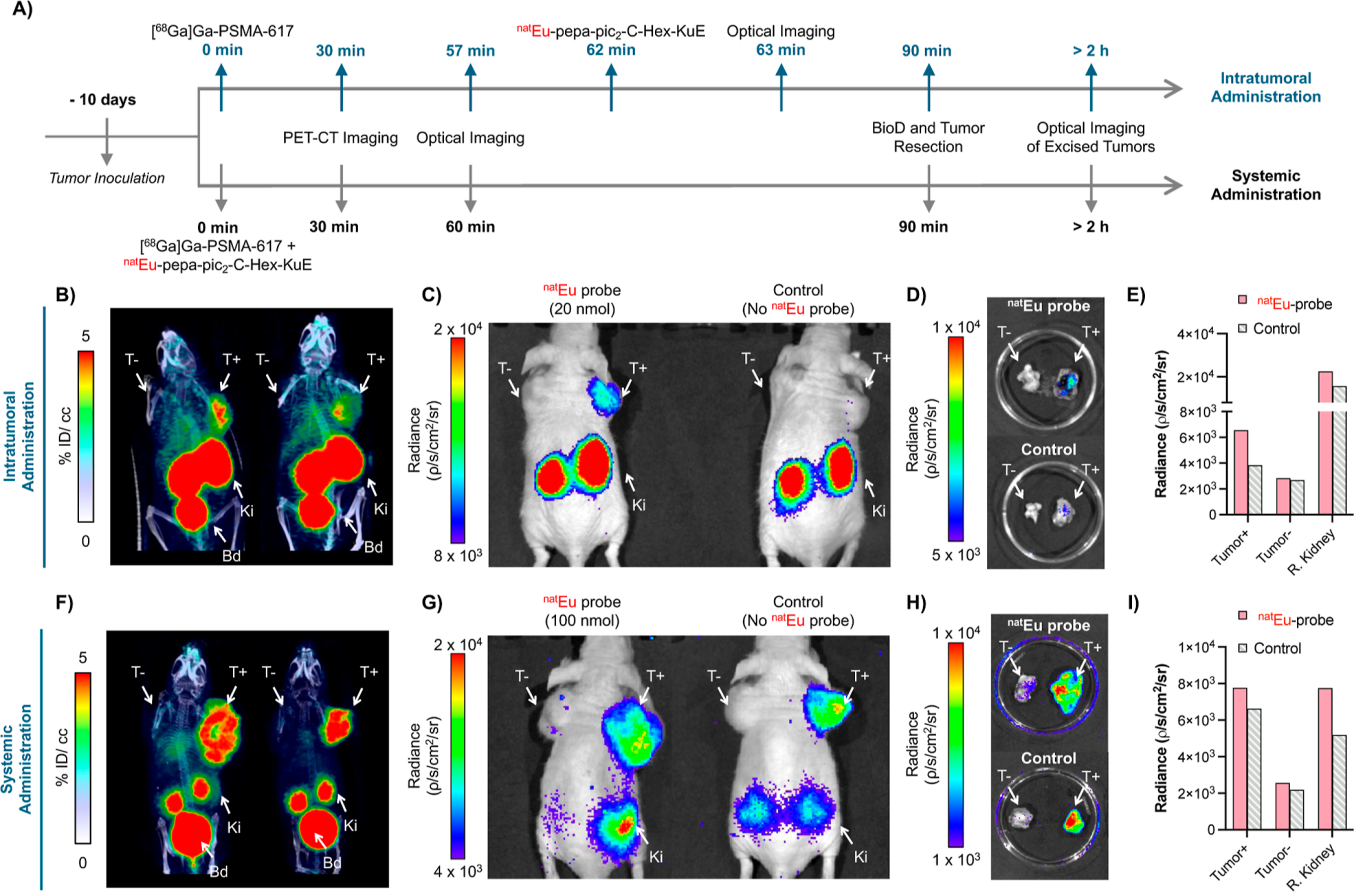
(A) Descriptive timeline of the in vivo optical imaging study with intratumoral and systemic administration of the luminescent probe Eu-pepa-pic_2_-C-Hex-KuE. (B) PET/CT imaging of [^68^Ga]­Ga-PSMA-617 (Cerenkov source) 30 min post administration of radiotracer to nude mice-bearing PSMA ± tumors. Images are normalized to units of %ID/cc and presented as MIPS. Activity is primarily shown in the bladder (Bd), kidneys (Ki), and PSMA+ (T+). % ID/cc is the percentage of injected dose per cubic centimeter. (C) Presurgical optical imaging of mice post PET/CT imaging from (b). Mouse on the left was injected with ^nat^Eu-pepa-pic_2_-C-Hex-KuE (20 nmol, Eu^3+^-probe delivered intratumorally) in the PSMA+ tumor (right shoulder). Mouse on the right side (control) did not receive the luminescent Eu-based probe. (D) PSMA ± tumors excised post optical imaging. (E) Region of interest analysis of the luminescence signals of selected organs from images shown in (C). (F) PET/CT imaging of nude mice-bearing PSMA ± tumors 30 min post coadministration of [^68^Ga]­Ga-PSMA-617 (Cerenkov source) and Eu-pepa-pic_2_-C-Hex-KuE (100 nmol, ^nat^Eu-probe) via intravenous injection (left mouse). Right mouse (control) did not receive the luminescent Eu^3+^ probe. (G) Presurgical optical imaging of mice post PET-CT imaging from (F). (H) PSMA ± tumors excised post optical imaging from (G). (I) Region of interest analysis of the luminescence signals of selected organs from images shown in (G).

We probed the systemic coadministration of the Cerenkov emitter and Eu^3+^ complex next. To this end, we first approximated the appropriate dose of both probes to achieve a detectable signal. Taking into consideration that most PSMA-targeted peptide conjugates show a 10% ID/g uptake at the target tumor site at 1–2 h post injection, we selected to administer 100 nmol of Eu-pepa-pic_2_-C-Hex-KuE combined with 295 μCi of [^68^Ga]­Ga-PSMA-617. Under these conditions, however, we were unable to observe a statistically significant enhancement of the optical signal in the tumor. The luminescence output strongly depends on the efficient and simultaneous colocalization of high concentrations of both the Cerenkov emitter [^68^Ga]­Ga-PSMA-617 and the red-luminescent probe ^nat^Eu-pepa_2_-pic-C-Hex-KuE within the target tissue. Since both agents are designed to bind the same membrane receptors, their accumulation is mutually competitive, which can partially diminish the optical enhancement at the tumor site when systemically administered compared to local administration. Covalently linking the Cerenkov emission source directly to the Eu-probe moiety could potentially yield a greater optical enhancement by eliminating competition between the two separate components. Notably, we observed statistically significant optical signal enhancement in the kidneys, which may indicate that the enhanced colocalization of the Eu^3+^ complex and the tracer results in above background/threshold optical signal. This is further supported by the imaging and biodistribution data, which show that [^68^Ga]­Ga-PSMA-617 and [^86^Y]­Y-pepa_2_-pic-C-Hex-KuE show a 6–10-fold enhancement of probe localization in kidneys when compared to tumor tissue.

### Compatibility with Beta Therapy-Avid Radionuclides

The β^–^ emitting isotope ^177^Lu^3+^ (*t*_1/2_ = 160 h, E_β‑_ = 149 keV) has become a standard in clinical radionuclide therapy, offering medium-energy β^–^ emissions. Alternatively, ^161^Tb^3+^ (*t*_1/2_ = 166 h, *E*_β‑_ = 154 keV), a promising next-generation alternative, emits β^–^ particles with energies similar to those of Lu-177 but additionally releases abundant low-energy conversion and Auger electrons. These short-range emissions can deliver highly localized cytotoxicity, making ^161^Tb particularly effective against micrometastases and single cancer cells that may be less responsive to conventional β^–^ therapy. With pepa-pic_2_-C-Hex-KuE readily compatible with the small, rare-earth Y and the medium-sized lanthanide Eu, we sought to probe the compatibility with ^177^Lu and ^161^Tb, respectively. The formation of [^177^Lu]­Lu-pepa-pic_2_-C-Hex-KuE and [^161^Tb]­Tb-pepa-pic_2_-C-Hex-KuE was achieved under identical reaction conditions as previously employed for the ^86^Y analogue (80 °C, pH 5.5), producing the corresponding radiolabeled products with 38.7 and 29.4 mCi/μmol molar activity, specifically (Figure 8bc, Supporting Information, Figure S47). Following radiochemical labeling and formulation in PBS, [^177^Lu]­Lu-pepa-pic_2_-C-hex-KuE and [^161^Tb]­Tb-pepa-pic_2_-C-hex-KuE were evaluated by single point biodistribution (2 h p.i.) and serial SPECT imaging at 0.5, 1, 1.5, 4, and 25 h in direct comparison with the clinically validated radiopharmaceutical [^177^Lu]­Lu-PSMA-617 as well as [^161^Tb]­Tb-PSMA-617 (Figure 8d–e, Supporting Information, Figures S52–S55, and Table S2).

**Figure 8:**
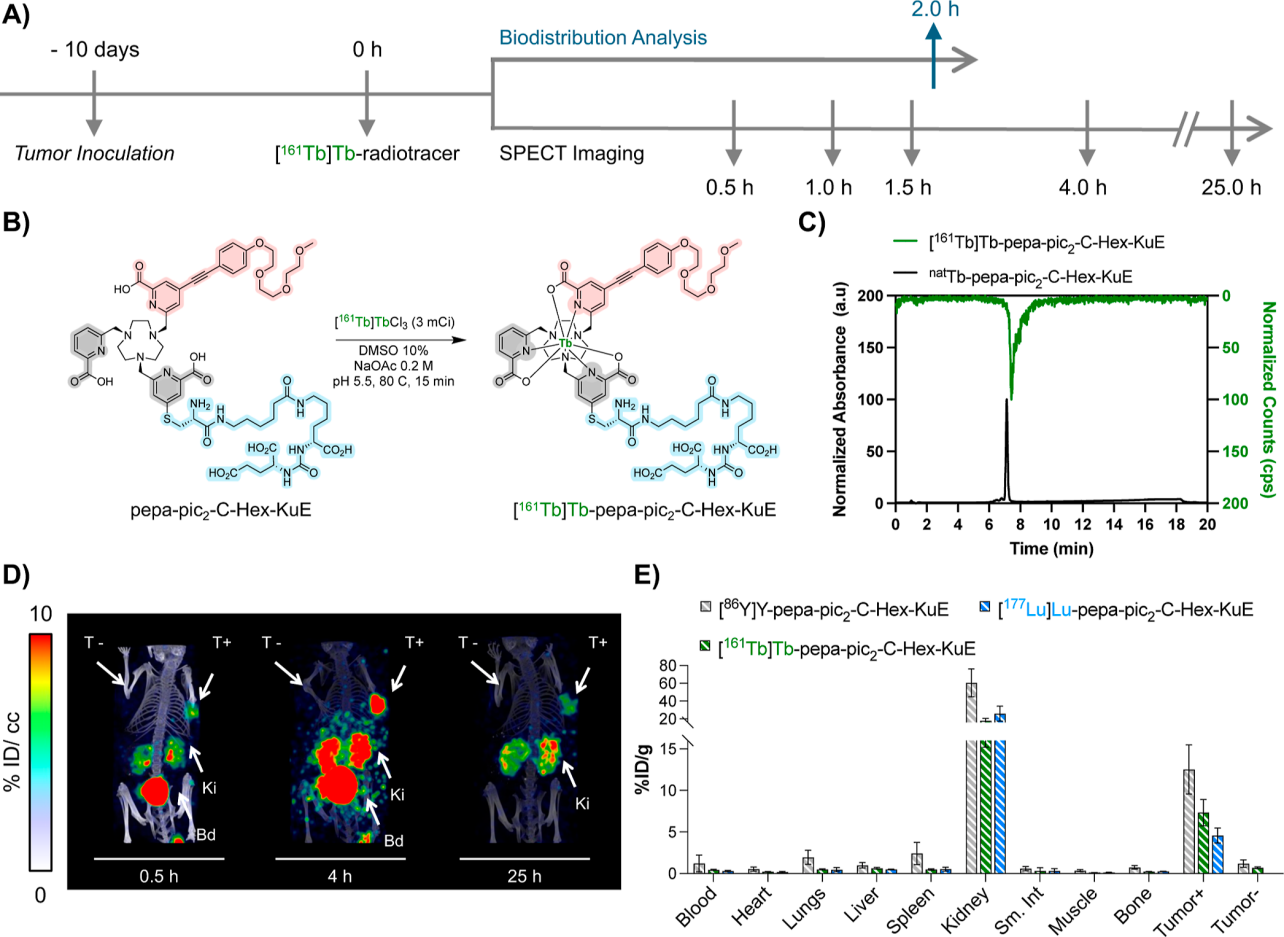
(A) Descriptive timeline of the animal study. (B) Scheme for the optimized radiosynthesis of [^161^Tb]­Tb-pepa-pic_2_-C-Hex-KuE. (C) Chromatographic analysis of [^161^Tb]­Tb-pepa-pic_2_-C-Hex-KuE and its corresponding nonradioactive complex. (D) Longitudinal in vivo SPECT imaging of [^161^Tb]­Tb-pepa-pic_2_-C-Hex-KuE 0.5, 4, and 25 h post administration of radiotracer to nude mice-bearing PSMA ± tumors. Images are normalized to units of % ID/cc and presented as MIPS. Activity is primarily shown in the bladder (Bd), kidneys (Ki), and PSMA-positive tumor (T+). % ID/cc is the percentage of injected dose per cubic centimeter. (E) Comparison of the ex vivo biodistribution profile of pepa-pic_2_-C-Hex-KuE radiolabeled with ^161^Tb, ^177^Lu, and ^86^Y 2 h post injection. Error bars represent ±1 standard deviation (*n* = 4). % ID/g of organ is the percentage of injected dose per gram of organ weight.

In vivo experiments in the PSMA ± tumor model (described vide supra) demonstrated a good correlation between the performance of [^86^Y]­Y-pepa-pic_2_-C-hex-KuE and its two therapeutic congeners [^177^Lu]­Lu-pepa-pic_2_-C-hex-KuE and [^161^Tb]­Tb-pepa-pic_2_-C-hex-KuE, with persistent, statistically significant uptake of activity in the PSMA-expressing tumor and predominant renal clearance. In comparison with [^177^Lu]­Lu-PSMA-617, prolonged kidney retention was observed, but no deposition in any other nontarget organs. These results indicate that the pepa-pic_2_ platform forms in vivo compatible, inert complexes with rare-earth ions with disparate ionic radii ranging from 1.032 to 1.12 Å.

## Conclusions

In this work, we developed and systematically evaluated a new generation of Eu^3+^-based molecular probes built upon modified polyazamacrocyclic-chelating scaffolds bearing pyridylalkynylaryl antennae. By tuning the sensitizing chromophores, we achieved complexes with reduced lipophilicity, improved aqueous solubility, and favorable pharmacokinetics while maintaining desirable photophysical properties. The structure–activity relationship study of four pyridylalkynylaryl antenna-bearing chelators revealed that incorporation of two pyridylalkynylaryl antennae does not significantly contribute to an enhancement of the luminescence quantum yields but instead increases hydrophobicity and compromises in vivo compatibility.

CRET imaging experiments further demonstrated that single-pyridylalkynylaryl constructs are sufficient for robust luminescence under clinically relevant photon flux conditions, while radiolabeling and biodistribution studies with ^86^Y established the superior hydrophilicity and rapid renal clearance of [^86^Y]­Y-pepa-pic_2_ relative to other analogs. Conjugation to a PSMA-targeting vector yielded [^86^Y]­Y-pepa-pic_2_-C-hex-KuE, which preserved specific binding to PSMA-expressing tumors and exhibited high in vivo stability, rapid clearance, and low off-target retention. PET/CT and optical imaging studies validated the probe’s dual-modality potential, with statistically significant tumor-to-background contrast and compatibility with in situ Cerenkov excitation. When systemically administered, our Eu-based probe provides a modest yet detectable enhancement of optical luminescence. Further optimization, specifically through covalent incorporation of a Cerenkov radiation source into the probe molecular scaffold, could significantly improve the probe’s sensitivity. Ongoing work in our laboratory is currently focused on achieving this goal. Finally, radiolabeling with therapeutic isotopes ^177^Lu^3+^ and ^161^Tb^3+^ confirmed the platform’s expanded, rare-earth ion compatibility and established its promise as a theranostic agent bridging diagnostic nuclear, optical imaging, and radionuclide therapy.

Overall, these findings highlight, for the first time, the potential of an all-rare-earth-based theranostic, molecular approach to diagnosis, surgical resection, and radiotherapeutic management of disease.

## Supplementary Material



## Notes

Experimental procedures and compound characterization (PDF)
