# Supplementary material for: A Trifunctional, Rare-Earth Theranostic Chelator Platform to Enable Diagnostic Nuclear Imaging, Surgical Resection, and Radiotherapy
Source: J Am Chem Soc. 2025 Dec 1;147(49):45303–14. doi: 10.1021/jacs.5c15147 (PMC12703665; doi:10.1021/jacs.5c15147)
Supplement: Supplementary file 1 [file ja5c15147_si_001.pdf]

Supporting Information for:

A Trifunctional, Rare-Earth Theranostic Chelator Platform to Enable Diagnostic Nuclear Imaging, Surgical Resection and Radiotherapy

M. Andrey Joaqui-Joaqui,[a] Georgia G. Sands,[a] Dariusz Śmiłowicz,[a] Mallory J. Gork,[a] Eduardo Aluicio-Sarduy,[b] Todd E. Barnhart,[b] Jonathan W. Engle[b,c] and Eszter Boros[a*].

[a] Department of Chemistry, University of Wisconsin Madison, 1101 University Avenue, Madison, Wisconsin, 53705, United States

[b] Department of Medical Physics, University of Wisconsin-Madison, 1111 Highland Avenue, Madison, Wisconsin 53705, United States

[c] Department of Radiology, University of Wisconsin-Madison, Madison, Wisconsin 53705, United States

Table of Contents

1 General Considerations 4
1.1 Spectroscopy and Mass Spectrometry Methods 4
1.2 High Performance Liquid Chromatography Methods 4
1.2.1 Semipreparative HPLC: 4
1.2.2 Analytical HPLC: 4
1.2.3 Analytical RadioHPLC 5
1.2.4 Liquid chromatography mass spectrometry (LCMS) 5
2 Chemical synthesis 6
2.1 Synthesis of model ligands 6
2.1.1 Synthesis of **2** 6
2.1.2 Synthesis of **3** 7
2.1.3 Synthesis of **4** and **5** 7
2.1.4 Synthesis of **6** 8
2.1.5 Synthesis of **mepa-pic$_2$** 9
2.1.6 Synthesis of **^{nat}Eu-mepa-pic$_2$** 10
2.1.7 Synthesis of **^{nat}Y-mepa-pic$_2$** 11
2.1.8 Synthesis of **mepa$_2$-pic** 12
2.1.9 Synthesis of **^{nat}Eu-mepa$_2$-pic** 13
2.1.10 Synthesis of **7-8** 15
2.1.11 Synthesis of **9** 16
2.1.12 Synthesis of **pepa-pic$_2$** 17
2.1.13 Synthesis of **^{nat}Eu-pepa-pic$_2$** 18

2.1.14 Synthesis of **^{nat}Y-pepa-pic$_2$** 19
2.1.15 Synthesis of **C-Hex-KuE** peptide 21
2.1.16 Synthesis of **10** 22
2.1.17 Synthesis of **11** 23
2.1.18 Synthesis of **12** 23
2.1.19 Synthesis of **pepa-pic$_2$-C-Hex-KuE** 24
2.1.20 Synthesis of ^{nat}Eu-pepa-pic$_2$-C-Hex-KuE 25
2.1.21 Synthesis of ^{nat}Lu-pepa-pic$_2$-C-Hex-KuE 26
2.1.22 Synthesis of ^{nat}Tb-pepa-pic$_2$-C-Hex-KuE 27
2.2 Complex synthesis and characterization (photophysical characterization) 27
2.2.1 Molar absorptivity determination of ligands **mepa-pic$_2$, mepa$_2$-pic, pepa-pic$_2$ and pepa$_2$-pic**. 27
2.2.2 Molar absorptivity determination of Eu-complexes. 32
2.2.3 Lifetimes and determination of the number of water molecules coordinated to Europium(III) 33
2.2.4 Luminescence Quantum Yields 35
3 Cerenkov Radiation-mediated Energy Transfer Phantom Imaging Assays 41
4 Radiolabeling Studies 42
4.1 Radiochemical synthesis and characterization of non-targeted complexes with Y-86. 42
4.1.1 Radiosynthesis of [^{86}Y]Y-mepa-pic$_2$ 42
4.1.2 Radiosynthesis of [^{86}Y]Y-pepa-pic$_2$ 42
4.1.3 Radiosynthesis of [^{86}Y]Y-pepa$_2$-pic 43
4.2 Radiochemical synthesis and characterization of targeted complexes of pepa-pic$_2$-C-Hex-KuE with Y-86, Lu-177 and Tb-161. 44
4.2.1 Radiosynthesis of [^{86}Y]Y-pepa-pic$_2$-C-Hex-KuE 44
4.2.2 Radiosynthesis of [^{177}Lu]Lu-pepa-pic$_2$-C-Hex-KuE 44
4.2.3 Radiosynthesis of [^{161}Tb]Tb-pepa-pic$_2$-C-Hex-KuE 45
4.2.4 Radiosynthesis of [^{177}Lu]Lu-PSMA-617 45
4.2.5 Radiosynthesis of [^{161}Tb]Tb-PSMA-617 46
4.3 LogD$_{7.4}$ Lipophilicity Measurements 46
5 In vivo studies 47
5.1 In vivo biodistribution of non-targeted constructs in naïve BALB/c mice 47
5.1.1 Urine metabolite analysis 48
5.2 *In vitro* optical imaging 48
5.3 In vivo biodistribution in PSMA +/- mice models, PET-CT and SPECT imaging. 48
5.3.1 Urine metabolite analysis 53

5.4 In vivo CRET optical imaging 54
5.4.1 Intratumoral Administration 54
5.4.2 Systemic Administration 55
5.5 Characterization data and spectroscopy of ligands 56
6 References 95

1 General Considerations

Unless otherwise noted, all starting materials were purchased from commercial sources and used without further purification.

1.1 Spectroscopy and Mass Spectrometry Methods

Nuclear Magnetic Resonance (NMR) Spectroscopy: All NMR data was collected on a Bruker Avance-500 spectrometer at the University of Wisconsin-Madison Department of Chemistry Paul Bender Chemical Instrumentation Center (CIC). Chemical shifts are reported as parts per million (ppm) and are referenced relative to TMS or residual solvent peaks. Deuterated solvents were obtained from Cambridge Isotope Laboratories (Tewskbury, MA, USA).

Mass spectrometry: Mass spectrometry data was collected on a Thermo Scientific Q Exactive Focus Orbitrap MS system at the University of Wisconsin-Madison Department of Chemistry Paul Bender Chemical Instrumentation Center (CIC).

Ultraviolet-Visible (UV-Vis) spectra: UV−visible spectra were recorded on a NanoDrop One^{C} instrument (AZY1706045) with 1 cm quartz cuvettes using sodium acetate buffer (10 mM, pH 5.5) as solvent unless otherwise stated.

Luminescence Spectra: Luminescence spectra were recorded on a Hitachi F-7100 FL spectrophotometer with 1 cm path length quartz cuvettes with four polished windows. Selection of solvents and instrument parameters are described in sections 2.2.3 and 2.2.4 where evaluation of luminescence quantum yields and lifetimes are described.

1.2 High Performance Liquid Chromatography Methods

1.2.1 Semipreparative HPLC:

Purification of precursors and final ligands was carried out using an Agilent 1260 Infinity II system equipped with a binary gradient pump, a manual injection loop, and a UV-detector set to detect UV absorption at 220 nm and 254 nm.

Method A: Gradient: 0-3 min: 5% B; 3-17 min: 5-50% B; 17-21 min: 50-95% B; 21-25 min: 95% B; 25-27 min: 95-5% B; 27-30: 5% B. A = water + 0.1% FA; B = MeCN + 0.1% FA.
Column: Phenomenex Luna® C18 column (5 µm, 250 mm × 21.2 mm, 100 Å) at a flow rate of 30 mL/min.

Method B: Gradient: 0-3 min: 5% B; 3-21 min: 5-95% B; 21-25 min: 95% B; 25-27 min: 95-5% B; 27-30: 5% B. A = water + 0.1% FA; B = MeCN + 0.1% FA.
Column: Phenomenex Luna® C18 column (5 µm, 250 mm × 10 mm, 100 Å) at a flow rate of 10 mL/min.

1.2.2 Analytical HPLC:

Purification and analytical HPLC analysis of metal complexes and ligands was carried out using an Agilent 1260 Infinity II system equipped with a binary gradient pump, an autosampler, and a UV-detector set to detect UV absorption at 220 nm and 254 nm.

Method C: Gradient: 0-2 min: 5% B; 2-16 min: 5-95% B; 10-12 min: 95% B; 16-19 min: 95-5%; B; 19-20 min: 5%. A = water + 0.1% FA; B = MeCN + 0.1% FA.
Column: Phenomenex Luna® C18 column (5 µm, 150 mm × 3 mm, 100 Å) at a flow rate of 0.8 mL/min

1.2.3 Analytical RadioHPLC

Analytical HPLC of radiolabeled complexes and non-radioactive references were analyzed on an Agilent 1260 Infinity II system equipped with a binary gradient pump, an autosampler, and a UV-detector set to detect UV absorption at 220 nm and 254 nm. The instrument was coupled to a LabLogic 1" NaI photomultiplier tubedetector with 2" lead shielding to enable RadioHPLC analyses.

Method D: Gradient: 0-2 min: 5% B; 2-14 min: 5-95% B; 14-16 min: 95% B; 16-16.5 min: 95-5% B; 16.5-20 min: 5%. A = water + 0.1% FA; B = MeCN + 0.1% FA (unless otherwise stated).
Column: Phenomenex Luna® C18 column (5 µm, 150 mm × 3 mm, 100 Å) at a flow rate of 0.8 mL/min.

1.2.4 Liquid chromatography mass spectrometry (LCMS)

LCMS analysis was carried out on a Phenomenex Luna® C18 column (5 µm, 150 mm × 3 mm, 100 Å) at a flow rate of 0.8 mL/min using a single quadrupole Agilent 1200 Infinity II LC/MSD system equipped with a binary gradient pump, UV-vis detector, automatic injector, and an atmospheric pressure electrospray ionization (AP-ESI) source. Ultraviolet absorption was recorded at 220 nm and 254 nm, and positive and negative mass spectra were collected from m/z = 100-2000.

Method E:
Gradient: 0-3 min: 5% B; 3-10 min: 5-95% B; 10-12 min: 95% B; 12-12.5 min: 95-5%; B; 12.5-16 min: 5%. A = water + 0.1% FA; B = MeCN + 0.1% FA.
Column: Phenomenex Luna® C18 column (5 µm, 150 mm × 3 mm, 100 Å) at a flow rate of 0.8 mL/min.

2 Chemical synthesis

N-Boc-tacn,[1] PSMA-617,[2] Br-Pic,[3] NO_2-pic,[4, 5] intermediate (**1**), Br-pepa, as well as pepa-pic$_2$ and its corresponding Y^{3+} and Eu^{3+} complexes were prepared as previously described in the literature.[5]

2.1 Synthesis of model ligands

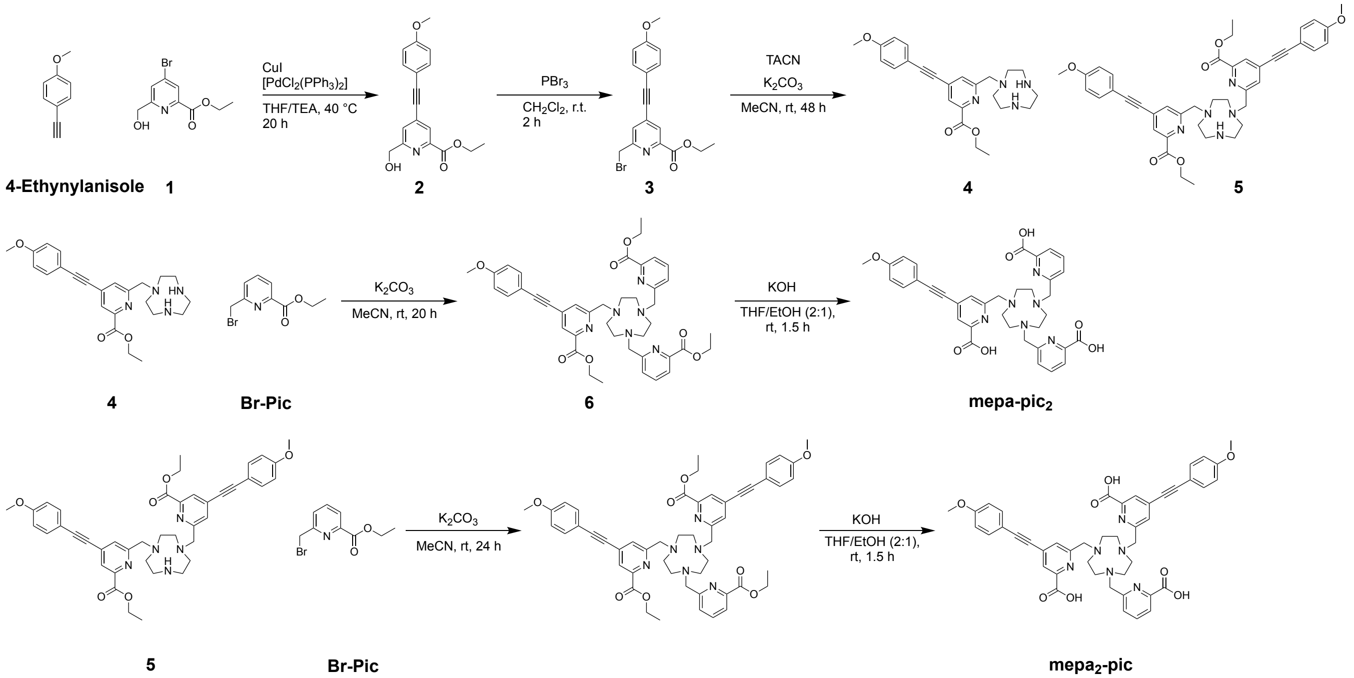

Scheme S1. Synthetic route used to afford **mepa-pic$_2$** and **mepa$_2$-pic**.

2.1.1 Synthesis of **2**

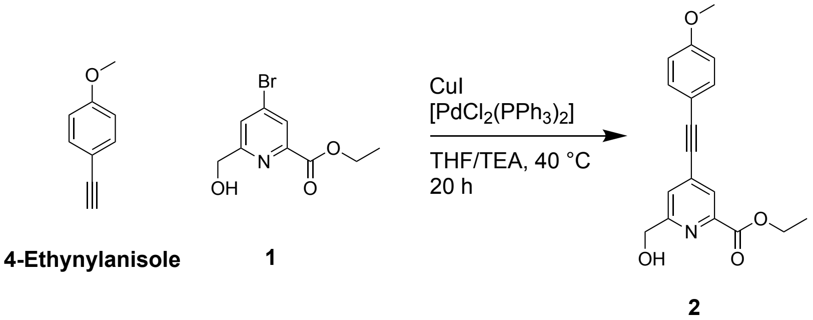

1-ethynyl-4-methoxybenzene (390 mg, 2.95 mmol) and ethyl 4-bromo-6-(hydroxymethyl)picolinate (778 mg, 2.99 mmol) were dissolved in freshly degassed THF: TEA (1:1, 11 mL). To this mixture,

[$(Ph_3P)_2PdCl_2$] (210 mg, 299 mmol) and CuI (113 mg, 593 mmol) were added under N_2 atmosphere and stirred while heating at 40 °C for 20 hours. The solvent was evaporated, the residue was resuspended in DCM (50 mL), washed with sat NH_4Cl (3 × 50 mL), brine (50 mL), dried over Na_2SO_4 and the solvent evaporated to yield a brown oil. Purification was carried out via silica gel flash chromatography using a gradient of EtOAc (30-100%) in hexanes. The title compound (499 mg, 295 mmol, 54%) was recovered as a brown oil.

^{1}H NMR (500 MHz, $CDCl_3$) δ 8.13 – 8.01 (m, 1H), 7.60 – 7.56 (m, 1H), 7.51 – 7.48 (m, 2H), 6.92 – 6.86 (m, 2H), 4.85 (s, 1H), 4.47 (q, J = 7.1 Hz, 1H), 1.44 (t, J = 7.1 Hz, 1H).

^{13}C NMR (126 MHz, $CDCl_3$) δ 164.84, 160.75, 160.42, 147.58, 133.82, 133.78, 125.72, 125.26, 114.39, 114.37, 95.85, 85.36, 64.57, 62.19, 55.51, 55.50.

ESI-MS: Calc m/z for $C_{18}H_{18}NO_4$ $[M+H]^+$: 312.1. Found: 312.1.

2.1.2 Synthesis of **3**

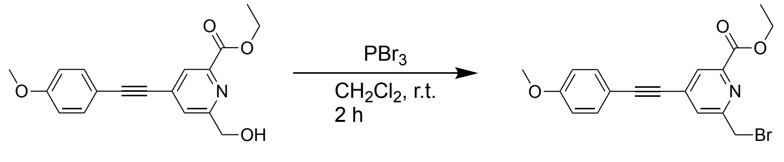

2 **3**

To a solution of intermediate **2** (401 mg, 1.29 mmol) in anhydrous dichloromethane (30 ml), PBr_3 (677 mg, 2.50 mmol) was dropwise added. The resulting mixture was stirred at room temperature for 2 hours. After this time, the crude was diluted with fresh dichloromethane (50 mL), washed with NaHCO3 (2 × 60 mL), once with brine, and dried over Na_2SO_4 to yield an oil. The latter was purified via silica gel flash chromatography using a gradient of EtOAc (0-50%) in hexanes to yield the desired compound as a white solid (yield: 483 mg, 32%)

^{13}H NMR (500 MHz, $CDCl_3$): δ 8.03 (d, J = 1.4 Hz, 1H), 7.68 (d, J = 1.4 Hz, 1H), 7.51 – 7.43 (m, 2H), 6.91 – 6.82 (m, 2H), 4.60 (s, 2H), 4.45 (q, J = 7.1 Hz, 2H), 3.80 (s, 3H), 1.41 (t, J = 7.1 Hz, 3H).

^{13}C NMR (126 MHz, $CDCl_3$) δ 164.49, 160.71, 157.60, 148.10, 134.30, 133.70, 128.25, 126.07, 114.30, 113.61, 96.23, 84.98, 62.21, 55.41, 33.00, 14.35.

ESI-HR-MS: Calc m/z for $C_{18}H_{17}BrNO_3$ $[M+H]^+$: 374.0386. Found: 374.0384.

2.1.3 Synthesis of **4** and **5**

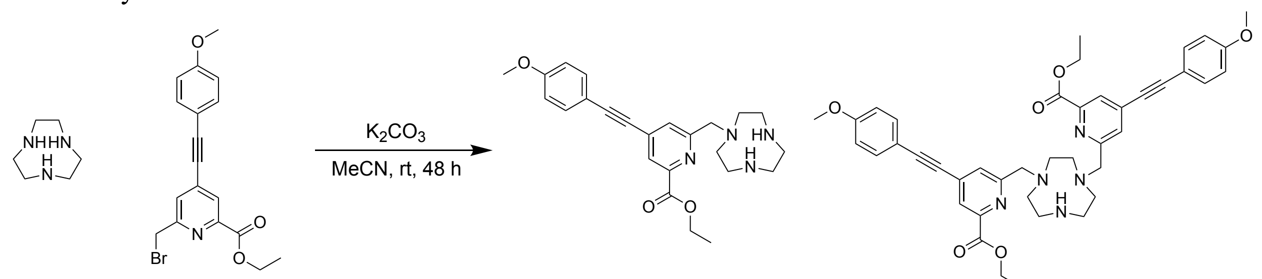

TACN **3** **4** **5**

TACN (205 mg, 1.58 mmol) and K_2CO_3 (271 mg, 1.96 mmol) were suspended in anhydrous acetonitrile (10 mL). To this mixture, **3** (115 mg, 307 µmol, in 10 mL anhydrous MeCN) was dropwise added over 20

minutes. The resulting crude was stirred at room temperature for 48 hours under N_2 atmosphere, filtered to remove excess base and the solvent evaporated to yield a yellowish oil. Purification was carried out via C-18 reversed phase flash chromatography using a gradient of acetonitrile (0-100%) supplemented with 0.1% formic acid to yield monoalkylated intermediate **4** (yield: 56.0 mg, 43%) and bisalkylated **5** (yield: 26.0 mg, 12%) as a yellowish oil and an off white solid, respectively.

Intermediate **4**

^{13}H NMR (500 MHz, MeOD) δ 8.08 – 8.05 (m, 1H), 7.62 (s, 1H), 7.54 (d, *J* = 8.8 Hz, 2H), 6.99 (d, *J* = 8.9 Hz, 2H), 4.52 – 4.47 (m, 2H), 4.17 (s, 2H), 3.85 (s, 3H), 3.43 – 2.82 (m, 12H), 1.48 – 1.44 (m, 4H).

^{13}C NMR (126 MHz, MeOD) δ 169.00, 167.15, 165.97, 162.54, 162.29, 162.07, 134.82, 128.61, 126.40, 115.48, 97.54, 85.65, 63.52, 59.61, 55.94, 53.45, 47.71, 47.36, 14.52.

ESI-HR-MS: Calc m/z for $C_{24}H_{31}N_4O_3$ $[M+H]^+$: 423.2396. Found: 423.2377.

Intermediate **5**

^{13}H NMR (500 MHz, MeOD) δ 8.55 (s, 1H), 7.83 (d, J = 1.4 Hz, 2H), 7.61 (d, J = 1.4 Hz, 2H), 7.42 (d, J = 8.7 Hz, 4H), 6.94 (d, J = 8.8 Hz, 4H), 4.44 (q, J = 7.1 Hz, 4H), 4.02 (s, 4H), 3.79 (s, 6H), 3.23 (t, J = 5.8 Hz, 4H), 2.97 (t, J = 5.8 Hz, 4H), 2.82 (bs, 4H), 1.40 (t, J = 7.1 Hz, 6H).

^{13}C NMR (126 MHz, MeOD): δ 170.25, 165.94, 162.35, 161.75, 135.17, 134.76, 128.65, 126.67, 115.40, 114.72, 96.80, 85.60, 63.25, 60.82, 55.88, 52.18, 48.75, 45.73, 14.56.

ESI-HR-MS: Calc m/z for $C_{42}H_{46}N_5O_6$ $[M+H]^+$: 716.3443. Found: 716.3447.

2.1.4 Synthesis of **6**

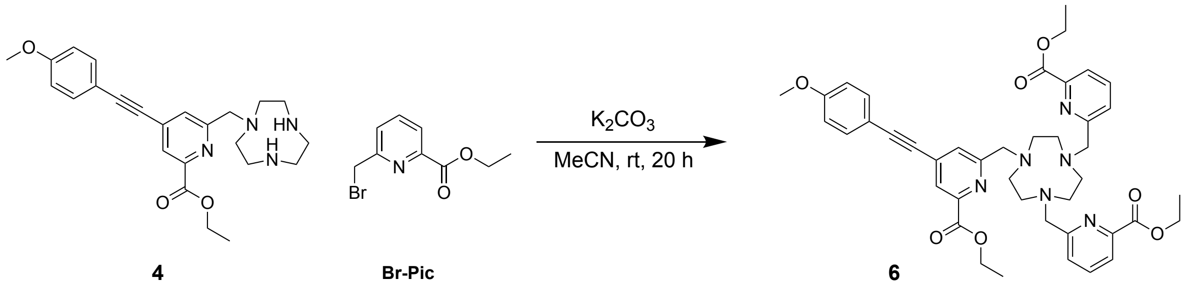

4 (54.5 mg, 129 μmol), K_2CO_3 (59.8 mg, 433 μmol) and Br-Pic (33.5 mg, 137 μmol) were dissolved in anhydrous acetonitrile (3 mL). The resulting mixture was stirred at room temperature for 20 hours under nitrogen atmosphere and then filtered to remove the excess K_2CO_3. The oil recovered after removing the solvent under reduced pressure was purified via C18 RP-HPLC using a gradient of acetonitrile in water with 0.1% formic acid using method A to yield (**6**) as colorless oil (yield: 14.0 mg, 14%).

^{1}H NMR (500 MHz, $CDCl_3$) δ 8.01 (d, *J* = 1.4 Hz, 1H), 7.97 (dd, *J* = 7.5, 1.2 Hz, 2H), 7.82 (t, *J* = 7.7 Hz, 2H), 7.77 (bs, 2H), 7.68 (bs, 1H), 7.49 (d, *J* = 8.8 Hz, 2H), 6.90 (d, *J* = 8.8 Hz, 2H), 4.39 (bs, 6H), 4.19 (bs, 4H), 4.12 (bs, 2H), 3.84 (s, 3H), 3.14 (bs, 12H), 1.37 (bs, 9H).

^{13}C NMR (126 MHz, $CDCl_3$) δ 165.09, 164.80, 160.79, 158.99, 158.24, 147.97, 147.81, 137.87, 133.90, 133.79, 127.96, 127.18, 125.79, 124.11, 114.40, 113.80, 96.07, 85.29, 62.84, 62.80, 62.11, 61.97, 55.53, 54.52, 54.28, 53.46, 14.41, 14.25.

ESI-MS: Calc m/z for $C_{42}H_{49}N_6O_7$ $[M+H]^+$: 749.4. Found: 749.1. Calc m/z for $C_{42}H_{50}N_6O_7$ $[M+2H]^{2+}$: 375.2. Found: 375.2.

2.1.5 Synthesis of **mepa-pic$_2$**

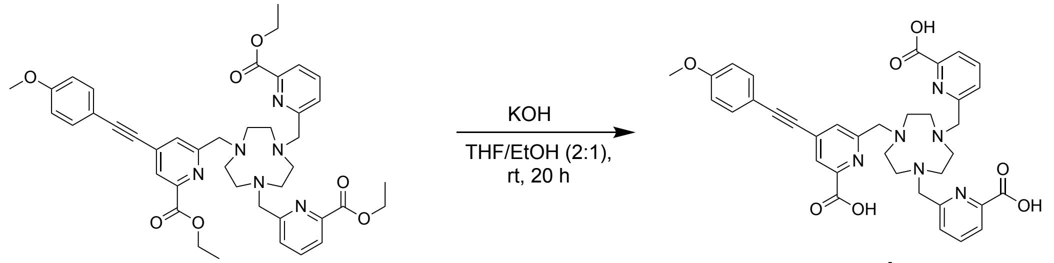

6 **mepa-plc$_2$**

Intermediate **6** (13.3 mg, 17.8 μmol) was first dissolved in a 2:1 mixture of THF:ethanol (5 mL) to which aqueous KOH 0.5 M (360 μL, 180 mmol) was added. The resulting mixture was stirred at room temperature for 2 hours. Next, the solvent was removed under reduced pressure, and the resulting reside purified via C18 RP-HPLC using a gradient of acetonitrile in water with 0.1% formic acid using method B. **mepa-pic$_2$** was recovered as an off-white solid (yield: 8, 70%).

^{1}H NMR (500 MHz, MeOD) δ 8.07 (s, 1H), 8.05 (bs, 2H), 7.91 (t, J = 7.7 Hz, 2H), 7.60 (d, J = 1.5 Hz, 1H), 7.56 (d, J = 7.6 Hz, 2H), 7.50 (d, J = 8.8 Hz, 2H), 6.97 (d, J = 8.8 Hz, 2H), 4.11 (s, 4H), 4.08 (s, 2H), 3.84 (s, 3H), 2.97 (s, 12H).

^{13}C NMR (126 MHz, MeOD) δ 169.91, 169.62, 166.10, 162.35, 157.41, 156.78, 154.24, 152.76, 139.70, 135.30, 134.73, 128.53, 127.96, 126.63, 125.46, 115.42, 114.91, 96.73, 86.08, 62.23, 62.08, 55.92, 53.09, 52.89.

ESI-HR-MS: Calc m/z for $C_{36}H_{37}N_6O_7$ $[M+H]^+$: 665.2718. Found: 665.2712.

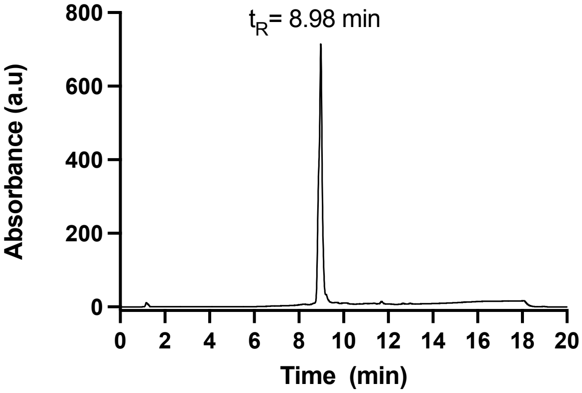

Figure S1. Chromatographic analysis of **mepa-pic$_2$**. Absorbance monitored at 254 nm. Retention time (t_R) = 8.98 min (Method C).

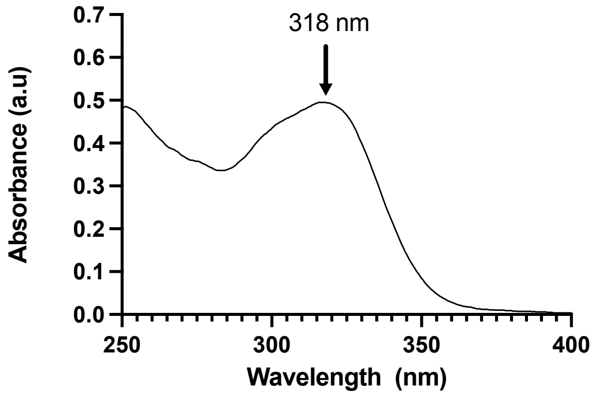

Figure S2. UV-Vis spectrum of mepa-pic$_2$ collected in 10 mM NaOAc pH 5.5.

2.1.6 Synthesis of ^{nat}**Eu-mepa-pic$_2$**

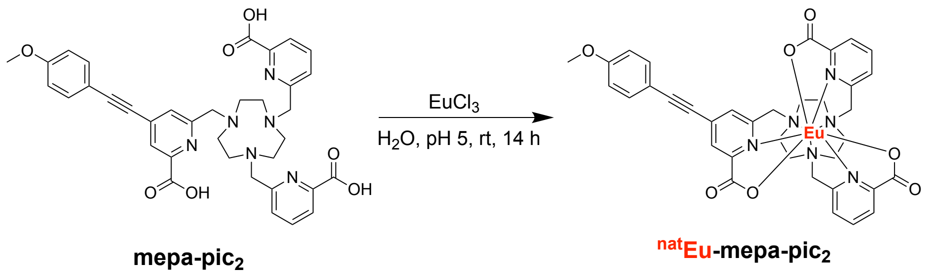

To **mepa-pic$_2$** (2.3 mg, 3.5 µmol) in DI water (2 mL) was added $EuCl_3 6H_2O$ (2.0 mg, 5.4 µmol, in 350 µL H_2O). The pH was adjusted to 5 by addition of 0.5 M KOH (21.0 µL, 11 µmol), and the resulting solution stirred at room temperature for 14 hours. The solvent was removed under reduced pressure and purification of the resulting residue was carried out via Sep-Pak® C18 cartridges using a gradient of acetonitrile (0-100%) in water as eluent. ^{nat}**Eu-mepa$_2$-pic** was recovered as an off-white solid (quant. yield).

ESI-HR-MS: Calc m/z for $C_{36}H_{34}EuN_6O_7$ $[M+H]^+$: 815.1696. Found: 815.1696. Calc m/z for $C_{36}H_{33}EuN_6NaO_7$ $[M+Na]^+$: 837.1521. Found: 815.1508.

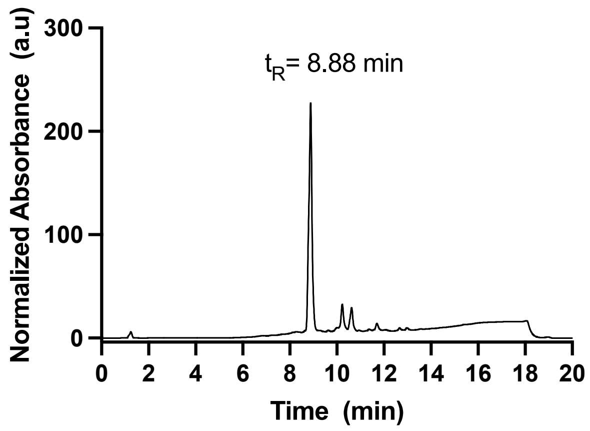

Figure S3. Chromatographic analysis of ^{nat}**Eu-mepa-pic$_2$**. Absorbance monitored at 254 nm. Retention time (t_R) = 8.88 min (Method C).

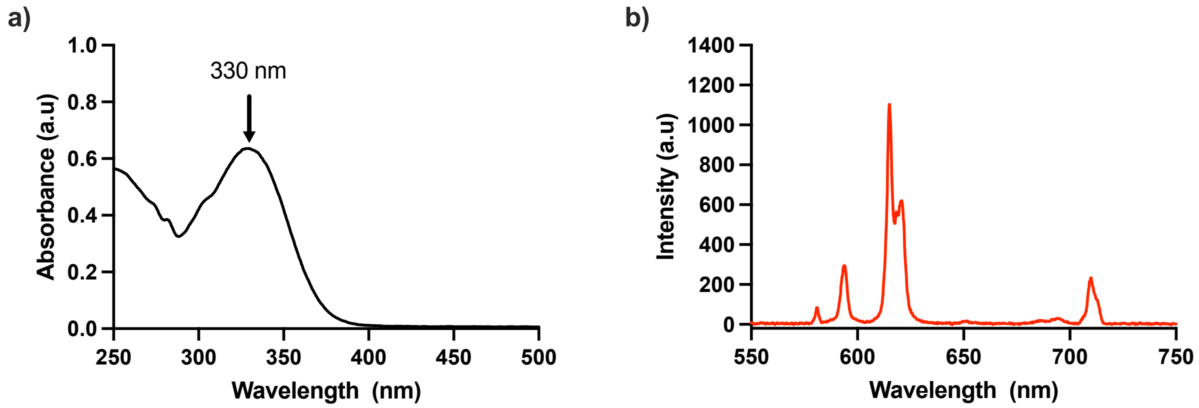

Figure S4. Optical characterization data for ^{nat}Eu-mepa-pic$_2$. A) UV-Vis spectrum collected in 10 mM NaOAc pH 5.5. B) Luminescence emission profile in 10 mM NaOAc pH 5.5. Excitation: 355 nm. Emission collected: 500-800 nm. Excitation and Emission slits: 1 nm. PMT voltage: 700 V.

2.1.7 Synthesis of ^{nat}**Y-mepa-pic$_2$**

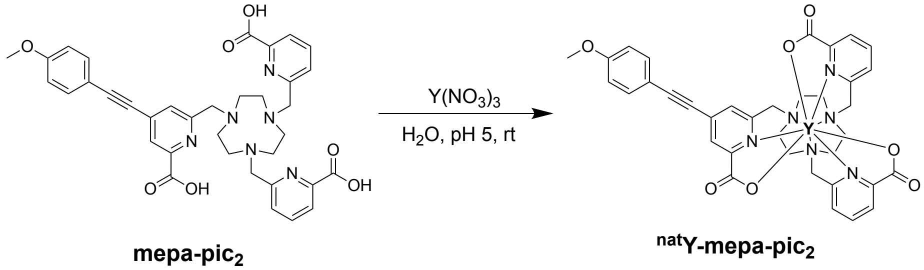

Synthesis of ^{nat}**Y-mepa-pic$_2$** was carried out on a sub-nmol scale following the procedure described in section 2.1.6 for the analog Eu-complex. $Y(NO_3)_3$ was used instead as the source of Y^{3+}, with full conversion assessed via LCMS.

ESI-MS: Calc m/z for $C_{36}H_{34}N_6O_7Y$ $[M+H]^+$: 751.2. Found: 751.2. Calc m/z for $C_{36}H_{34}N_6O_7Y$ $[M+2H]^{2+}$: 376.1. Found: 376.2.

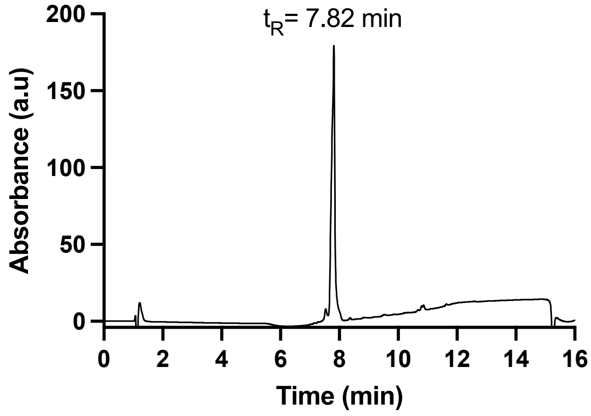

Figure S5. Chromatographic analysis of ^{nat}**Y-mepa-pic$_2$**. Absorbance monitored at 254 nm. Retention time (t_R) = 7.82 min (Method E).

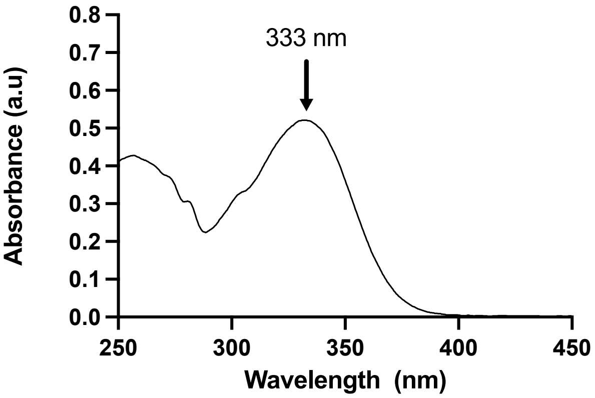

Figure S6. UV-Vis spectrum of **natY-mepa-pic$_2$** collected in 10 mM NaOAc pH 5.5.

2.1.8 Synthesis of **mepa$_2$-pic**.

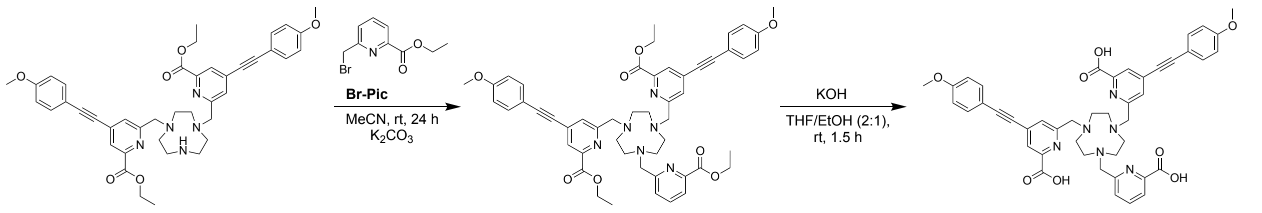

5 **mepa$_2$-pic**

5 (27.7 mg, 38.7 µmol), Br-Pic (15.7 mg, 64.3 µmol), and K_2CO_3 (17.6 mg, 127 µmol) were suspended in anhydrous acetonitrile and stirred at room temperature for 24 hours. After this time, the crude was filtered, and the solvent evaporated to yield a yellowish oil that was used for the next step without further purification. The oil was resuspended in a 1:4 mix of EtOH:THF (5 mL), mixed with KOH 0.5 M (400 µL, 200 µmol) and stirred for 1.5 hours at room temperature, after which the solvent was removed, and the resulting oil purified via C18 RP-HPLC using a gradient of acetonitrile in water with 0.1% formic acid using method A. **mepa$_2$-pic** was recovered as an off-white solid (yield: 16.0 mg, 52% over two steps).

^{1}H NMR (500 MHz, MeOD) δ 7.99 (d, J = 7.8 Hz, 1H), 7.93 (s, 2H), 7.91 (t, J = 7.7 Hz, 1H), 7.67 (s, 2H), 7.60 (d, J = 7.7 Hz, 1H), 7.48 (d, J = 8.8 Hz, 4H), 6.96 (d, J = 8.8 Hz, 4H), 4.43 (s, 2H), 4.36 (s, 4H), 3.83 (s, 6H), 3.60 – 3.31 (bs, 12H).

^{13}C NMR (126 MHz, MeOD) δ 165.90, 161.01, 156.81, 155.58, 148.29, 147.89, 138.33, 134.14, 133.42, 133.37, 127.64, 126.89, 125.53, 124.15, 114.01, 113.30, 95.88, 84.35, 59.81, 59.78, 54.52, 51.71, 51.67., 51.45.

ESI-HR-MS: Calc m/z for $C_{45}H_{43}N_6O_8$ $[M+H]^+$: 795.3137. Found: 795.3129.

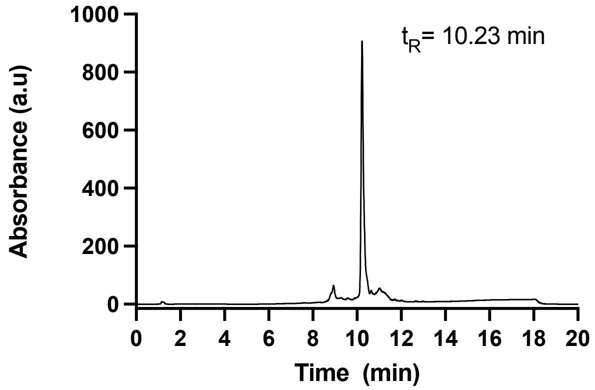

Figure S7. Chromatographic analysis of **mepa$_2$-pic**. Absorbance monitored at 254 nm. Retention time (t_R) = 10.23 min (Method C).

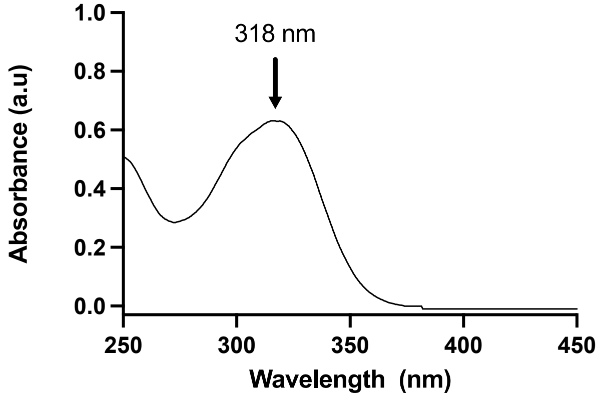

Figure S8. UV-Vis spectrum of **mepa$_2$-pic** collected in 10 mM NaOAc pH 5.5.

2.1.9 Synthesis of **^{nat}Eu-mepa$_2$-pic**

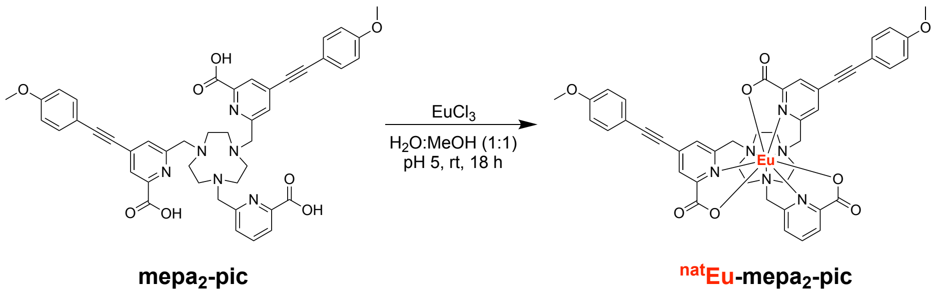

mepa$_2$-pic (3.5 mg, 4.4 µmol) and $EuCl_36H_2O$ (2.4 mg, 6.6 µmol) were dissolved in a 1:1 mix of MeOH:H_2O (3 mL), the pH adjusted to 5 by addition of KOH 0.5 M (5 eq), and the resulting solution stirred at room temperature overnight. Purification of the complex was carried out via Sep-Pak® C18 cartridges using a gradient of acetonitrile (0-100%) in water as eluent. **^{nat}Eu-mepa$_2$-pic** was recovered as an off-white solid (yield: 4.1 mg, 99%)

ESI-HR-MS: Calc m/z for $C_{45}H_{40}EuN_6O_8$ $[M+H]^+$: 945.2115. Found: 945.2116. Calc m/z for $C_{45}H_{39}EuN_6NaO_8$ $[M+Na]^+$: 967.1939. Found 967.1925.

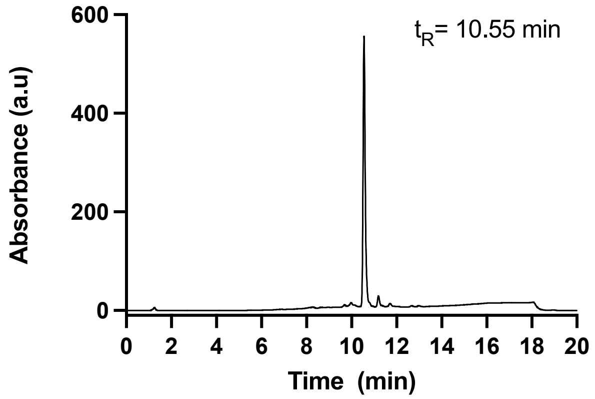

Figure S9. Chromatographic analysis of **^{nat}Eu-mepa$_2$-pic**. Absorbance monitored at 254 nm. Retention time (t_R) = 10.55 min (Method C).

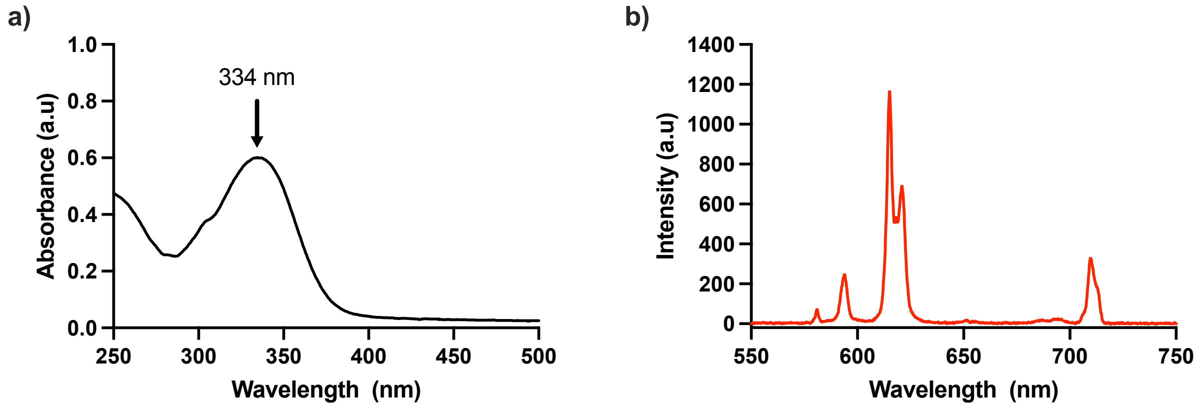

Figure S10. Optical characterization data for **^{nat}Eu-mepa$_2$-pic**. A) UV-Vis spectrum collected in 10 mM NaOAc pH 5.5. B) Luminescence emission profile in 10 mM NaOAc pH 5.5. Excitation: 355 nm. Emission collected: 500-800 nm. Excitation and Emission slits: 1 nm. PMT voltage: 700 V.

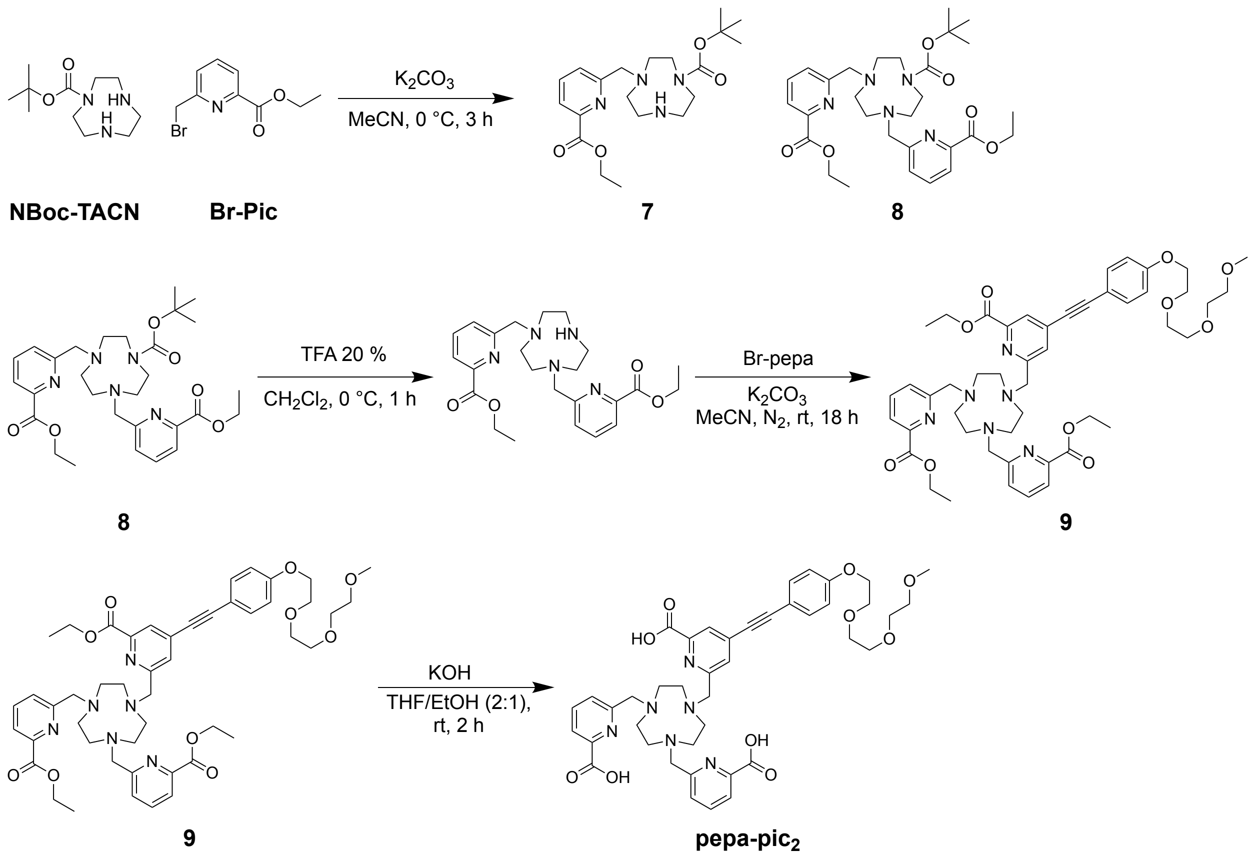

Scheme S2. Synthetic route used to afford **pepa-pic$_2$**.

2.1.10 Synthesis of **7-8**

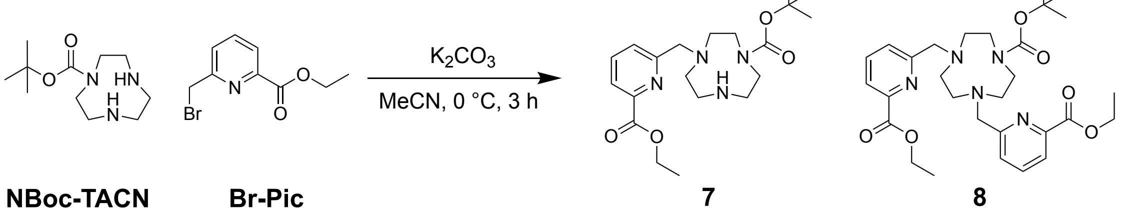

To a mixture of N-Boc-TACN (193.0 mg, 842 µmol) and K_2CO_3 (345 mg, 2.52 mmol) in anhydrous acetonitrile (10 mL) Br-Pic (184 mg, 755 µmol, in 10 mL MeCN) was added over the course of 30 minutes while stirring at 0-4 °C. The resulting crude was then stirred at 0-4 °C for 3 hours. After this time, the crude was filtered to remove the excess of K_2CO_3. Purification was carried out via reversed phase C-18 silica gel flash chromatography using a gradient of acetonitrile (0-100%) in water with 0.1% formic acid. Both, **7** (104 mg, 265 µmol, 32%) and **8** (68.9 mg, 124 µmol, 15%) were recovered as sticky solids.

Intermediate **7**

^{1}H NMR (500 MHz, CD_3CN) δ 8.40 (s, 1H), 7.98 (d, *J* = 7.6 Hz, 1H), 7.90 (td, *J* = 7.8, 3.2 Hz, 1H), 7.50 (t, *J* = 7.5 Hz, 1H), 4.41 (q, *J* = 6.9 Hz, 2H), 4.07 (s, 2H), 3.67 – 3.61 (m, 2H), 3.36 – 3.29 (m, 2H), 3.26 – 3.19 (m, 2H), 3.03 – 2.99 (m, 2H), 2.92 – 2.89 (m, 2H), 2.75 – 2.68 (m, 2H), 1.47 – 1.33 (m, 12H).

^{13}C NMR (126 MHz, CD_3CN) δ 167.48, 165.77, 161.56, 156.11, 148.43, 148.36, 139.34, 139.31, 127.22, 127.16, 124.58, 124.54, 80.70, 80.45, 62.85, 62.82, 60.73, 60.57, 55.77, 55.59, 52.63, 52.32, 52.07, 50.55, 50.34, 48.43, 48.04, 47.62, 46.96, 28.61, 28.57, 14.60. Note the appearance of more C-13 signals than expected, likely caused by conformational restrictions in the macrocycle ring.

ESI-HRMS: Calc m/z for $C_{20}H_{33}N_4O_4$ $[M+H]^+$: 393.2496. Found: 393.2491.

Intermediate **8**

^{1}H NMR (500 MHz, CD_3CN) δ 7.91 (dd, *J* = 7.7, 2.2 Hz, 2H), 7.85 (q, *J* = 7.5 Hz, 2H), 7.71 (d, *J* = 7.7 Hz, 1H), 7.65 (d, *J* = 7.7 Hz, 1H), 4.33 (q, *J* = 7.1 Hz, 4H), 4.00 (s, 4H), 3.43 – 3.38 (m, 4H), 3.15 (t, *J* = 4.7 Hz, 2H), 3.03 (t, *J* = 4.7 Hz, 2H), 2.83 (bs, 2H), 2.77 (bs, 2H), 1.42 (s, 9H), 1.34 (t, *J* = 7.1 Hz, 6H).

^{13}C NMR (126 MHz, CD_3CN) δ 166.12, 165.63, 156.30, 148.62, 148.51, 138.71, 138.65, 127.45, 127.36, 124.42, 80.02, 63.66, 62.44, 56.96, 56.25, 55.70, 55.63, 50.94, 50.14, 28.77, 14.61. Note the appearance of more C-13 signals than expected, likely caused by the conformational restrictions in the macrocycle ring.

ESI-HRMS: Calc m/z for $C_{29}H_{42}N_5O_6$ $[M+H]^+$: 556.3130. Found: 556.3125.

2.1.11 Synthesis of **9**

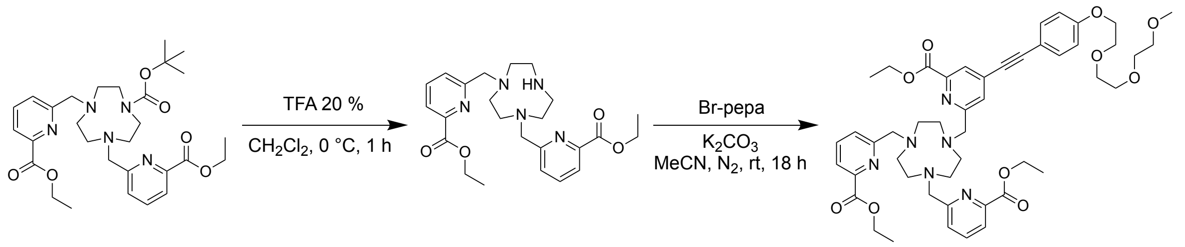

8 **9**

Deprotection of intermediate **8** was carried out in TFA 20% in DCM with progress monitored via LCMS. Upon completion, the solvent is removed under reduced pressure and the deprotected product used without further purification. Next, the deprotected product (95.1 mg, 209 µmol), Br-pepa (101 mg, 200 µmol) and K_2CO_3 (202 mg, 1.46 mmol) were dissolved in anhydrous acetonitrile and stirred at room temperature for 18 hours. The resulting crude was filtered, and the solvent evaporated under reduced pressure to yield a yellowish oil. Purification was carried out via C-18 silica gel flash chromatography using a gradient of acetonitrile (0-100%) in water with formic acid 0.1% as eluent to yield **9** as a colorless oil (yield: 63 mg, 34%).

^{1}H NMR (500 MHz, $CDCl_3$) δ 7.96 (d, *J* = 1.4 Hz, 1H), 7.94 (d, *J* = 7.7 Hz, 2H), 7.81 (t, *J* = 7.8 Hz, 2H), 7.64 (d, *J* = 7.8 Hz, 2H), 7.57 (s, 1H), 7.45 (d, *J* = 8.8 Hz, 2H), 6.90 (d, *J* = 8.9 Hz, 2H), 4.30 (bs, 10H), 4.19 (bs, 2H), 4.16 – 4.08 (m, 2H), 3.87 – 3.82 (m, 2H), 3.73 – 3.70 (m, 2H), 3.68 – 3.65 (m, 2H), 3.65 – 3.62 (m, 2H), 3.55 – 3.50 (m, 2H), 3.35 (s, 3H), 3.34 – 3.14 (m, 12H), 1.31 (t, *J* = 7.1 Hz, 9H).

^{13}C NMR (126 MHz, $CDCl_3$) δ 166.40, 164.74, 164.55, 160.06, 157.84, 156.63, 147.84, 147.75, 138.10, 134.04, 133.71, 127.81, 127.06, 125.88, 124.30, 114.98, 113.71, 96.44, 85.00, 72.00, 70.94, 70.73, 70.64, 69.65, 67.67, 62.08, 61.92, 61.49, 61.09, 59.10, 53.86, 53.74, 53.56, 14.32.

ESI-MS: Calc m/z for $C_{48}H_{61}N_6O_{10}$ $[M+H]^+$: 881.4. Found: 881.4. Calc m/z for $C_{48}H_{62}N_6O_{10}$ $[M+2H]^{2+}$: 441.2. Found: 441.3.

2.1.12 Synthesis of **pepa-pic₂**

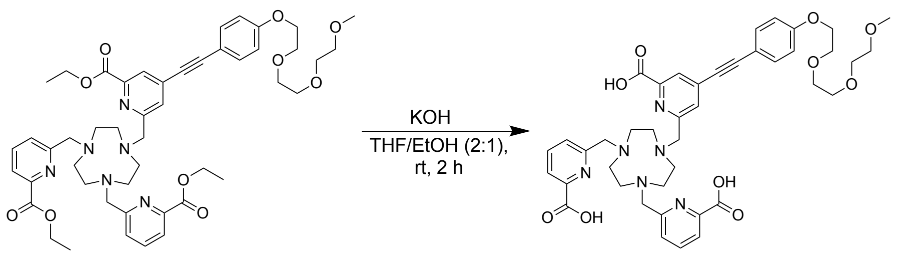

To a solution of **9** (60.0 mg, 68.1μmol) in a 1:2 mixture of THF:EtOH (1.5 mL) 1M KOH (680 mL, 681 μmol) was added. The resulting crude was allowed to stir at room temperature for 2 hours. Purification was carried out via C-18 reversed-phase HPLC using a gradient of MeCN in water with 0.1% formic acid according to method A. **pepa-pic$_2$** was recovered as an off-white solid (yield: 25.6 mg, 47%)

^{1}H NMR (500 MHz, MeOD) δ 8.04 – 7.99 (m, 3H), 7.89 (t, *J* = 7.8 Hz, 2H), 7.61 (s, 1H), 7.55 (d, *J* = 7.7 Hz, 2H), 7.50 (d, *J* = 8.9 Hz, 2H), 6.99 (d, *J* = 8.9 Hz, 2H), 4.21 – 4.15 (m, 8H), 3.88 – 3.83 (m, 2H), 3.74 – 3.68 (m, 2H), 3.67 – 3.62 (m, 4H), 3.56 – 3.50 (m, 2H), 3.35 (s, 3H), 3.12 (s, 12H).

^{13}C NMR (126 MHz, MeOD) δ 169.71, 169.28, 165.86, 161.50, 157.69, 157.14, 153.50, 151.82, 139.56, 135.12, 134.73, 128.36, 127.80, 126.34, 125.16, 116.03, 115.13, 96.55, 86.13, 72.96, 71.78, 71.58, 71.40, 70.71, 68.81, 61.68, 59.08, 53.24, 53.09 (×2).

ESI-HR-MS: Calc m/z for $C_{42}H_{49}N_6O_{10}$ $[M+H]^+$: 797.3505. Found: 797.3495

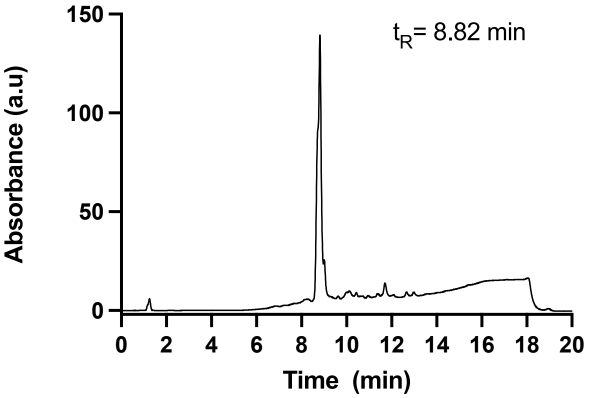

Figure S11. Chromatographic analysis **pepa-pic$_2$**. Absorbance monitored at 254 nm. Retention time (t_R) = 8.82 min (Method C).

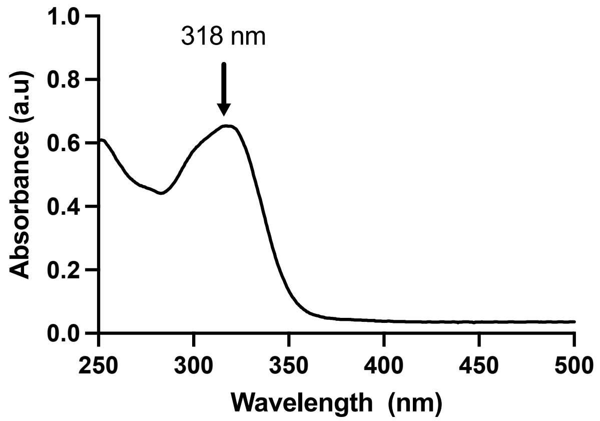

Figure S12. UV-Vis spectrum of **pepa-pic₂** collected in 10 mM NaOAc pH 5.5.

2.1.13 Synthesis of ^{nat}**Eu-pepa-pic₂**

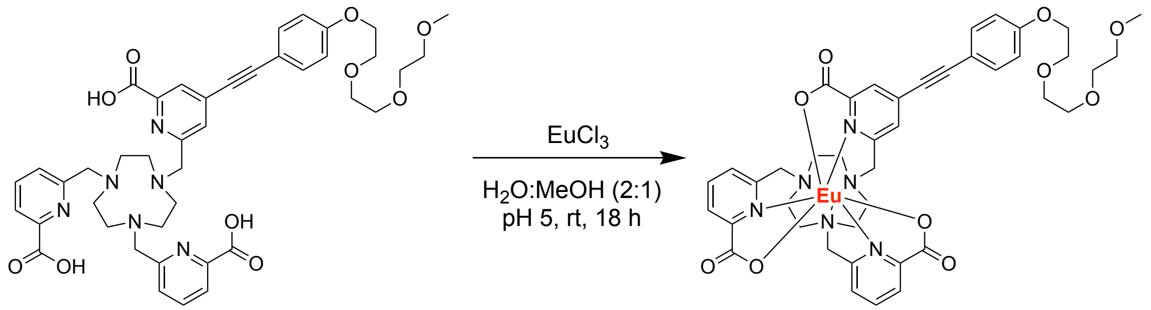

pepa-pic₂ ^{nat}**Eu-pepa-pic₂**

To **pepa-pic₂** (6.7 mg, 8.4 µmol) dissolved in 2 ml of water was added $EuCl_36H_2O$ (7.1 mg, 19 µmol). KOH 1M (40 µL, 40 µmol) was added to adjust the final pH to 5, followed by addition of MeOH (1 mL). and the resulting mixture was stirred at room temperature overnight. The titled compound was purified via Sep-Pak® C18 cartridges using a gradient of acetonitrile (0-100%) in water as eluent (yield: 7.1 mg, 89%)

ESI-HR-MS: Calc m/z for $C_{42}H_{46}EuN_6O_{10}$ $[M+H]^+$: 947.2482. Found: 947.2476. Calc m/z for $C_{42}H_{45}EuN_6NaO_{10}$ $[M+H]^+$: 969.2307. Found: 969.2299.

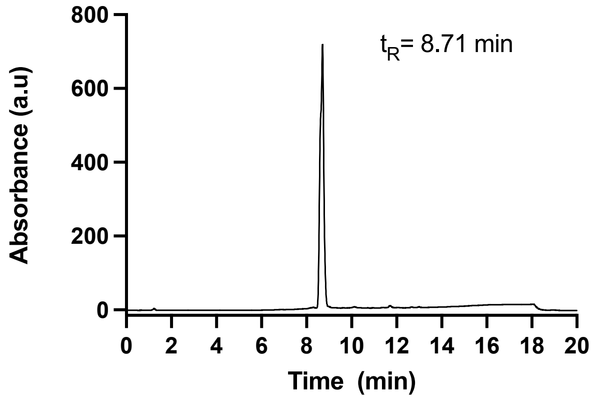

Figure S13. Chromatographic analysis **^{nat}Eu-pepa-pic$_2$**. Absorbance monitored at 254 nm. Retention time (t_R) = 8.71 min (Method C).

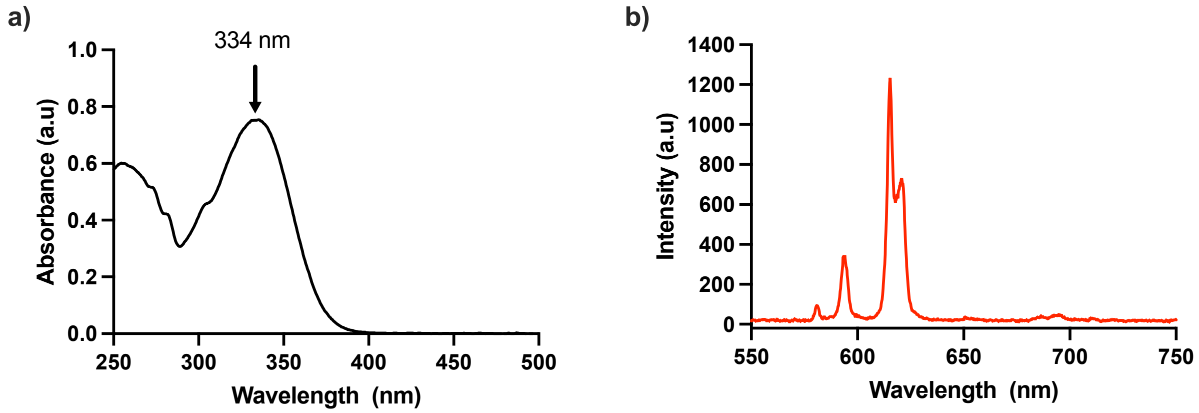

Figure S14. Optical characterization data for **^{nat}Eu-pepa-pic$_2$**. A) UV-Vis spectrum collected in 10 mM NaOAc pH 5.5. B) Luminescence emission profile in 10 mM NaOAc pH 5.5. Excitation: 355 nm. Emission collected: 500-800 nm. Excitation and Emission slits: 1 nm. PMT voltage: 700 V.

2.1.14 Synthesis of **^{nat}Y-pepa-pic$_2$**

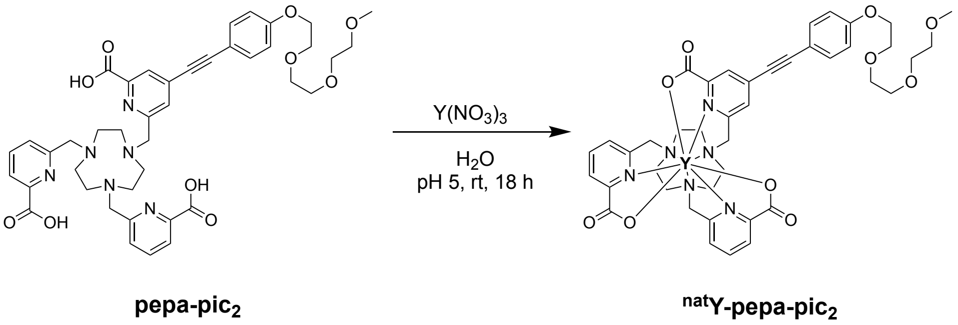

Synthesis of **^{nat}Y-pepa-pic$_2$** was carried out on a sub-nmol scale following a procedure described in section 2.1.7.

ESI-MS: Calc m/z for $C_{42}H_{46}N_6O_{10}Y$ $[M+H]^+$: 883.2. Found: 883.2. Calc m/z for $C_{42}H_{47}N_6O_{10}Y$ $[M+2H]^{2+}$: 442.1. Found: 442.2.

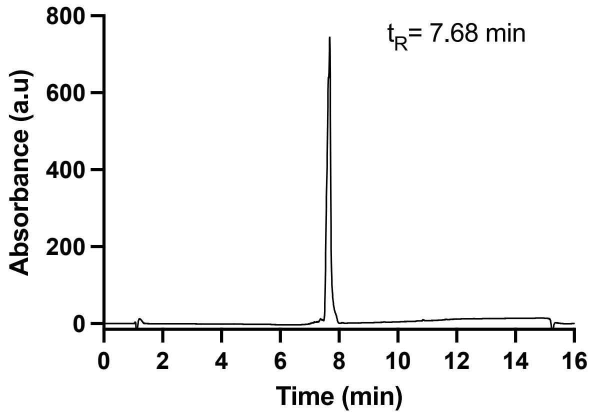

Figure S15. Chromatographic analysis **^{nat}Y-pepa-pic$_2$**. Absorbance monitored at 254 nm. Retention time (t_R) = 7.68 min (Method E).

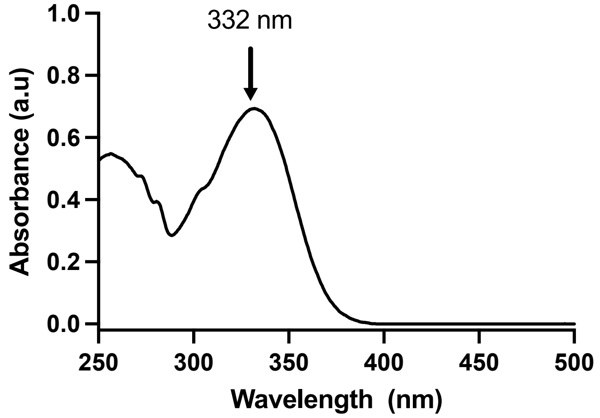

Figure S16. UV-Vis spectrum of **^{nat}Y-pepa-pic$_2$** collected in 10 mM NaOAc pH 5.5.

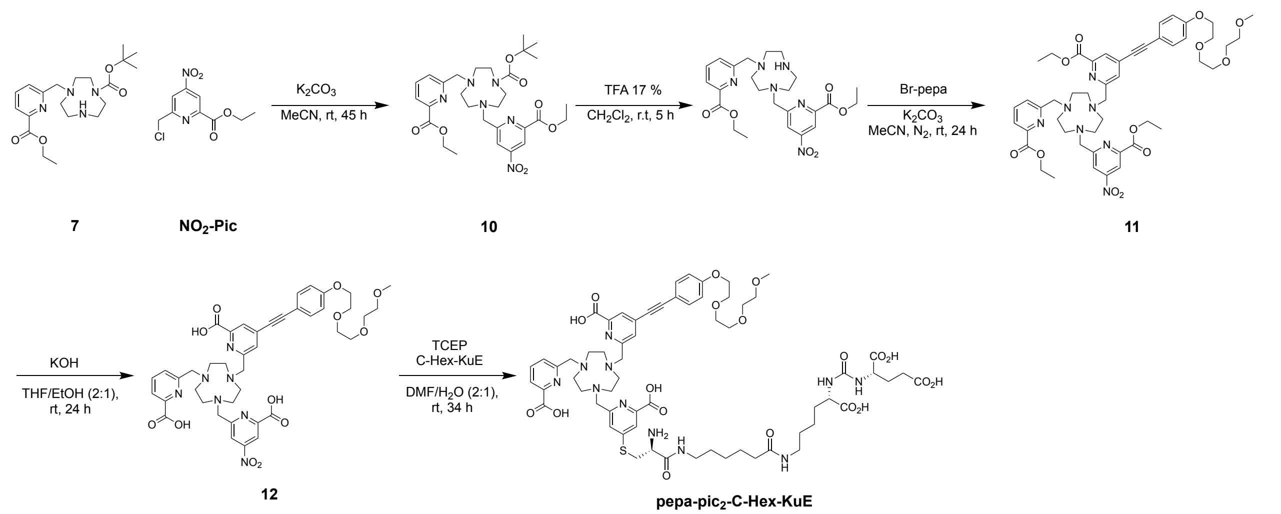

Scheme S3. Schematic route for the synthesis of the targeted conjugate pepa-pic$_2$-C-Hex-KuE.

2.1.15 Synthesis of **C-Hex-KuE** peptide

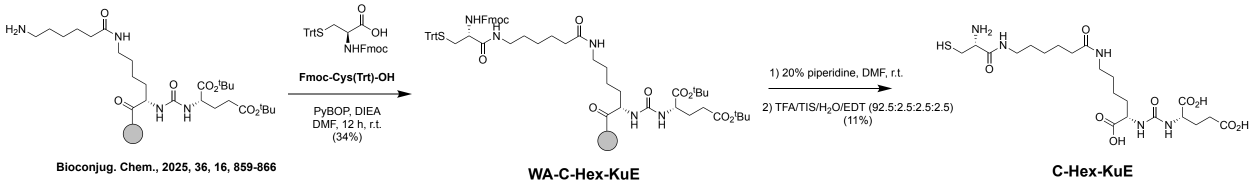

The synthesis of C-Hex-KuE was performed on a 0.16 mmol scale using Wang (WA) resin (200 mg, 0.8 mmol/g). The resin-bound compound WA-C-Hex-KuE (synthesized by us previously, *Bioconjug. Chem.,* **2025,** 36, 16, 859-866) was swollen in DCM (2 mL) and DMF (2 mL) for 1 minute, repeated three times. Fmoc-Cys(Trt)-OH (481 mg, 0.64 mmol), PyBOP (167 mg, 0.32 mmol) and DIEA (111 µL, 0.64 mmol) were dissolved in DMF and loaded onto the resin. The syringe containing the resin and solution was shaken for 12 hours at room temperature. Removal of the Fmoc group was conducted by treating the resin with 20% piperidine in DMF (3 mL) for 15 minutes (twice). The products were washed, dried, and stored at -20 °C until used. To release the final C-Hex-KuE peptide from the solid support, the resin-bound peptide was treated with a mixture of TFA/TIS/H_2O/EDT (92.5%/2.5%/2.5%/2.5%) for 1 hour. Purification was carried out via C-18 reversed-phase HPLC according to method A.

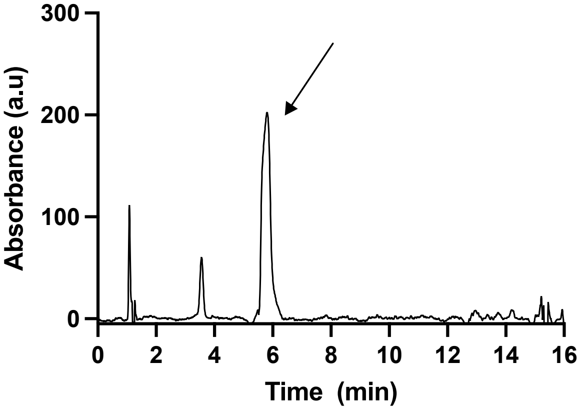

Figure S17. Baseline corrected chromatogram of C18 HPLC purified **C-Hex-KuE** peptide. Absorbance monitored at 220 nm. Retention time (t_R) = 5.84 min (Method E).

ESI-MS: Calc m/z for $C_{21}H_{38}N_5O_9S$ $[M+H]^+$: 536.2. Found: 536.1.

2.1.16 Synthesis of **10**

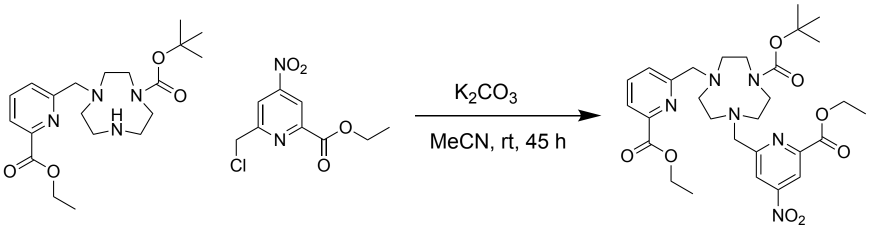

7 **NO2-Pic** **10**

To **7** (40.2 mg, 102 µmol) dissolved in anhydrous acetonitrile (2 mL), K_2CO_3 (49.5 mg, 358 µmol) was added while stirring under N_2 atmosphere. To the resulting mixture, NO_2-Pic (30.8 mg, 126 µmol in 2 mL anhydrous CH_3CN) was added. The crude was then allowed to stir at room temperature. Reaction was monitored via LCMS. After 18 hours the reaction had not gone to completion, thus a second portion of NO_2-Pic (23.9 mg, 97.7 µmol) was added and stirred at room temperature for 3 hours without much progress. Last, a third portion of NO_2-Pic (53.4 mg, 219 µmol) was added, and the crude stirred while heating at 40 °C for 24 hours. After this time, volatiles were removed under reduced pressure to yield a yellowish oil. Purification was carried out via C-18 reversed-phase HPLC using a gradient of MeCN in water with 0.1% formic acid according to method A. Yield: 32.7 mg (53%)

^{1}H NMR (500 MHz, MeOD) δ 8.50 (s, 1H), 8.40 (d, *J* = 13.5 Hz, 1H), 8.01 – 7.94 (m, 2H), 7.69 – 7.62 (m, 1H), 4.65 (s, 1H), 4.56 (s, 1H), 4.46 (d, *J* = 20.2 Hz, 2H), 4.39 – 4.31 (m, 2H), 4.24 (dq, *J* = 14.3, 7.2 Hz, 2H), 3.90 – 3.32 (m, 8H), 3.30 – 3.06 (m, 4H), 1.48 (s, 5H), 1.38 – 1.25 (m, 6H).

^{13}C NMR (126 MHz, MeOD) δ 164.01, 163.37, 155.72, 155.40, 149.54, 147.05, 138.56, 126.58, 124.27, 124.17, 119.20, 119.10, 116.18, 116.11, 80.69, 80.55, 62.45, 62.08, 61.76, 61.59, 61.54, 60.81, 60.50, 57.05, 56.20, 55.98, 55.74, 53.61, 53.33, 49.87, 49.21, 48.74, 48.51, 27.29, 13.06, 13.01.

ESI-HR-MS: Calc for $C_{29}H_{41}N_6O_8$ $[M+H]^+$: 601.2980. Found: 601.2978.

2.1.17 Synthesis of **11**

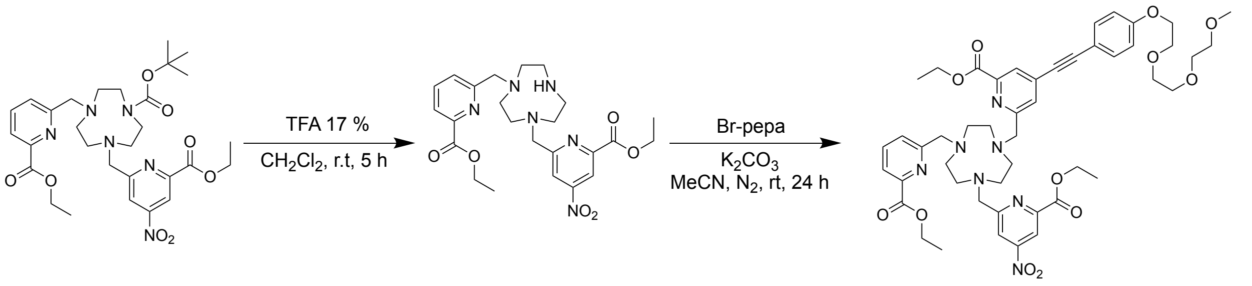

10 **11**

To a solution **10** (335 mg, 558 µmol) in dichloromethane (10 mL) at 0 °C was added TFA (2 mL) and stirred at room temperature for 5 hours. Next, volatiles were removed under reduced pressure. The resulting yellowish oil was mixed with K_2CO_3 (269 mg, 1.95 mmol) and Br-pepa (323 mg, 638 µmol) in anhydrous acetonitrile (2 mL) and stirred at room temperature under nitrogen atmosphere for 24 hours. The suspension was filtered and purified by C18 reversed flash chromatography using a gradient of MeCN in water both with 0.1% formic acid. Yield: 118 mg (23%)

^{1}H NMR (500 MHz, MeOD) δ 8.56 (s, 1H), 8.52 (s, 1H), 8.04 – 7.90 (m, 3H), 7.79 (bs, 2H), 7.47 (d, *J* = 8.8 Hz, 2H), 6.99 (d, *J* = 8.8 Hz, 2H), 4.50 – 4.22 (m, 8H), 4.21 – 4.15 (m, 2H), 4.14 – 3.88 (m, 4H), 3.87 – 3.83 (m, 2H), 3.73 – 3.69 (m, 2H), 3.67 – 3.62 (m, 4H), 3.55 – 3.50 (m, 2H), 3.35 (s, 3H), 2.97 (bs, 12H), 1.38 (s, 9H).

^{13}C NMR (126 MHz, MeOD) δ 170.26, 165.83, 165.30, 164.78, 161.61, 156.83, 148.83, 148.60, 139.35, 134.75, 129.29, 128.33, 120.29, 120.22, 117.27, 116.07, 114.88, 85.86, 72.97, 71.78, 71.59, 71.40, 70.69, 68.83, 63.63, 63.13, 62.93, 59.09, 58.32, 18.37, 14.54, 14.51, 14.48.

ESI-HR-MS: Calc m/z for $C_{48}H_{60}N_7O_{12}$ $[M+H]^+$: 926.4294. Found: 926.4281. Calc m/z for $C_{48}H_{59}N_7NaO_{12}$ $[M+Na]^+$: 948.4119. Found: 948.4099.

2.1.18 Synthesis of **12**

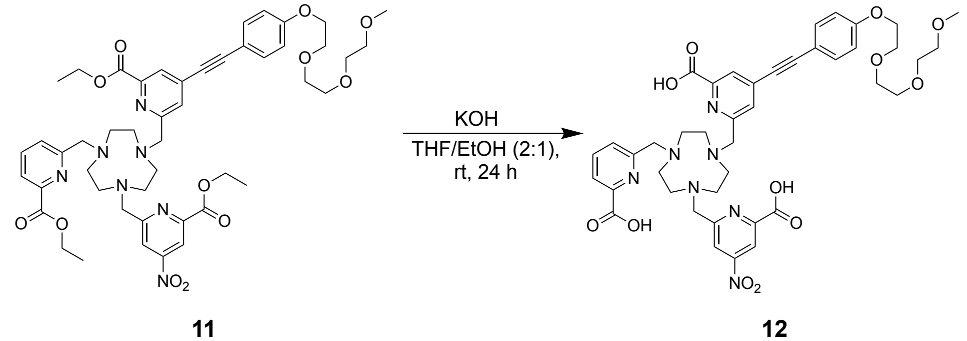

11 **12**

To a solution of **11** (118 mg, 128 µmol) in THF (4 mL) was added KOH 1 M (1.28 mL, 1.28 mmol). The solution was stirred at room temperature for 24 hours. Volatiles were removed and the residue purified via C-18 reversed-phase HPLC using a gradient of MeCN in water with 0.1% formic acid according to method A. Yield: 48.8 mg (45%)

^{1}H NMR (500 MHz, CD_3CN) δ 8.44 (s, 1H), 8.19 (s, 1H), 8.02 – 7.91(m, 3H), 7.63 – 7.61(m, 2H), 7.51 (d, *J* = 9.1 Hz, 2H), 6.97 (d, *J* = 9.1 Hz, 2H), 4.46 (s, 2H), 4.32 (d, *J* = 14.2 Hz, 4H), 4.18 – 4.12 (m, 2H), 3.82 – 3.76 (m, 2H), 3.65 – 3.61 (m, 2H), 3.59 – 3.53 (m, 4H), 3.52 – 2.92 (m, 17H).

^{13}C NMR (126 MHz, CD_3CN) δ 165.78, 165.62, 165.21, 161.78, 161.17, 161.01, 160.73, 156.36, 154.36, 148.02, 140.14, 134.74, 129.22, 128.69, 126.27, 125.02, 120.07, 117.26, 115.94, 114.26, 97.13, 85.61, 72.57, 71.37, 71.11, 71.00, 70.07, 68.70, 60.72, 60.38, 58.86, 52.95, 51.89, 51.64, 51.18.

ESI-HR-MS: Calc m/z for $C_{42}H_{47}KN_7O_{12}$ $[M+K]^+$: 880.2920. Found: 880.2822.

2.1.19 Synthesis of **pepa-pic₂-C-Hex-KuE**

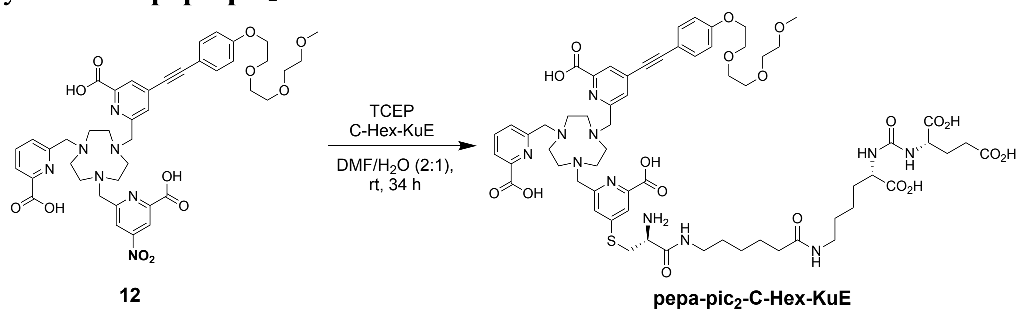

12 (8.5 mg, 10 µmol), C-Hex-KuE (10.4 mg, 19.4 µmol) and TCEP HCl (10.9 mg, 38.0 µmol) were dissolved in a 1:3 freshly degassed mixture of NH_4HCO_3 100 mM pH 7.8 buffer: DMF (1.5 mL). The resulting mixture was stirred at room temperature under nitrogen atmosphere for 35 hours. The solvent was removed under reduced pressure and the residue purified via HPLC according to method B to yield an off-white solid. Yield: 5.2 mg (39%).

ESI-MS: Calc m/z for $C_{63}H_{84}N_{11}O_{19}S$ $[M+H]^+$: 1330.6. Found 1330.1. Calc m/z for $C_{63}H_{85}N_{11}O_{19}S$ $[M+2H]^{2+}$: 665.8. Found 665.7. Calc for $C_{63}H_{86}N_{11}O_{19}S$ $[M+3H]^{3+}$: 444.2. Found 444.2.

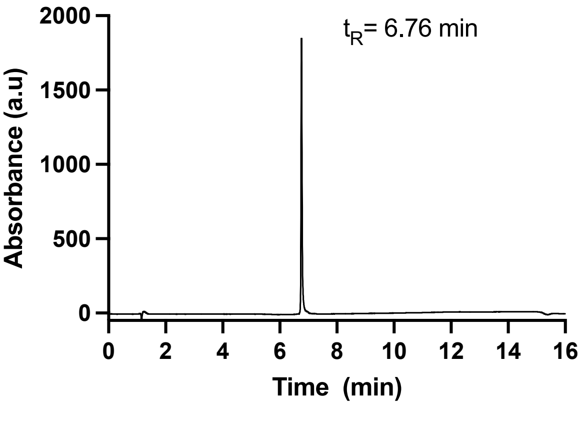

Figure S18. Chromatographic analysis **pepa-pic₂-C-Hex-KuE**. Absorbance monitored at 254 nm. Retention time (t_R) = 6.76 min (Method E).

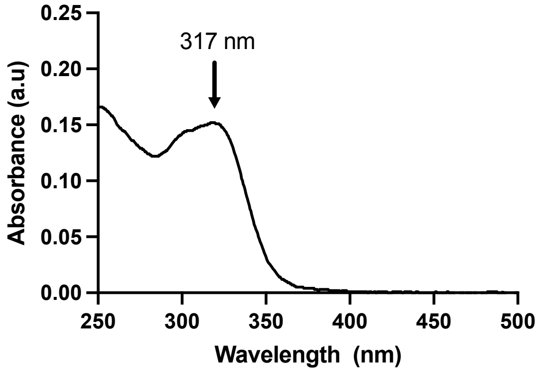

Figure S19. UV-Vis spectrum of **pepa-pic$_2$-C-Hex-KuE** collected in 10 mM NaOAc pH 5.5.

2.1.20 Synthesis of ^{nat}Eu-pepa-pic$_2$-C-Hex-KuE

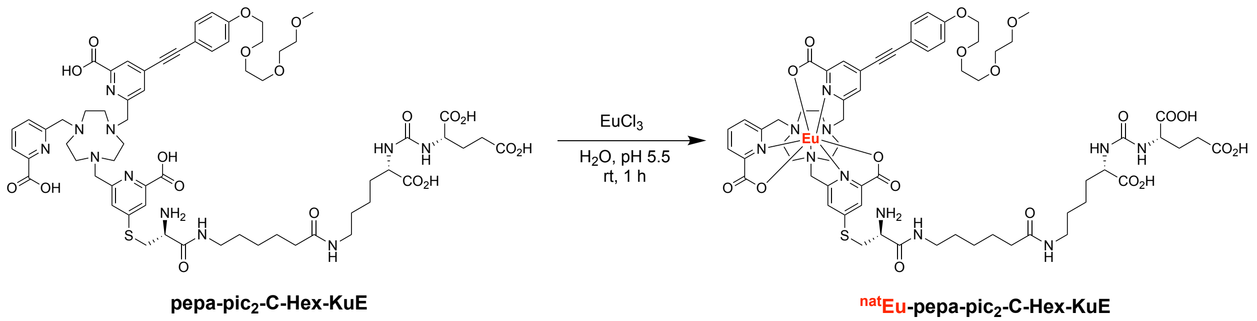

To an aqueous solution of **pepa-pic-C-Hex-KuE** (2.59 mM, 130 µL, 0.337 µmol) was added $EuCl_3$ (10.6 mM, 64 µL, 0.680 µmol), followed by 1M KOH (1.62 µL, 1.62 µmol) to adjust the pH to 5-6. Reaction progress was monitored via LCMS. After 1 hour, the crude was loaded into a Sep-Pak® C18 cartridge, washed extensively with H_2O, and then eluted with a mixture 1:1 of MeCN:H_2O to yield the title compound as a white solid (yield: 0.337 µmol, 99%)

ESI-HR-MS: Calc m/z for $C_{63}H_{79}EuN_{11}O_{19}S$ [M-H]$^-$: 1478.4487. Found: 1478.4486.

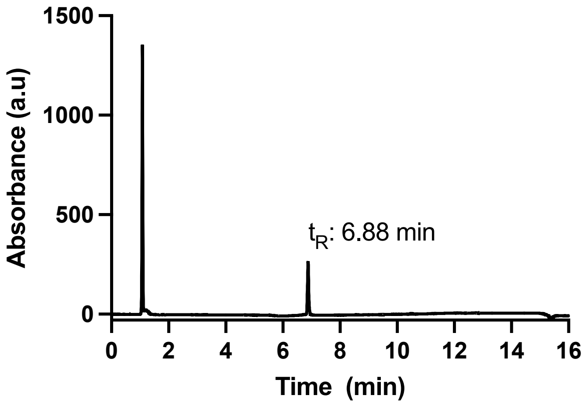

Figure S20. Chromatographic analysis **^{nat}Eu-pepa-pic$_2$-C-Hex-KuE**. Absorbance monitored at 254 nm. Retention time (t_R) = 6.88 min (Method E). Note the solvent front around 1 min.

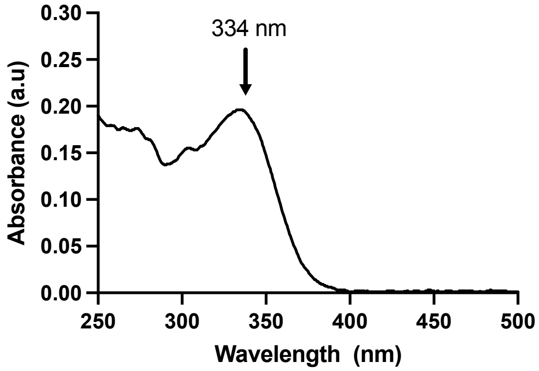

Figure S21. UV-Vis spectrum of **natEu-pepa-pic$_2$-C-Hex-KuE** collected in 10 mM NaOAc pH 5.5.

2.1.21 Synthesis of natLu-pepa-pic$_2$-C-Hex-KuE

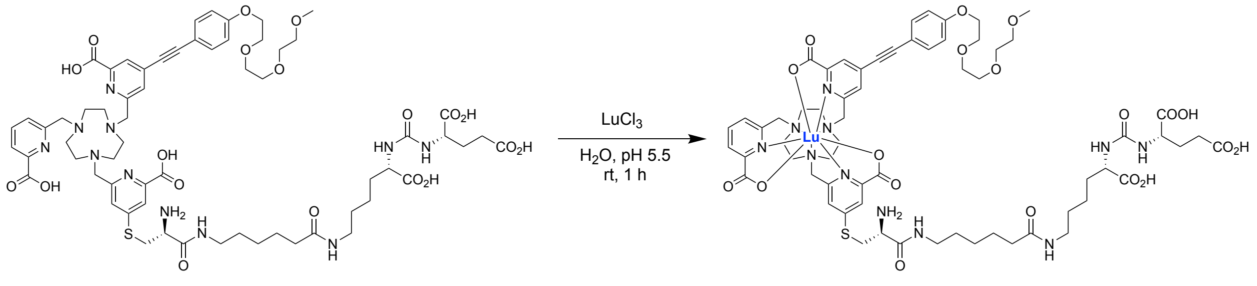

pepa-pic$_2$-C-Hex-KuE

natLu-pepa-pic$_2$-C-Hex-KuE

Synthesis of **natLu-pepa-pic$_2$-C-Hex-KuE** was carried out on a sub-nmol scale following the procedure previously described in section 2.1.20, using $LuCl_3$ as source of Lu^{3+}, with full conversion assessed via LCMS. No purification (desalting) was conducted.

ESI-MS: Calc m/z for $C_{63}H_{81}LuN_{11}O_{19}S$ $[M+H]^+$: 1502.5. Found: 1502.9. Calc m/z for $C_{63}H_{82}LuN_{11}O_{19}S$ $[M+2H]^{2+}$: 751.7. Found: 751.8.

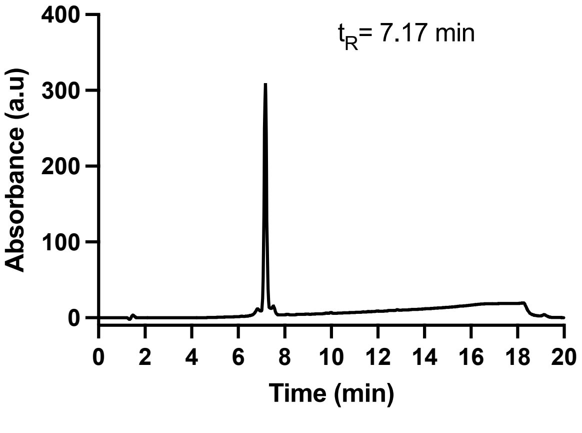

Figure S22. Chromatographic analysis **natLu-pepa-pic$_2$-C-Hex-KuE**. Absorbance monitored at 254 nm. Retention time (t_R) = 7.17 min (Method D).

2.1.22 Synthesis of ^{nat}Tb-pepa-pic$_2$-C-Hex-KuE

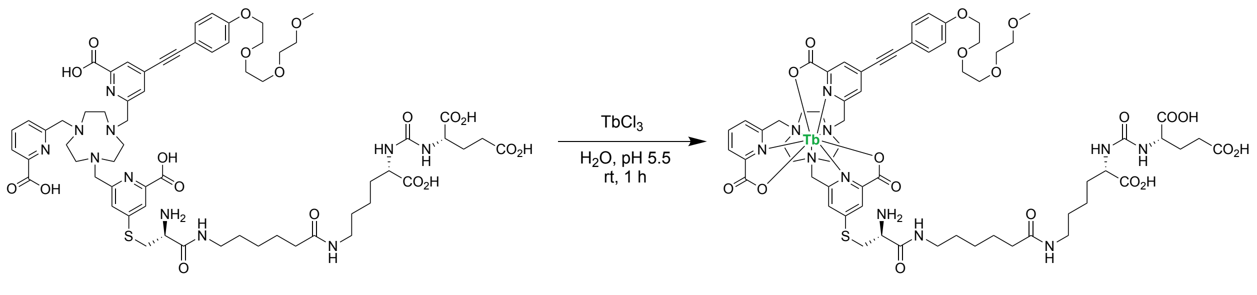

Synthesis of **^{nat}Tb-pepa-pic$_2$-C-Hex-KuE** was carried out on a sub-nmol scale as described in section 2.1.20, using $TbCl_3$ as a source of Tb^{3+}, with full conversion monitored via LCMS. No purification (desalting) was conducted.

ESI-MS: Calc m/z for $C_{63}H_{81}N_{11}O_{19}STb$ $[M+H]^+$: 1486.5. Found: 1486.9. Calc m/z for $C_{63}H_{82}N_{11}O_{19}STb$ $[M+2H]^{2+}$: 743.7. Found: 743.6.

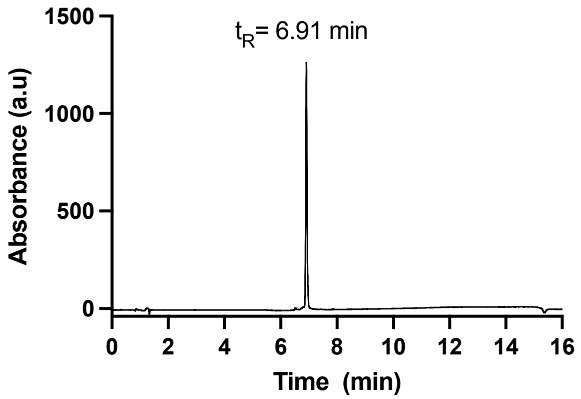

Figure S23. Chromatographic analysis **^{nat}Tb-pepa-pic$_2$-C-Hex-KuE**. Absorbance monitored at 254 nm. Retention time (t_R) = 6.91 min (Method E).

2.2 Complex synthesis and characterization (photophysical characterization)

2.2.1 Molar absorptivity determination of ligands **mepa-pic$_2$, mepa$_2$-pic, pepa-pic$_2$ and pepa$_2$-pic**.

To determine the concentration and molar absorptivity of each ligand used for subsequent radiolabeling experiments, spectrophotometric titrations were carried out with Y^{3+}. The formation of each Yttrium complex was monitored by UV-Vis spectroscopy using a 1 cm path length quartz cuvette and a NanoDrop One^C spectrophotometer. The pH of the solution during the titration was kept constant (pH 5.5) by using 10 mM sodium acetate buffer pH 5.5.

Stock solutions of the ligands **mepa-pic$_2$, pepa-pic$_2$ and pepa$_2$-pic** were prepared in 10 mM sodium acetate buffer pH 5.5. A stock solution of **mepa$_2$-pic** was prepared in a similar manner, but DMSO (2.5% final concentration) was used due to its limited solubility. For the titrations, the solutions of ligands used were diluted so that the initial absorbance at 330 nm was between 0.4 – 0.6.

For **pepa-pic₂**, a 25.1 µM (1000 µL, 25.2 nmol) stock solution was titrated with 5 µL (2 nmol) aliquots of Y^{3+} (as determined by ICP-OES). Similarly, a 13.9 µM (1005 µL, 14.0 nmol) stock solution of **pepa₂-pic** was titrated with 5 µL (1.3 nmol) aliquots of Y^{3+}; a 20.5 µM (1010 µL, 20.7 nmol) stock solution of **mepa-pic₂** was titrated with 5 µL (1.4 nmol) aliquots of Y^{3+}; and a 9.21 µM (1050 µL, 9.68 nmol) stock solution of **mepa₂-pic** was titrated with 5 µL (1.6 – 3.4 nmol) aliquots of Y^{3+}.The end point of the titrations was determined by the inflection point of the absorbance intensity at 330-332 nm, diagnostic of complex formation, and the exact concentration in the original stock solution calculated.

With the exact concentration of each ligand determined, standard curves ranging from 1.0 – 0.1 absorbance at the maximum wavelength of absorbance were prepared and plotted as a function of concentration. The slopes were determined using simple linear regression in Graph Pad Prism. Each molar absorptivity (ε) was calculated from the slope of the appropriate standard curve according to the Beer-Lambert law.

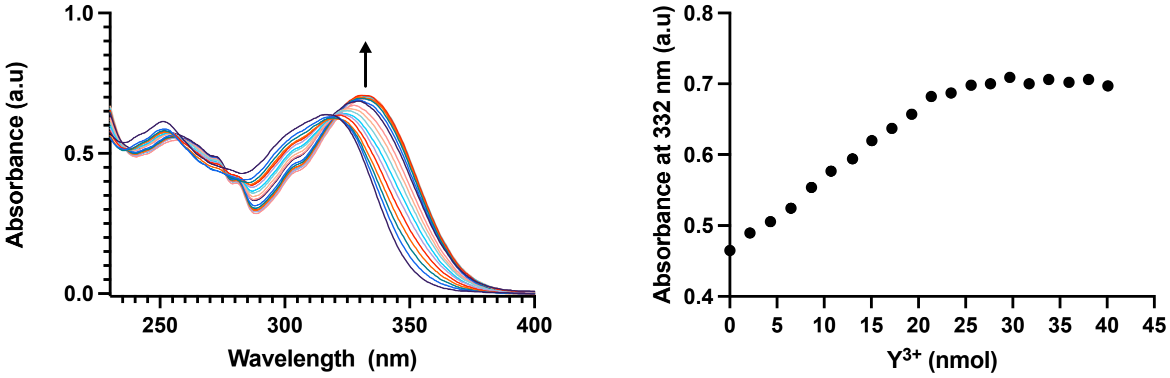

Figure S24. Spectrophotometric titration of **pepa-pic₂** with Y^{3+}. UV-vis absorbance spectra of pepa-pic₂ upon Y^{3+} addition (left) and UV-vis titration to endpoint to determine ligand concentration (right).

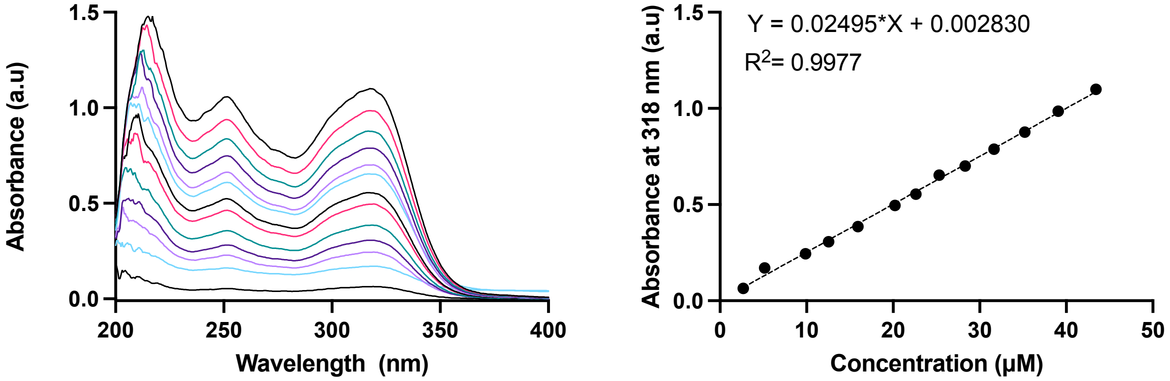

Figure S25. UV-Vis analysis of **pepa-pic₂** to determine molar extinction coefficient. UV-vis absorbance spectra of pepa-pic₂ at decreasing concentration (left) and analysis of spectra at 318 nm used to determine the molar extinction coefficient (right).

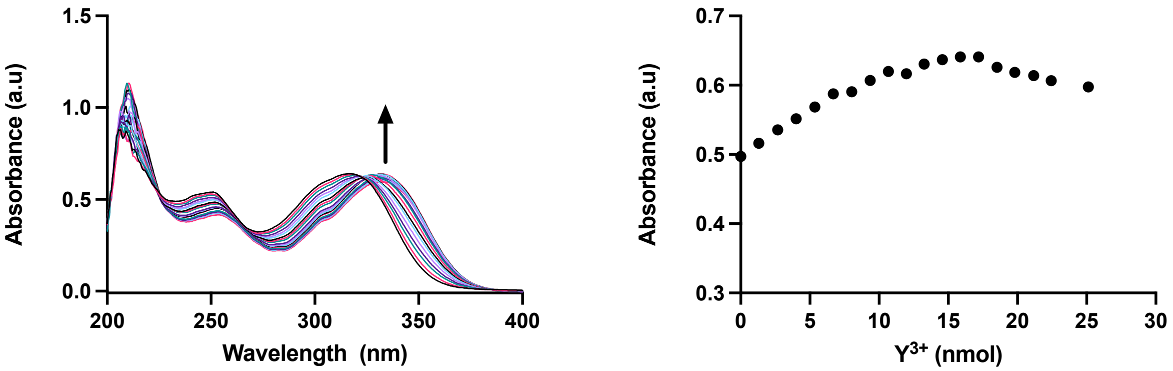

Figure S26. Spectrophotometric titration of **pepa$_2$-pic** with Y^{3+}. UV-vis absorbance spectra of pepa$_2$-pic upon Y^{3+} addition (left) and UV-vis titration to endpoint to determine ligand concentration (right).

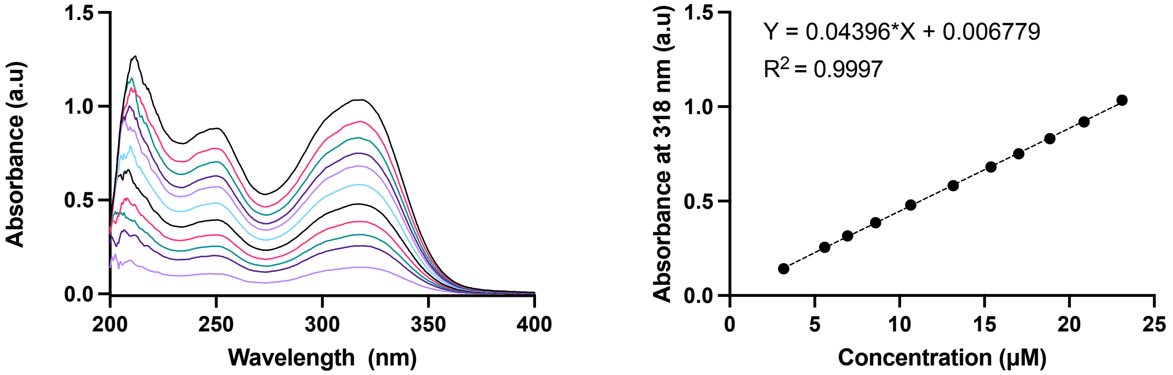

Figure S27. UV-Vis analysis of **pepa$_2$-pic** to determine molar extinction coefficient. UV-vis absorbance spectra of pepa$_2$-pic at decreasing concentration (left) and analysis of spectra at 318 nm used to determine the molar extinction coefficient (right).

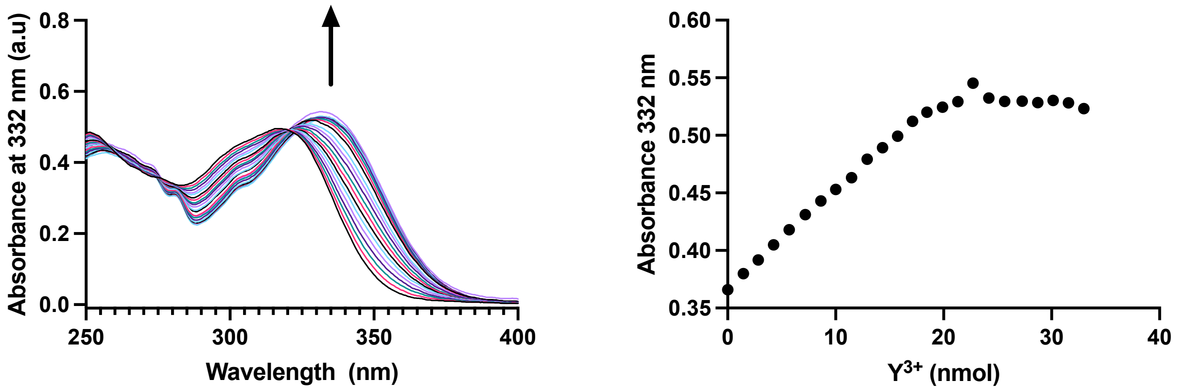

Figure S28. Spectrophotometric titration of **mepa-pic$_2$** with Y^{3+}. UV-vis absorbance spectra of mepa-pic$_2$ upon Y^{3+} addition (left) and UV-vis titration to endpoint to determine ligand concentration (right).

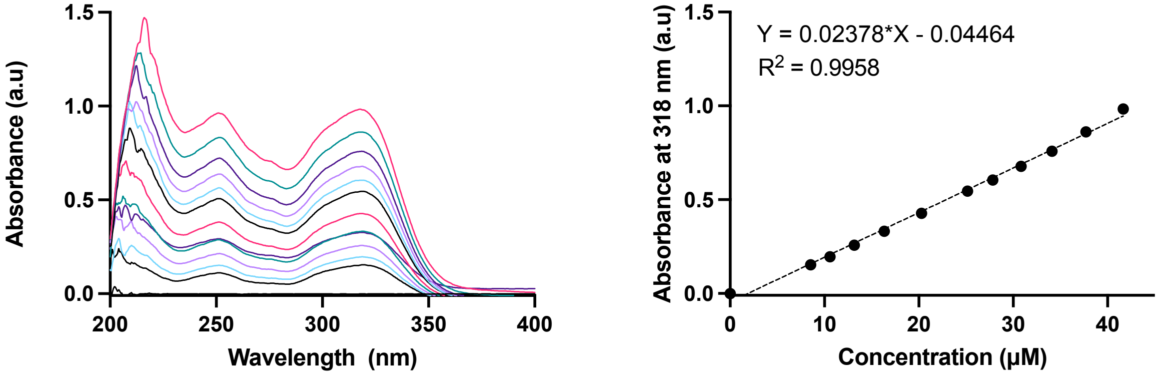

Figure S29. UV-Vis analysis of **mepa-pic$_2$** to determine molar extinction coefficient. UV-vis absorbance spectra of **mepa-pic$_2$** at decreasing concentration (left) and analysis of spectra at 318 nm used to determine the molar extinction coefficient (right).

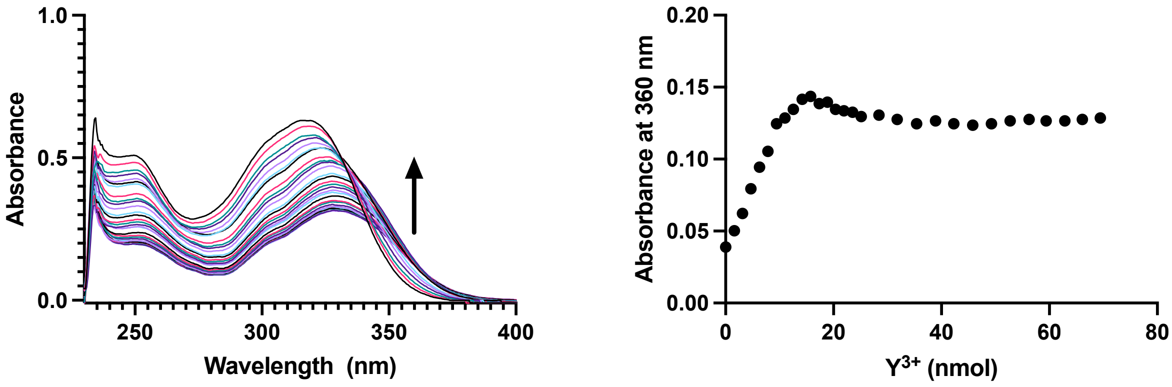

Figure S30. Spectrophotometric titration of **mepa$_2$-pic** with Y^{3+}. UV-vis absorbance spectra of **mepa$_2$-pic** upon Y^{3+} addition (left) and UV-vis titration to endpoint to determine ligand concentration (right).

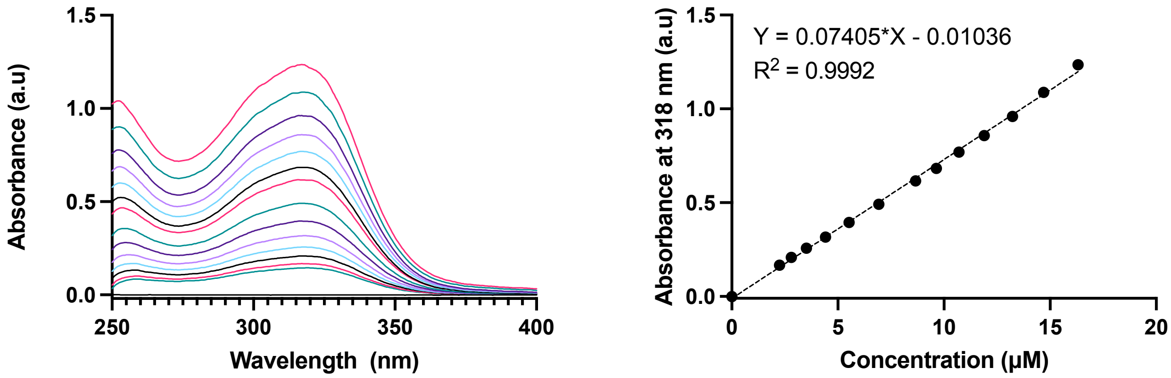

Figure S31. UV-Vis analysis of **mepa$_2$-pic** to determine molar extinction coefficient. UV-vis absorbance spectra of **mepa$_2$-pic** at decreasing concentration (left) and analysis of spectra at 318 nm used to determine the molar extinction coefficient (right).

2.2.2 Molar absorptivity determination of Eu-complexes.

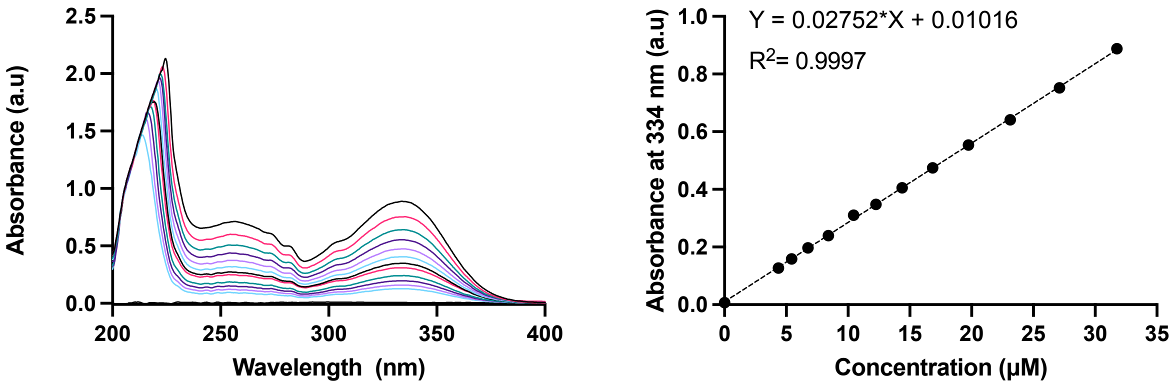

Figure S32. UV-Vis analysis of **^{nat}Eu-pepa-pic$_2$** to determine molar extinction coefficient. UV-vis absorbance spectra of **^{nat}Eu-pepa-pic$_2$** at decreasing concentration (left) and analysis of spectra at 334 nm used to determine the molar extinction coefficient (right).

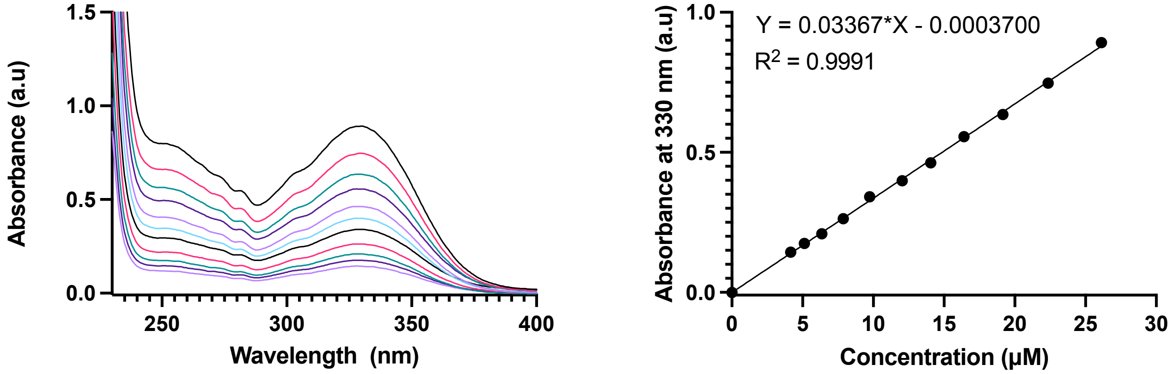

Figure S33. UV-Vis analysis of **^{nat}Eu-mepa-pic$_2$** to determine molar extinction coefficient. UV-vis absorbance spectra of **^{nat}Eu-mepa-pic$_2$** at decreasing concentration (left) and analysis of spectra at 334 nm used to determine the molar extinction coefficient (right).

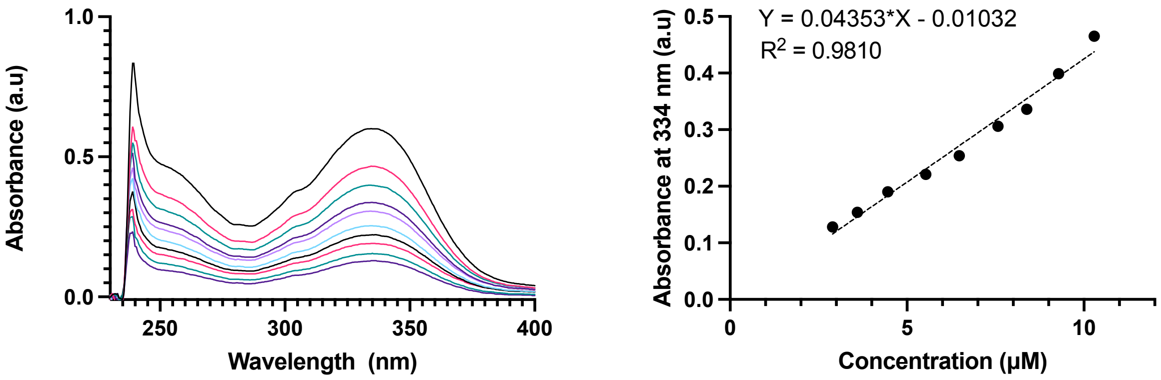

Figure S34. UV-Vis analysis of **^{nat}Eu-mepa$_2$-pic** to determine molar extinction coefficient. UV-vis absorbance spectra of **^{nat}Eu-mepa$_2$-pic** at decreasing concentration (left) and analysis of spectra at 334 nm used to determine the molar extinction coefficient (right).

2.2.3 Lifetimes and determination of the number of water molecules coordinated to Europium(III)

The lifetimes in H_2O (0.87 ms) and D_2O (1.13 ms), as well as inner hydration numbers (q=0) for **^{nat}Eu-pepa$_2$-pic** were previously determined and reported in our previous work.[5]

Europium complexes **^{nat}Eu-mepa-pic$_2$, ^{nat}Eu-mepa$_2$-pic and ^{nat}Eu-pepa-pic$_2$** were dissolved in DI water at concentrations such that the absorbance was equal or lower than 0.10 at 330 nm. For measurements conducted in D_2O, Eu^{III}-complexes were first dissolved in D_2O and then freeze dried overnight. The process was repeated three times before preparing the final solutions used to estimate their lifetimes. All measurements were done at room temperature.

Lifetime values were extracted by fitting the luminescent decay curves to the equation described below using GraphPad Prism:

$$I = (I_0 - P) * e^{-\frac{x}{\tau}} + P \qquad \text{Equation S1}$$

Where:

I is the luminescence intensity at 620 nm at any time t.

I$_0$ is the I value when X (time in milliseconds) is zero.

P is the *I* value at infinite times, expressed in the same units as *I*.

Tau is the luminescence lifetime, expressed in the same units as the X axis.

Instrument parameters:

Chopping speed: 40 Hz; Scan time: 20 ms; Excitation wavelength: 338.0 nm. Emission Wavelength: 620.0 nm. Excitation Slit: 5.0 nm. Emission Slit: 5.0 nm. PMT Voltage: 400 V. Response: 0.5 s

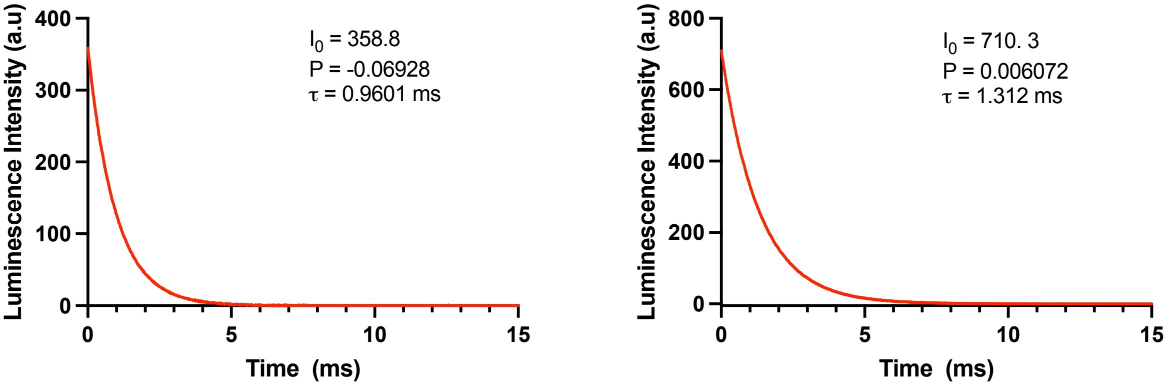

Figure S35. Luminescence lifetime data of ^{nat}**Eu-mepa-pic**$_{\mathbf{2}}$. Emission intensity decay in H_2O (left) and D_2O (right).

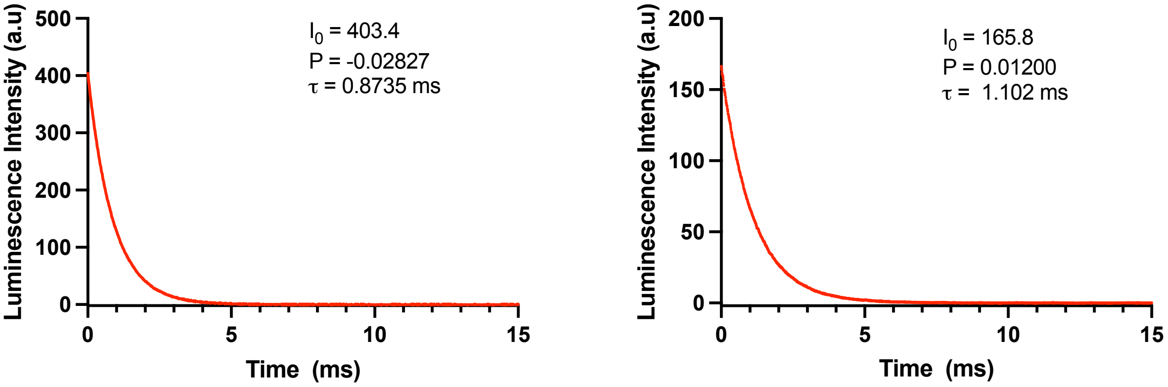

Figure S36. Luminescence lifetime data of ^{nat}**Eu-mepa**$_{\mathbf{2}}$**-pic**. Emission intensity decay in H_2O (left) and D_2O (right).

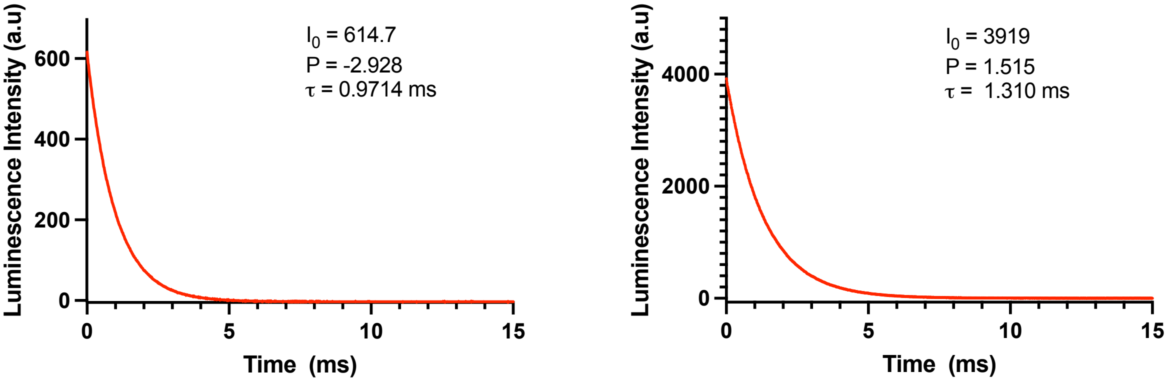

Figure S37. Luminescence lifetime data of ^{nat}**Eu-pepa-pic**$_{\mathbf{2}}$. Emission intensity decay in H_2O (left) and D_2O (right).

The determination of the number of water molecules, q, coordinated to Europium(III) ions in solution was calculated from luminescence decay lifetimes using Equation S2.

$$q = A\left[\left(\frac{1}{\tau_{H_2O}} - \frac{1}{\tau_{D_2O}}\right) - B\right] \qquad \text{Equation S2}$$

where τ_{H_2O} and τ_{D_2O} correspond to the measured luminescence decay lifetime (in milliseconds) in water or deuterated water. The constants A and B have been determined as A = 1.11 and B = 0.3135 by Horrocks;[6] or A = 1.2 and B = 0.2535 by Woods.[7]

2.2.4 Luminescence Quantum Yields

Luminescence quantum yields were measured at room temperature according to methods previously described in the literature[8, 9] and relative to the reference sample **^{nat}Eu-pepa$_2$-pic** (Ex= 355 nm, Φ =31.7%)[5]

Stock solutions of **^{nat}Eu-mepa-pic$_2$** and **^{nat}Eu-pepa$_2$-pic** were prepared in water, whereas **^{nat}Eu-mepa$_2$-pic** and **^{nat}Eu-pepa-pic$_2$** were prepared in 20% EtOH in water at concentrations such that the absorbance was 0.1 at 355 nm. Absorption spectra and luminescence emission spectra of series of dilutions of each Eu^{III}-complex (absorbance values ranging from 0.10 – 0.02 a.u) were recorded at room temperature.

For each Eu^{III}-complex, the luminescence emission spectra were integrated (from 500 – 800 nm) and plotted as a function of the absorbance at 355 nm. A linear fit was applied and the slope extracted to verify the linear relationship between luminescence intensity and concentration. Luminescence quantum yields were calculated according to Equation S3 as described in previous publications.[8, 9]

$$\Phi_s = \Phi_{ref}\frac{m_s}{m_{ref}}\left(\frac{n_s}{n_{ref}}\right)^2 \qquad \text{Equation S3}$$

Where:

Φ_s is the quantum yield of the sample

Φ_{ref} is the quantum yield of the reference

m_s is the slope of the fitted straight line of the luminescence vs absorbance plot for the sample

m_{ref} is the slope of the fitted straight line of the luminescence vs absorbance plot for the reference

n_s is the refractive index of the sample

n_{ref} is the refractive index of the reference

The refractive indexes of water and the mixture EtOH/H_2O (80/20) are 1.33252 and 1.34600, respectively.[10]

Luminescence and optical absorbance data from the reference sample **Eu-pepa$_2$-pic** were used to estimate the quantum yields of Eu^{III}-complexes samples measured on the same day. If measurements of investigated

Eu^{III}-complexes were conducted on a different day, fresh reference samples were prepared, and new data was collected accordingly.

Instrument parameters:

Chopping speed: 40 Hz; Excitation wavelength: 355.0 nm. Emission range: 360.0 – 800.0 nm. Excitation Slit: 1.0 nm. Emission Slit: 1.0 nm. PMT Voltage: 700 V. Response: 0.05 s. Scan speed: 1200 nm/min.

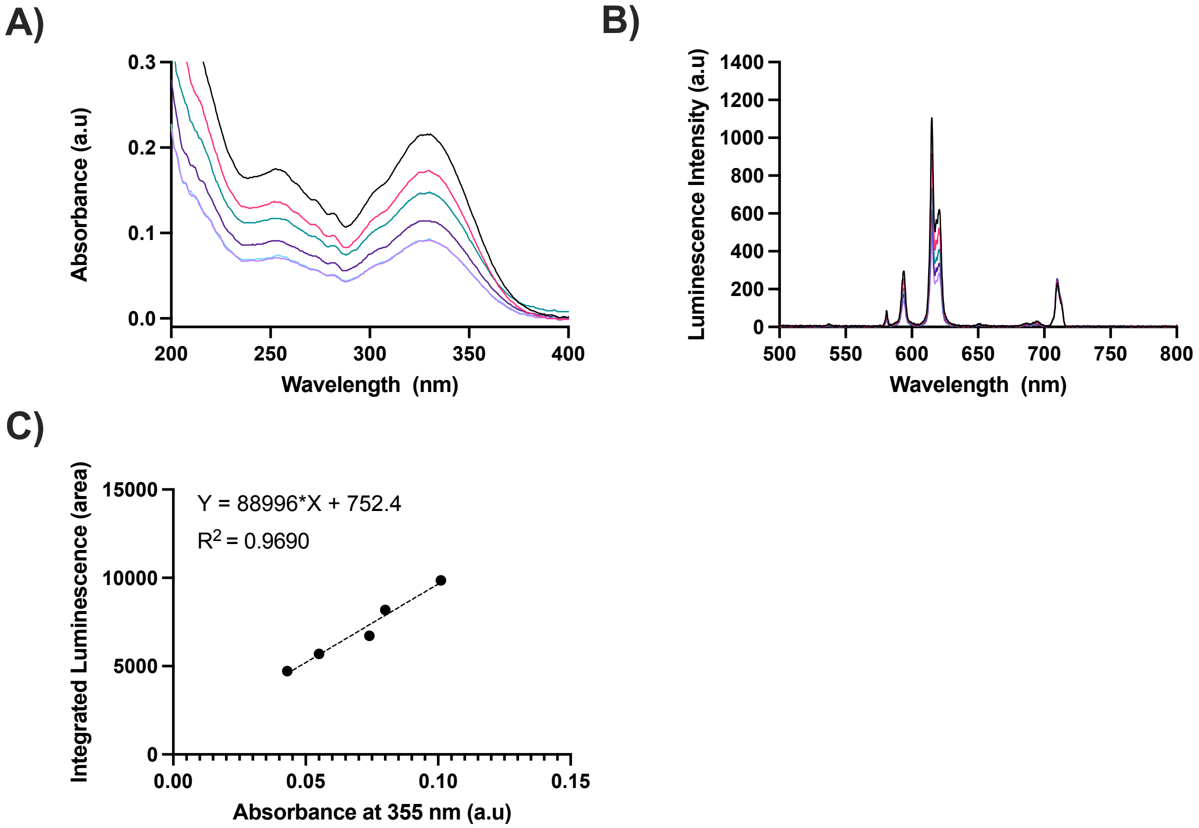

Figure S38. Data for the determination of the luminescence quantum yield of ^{nat}**Eu-mepa-pic$_2$**. A) UV-Vis spectra for solutions with various concentrations of ^{nat}**Eu-mepa-pic$_2$** in water. B) Luminescence spectra of solutions of ^{nat}**Eu-mepa-pic$_2$**. C) Plot of the integrated luminescence emission intensity of ^{nat}**Eu-mepa-pic$_2$** (500 – 800 nm) vs its absorbance at five different concentrations. The slope of the resulting linear fit is used to calculate the quantum yield by comparing it to the slope of a reference sample.

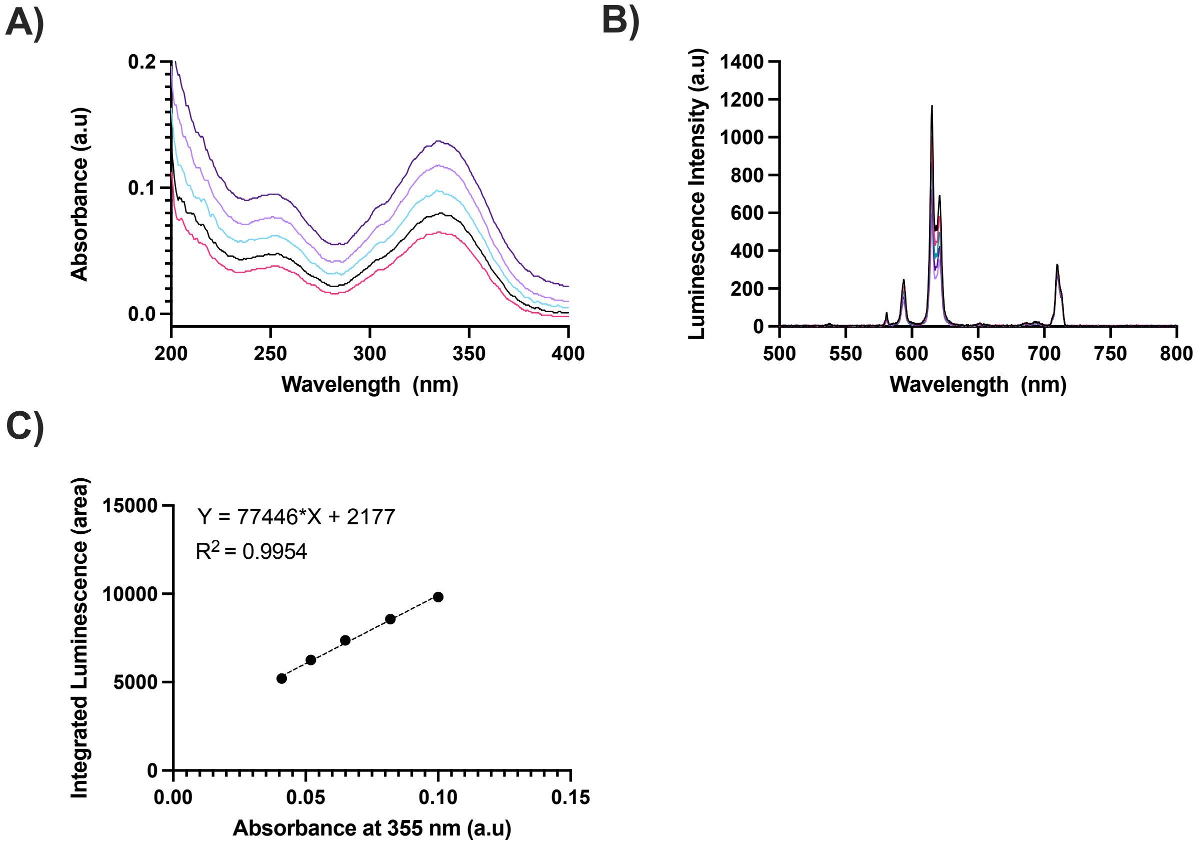

Figure S39. Data for the determination of the luminescence quantum yield of **^{nat}Eu-mepa$_2$-pic**. A) UV-Vis spectra for solutions with various concentrations of **^{nat}Eu-mepa$_2$-pic** in 20% EtOH in water. B) Luminescence spectra of solutions of **^{nat}Eu-mepa$_2$-pic**. C) Plot of the integrated luminescence emission intensity of **^{nat}Eu-mepa$_2$-pic** (500 – 800 nm) vs its absorbance at five different concentrations. The slope of the resulting linear fit is used to calculate the quantum yield by comparing it to the slope of a reference sample.

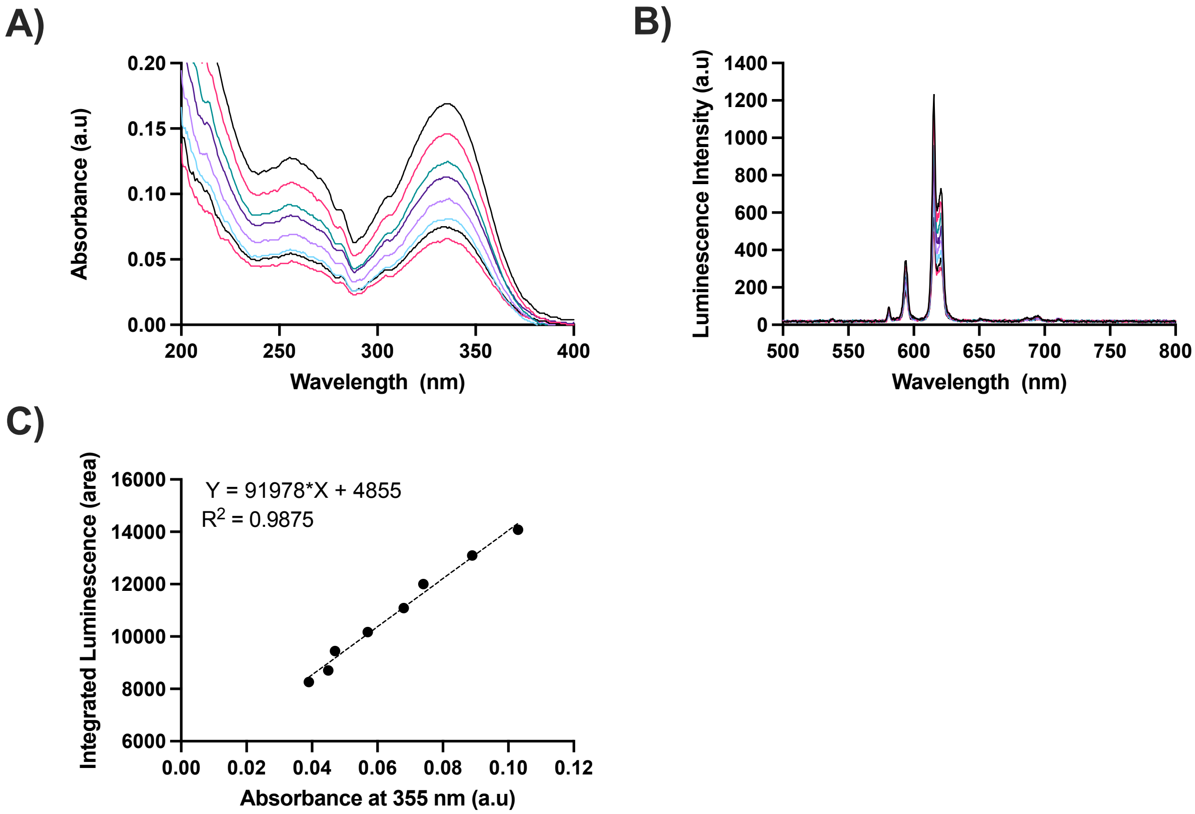

Figure S40. Data for the determination of the luminescence quantum yield of **^{nat}Eu-pepa-pic$_2$**. A) UV-Vis spectra for solutions with various concentrations of **^{nat}Eu-pepa-pic$_2$** in 20% EtOH in water. B) Luminescence spectra of solutions of **^{nat}Eu-pepa-pic$_2$**. C) Plot of the integrated luminescence emission intensity of **^{nat}Eu-pepa-pic$_2$** (500 – 800 nm) vs its absorbance at eight different concentrations. The slope of the resulting linear fit is used to calculate the quantum yield by comparing it to the slope of a reference sample.

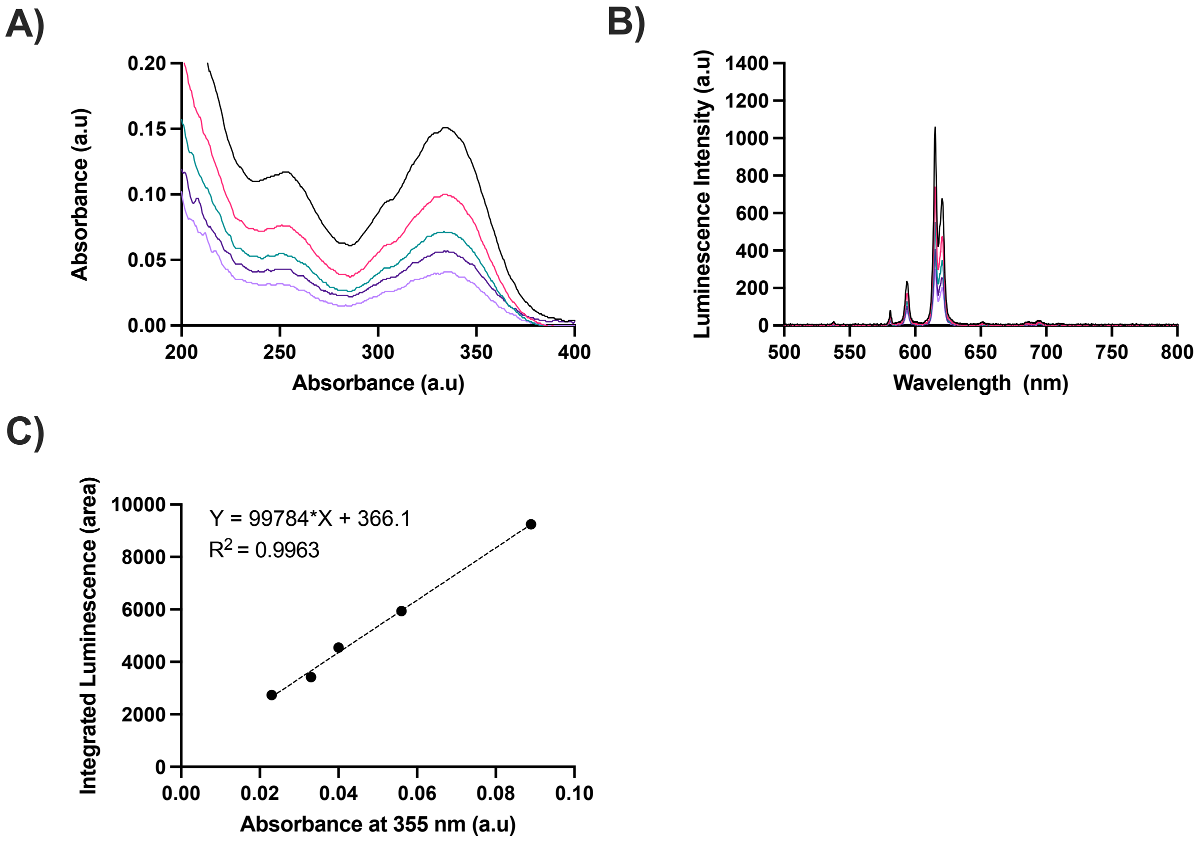

Figure S41. Luminescence emission and optical absorbance data for reference complex **^{nat}Eu-pepa$_2$-pic**. A) UV-Vis spectra for solutions with various concentrations of **^{nat}Eu-pepa$_2$-pic** in water. B) Luminescence spectra of solutions of **^{nat}Eu-pepa$_2$-pic**. C) Plot of the integrated luminescence emission intensity of **^{nat}Eu-pepa$_2$-pic** (500 – 800 nm) vs its absorbance at five different concentrations. The slope of the resulting linear fit is used as the slope of the reference sample required to calculate the quantum yields of **^{nat}Eu-mepa-pic$_2$** and **^{nat}Eu-mepa$_2$-pic**.

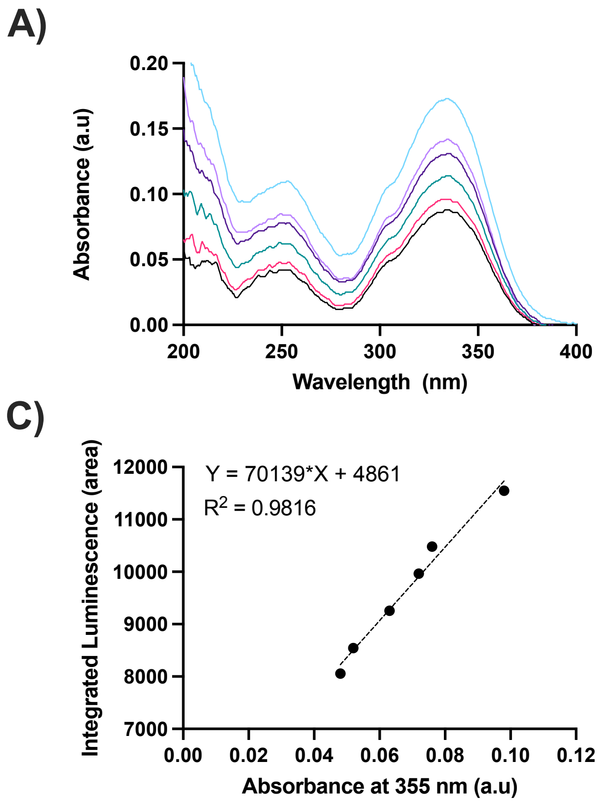

Figure S42. Luminescence emission and optical absorbance data for reference complex **^{nat}Eu-pepa$_2$-pic**. A) UV-Vis spectra for solutions with various concentrations of **^{nat}Eu-pepa$_2$-pic** in water. B) Luminescence spectra of solutions of **^{nat}Eu-pepa$_2$-pic**. C) Plot of the integrated luminescence emission intensity of **^{nat}Eu-pepa$_2$-pic** (500 – 800 nm) vs its absorbance at five different concentrations. The slope of the resulting linear fit is used as the slope of the reference sample required to calculate the quantum yield of **^{nat}Eu-pepa-pic$_2$**.

3 Cerenkov Radiation-mediated Energy Transfer Phantom Imaging Assays

Solutions of **^{nat}Eu-mepa-pic$_2$, ^{nat}Eu-mepa$_2$-pic, ^{nat}Eu-pepa-pic$_2$** and **^{nat}Eu-pepa$_2$-pic** (0.1 – 50 nmol) were prepared in a mixture of DPBS 1X buffer (170 µL) and DMSO (32 µL) in microcentrifuge Eppendorf tubes. The solutions were doped with [^{68}Ga]GaCl$_3$ or Y-90 (10 µL, 10-19 µCi), vortexed and spined down for 5 seconds. Real-time luminescence imaging was recorded in a Perkin Elmer IVIS Lumina Series III imaging system at room temperature. Eppendorf tube plastic caps are removed prior to imaging.

Imaging parameters:

Excitation filter: No excitation (excitation channel blocked); emission Filter: 620 nm; binning factor of 4; field of view set to '22.4' and exposure time of 5 minutes. Images and regions of interest (ROI) analysis was carried out using Living Image software (version 4.8.2).

The limit of detection (LoD) was calculated according to the equation depicted below:

$$LoD = bg + 3\sigma \qquad \textbf{Equation S4}$$

Where:

bg is the average value of the background measured and σ is the standard deviation of the background measurements.

4 Radiolabeling Studies

4.1 Radiochemical synthesis and characterization of non-targeted complexes with Y-86.

Stock solutions of **pepa-pic$_2$**, **mepa-pic$_2$** (DMSO 3% in H_2O) and **pepa$_2$-pic** (DMSO 18% in water) were freshly prepared, and their concentrations checked by UV-Vis.

4.1.1 Radiosynthesis of [^{86}Y]Y-mepa-pic$_2$

To [^{86}Y]YCl_3 (8.0 μL, 276 μCi) and mepa-pic$_2$ (20 μL, 2 mM) in 50 μL of water was added NaOAc (40 μL, 1 M, pH 5.5) and DMSO (10 μL). The resulting mixture was vortexed and heated at 85 °C for 25 minutes. Quantitative labeling was confirmed via radio-HPLC (Method D, R_t = 8.28 min – Absorbance monitored at 254 nm).

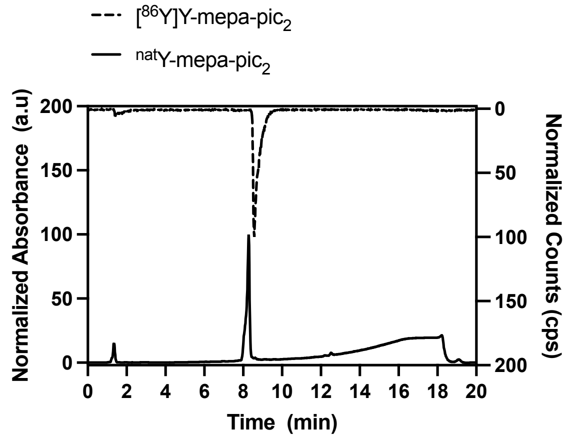

Figure S43. Chromatographic analysis of **[^{86}Y]Y-mepa-pic$_2$** and its corresponding non-radioactive reference complex. Absorbance of non-radioactive samples was monitored at 254 nm.

4.1.2 Radiosynthesis of [^{86}Y]Y-pepa-pic$_2$

To [^{86}Y]YCl_3 (7.5 μL, 256 μCi) and pepa-pic2 (10 μL, 2 mM) in 50 μL of water was added NaOAc (40 μL, 1 M, pH 5.5). The resulting mixture was vortexed and heated at 85 °C for 15 minutes. Quantitative labeling was confirmed via radio-HPLC (Method D, R_t = 8.10 min – Absorbance monitored at 254 nm).

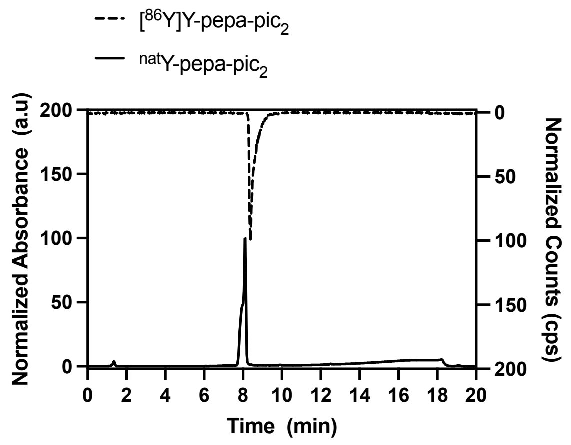

Figure S44. Chromatographic analysis of **[86Y]Y-pepa-pic2** and its corresponding non-radioactive reference complex. Absorbance of non-radioactive samples was monitored at 254 nm.

4.1.3 Radiosynthesis of [86Y]Y-pepa2-pic

To [86Y]YCl_3 (250 µL, 288 µCi) and pepa2-pic (20 µL, 2 mM) was added NaOAc (70 µL, 2 M, pH 5.5) and DMSO (10 uL). The resulting mixture was vortexed and heated at 85 °C for 15 minutes. Quantitative labeling was confirmed via radio-HPLC (Method D, R_t = 9.62 min – Absorbance monitored at 254 nm).

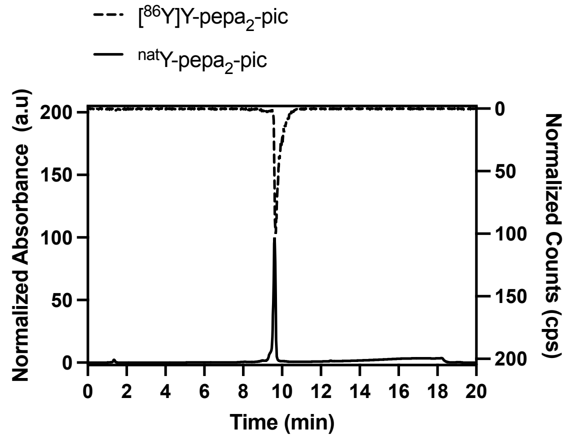

Figure S45. Chromatographic analysis of **[86Y]Y-pepa2-pic** and its corresponding non-radioactive reference complex. Absorbance of non-radioactive samples was monitored at 254 nm**.**

4.2 Radiochemical synthesis and characterization of targeted complexes of pepa-pic$_2$-C-Hex-KuE with Y-86, Lu-177 and Tb-161.

Stock solutions of **pepa-pic$_2$-C-Hex-KuE** and **PSMA-617** (ε_{277} = 4268 M^{-1} cm^{-1})[11] were freshly prepared in water and their concentrations estimated via UV-Vis. The molar extinction coefficient of the non-targeted analog **pepa-pic$_2$** (ε_{318} = 24950 M^{-1} cm^{-1}) was used to calculate the concentration of **pepa-pic$_2$-C-Hex-KuE** in solution.

[^{161}Tb]TbCl_3 was purchased from Terthera Terbium Theranostics and received at a specific activity of 377 mCi/mL in HCl 0.05 M. [^{177}Lu]LuCl_3, on the other hand, was acquired from Shine Technologies, and received in a solution of HCl 0.04 M at a specific activity of 625 mCi/mL.

4.2.1 Radiosynthesis of [^{86}Y]Y-pepa-pic$_2$-C-Hex-KuE

To a solution of [^{86}Y]YCl_3 (19.0 µL, 408 µCi) and **pepa-pic$_2$-C-Hex-KuE** (55.0 µL, 77.2 µM) was added NaOAc buffer (40.0 µL, 2 M, pH 5.5) and water (86.0 µL). The resulting mixture was vortexed and heated at 85 °C for 15 minutes. Quantitative labeling was confirmed via radio-HPLC (Method D, R_t = 7.78 min – Absorbance monitored at 254 nm).

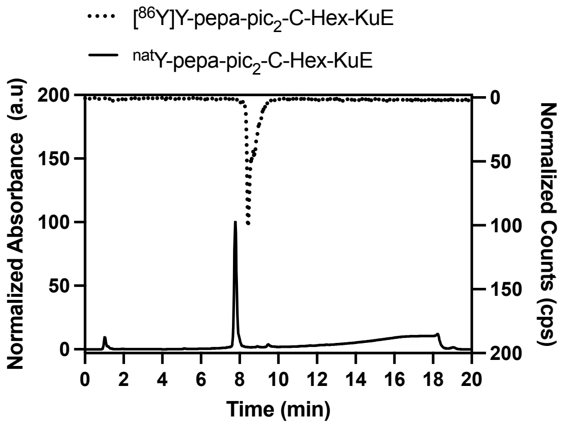

Figure S46. Chromatographic analysis of **[^{86}Y]Y-pepa-pic$_2$-C-Hex-KuE** and its corresponding non-radioactive reference complex. Absorbance of non-radioactive samples was monitored at 254 nm.

4.2.2 Radiosynthesis of [^{177}Lu]Lu-pepa-pic$_2$-C-Hex-KuE

[^{177}Lu]LuCl_3 (20.0 µL, 3.00 mCi) and pepa-pic$_2$-C-Hex-KuE (150 µL, 517 µM) were dissolved in a mixture of NaOAc buffer (40.0 µL, 0.5 M, pH 5.5) and DMSO (20.0 µL). The resulting mixture was vortexed and heated at 80 °C for 50 minutes. Quantitative labeling was confirmed via radio-HPLC (Method D, R_t = 7.17 min – Absorbance monitored at 254 nm).

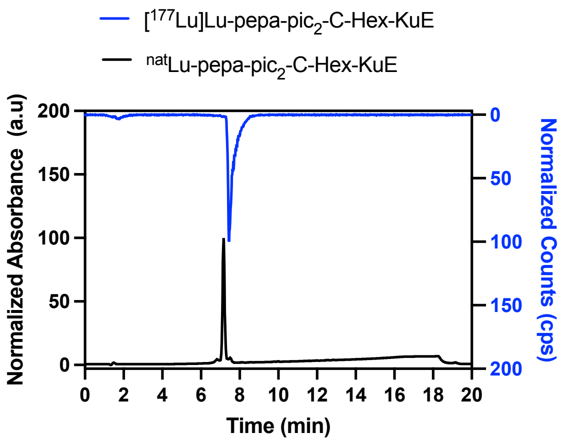

Figure S47. Chromatographic analysis of **[^{177}Lu]Lu-pepa-pic$_2$-C-Hex-KuE** and its corresponding non-radioactive reference complex. Absorbance of non-radioactive samples was monitored at 254 nm**.**

4.2.3 Radiosynthesis of [^{161}Tb]Tb-pepa-pic$_2$-C-Hex-KuE

To a solution of [^{161}Tb]$TbCl_3$ (9.34 µL, 3.52 mCi) and **pepa-pic$_2$-C-Hex-KuE** (46.2 µL, 2.59 mM) was added NaOAc buffer (20.0 µL, 1 M, pH 5.5). The resulting mixture was vortexed and heated at 80 °C for 20 minutes. Purification of the ^{161}Tb-labeled tracer was carried out via radio-HPLC (Method D, R_t = 7.13 min – Absorbance monitored at 254 nm). Solvent was evaporated using a stream of N_2 gas, and the resulting tracer resuspended in appropriate media suitable for *in vivo* studies.

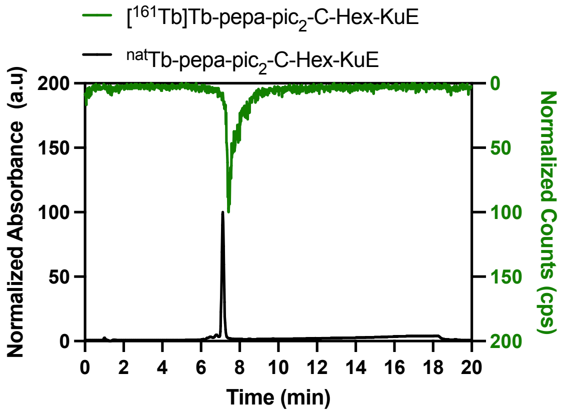

Figure S48. Chromatographic analysis of **[^{161}Tb]Tb-pepa-pic$_2$-C-Hex-KuE** and its corresponding non-radioactive complex. Absorbance of non-radioactive samples was monitored at 254 nm**.**

4.2.4 Radiosynthesis of [^{177}Lu]Lu-PSMA-617

[^{177}Lu]$LuCl_3$ (20.0 µL, 3.02 mCi) and **PSMA-617** (11.2 µL, 5.36 mM) were dissolved in a mixture of NaOAc buffer (80.0 µL, 0.5 M, pH 5.5), DMSO (10.0 µL) and water (80.0 µL). The resulting mixture was vortexed and heated at 80 °C for 30 minutes. Quantitative labeling was confirmed via radio-HPLC HPLC (Method D, R_t = 7.77 min – Absorbance monitored at 254 nm).

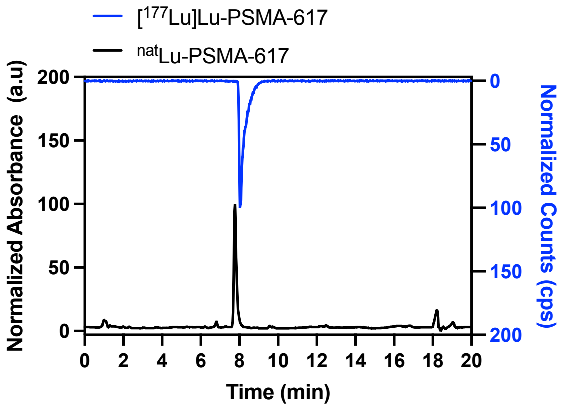

Figure S49. Chromatographic analysis of **[^{177}Lu]Lu-PSMA-617** and its corresponding non-radioactive reference complex. Absorbance of non-radioactive samples was monitored at 254 nm.

4.2.5 Radiosynthesis of [^{161}Tb]Tb-PSMA-617

[^{161}Tb]TbCl$_3$ (9.34 µL, 4.0 mCi) and **PSMA-617** (12.2 µL, 9.80 µM) were dissolved in a mixture of NaOAc buffer (20.0 µL, 1 M, pH 5.5), DMSO (20.0 µL) and water (38.4 µL). The resulting mixture was vortexed and heated at 80 °C for 20 minutes. Quantitative labeling was confirmed via radio-HPLC (Method D, R_t = 7.73 min – Absorbance monitored at 254 nm).

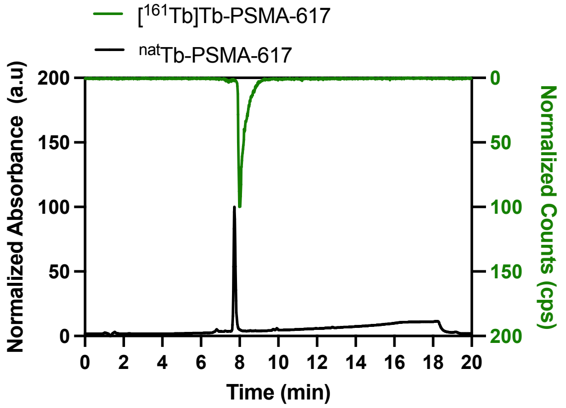

Figure S50. Chromatographic analysis of **[^{161}Tb]Tb-PSMA-617** and its corresponding non-radioactive reference complex. Absorbance of non-radioactive samples was monitored at 254 nm.

4.3 LogD$_{7.4}$ Lipophilicity Measurements

pepa-pic$_2$, **mepa-pic$_2$, pepa$_2$-pic** and **pepa$_2$-pic-PSMA** were radiolabeled with [^{86}Y]YCl$_3$ and formulated in DPBS 1X pH 7.4 at a concentration of 25-29 µCi/mL. 1-Octanol (500 µL, previously washed with DPBS 2X) was mixed with ^{86}Y-radiolabeled tracer in DPBS 1X (500 µL, 12.5 – 14.5 µCi). Mixtures were vortexed and shaken at room temperature for 1 hour. Samples were centrifuged for 5 min at 5000 rpm to separate

both layers. Aliquots (100 μL) of each layer were removed and the concentration of ^{86}Y-radiolabeled tracer estimated using gamma counting (Hidex Automatic Gamma Counter). $LogD_{7.4}$ values calculated according to equation S5.

$$\log D_{7.4} = \log \frac{[C_{Octanol}]}{[C_{DPBS}]} \quad \text{Equation S5}$$

Where C is the concentration of radiotracer found in the octanol or PBS layer.

5 In vivo studies

All animal experiments were conducted with the approval of the University of Wisconsin-Madison Institutional Animal Care and Use Committee (IACUC). All studies were conducted in accordance with the relevant guidelines and regulations and approved under protocol number M006738 (PI: Boros) and conducted at UW-Madison School of Medicine and Public Health, at the Small Animal Imaging and Radiotherapy Facility (SAIRF).

5.1 In vivo biodistribution of non-targeted constructs in naïve BALB/c mice.

Female BALB/c mice were purchased from Jackson Laboratory. Biodistribution profiles of Y-86 labeled **mepa-pic$_2$, pepa-pic$_2$ and pepa$_2$-pic$_2$** were first evaluated in healthy naïve mice. To this end, 100 μL (containing 20-35 μCi) of radiotracer formulated in DPBS 1X buffer were administered via tail vein injection into 8–10-week-old female balb/c mice (n = 4 per cohort). Mice were euthanized 1 hour post injection and biodistribution studies were conducted. Urine and blood were collected, organs were harvested, intestinal content removed, and activity assessed via a gamma counting (Hidex Automatic Gamma Counter). Recorded measurements of counts per minute (cpm) were decay-corrected to the time of injection of the radiotracer, converted to units of Ci by using the efficiency of the Gamma Counter for Ga-68, and the amount of activity present in each organ was expressed as percentage of injected dose per gram of organ weight (% ID/g).

Table S1. Tabulated values for the biodistribution of [^{86}Y]Y-mepa-pic$_2$, [^{86}Y]Y-pepa-pic$_2$, and [^{86}Y]Y-pepa$_2$-pic in naïve BALB/c mice 1-hour post-injection of radiotracer. Values expressed as % ID/g. Error expressed as ± 1 SD for n = 4.

Organs	[^{86}Y]Y-mepa-pic$_2$	[^{86}Y]Y-pepa-pic$_2$	[^{86}Y]Y-pepa$_2$-pic
Blood	1.66 ± 0.90	0.78 ± 0.19	11.79 ± 0.72
Heart	1.63 ± 0.59	1.26 ± 0.50	4.34 ± 0.46
Lungs	2.26 ± 0.88	1.75 ± 0.50	9.05 ± 1.46
Liver	4.94 ± 2.70	1.48 ± 0.39	4.78 ± 0.25
Spleen	3.07 ± 1.47	2.62 ± 1.52	2.96 ± 0.55
Kidney	7.75 ± 3.57	4.65 ± 1.26	12.24 ± 1.15
Small Intestine	6.17 ± 2.13	3.46 ± 1.31	2.02 ± 0.39
Large Intestine	0.68 ± 0.28	0.97 ± 0.36	1.67 ± 0.54
Muscle	0.84 ± 0.13	0.48 ± 0.21	1.38 ± 0.36
Bone	1.89 ± 0.38	1.07 ± 0.46	2.47 ± 0.38

5.1.1 Urine metabolite analysis

Metabolite analysis was performed by analyzing aliquots of 100 µL of mouse urine collected during biodistribution studies. If the collected urine volume was less than 100 µL, 1X DPBS was added in order to bring it up to a final volume of 100 µL. In cases where the radioactivity present in urine decayed to levels too low for detection by HPLC, fractions eluted from the radioHPLC were collected every 30 seconds, and the amount of radioactivity was estimated via gamma counting (Hidex Automatic Gamma Counter). The chromatogram was then reconstructed by plotting the recorded counts per minute recorded for each fraction as a function of time.

5.2 *In vitro* optical imaging

Tumor tissues were harvested from male, nude, athymic mice bearing xenografts from PC3-PIP (PSMA+) and PC3-flu (PSMA-) were fixed for (24) hours in 10% neutral buffered formalin, processed and embedded into paraffin blocks. Tissues were sectioned at 5µm, dried and then deparaffinized with a 25-minute oven bake at 60°C, followed by a series of xylene and alcohol washes

Antigens were prepared in PerkinElmer's 1X Antigen Retrieval pH 9.0 (AR900250ML) for 8 minutes in the microwave. Tissue sections were blocked with 1X Antibody Diluent/Block buffer (Akoya).

Ab-PSMA, ^{nat}Eu-pepa-pic$_2$-C-Hex-KuE and DAPI staining.

Tissues were stained with Ab-PSMA (Ventana 760-6076 ; 11.75 µg/mL ; 100uL per slide) for 30 minutes at room temperature. Slides were washed with TBS-T and secondary goat anti-rabbit was applied for 10 minutes at room temperature. Slides were washed with TBS-T and Akoya's Opal dye 520 was applied for 10 minutes at room temperature. Antigen retrieval and blocking steps were performed again.

An aqueous solution of ^{nat}Eu-pepa-pic$_2$-C-Hex-KuE (10 µM) was diluted at 1:1 with 1X Diluent/Block (Akoya) and 100 µL were applied per slide for 2 hours at room temperature. For DAPI staining, DAPI working solution (Akoya) was applied for 10 minutes at room temperature before mounting with ProLong™ Gold Antifade Mountant. Slides were imaged by Nuance Multispectral Scope at 20x objective.

5.3 In vivo biodistribution in PSMA +/- mice models, PET-CT and SPECT imaging.

Male, nude, athymic mice were purchased from Jackson Laboratory. Ten days preceding the imaging study, mice were implanted with 1×10^6 cells (PC-3 PIP on the right, PC-3 flu on the left shoulder) in 100 µL of a 1:2 mixture of DPBS 1X/matrigel matrix subcutaneously.

A solution of 100 µL of labeled radiotracer was administered via tail vein injection into 8–10-week-old male nude mice (n = 4 per cohort) bearing PSMA+/- tumors.

For biodistribution studies, mice were euthanized at 2 hours (n=4) post-injection of the radio tracer. Organs were extracted, intestinal content removed, and activity assessed via a gamma counter. Biodistribution analysis values are represented as percentage of injected dose per gram of organ weight (% ID/g).

Table S2. Tabulated values for the biodistribution **pepa-pic$_2$-C-Hex-KuE** radiolabeled with Y-86, Lu-177 and Tb-161 in a PSMA+/- xenograft mouse model 2-hour post-injection. Values expressed as % ID/g. Error expressed as ± 1 SD for n = 4.

Organs	[^{86}Y]Y^{III}-pepa-pic$_2$-C-Hex-KuE	[^{177}Lu]Lu^{III}-pepa-pic$_2$-C-Hex-KuE	[^{161}Tb]Tb^{III}-pepa-pic$_2$-C-Hex-KuE
Blood	1.22 ± 1.01	0.30 ± 0.10	0.45 ± 0.06
Heart	0.54 ± 0.25	0.19 ± 0.07	0.21 ± 0.03
Lungs	1.96 ± 0.84	0.47 ± 0.26	0.50 ± 0.07
Liver	1.00 ± 0.32	0.50 ± 0.05	0.64 ± 0.09
Spleen	2.43 ± 1.34	0.53 ± 0.24	0.50 ± 0.09
Kidney	60.48 ± 15.62	25.88 ± 8.51	17.89 ± 2.70
Small Intestine	0.59 ± 0.25	0.33 ± 0.27	0.33 ± 0.37
Large Intestine	1.64 ± 0.79	0.49 ± 0.27	0.98 ± 1.44
Muscle	0.35 ± 0.13	0.11 ± 0.05	0.11 ± 0.03
Bone	0.74 ± 0.21	0.25 ± 0.02	0.24 ± 0.03
Tumor +	12.53 ± 2.95	4.57 ± 0.90	7.33 ± 1.59
Tumor -	1.20 ±0.45	-	0.70 ± 1.59

Representative PET-CT (Y-86) or SPECT (Lu-177, Tb-161) images were acquired at different time points starting as early as 0.5 h and up to 25-hours post administration of radiotracer. Mice were initially anesthetized with isoflurane gas at 4% isoflurane in oxygen and maintained during scans at 2.0%. PET-CT and SPECT images were normalized to units of percentage of injected dose per cubic centimeter and presented as maximum intensity projected scan (MIPS) images.

PET-CT scans were acquired in a Siemens Inveon Hybrid MicroPET/CT Scanner (Siemens Medical Solutions USA, Inc., Knoxville, TN), and image processing and region of interest (ROI) analysis conducted using the Inveon Research Workstation analysis software. ROI analysis of the PET/CT images was used to estimate tracer uptake in major organs or tissues, and quantitative results are given as percentage of injected dose per cubic centimeter (%ID/cc).

SPECT scans, on the other hand, were collected in a MILabs U-SPECT/CTUHR (ultra high-resolution) system and image processing was carried out using Imalytics preclinical image analysis software.

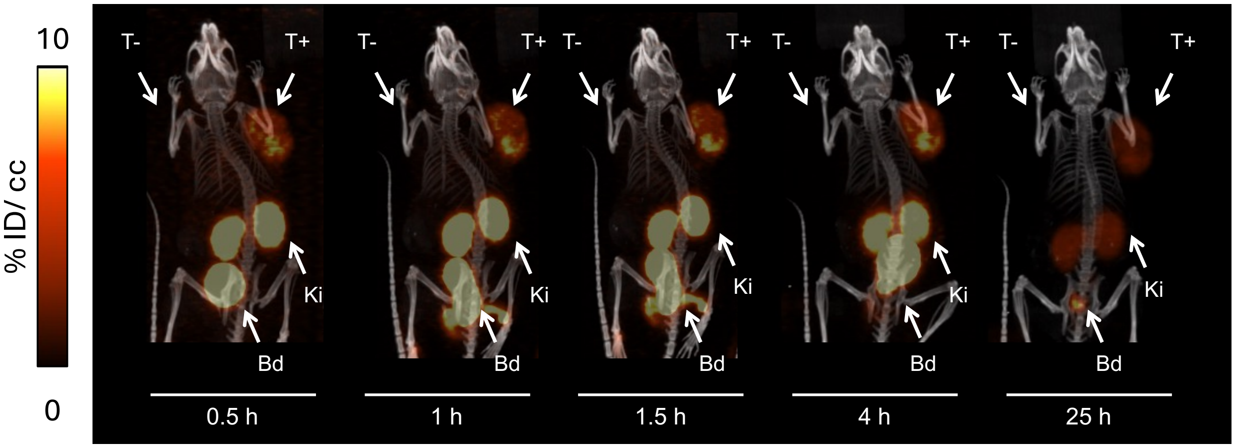

Figure S51. Representative longitudinal in vivo PET/CT imaging of **[^{86}Y]Y-pepa-pic$_2$-C-Hex-KuE** 0.5, 1, 1.5, 4- and 25-hours post administration of radiotracer to nude mice bearing PSMA +/- tumors. Images are normalized to units of %ID/cc and presented as maximum intensity projection scans (MIPS). Activity is primarily shown in the bladder (Bd), kidneys (Ki), and PSMA-positive tumor (T+). % ID/cc is the percentage of injected dose per cubic centimeter. Initial dose of radiotracer administered: 91 μCi.

Table S3. Region of interest (ROI) quantitative analysis of *in vivo* PET-CT images of a mouse bearing PSMA +/- tumors. Results are given as percentage of injected dose per cubic centimeter (%ID/cc).

**Organs**	**0.5 h**	**1 h**	**1.5 h**	**4 h**	**25 h**
Tumor +	4.6 ± 1.7	4.4 ± 1.8	5.2 ± 1.8	5.2 ± 1.7	3.3 ± 1.0
L. Kidney	12.7 ± 1.6	12.2 ± 5.5	12.2 ± 3.4	8.8 ± 1.9	4.0 ± 1.2
R. Kidney	12.0 ± 2.4	11.9 ± 3.0	12.7 ± 2.9	8.8 ± 2.3	4.0 ± 1.2
Muscle	0.8 ± 0.3	0.8 ± 0.4	0.6 ± 0.4	0.4 ± 0.2	0.3 ± 0.3
Heart	1.7 ± 0.5	1.2 ± 0.3	1.0 ± 0.5	0.5 ± 0.3	0.1 ± 0.1
Bone	1.3 ± 0.5	1.0 ± 0.5	0.8 ± 0.4	0.5 ± 0.3	0.3 ± 0.2
Liver	1.4 ± 0.5	1.1 ± 0.3	0.9 ± 0.4	0.7 ± 0.4	0.3 ± 0.2

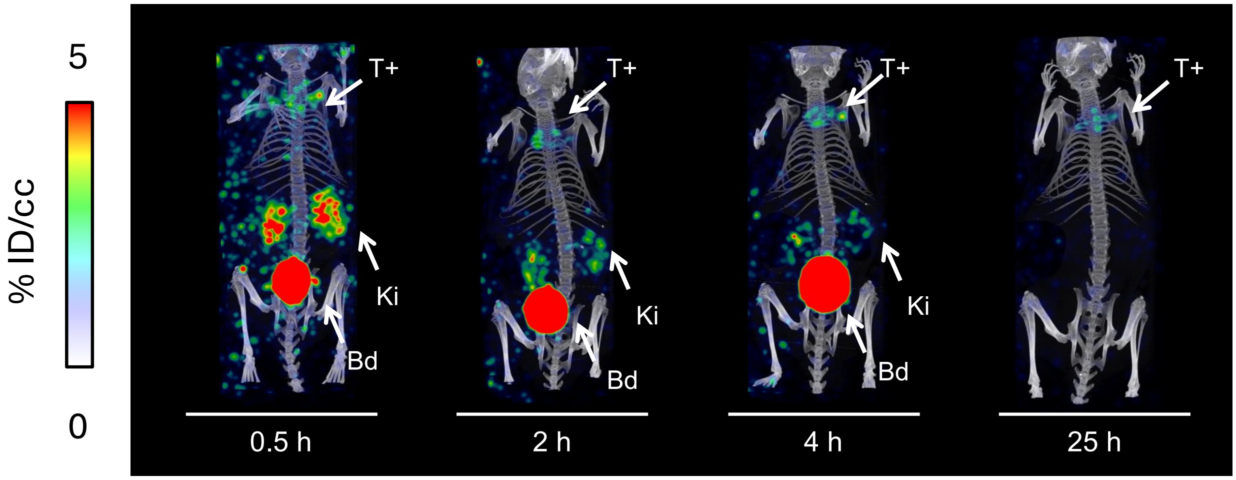

Figure S52. Representative longitudinal in vivo SPECT imaging of **[^{177}Lu]Lu-PSMA-617** 0.5, 2, 4- and 25-hours post administration of radiotracer to nude mice bearing PSMA + tumors. Images are normalized to units of %ID/cc and presented as maximum intensity projection scans (MIPS). Activity is primarily shown in the bladder (Bd), kidneys (Ki), and PSMA-positive tumor (T+). % ID/cc is the percentage of injected dose per cubic centimeter. Initial dose of radiotracer administered: 310 µCi.

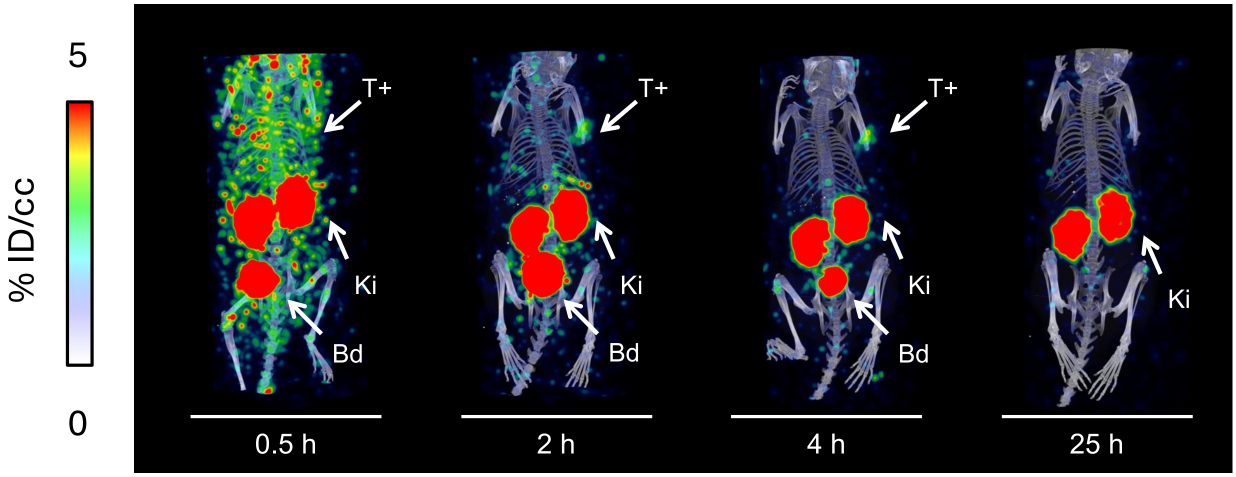

Figure S53. Representative longitudinal in vivo SPECT imaging of **[^{177}Lu]Lu-pepa-pic$_2$-C-Hex-KuE** 0.5, 2, 4- and 25-hours post administration of radiotracer to nude mice bearing PSMA + tumors. Images are normalized to units of %ID/cc and presented as maximum intensity projection scans (MIPS). Activity is primarily shown in the bladder (Bd), kidneys (Ki), and PSMA-positive tumor (T+). % ID/cc is the percentage of injected dose per cubic centimeter. Initial dose of radiotracer administered: 313 µCi.

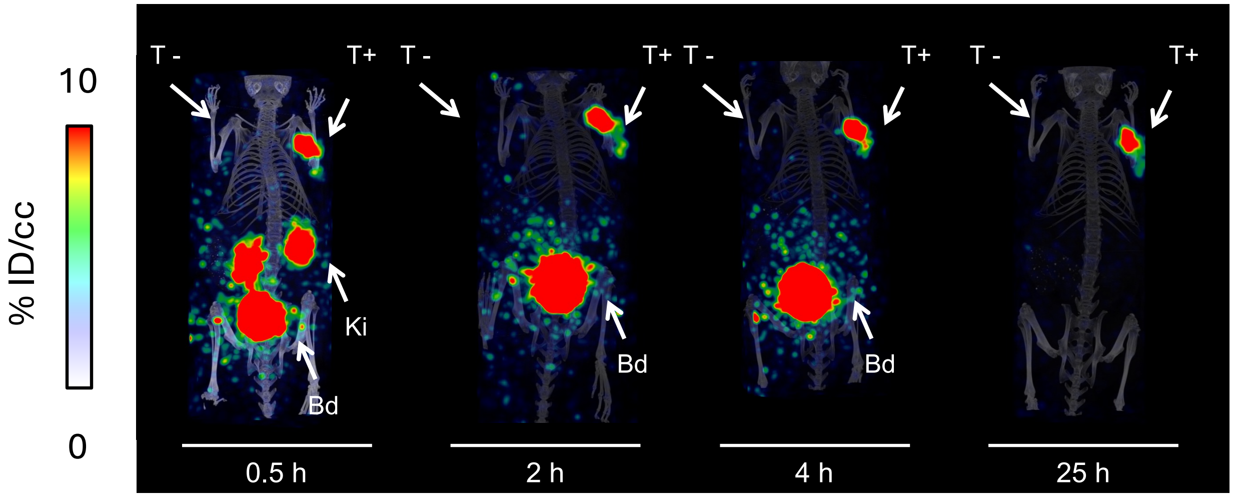

Figure S54. Representative longitudinal *in vivo* SPECT imaging of **[^{161}Tb]Tb-PSMA-617** 0.5, 2, 4- and 25-hours post administration of radiotracer to nude mice bearing PSMA +/- tumors. Images are normalized to units of %ID/cc and presented as maximum intensity projection scans (MIPS). Activity is primarily shown in the bladder (Bd), kidneys (Ki), and PSMA-positive tumor (T+). % ID/cc is the percentage of injected dose per cubic centimeter. Initial dose of radiotracer administered: 325 μCi.

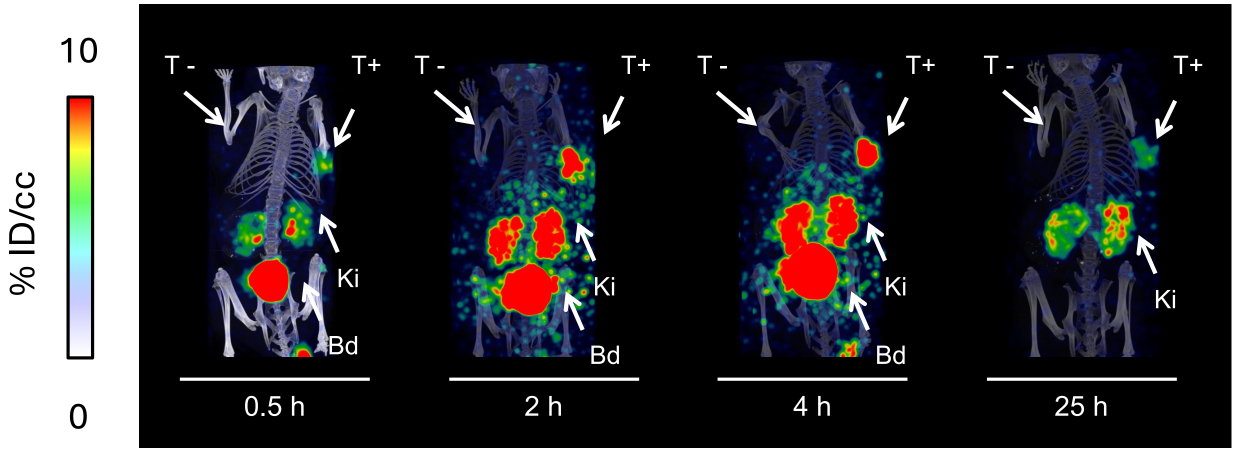

Figure S55. Representative longitudinal in vivo SPECT imaging of **[^{161}Tb]Tb- pepa-pic$_2$-C-Hex-KuE** 0.5, 2, 4- and 25-hours post administration of radiotracer to nude mice bearing PSMA +/- tumors. Images are normalized to units of %ID/cc and presented as maximum intensity projection scans (MIPS). Activity is primarily shown in the bladder (Bd), kidneys (Ki), and PSMA-positive tumor (T+). % ID/cc is the percentage of injected dose per cubic centimeter. Initial dose of radiotracer administered: 267 μCi.

5.3.1 Urine metabolite analysis

Metabolite analysis was performed as previously described in section 5.1.1.

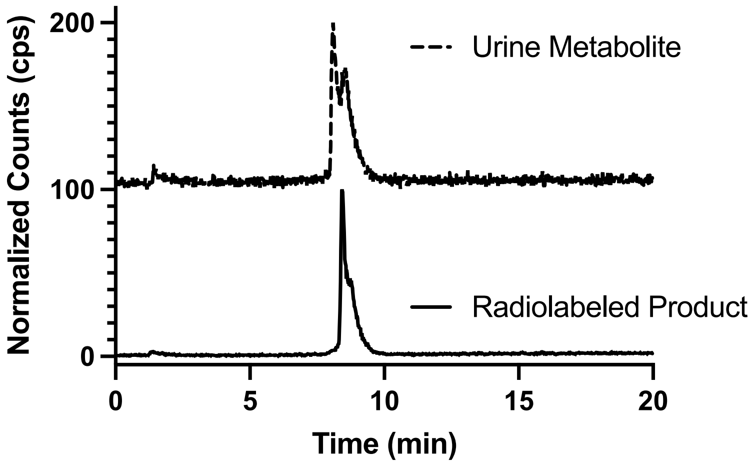

Figure S56. Comparative chromatographic analysis of **[^{86}Y]Y-pepa-pic$_2$-C-Hex-KuE** in the original radiolabeled product, and urine metabolite products found *ex vivo*.

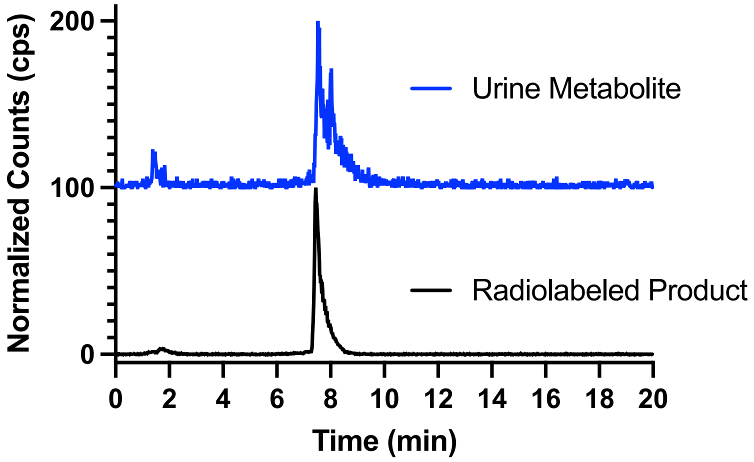

Figure S57. Comparative chromatographic analysis of **[^{177}Lu]Lu-pepa-pic$_2$-C-Hex-KuE** in the original radiolabeled product, the formulation of tracer in 1X DPBS and urine metabolite products found *ex vivo*.

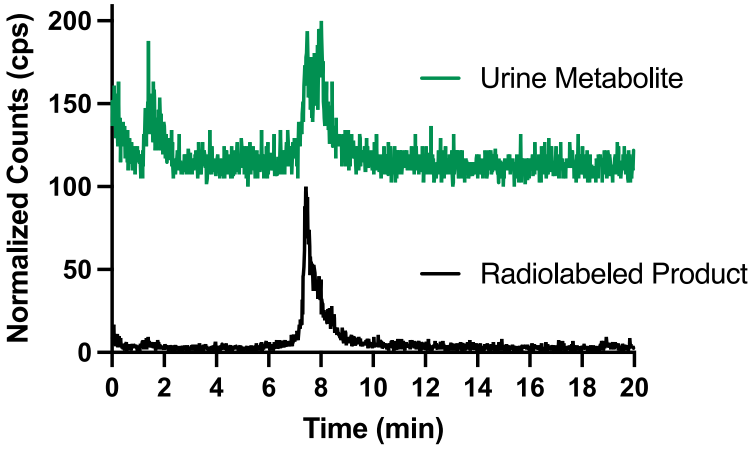

Figure S58. Comparative chromatographic analysis of **[^{161}Tb]Tb-pepa-pic$_2$-C-Hex-KuE** in the original radiolabeled product, the formulation of tracer in 1X DPBS and urine metabolite products found *ex vivo*.

5.4 In vivo CRET optical imaging

PSMA+/- tumors were implanted into male, nude, athymic mice ten days preceding the imaging study, as previously described in section 5.3.

5.4.1 Intratumoral Administration

A solution of 100 µL of [^{68}Ga]Ga-PSMA-617 (290 µCi) in DPBS buffer was administered via tail vein injection into a nude mouse bearing PSMA+ (right) and PSMA- (left) tumors. PET-CT images were collected 30 min post-injection of the radiotracer, followed by administration of ^{nat}Eu-pepa-pic$_2$-C-Hex-KuE (20 nmol, in xx) into the PSMA+ tumor 57 min post injection of the ^{68}Ga-tracer. Optical luminescence images were collected in an IVIS Spectrum Imaging System (Perkin Elmer) according to the parameters described below:

Photographic image:

Pixel Width/Height: 1; Binning Factor: 4; Image Units: counts; *f* Number: 8; Field of View: 13.3; Filter Position: 1; Emission filter: Open; Exposure Time Sec: 0.2; Subject size: 1.5.

Luminescent image:

Pixel Width/Height: 1; Binning Factor: 8; Luminescent Exposure (Seconds):300; *f* Number: 1; Field of View: 13.3; Emission filter: 620; Filter Position: 9; Excitation filter: Block; Subject size: 1.5; Subject Type: Mouse

Following the completion of the full body luminescence scan, the mouse was euthanized (90 min post injection of ^{68}Ga-radiotracer), and optical imaging scans of the resected tumors were collected. Subsequently, the remaining organs were harvested, their intestinal contents removed, and their radioactivity measured with a Hidex Automatic Gamma Counter. The measured counts per minute (cpm) were decay-corrected to the injection time and converted to curies (Ci) using the Gamma Counter's efficiency for Ga-68. The activity in each organ was expressed as the percentage of the injected dose per gram of organ weight (% ID/g).

Table S4. Region of interest (ROI) quantitative analysis of *in vivo* luminescence observed in mice bearing PSMA +/- tumors with **^{nat}Eu-pepa-pic$_2$-C-Hex-KuE** administered intratumorally.

Organs	Mouse with ^{nat}Eu-probe			Control Mouse (No ^{nat}Eu-probe administered)		
	Total flux (p/s)	Avg Radiance (p/s/cm^{-1}/sr)	Stdev Radiance	Total flux (p/s)	Avg Radiance (p/s/cm^{-1}/sr)	Stdev Radiance
Tumor -	4.60E+04	2.84E+03	7.05E+02	4.36E+04	2.68E+03	7.87E+02
Tumor +	2.01E+05	6.55E+03	3.64E+03	1.19E+05	3.84E+03	2.03E+03
R. Kidney	7.20E+05	2.25E+04	1.69E+04	5.01E+05	1.57E+04	1.15E+04

A second mouse that only received the initial dose of [^{68}Ga]Ga-PSMA-617 (310 µCi) was included as a control reference for comparison purposes.

5.4.2 Systemic Administration

A solution of 100 µL of [^{68}Ga]Ga-PSMA-617 (259 µCi) and **^{nat}Eu-pepa-pic$_2$-C-Hex-KuE** (100 nmol) was prepared in DPBS 1X and DMSO <5% and administered into a nude mouse bearing PSMA+ (right) and PSMA- (left) tumors via-tail vein injection.

PET-CT images, and luminescence optical images of full body were collected as previously described for the intratumorally administration agents, at 30 min p.i- and 60-min p.i respectively. Mice were euthanized 90 min p.i, organs were harvested and optical imaging of resected tumors as well as biodistribution analysis was conducted.

Table S 5. Region of interest (ROI) quantitative analysis of in vivo luminescence observed in mice bearing PSMA +/- tumors with **^{nat}Eu-pepa-pic$_2$-C-Hex-KuE** administered systemically.

Organs	Mouse with ^{nat}Eu-probe			Control Mouse (No ^{nat}Eu-probe administered)		
	Total flux (p/s)	Avg Radiance (p/s/cm^{-1}/sr)	Stdev Radiance	Total flux (p/s)	Avg Radiance (p/s/cm^{-1}/sr)	Stdev Radiance
Tumor -	6.16E+04	2.56E+03	1.02E+03	4.80E+04	2.18E+03	8.33E+02
Tumor +	3.53E+05	7.77E+03	3.89E+03	2.18E+05	6.62E+03	4.56E+03
R. Kidney	2.62E+05	7.74E+03	4.30E+03	1.71E+05	5.18E+03	1.71E+02

5.5 Characterization data and spectroscopy of ligands

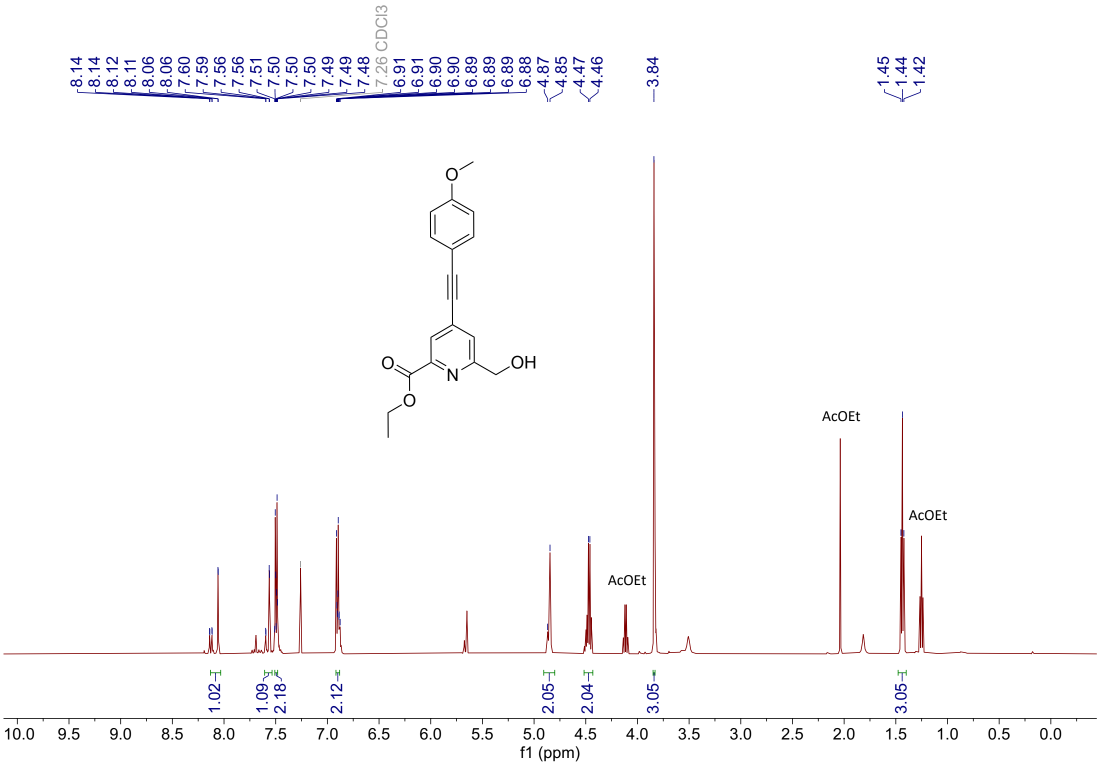

Figure S59. ^{1}H NMR spectrum of **2** (500 MHz, $CDCl_3$).

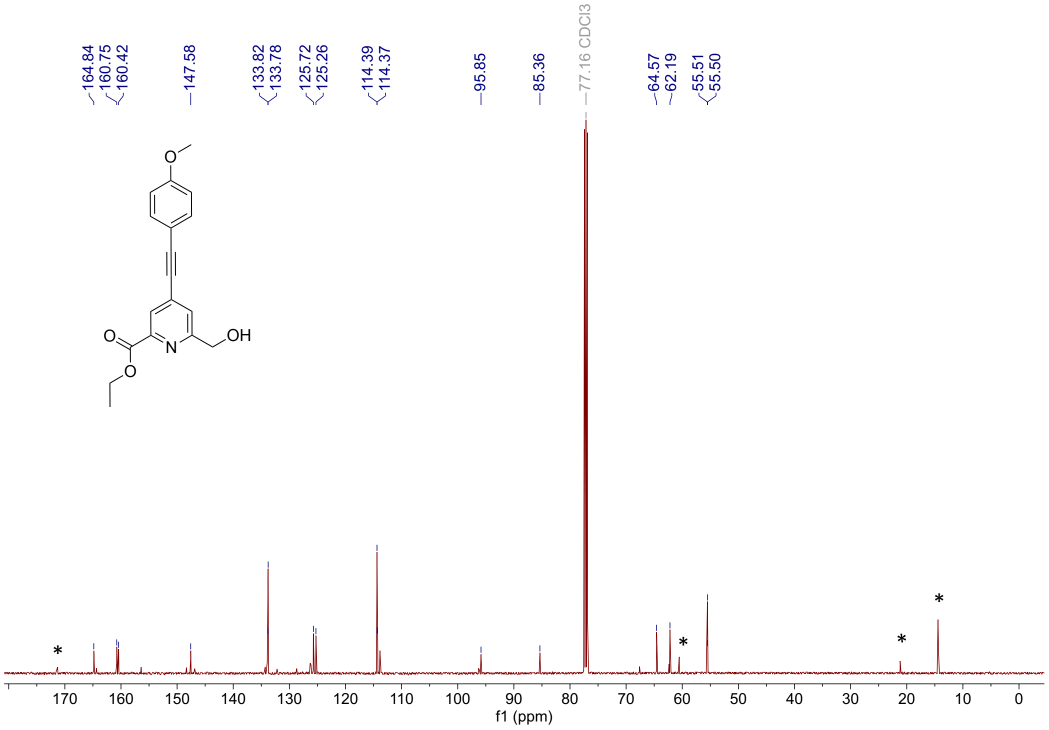

Figure S60. ^{13}C NMR spectrum of **2** (126 MHz, $CDCl_3$). *Trace Ethyl Acetate.

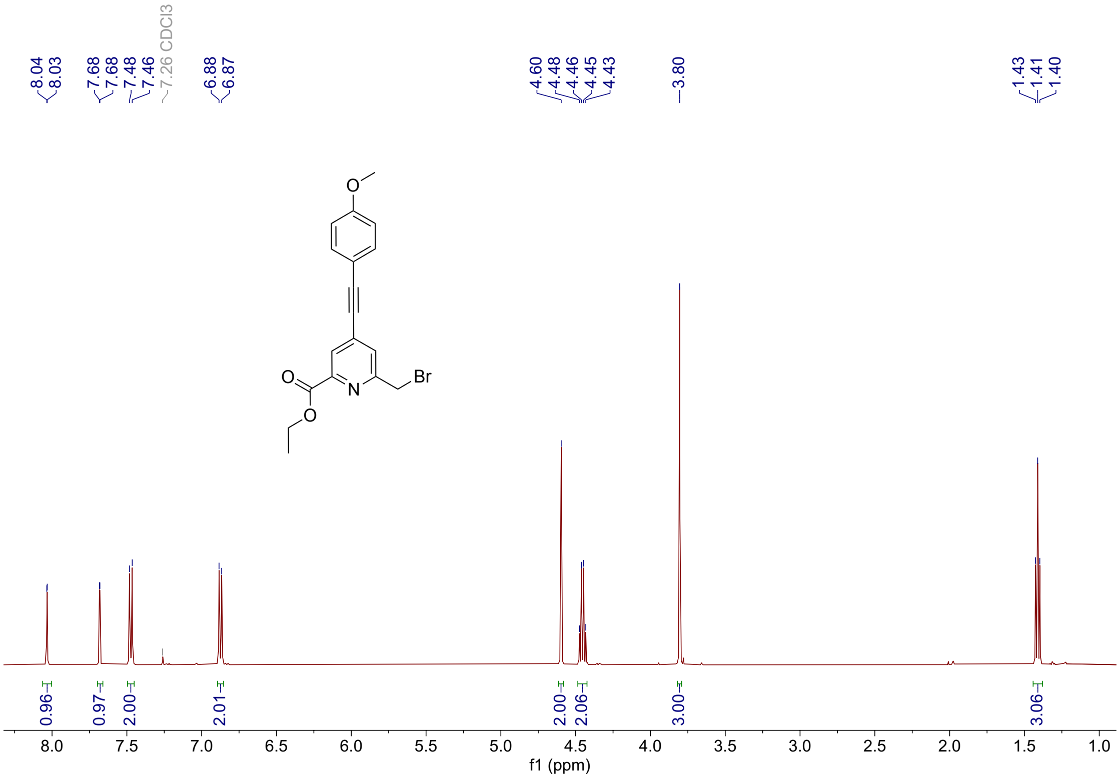

Figure S61. ^{1}H NMR spectrum of **3** (500 MHz, $CDCl_3$).

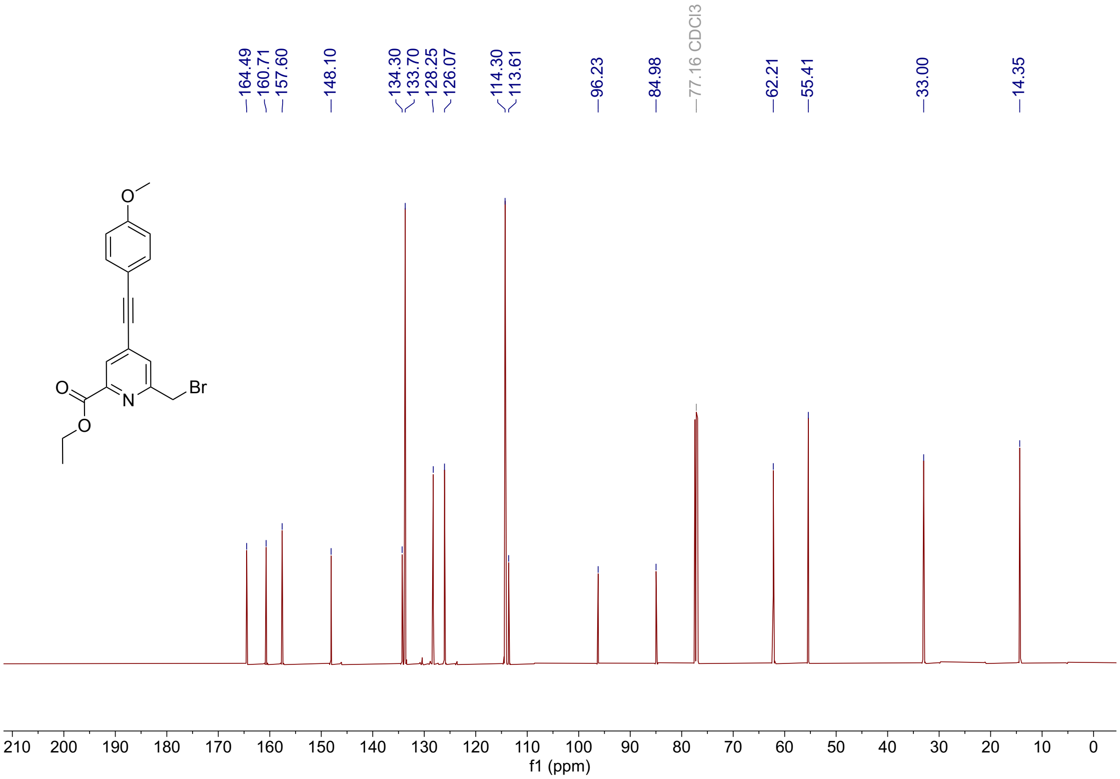

Figure S62. ^{13}C NMR spectrum of **3** (126 MHz, $CDCl_3$).

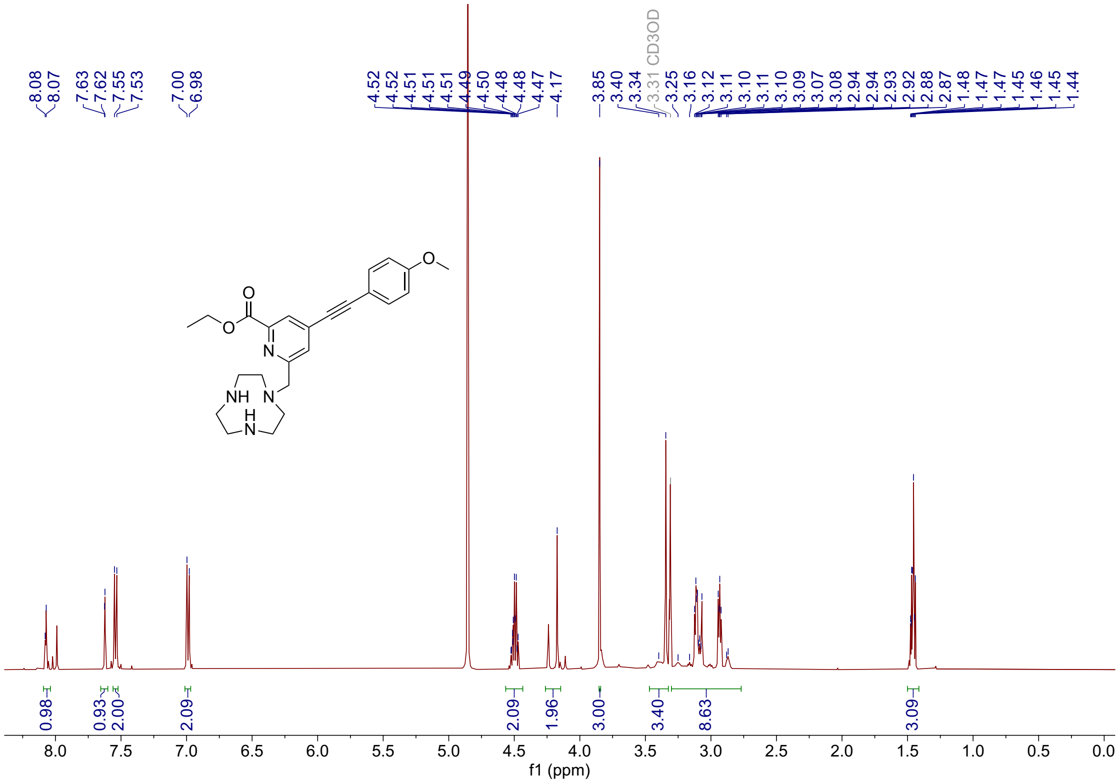

Figure S63. ^{1}H NMR spectrum of **4** (500 MHz, MeOD-d_4).

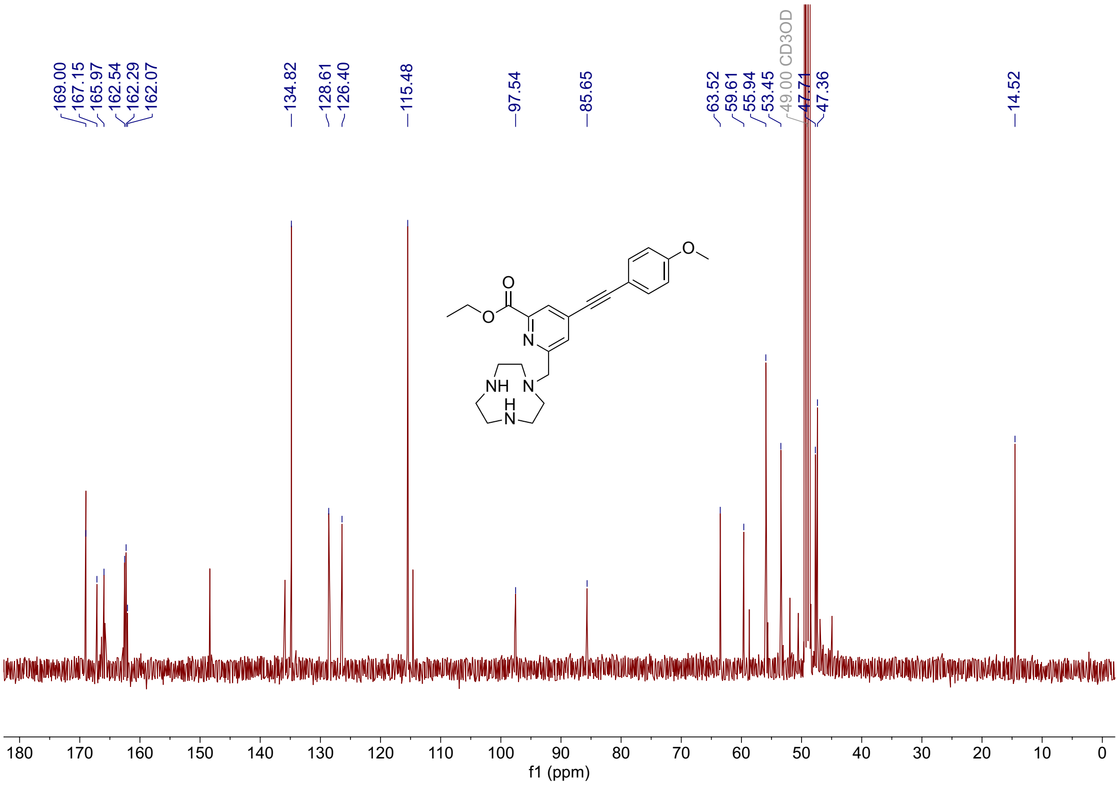

Figure S64. ^{13}C NMR spectrum of **4** (126 MHz, MeOD-d_4).

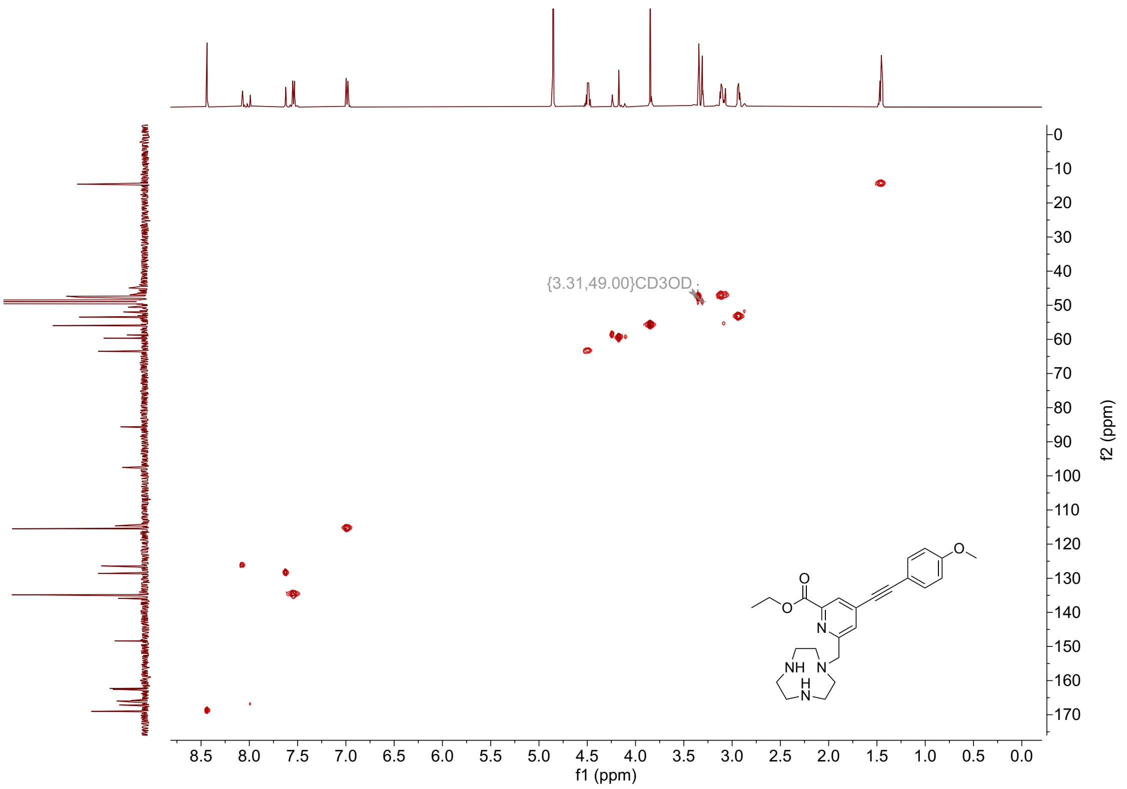

Figure S65. ^{1}H- ^{13}C HSQC NMR spectrum of **4** (500 MHz, MeOD-d_4).

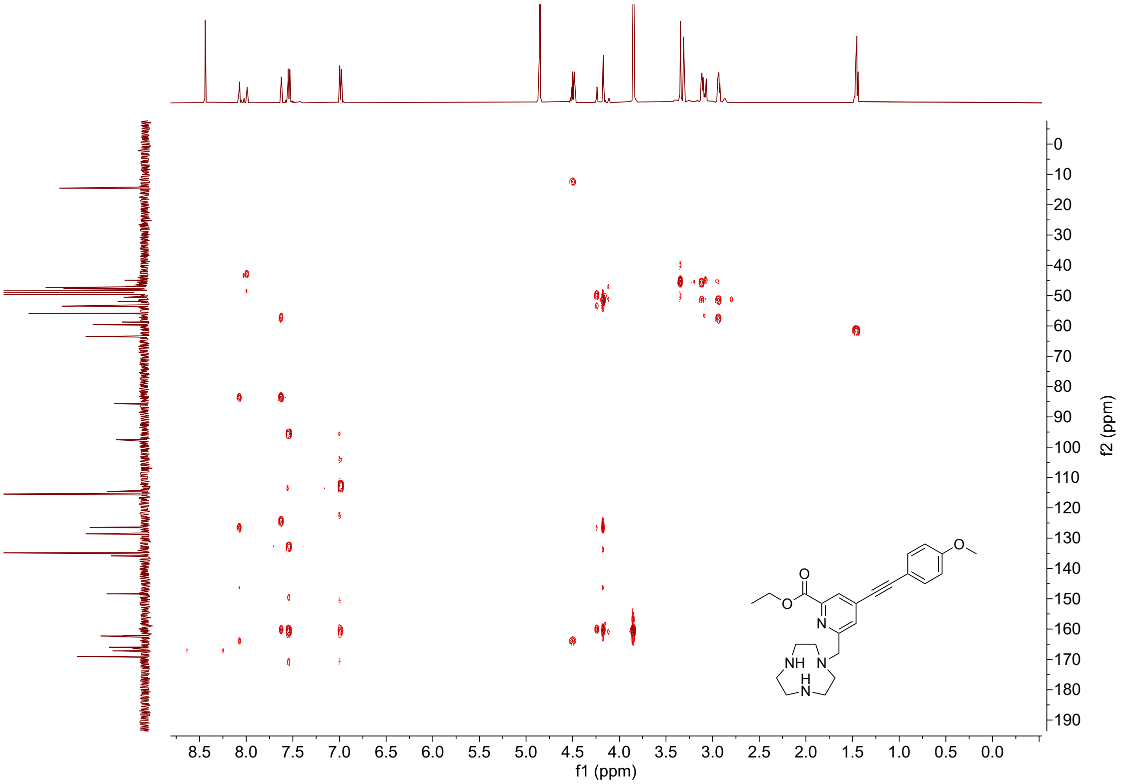

Figure S 66. ^{1}H- ^{13}C HMBC NMR spectrum of **4** (500 MHz, MeOD-d_4).

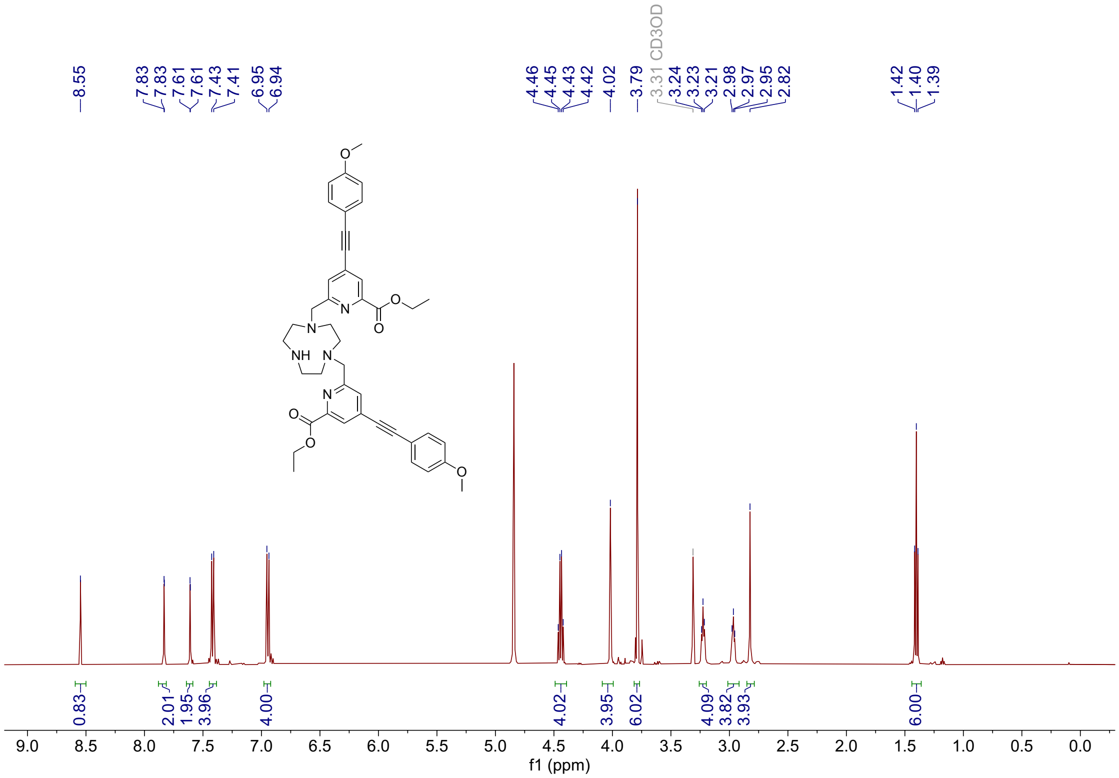

Figure S67. ^{1}H NMR spectrum of **5** (500 MHz, MeOD-d_4).

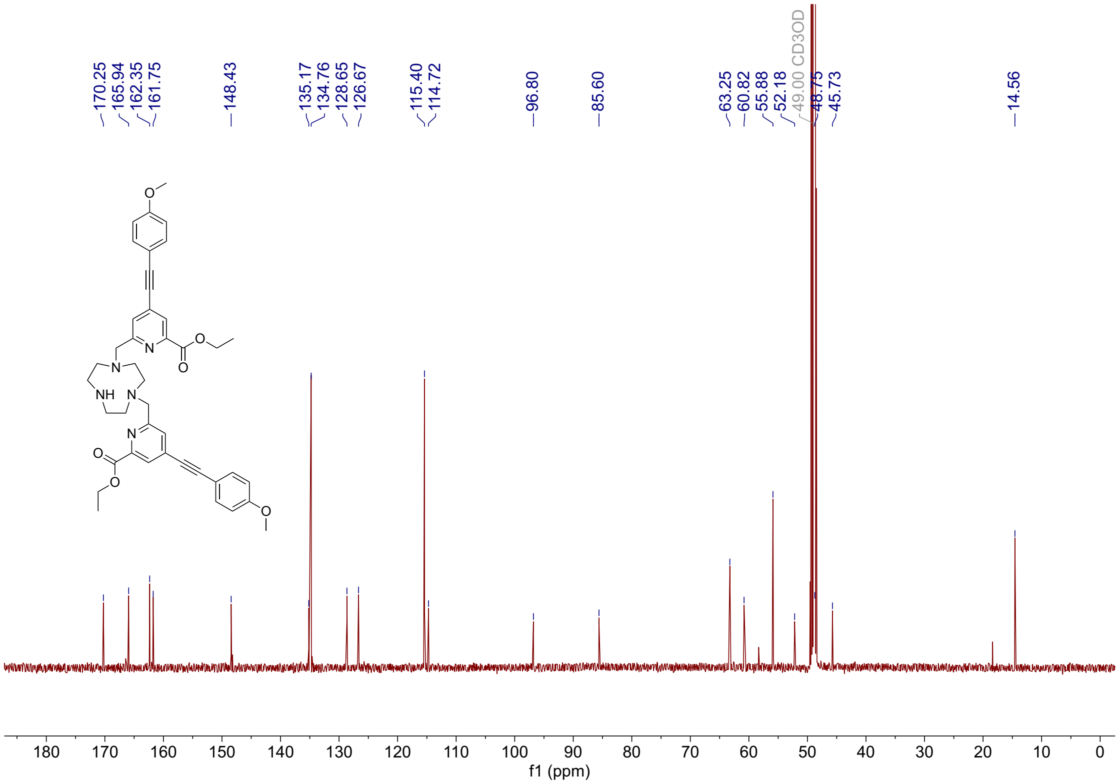

Figure S68. ^{13}C NMR spectrum of **5** (126 MHz, MeOD-d_4).

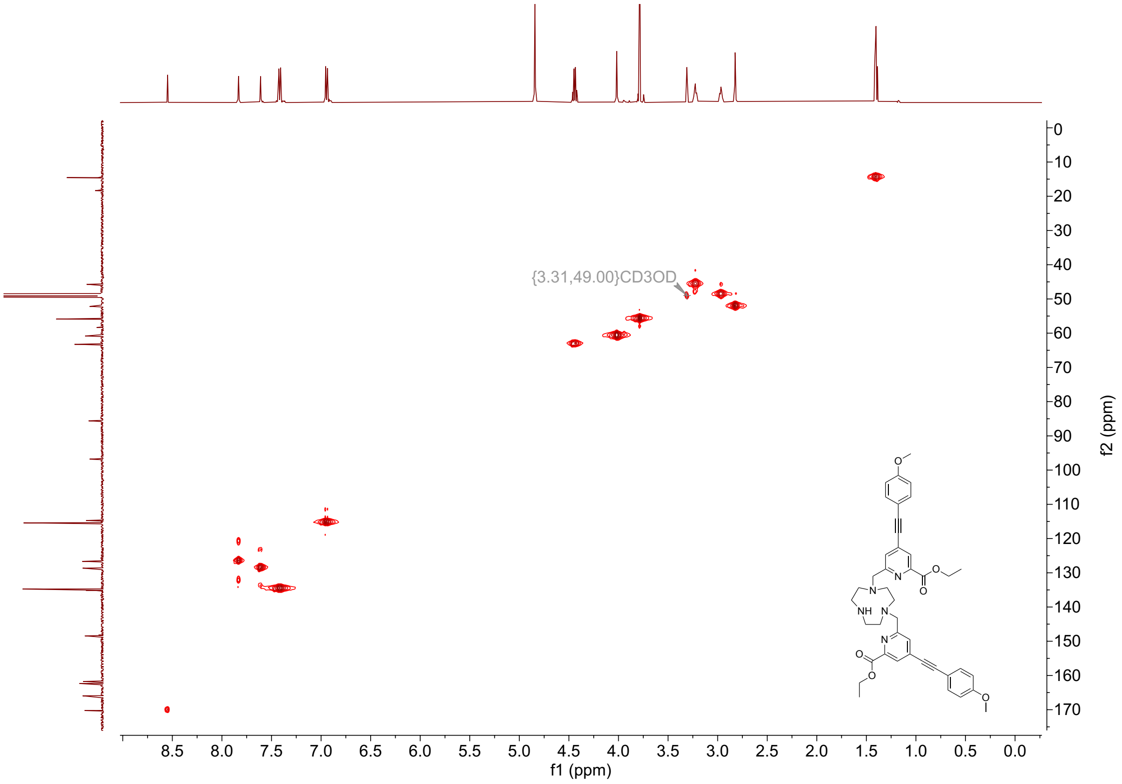

Figure S69. ^{1}H- ^{13}C HSQC NMR of **5** (500 MHz, MeOD-d_4).

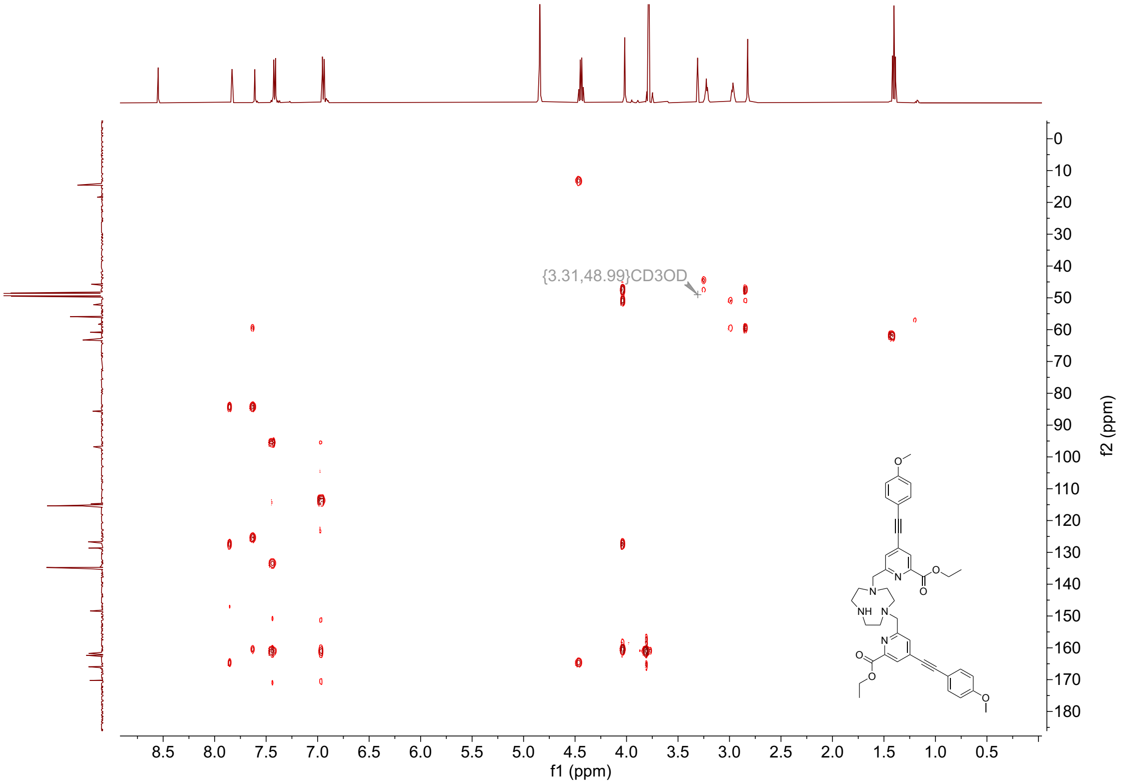

Figure S70. ^{1}H- ^{13}C HMBC NMR spectrum of **5** (500 MHz, MeOD-d_4).

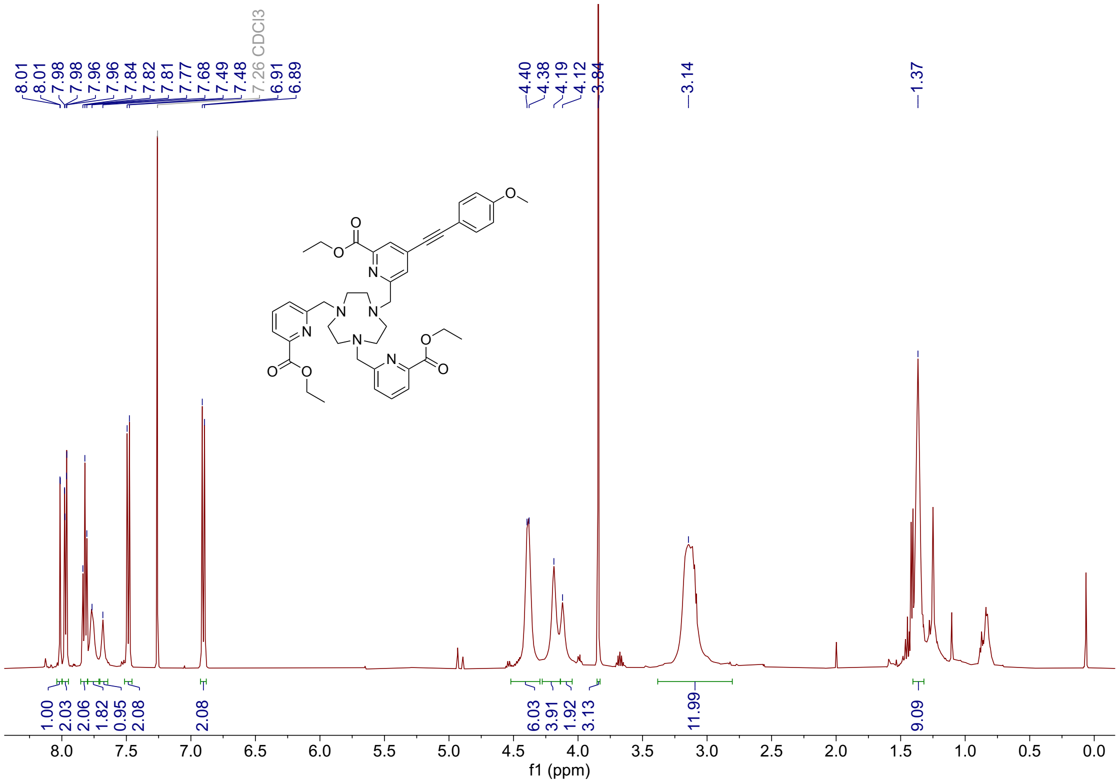

Figure S71. ^{1}H NMR spectrum of **6** (500 MHz, $CDCl_3$).

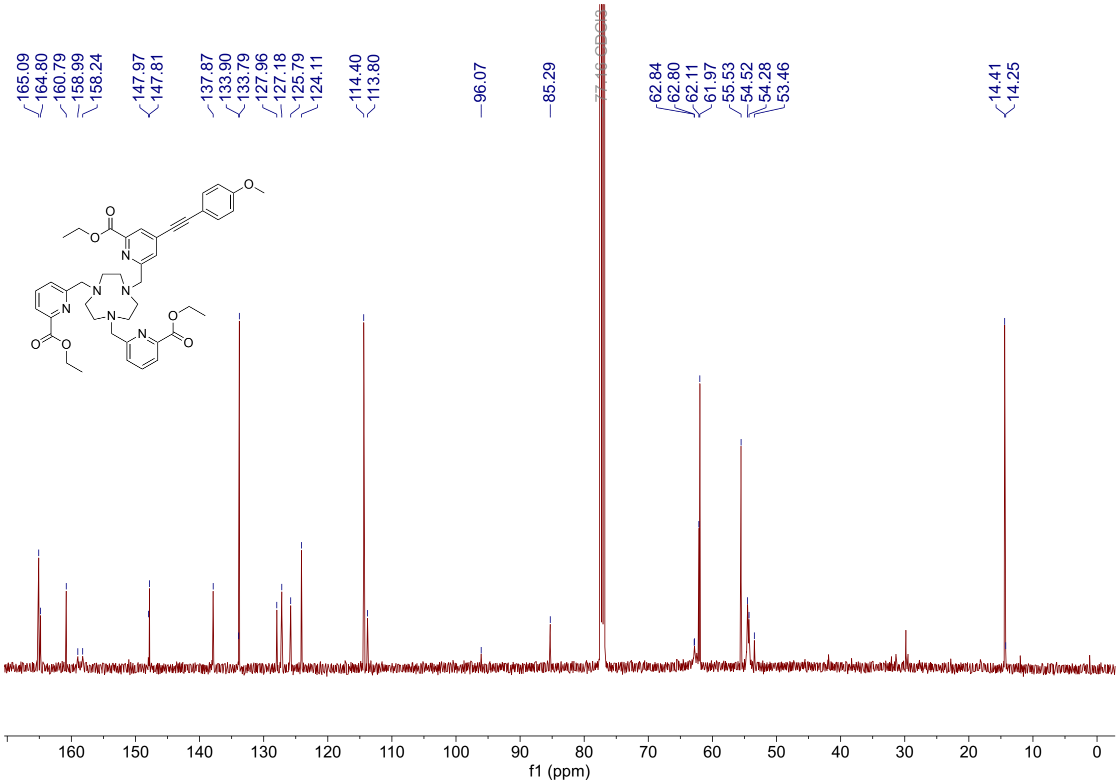

Figure S72. ^{13}C NMR spectrum of **6** (126 MHz, $CDCl_3$).

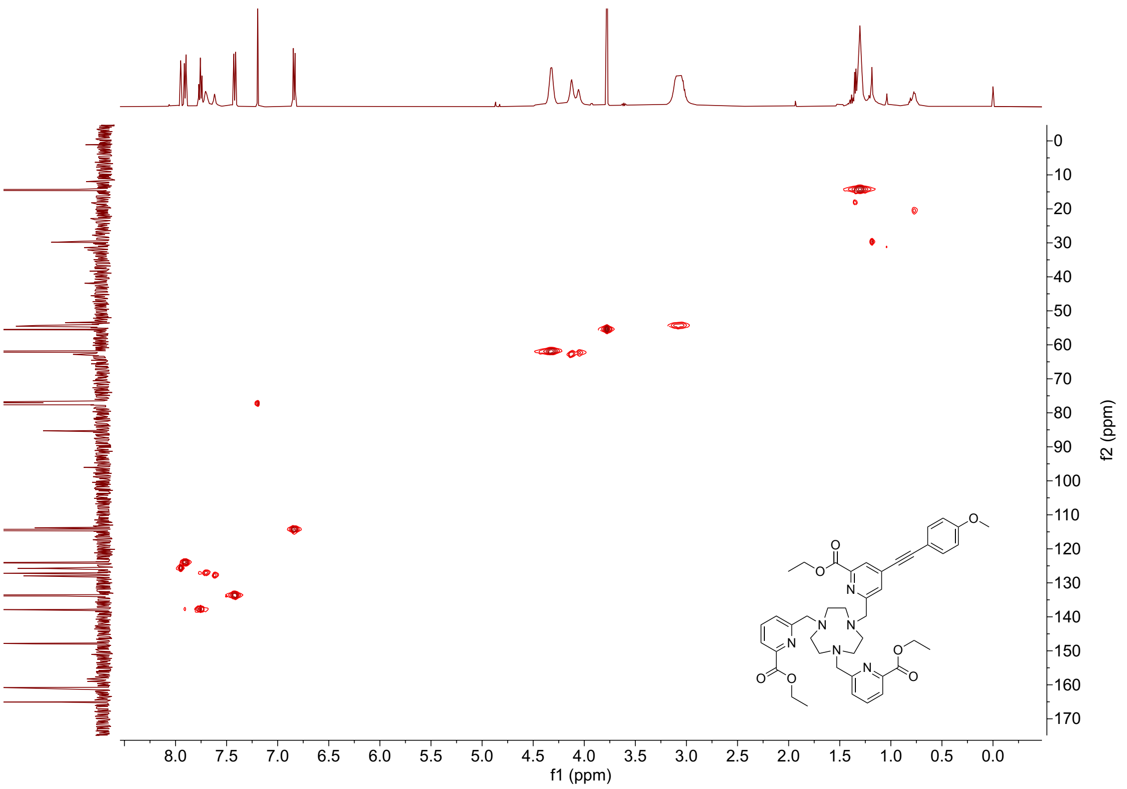

Figure S73. ^{1}H-^{13}C HSQC NMR spectrum of **6** (500 MHz, $CDCl_3$).

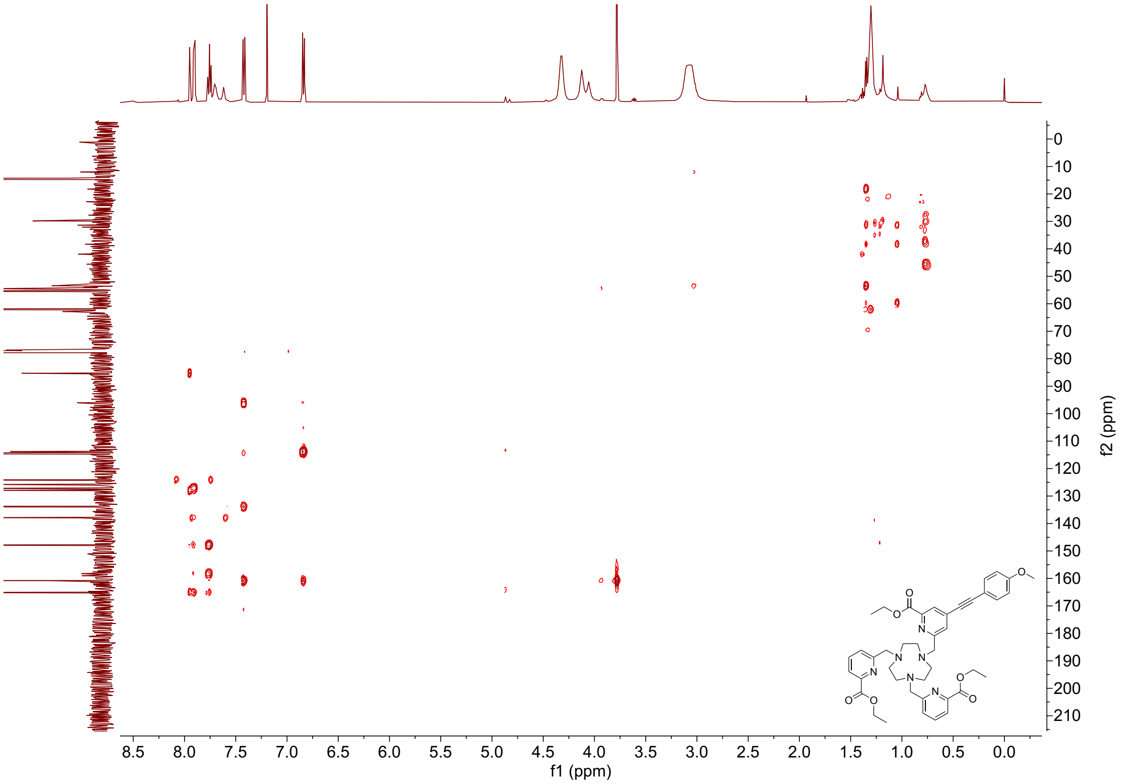

Figure S74. ^{1}H-^{13}C HMBC NMR spectrum of **6** (500 MHz, $CDCl_3$).

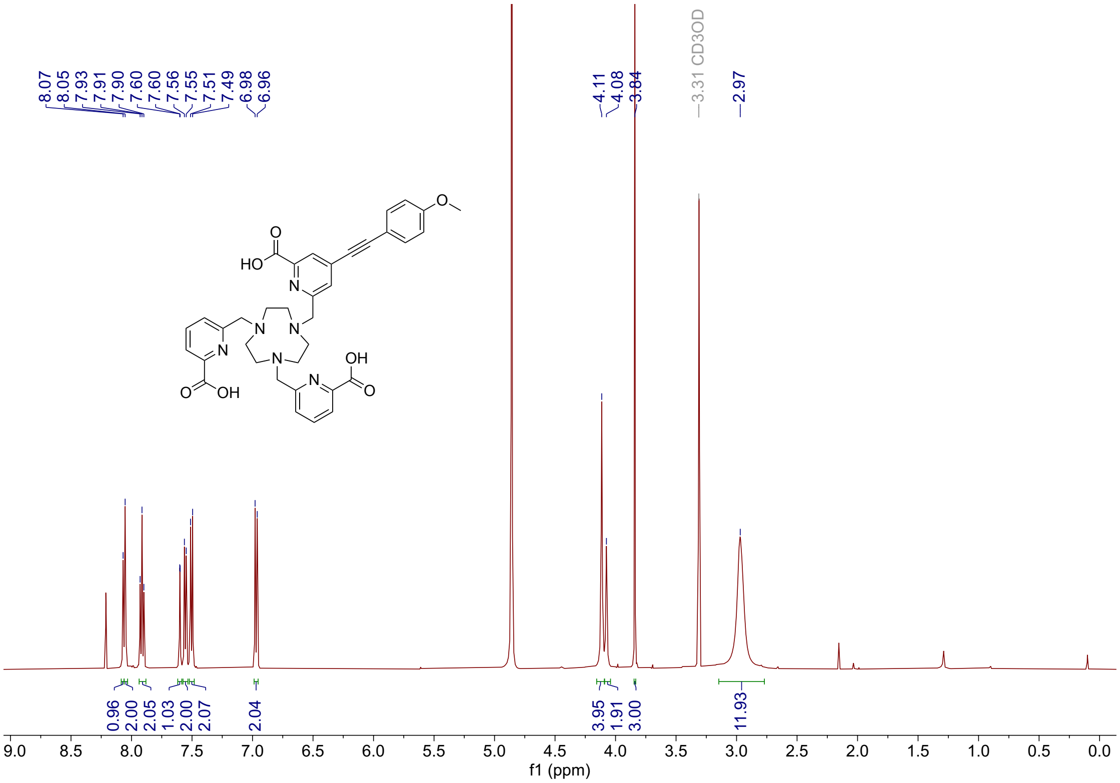

Figure S75. ^{1}H NMR spectrum of **mepa-pic$_2$** (500 MHz, MeOD-d_4).

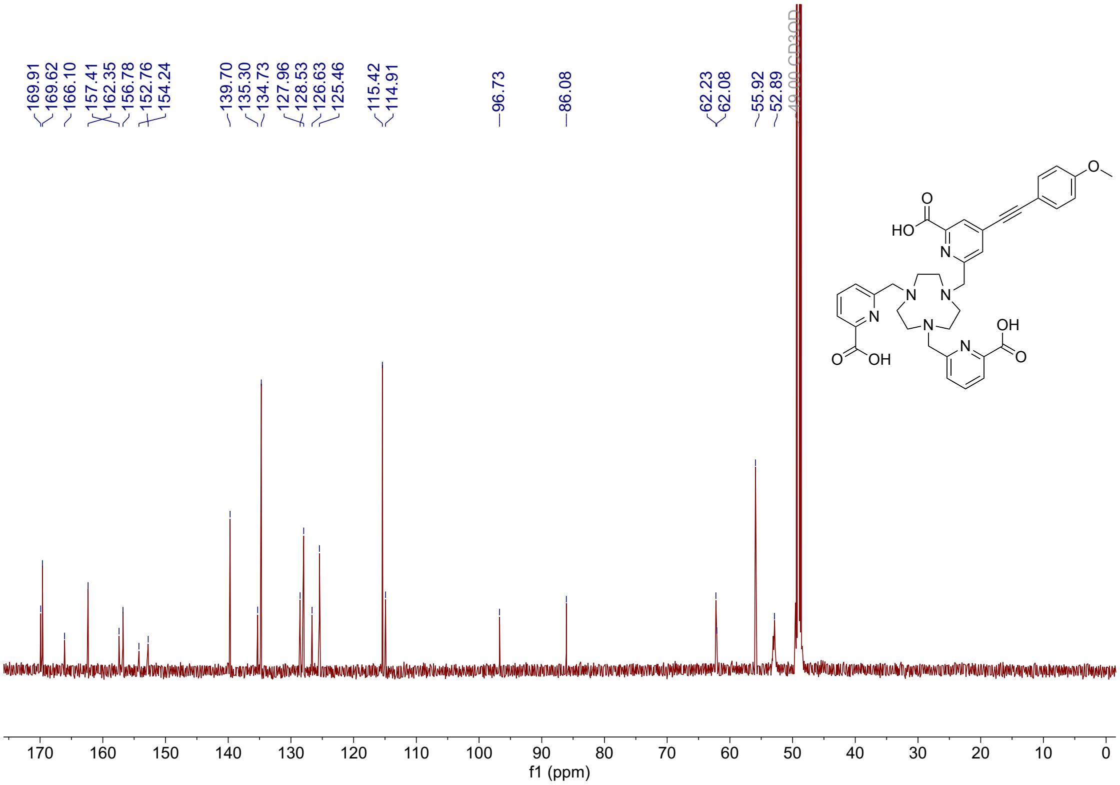

Figure S76. ^{13}C NMR spectrum of **mepa-pic$_2$** (126 MHz, MeOD-d_4).

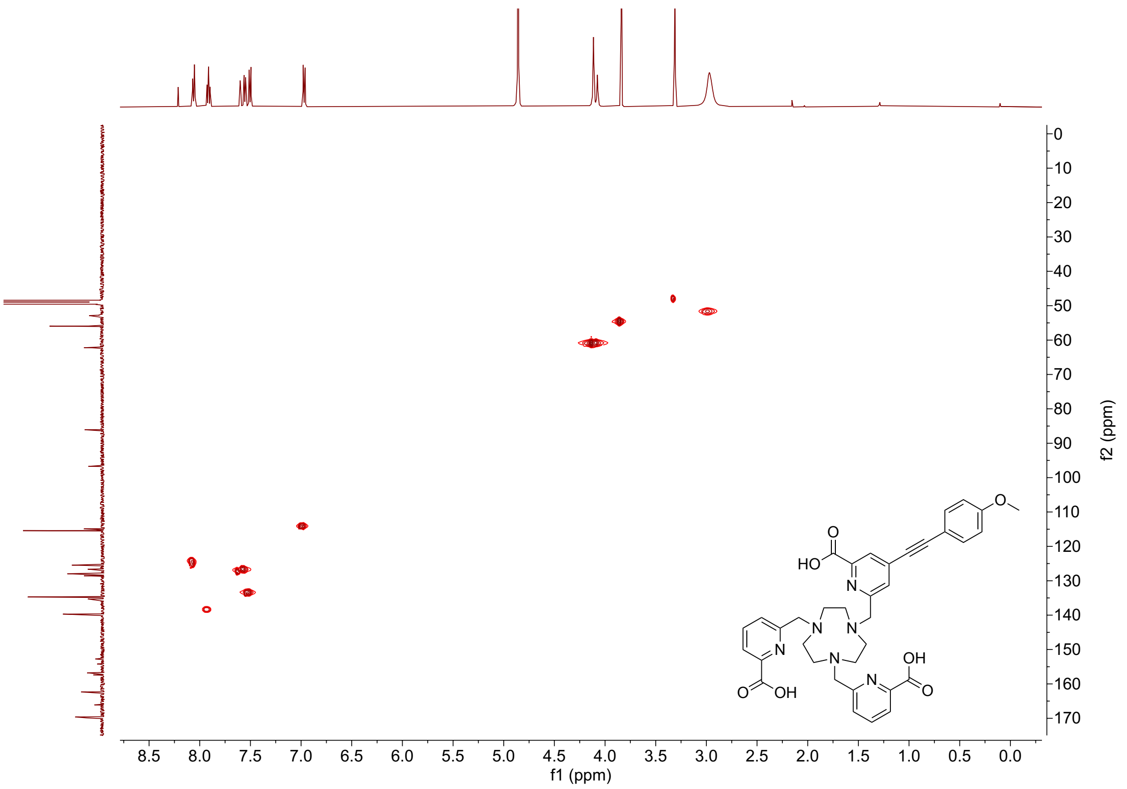

Figure S77. ^{1}H-^{13}C HSQC NMR spectrum of **mepa-pic$_2$** (500 MHz, MeOD-d_4).

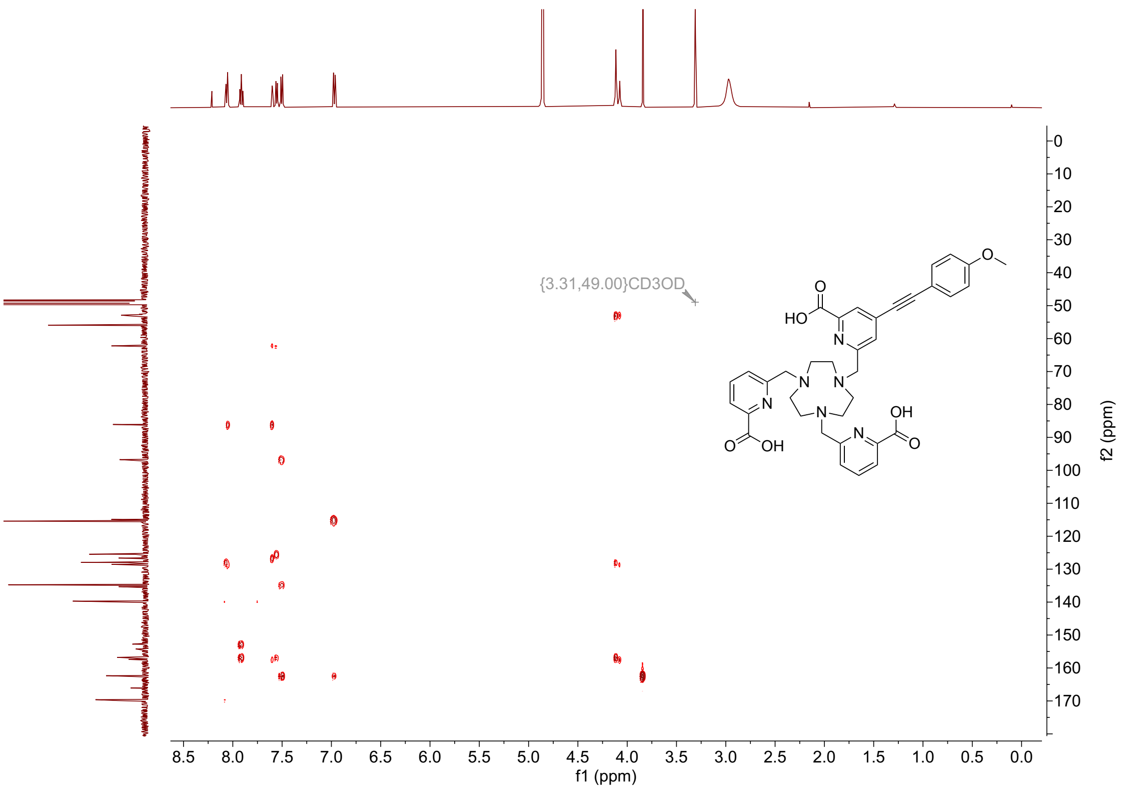

Figure S78. ^{1}H-^{13}C HMBC NMR spectrum of **mepa-pic$_2$** (500 MHz, MeOD-d_4).

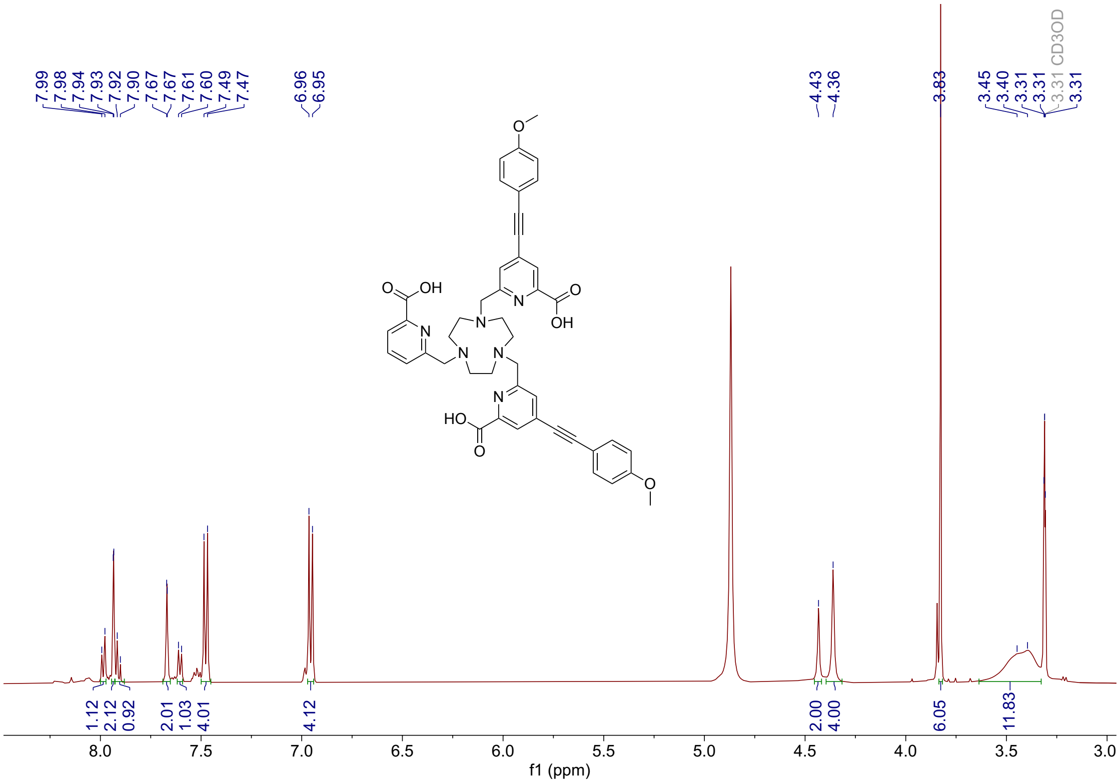

Figure S79. ^{1}H NMR spectrum of **mepa$_2$-pic** (500 MHz, MeOD-d_4).

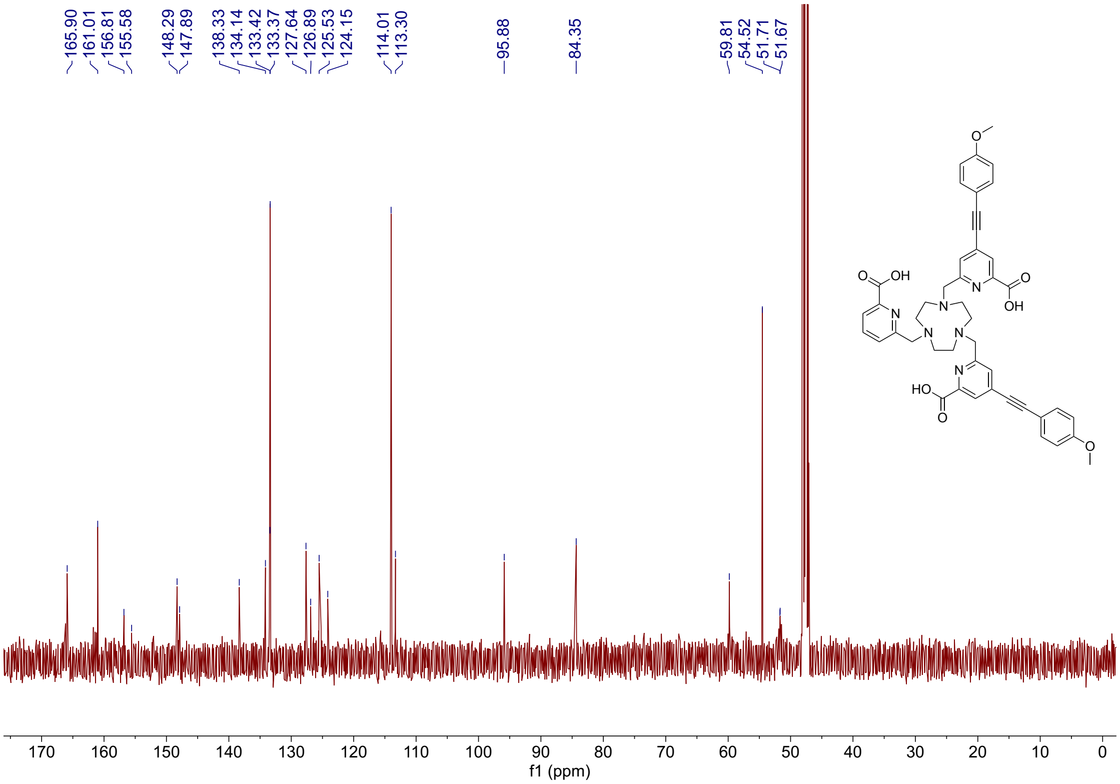

Figure S80. ^{13}C NMR spectrum of **mepa$_2$-pic** (126 MHz, MeOD-d_4).

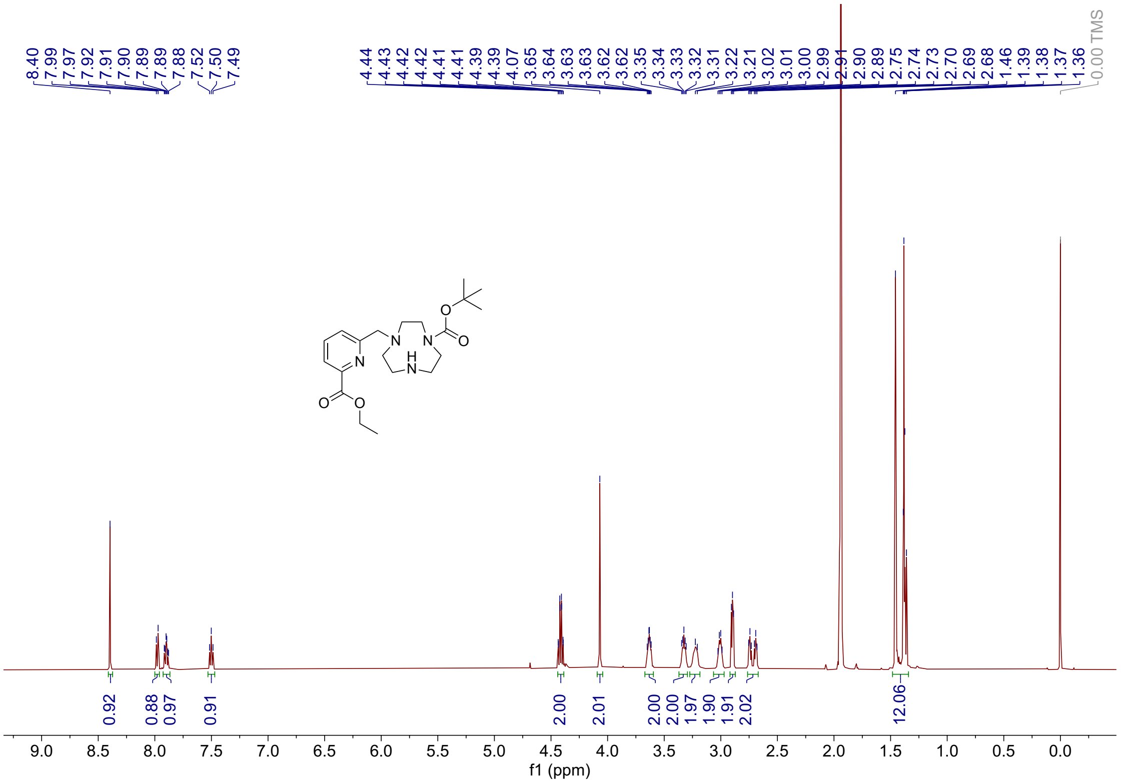

Figure S81. ^{1}H NMR spectrum of **7** (500 MHz, CD_3CN).

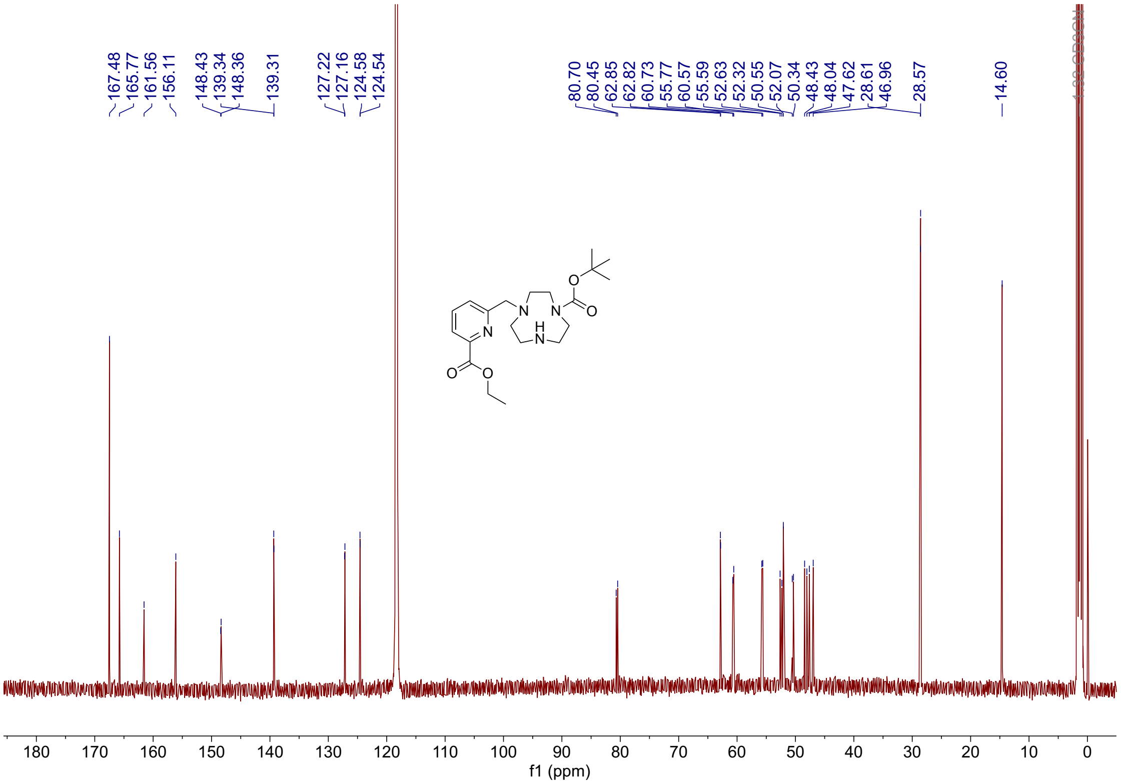

Figure S82. ^{13}C NMR spectrum of **7** (126 MHz, CD_3CN).

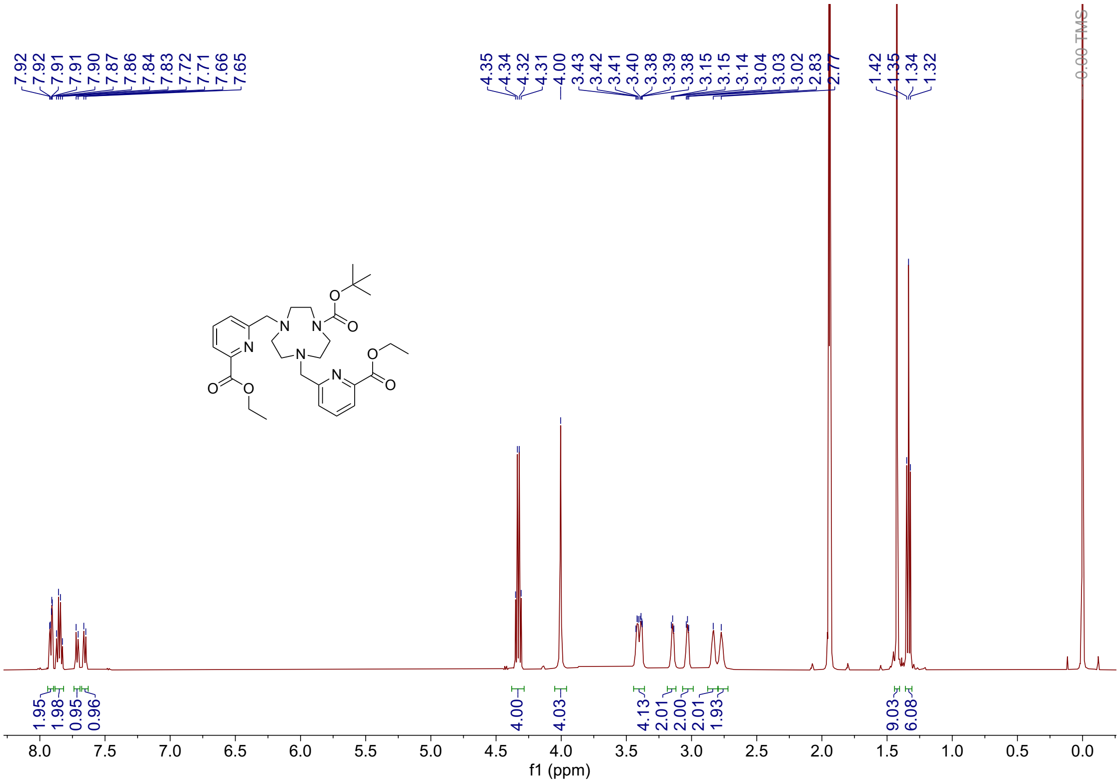

Figure S83. ^{1}H NMR spectrum of **8** (500 MHz, CD_3CN).

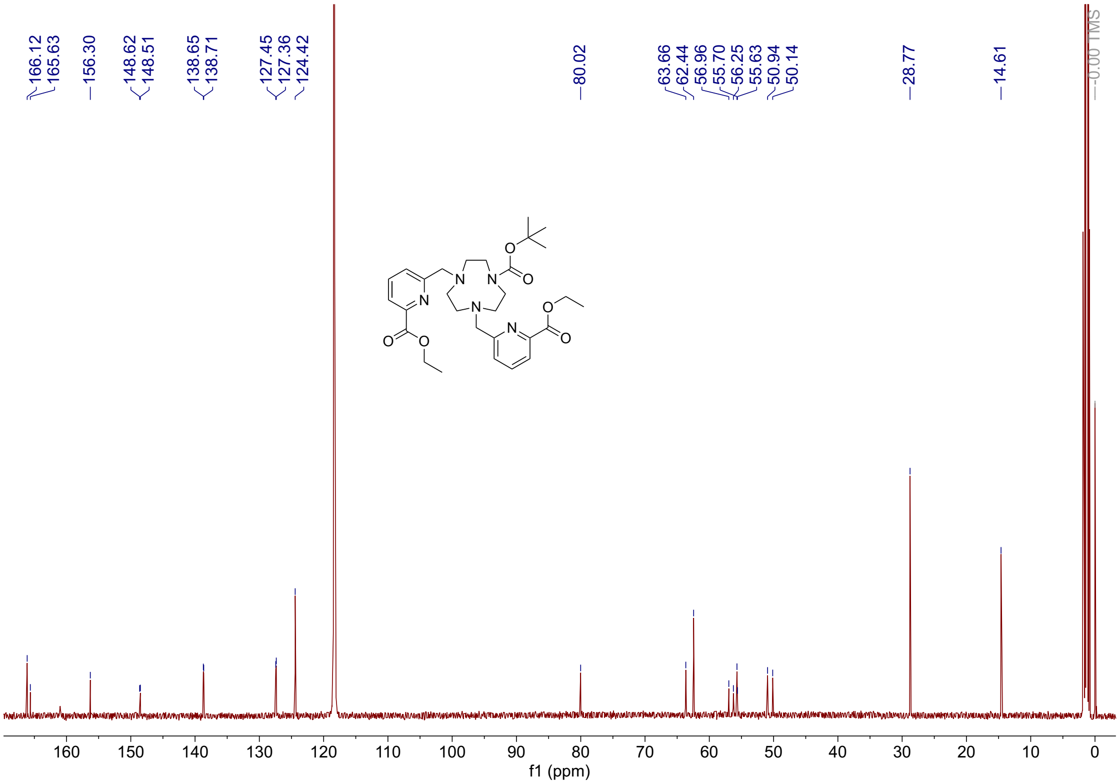

Figure S84. ^{13}C NMR spectrum of **8** (126 MHz, CD_3CN).

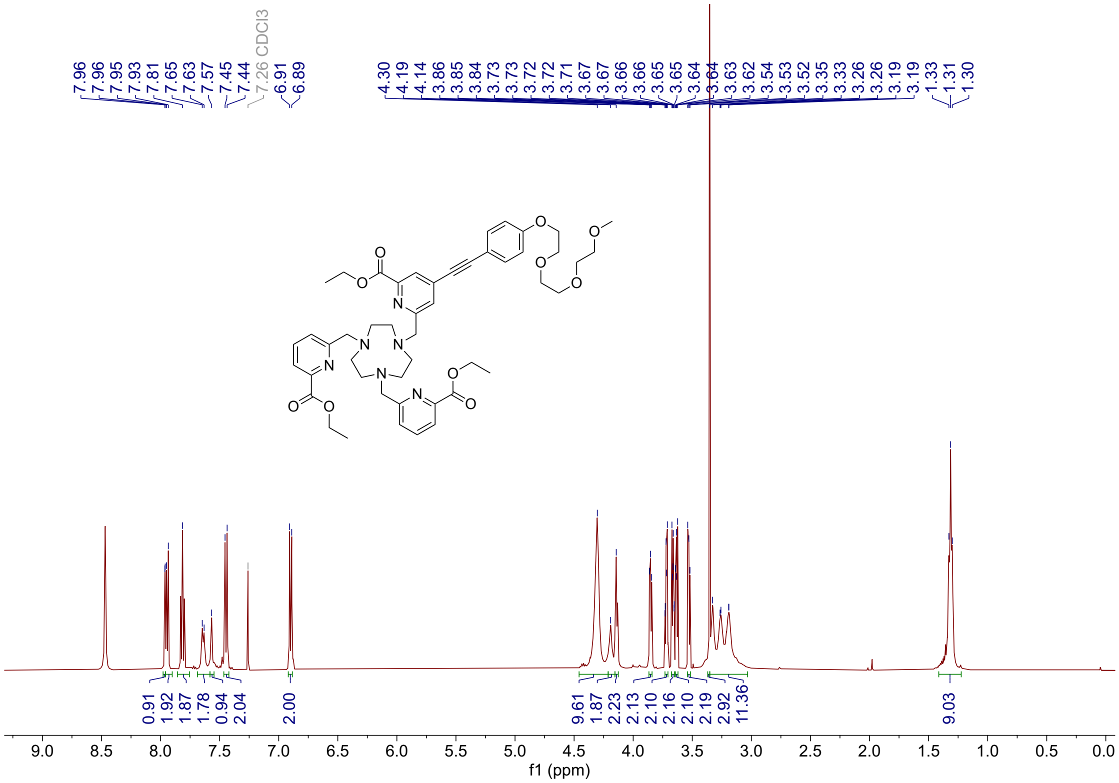

Figure S85. ^{1}H NMR spectrum of **9** (500 MHz, $CDCl_3$).

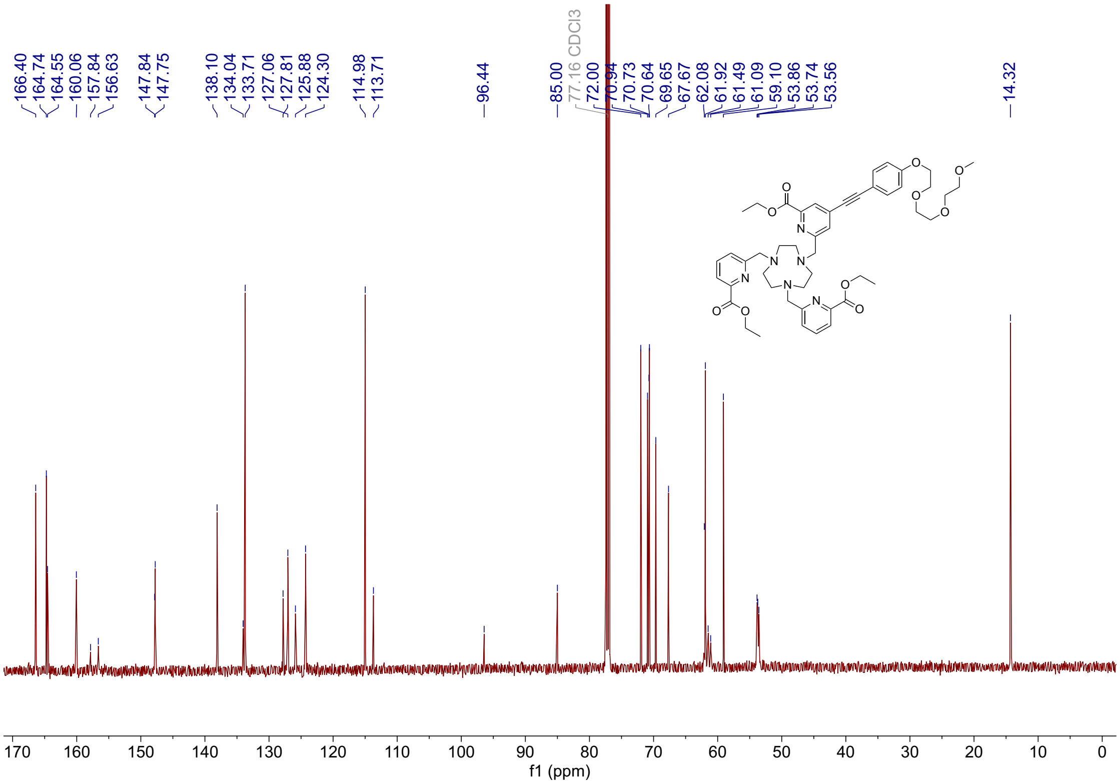

Figure S86. ^{13}C NMR spectrum of **9** (126 MHz, $CDCl_3$).

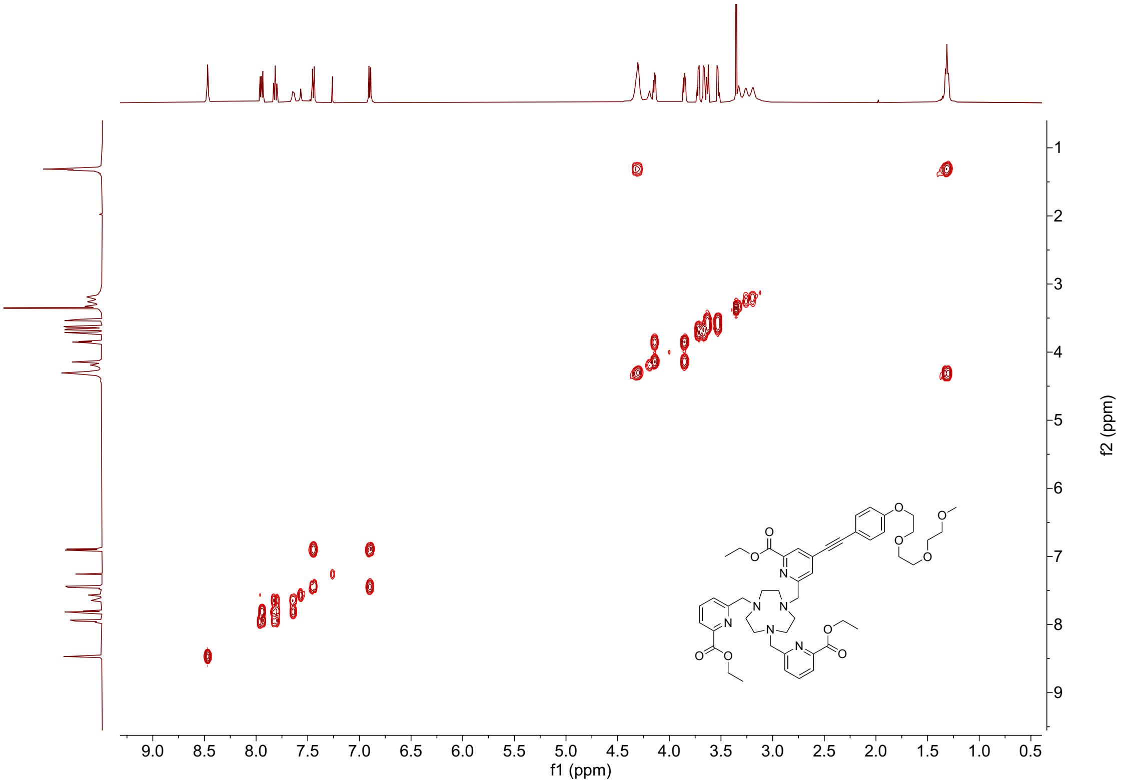

Figure S87. ^{1}H-^{1}H COSY NMR spectrum of **9** (500 MHz, $CDCl_3$).

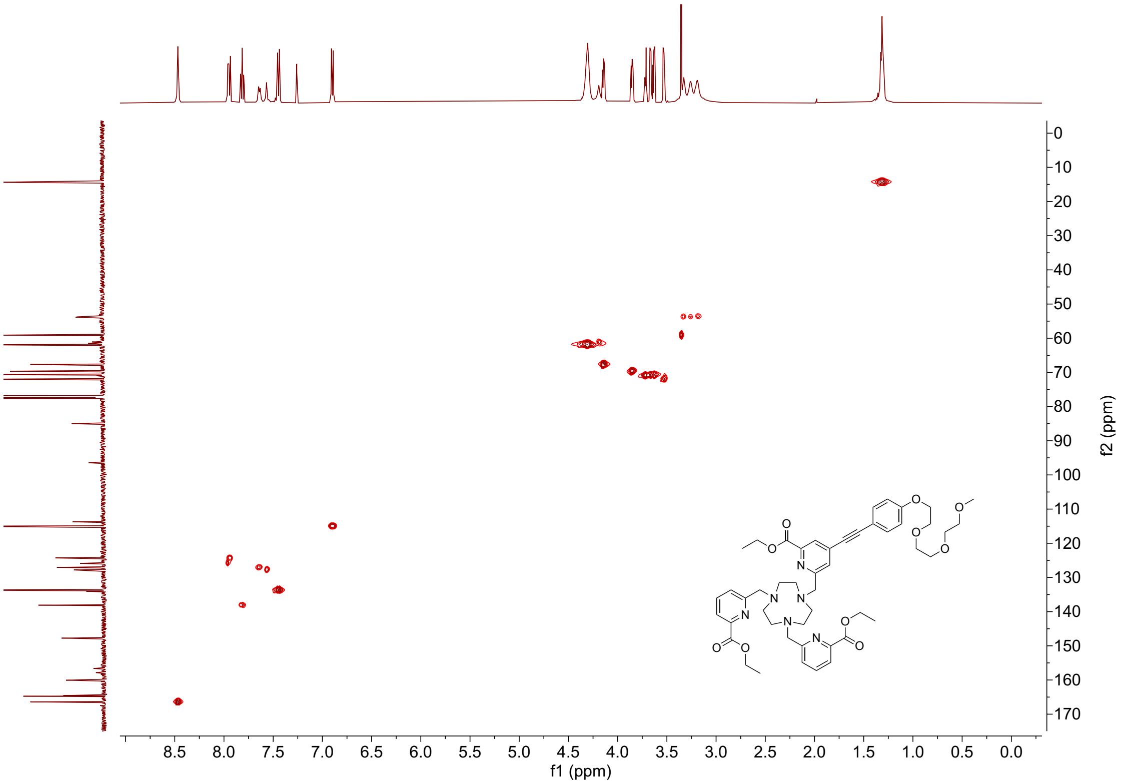

Figure S88. ^{1}H-^{13}C HSQC NMR spectrum of **9** (500 MHz, $CDCl_3$).

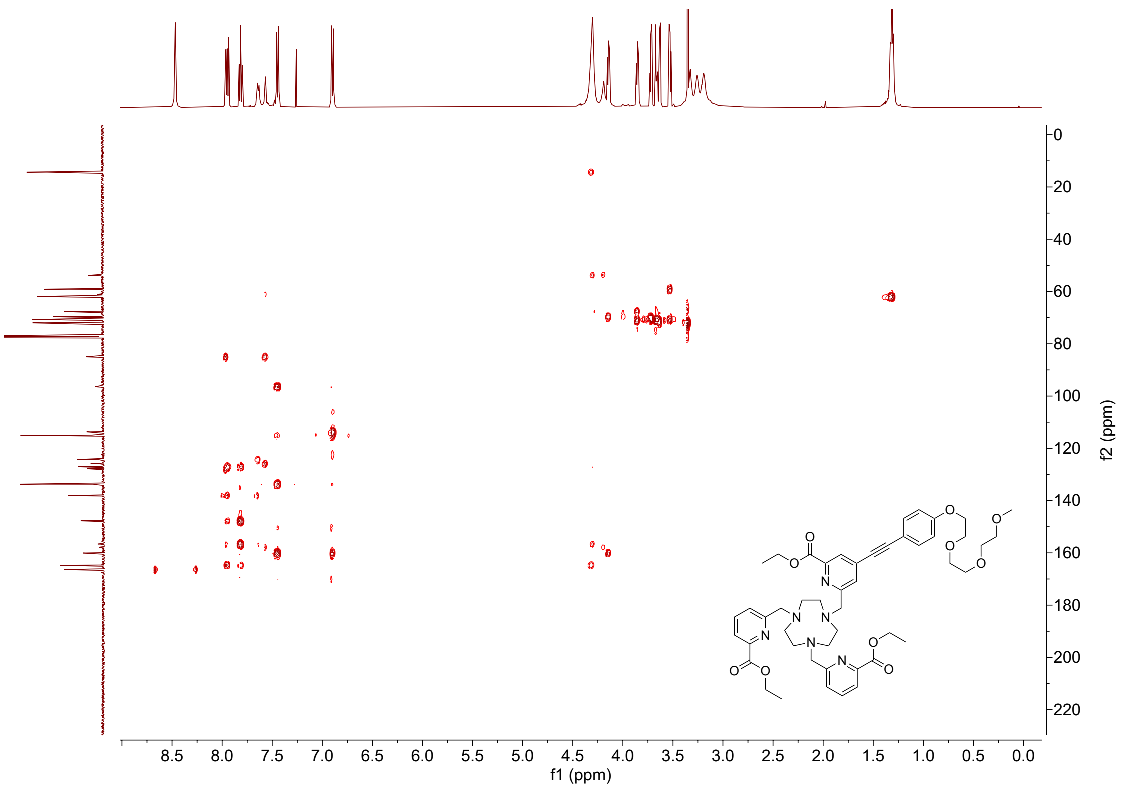

Figure S89. ^{1}H-^{13}C HMBC NMR spectrum of **9** (500 MHz, $CDCl_3$).

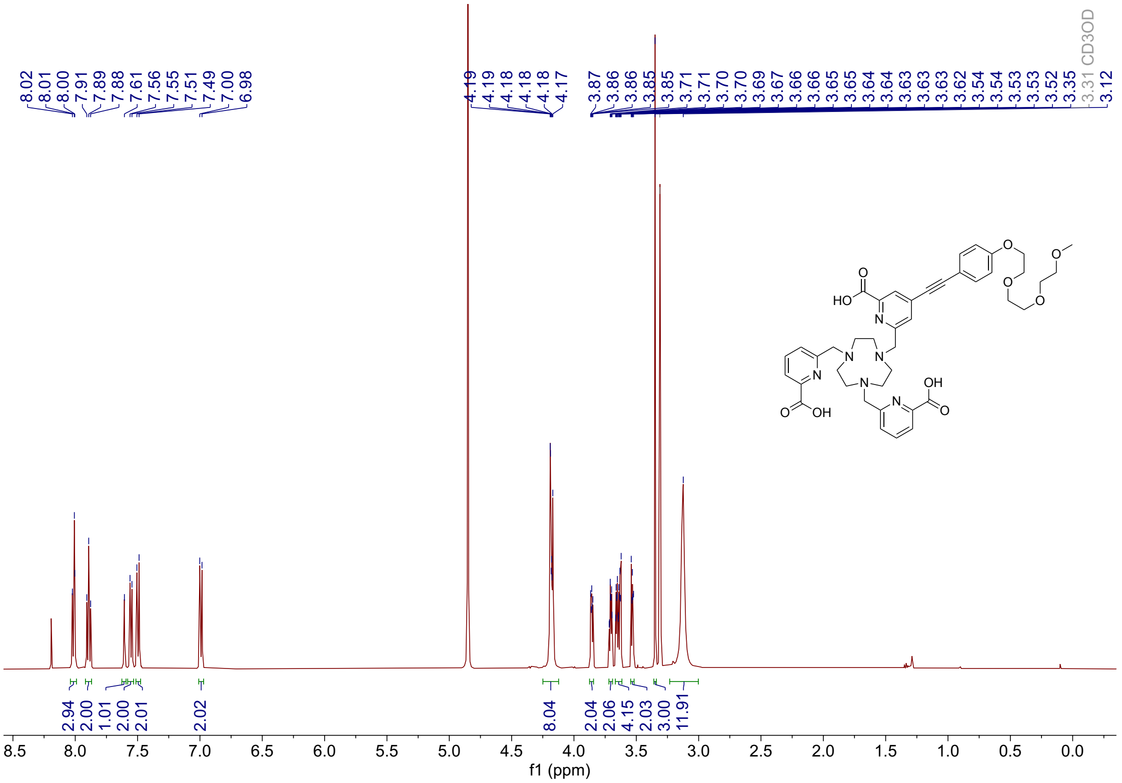

Figure S90. ^{1}H NMR spectrum of **pepa-pic$_2$** (500 MHz, MeOD-d_4).

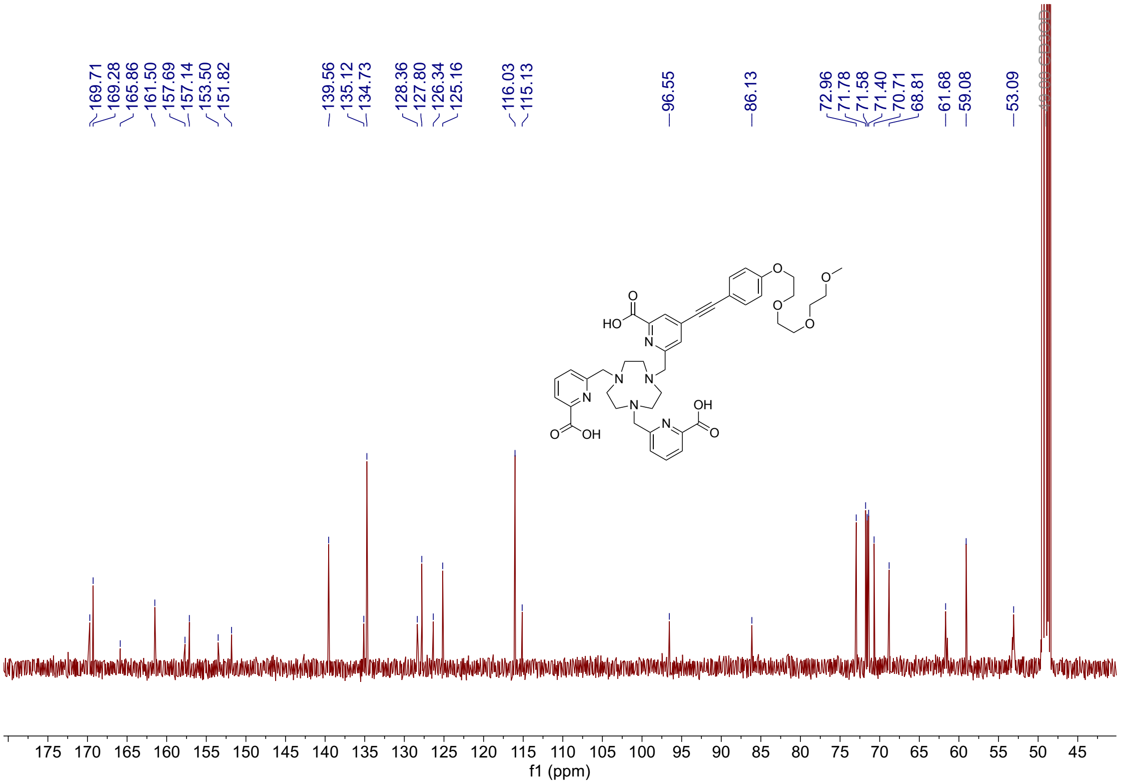

Figure S91. ^{13}C NMR spectrum of **pepa-pic$_2$** (126 MHz, MeOD-d_4).

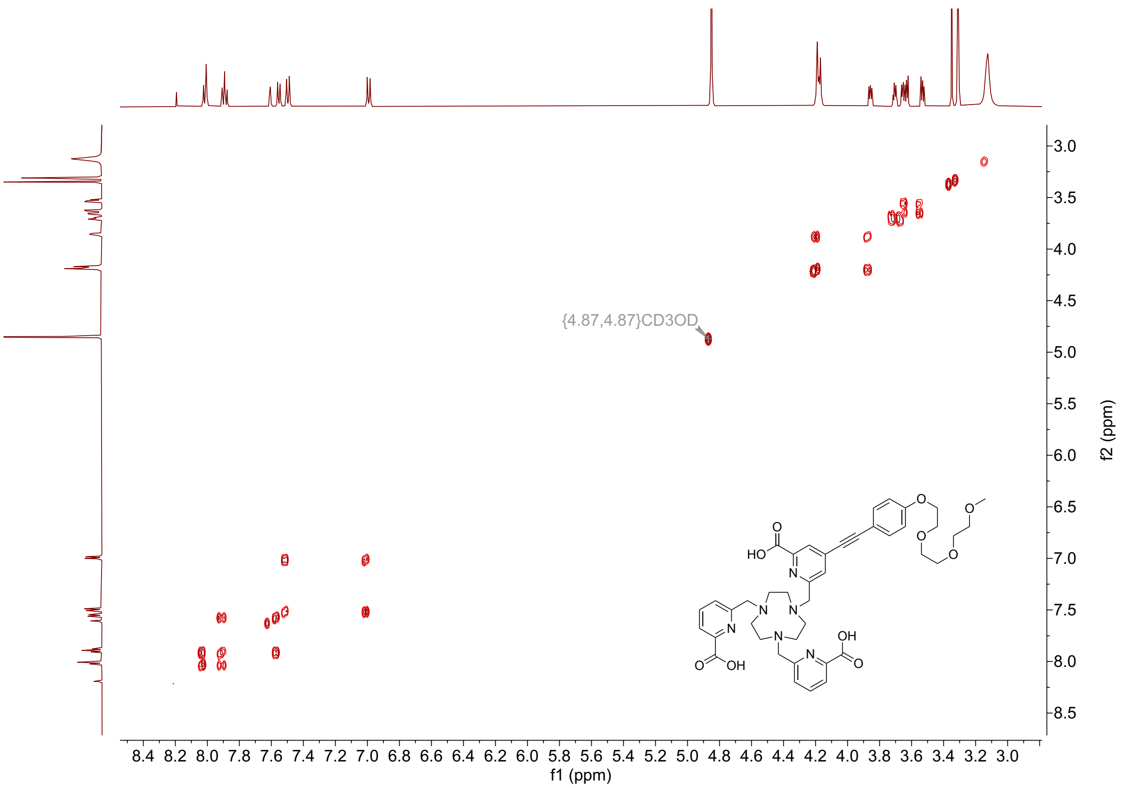

Figure S92. ^{1}H-^{1}H COSY NMR spectrum of **pepa-pic₂** (500 MHz, MeOD-d_4).

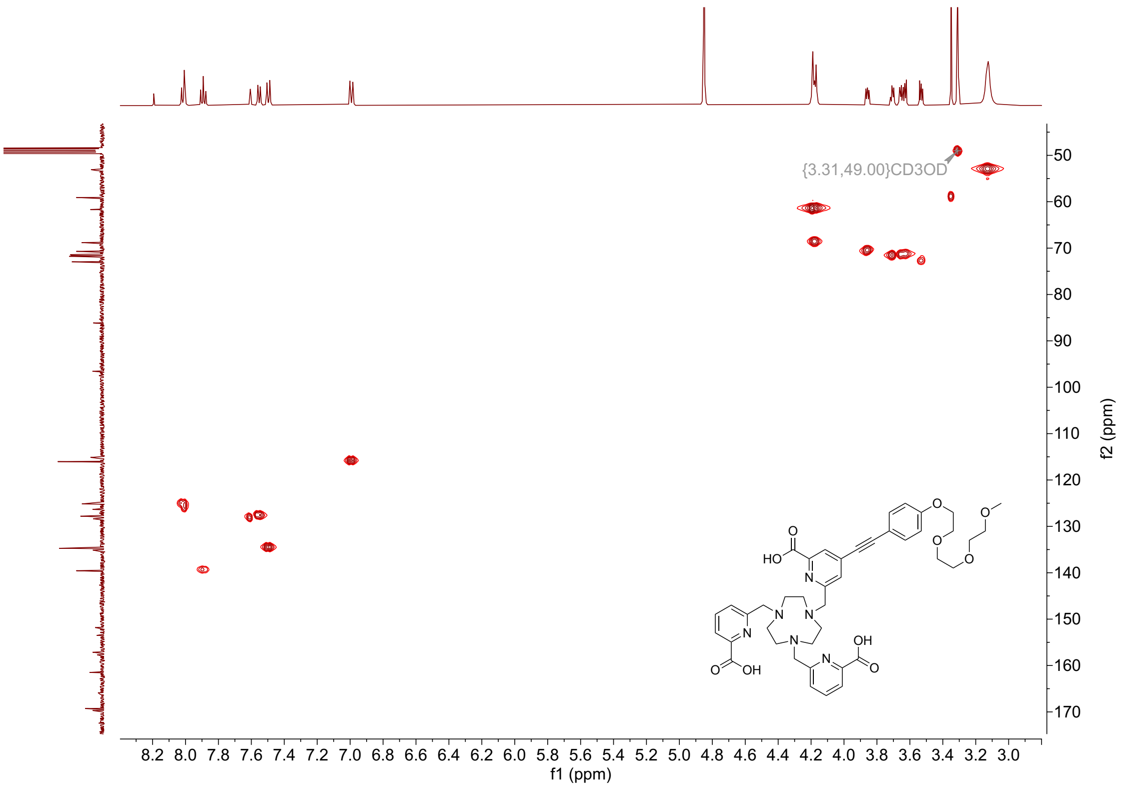

Figure S93. ^{1}H-^{13}C HSQC NMR spectrum of **pepa-pic₂** (500 MHz, MeOD-d_4).

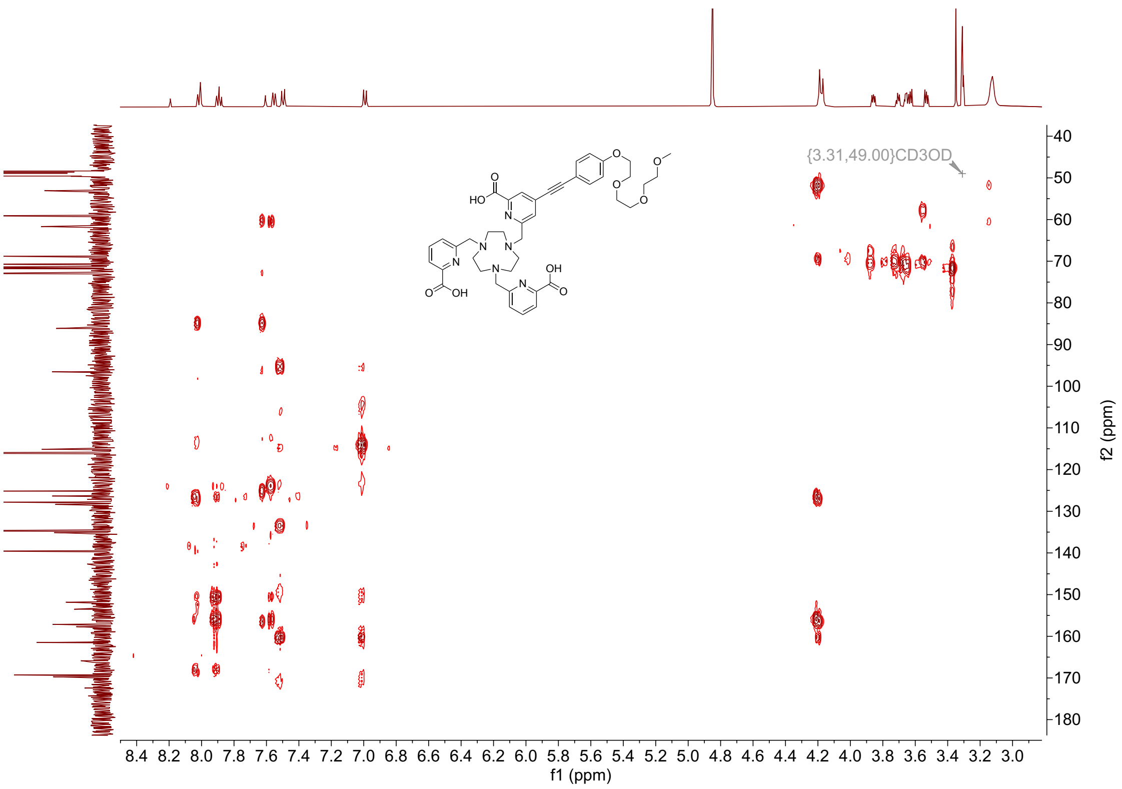

Figure S94. ^{1}H-^{13}C HMBC NMR spectrum of **pepa-pic$_2$** (500 MHz, MeOD-d_4).

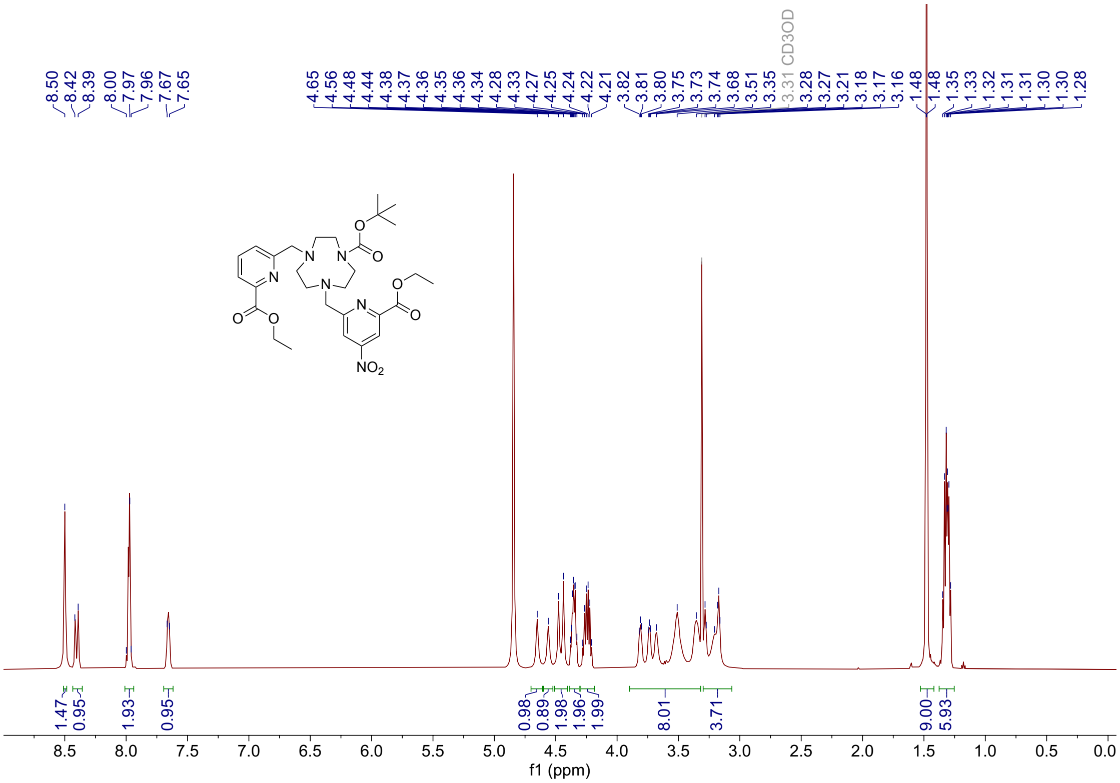

Figure S95. ^{1}H NMR spectrum of **10** (500 MHz, MeOD-d_4).

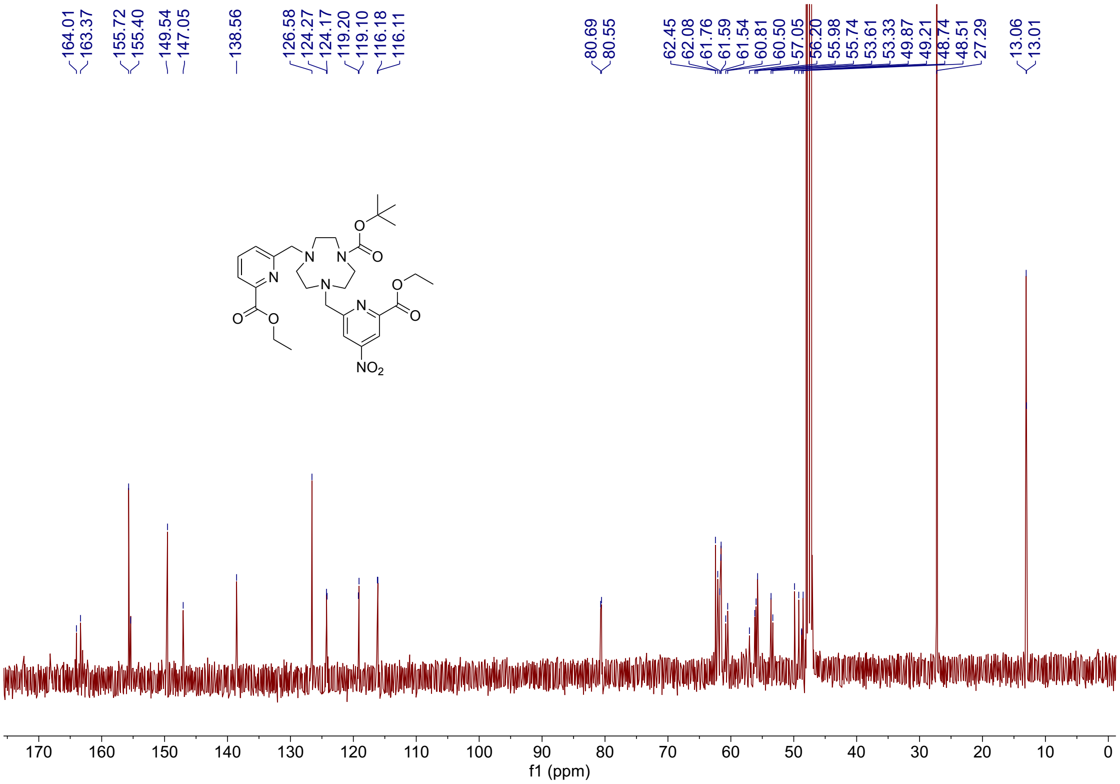

Figure S96. ^{13}C NMR spectrum of **10** (126 MHz, MeOD-d_4).

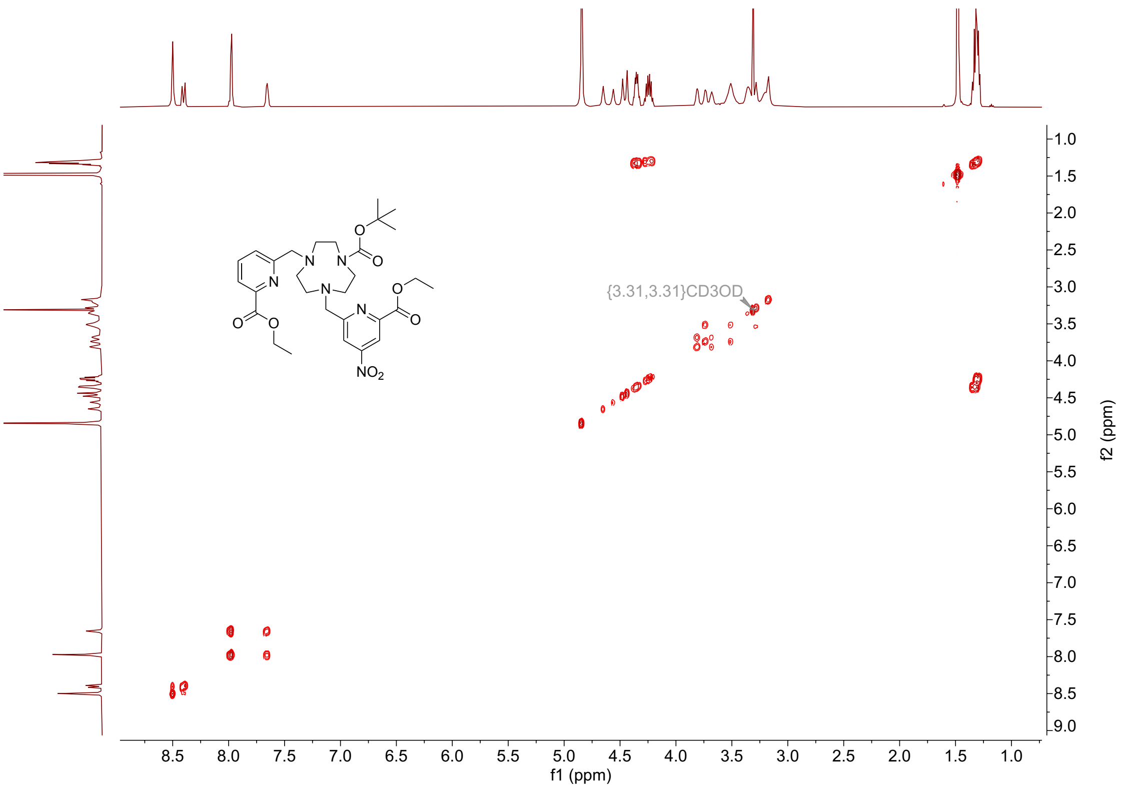

Figure S97. ^{1}H-^{1}H COSY NMR spectrum of **10** (500 MHz, MeOD-d_4).

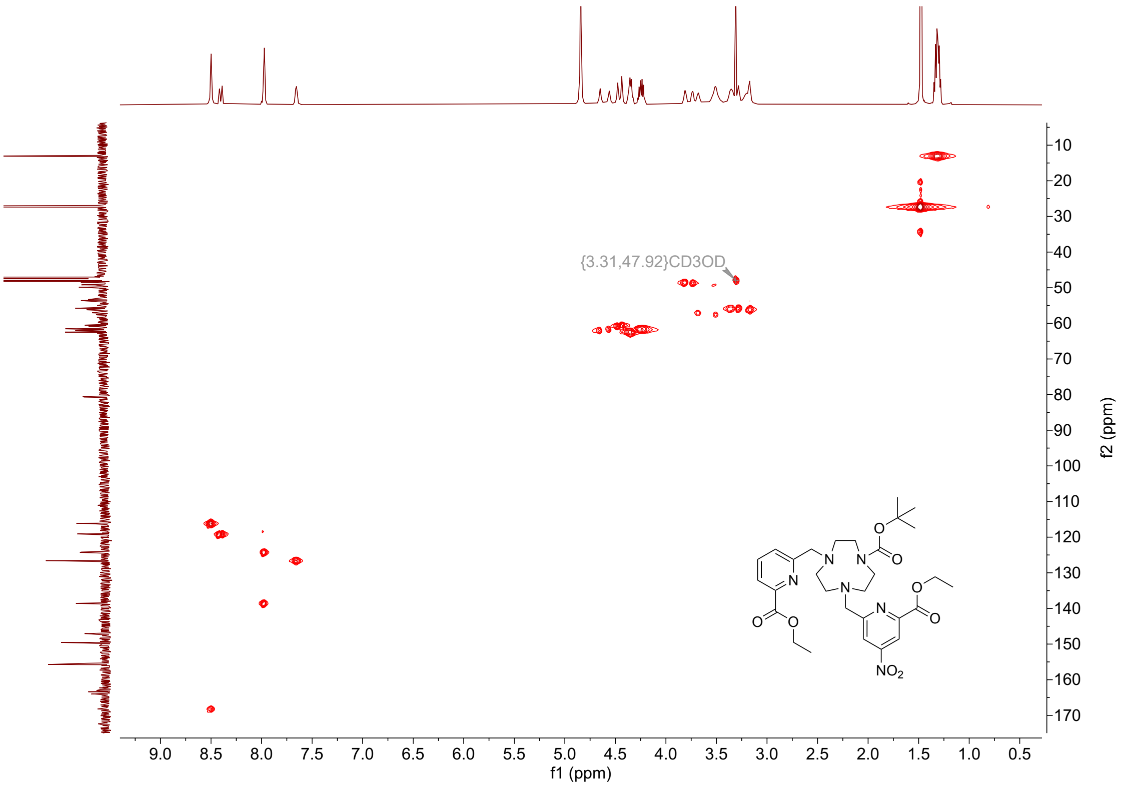

Figure S98. ^{1}H-^{13}C HSQC NMR spectrum of **10** (500 MHz, MeOD-d_4).

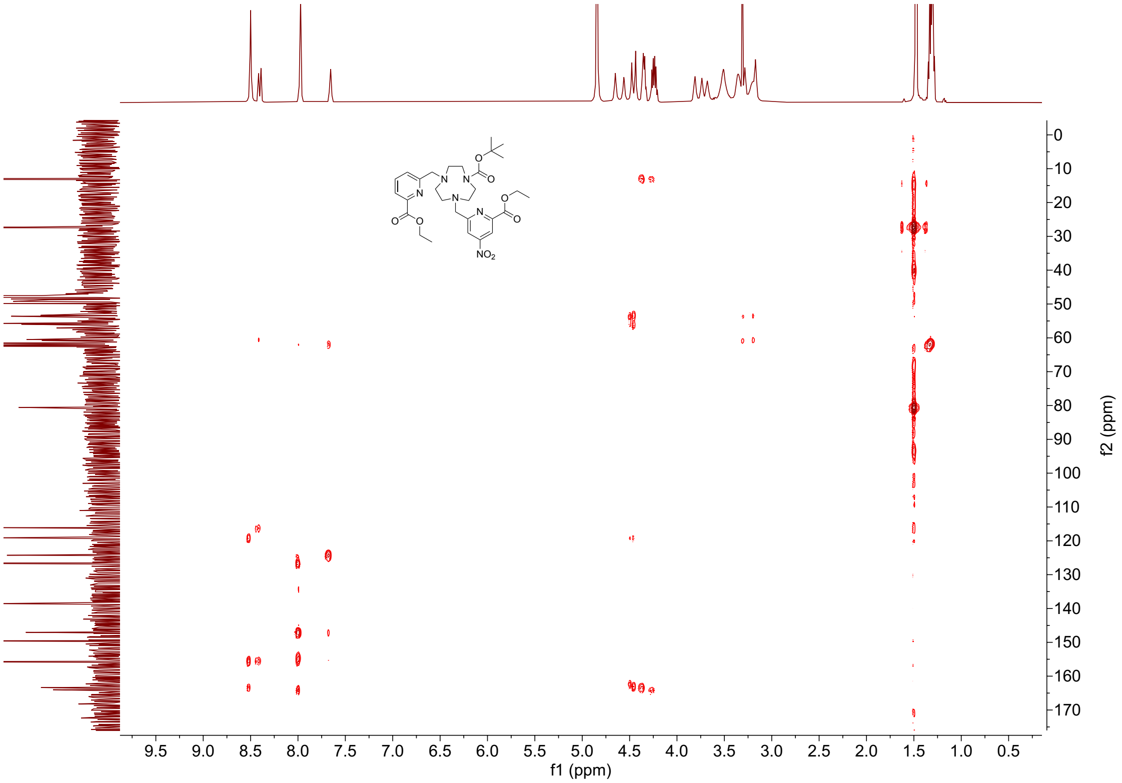

Figure S 99. ^{1}H-^{13}C HMBC NMR spectrum of **10** (500 MHz, MeOD-d_4).

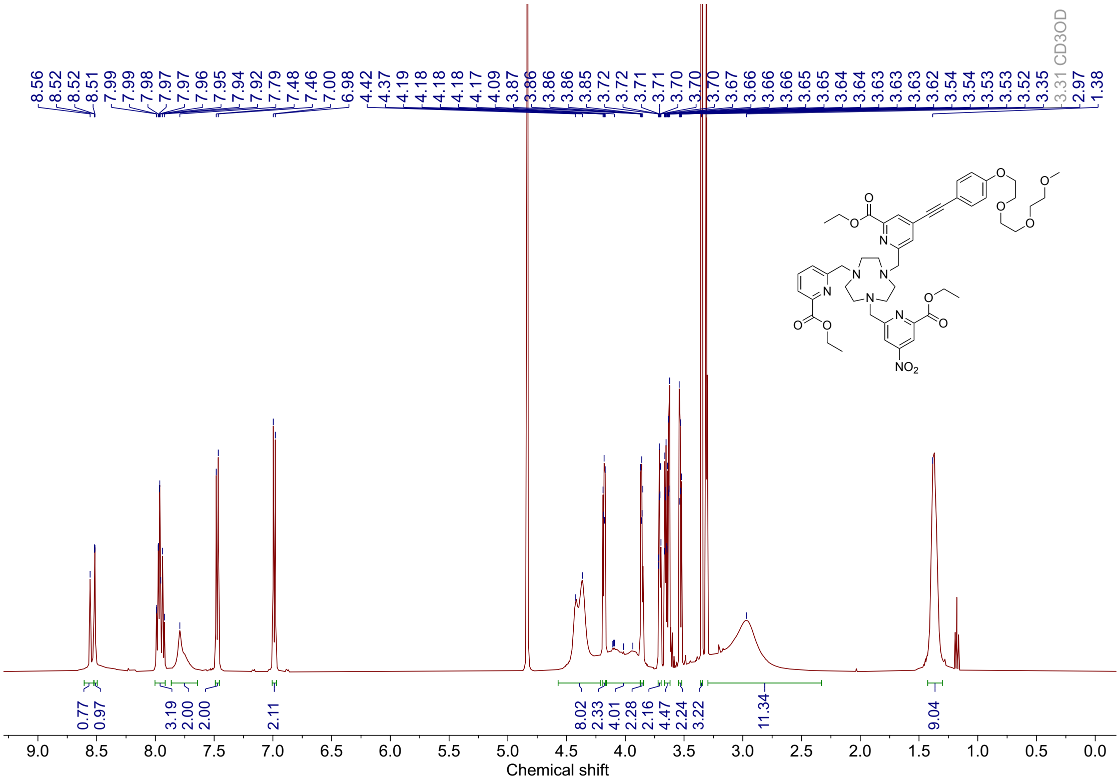

Figure S100. ^{1}H NMR spectrum of **11** (500 MHz, MeOD-d_4).

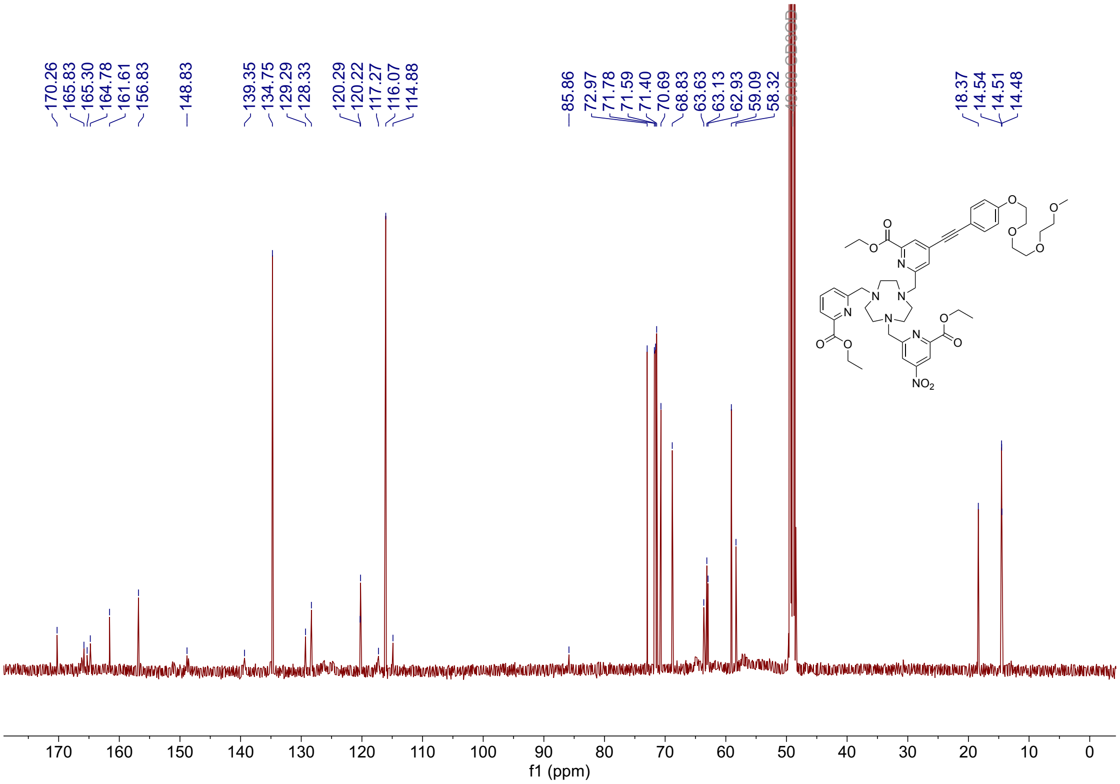

Figure S101. ^{13}C NMR spectrum of **11** (126 MHz, MeOD-d_4).

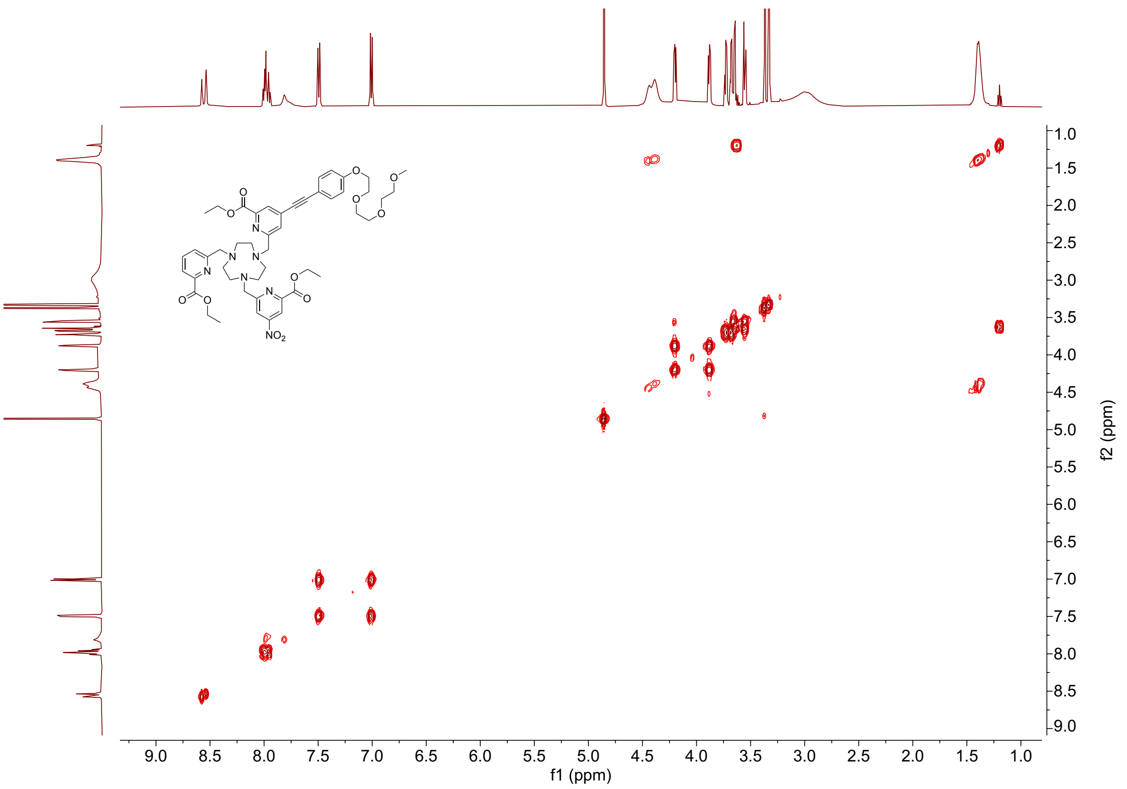

Figure S102. ^{1}H-^{1}H COSY NMR spectrum of **11** (500 MHz, MeOD-d_4).

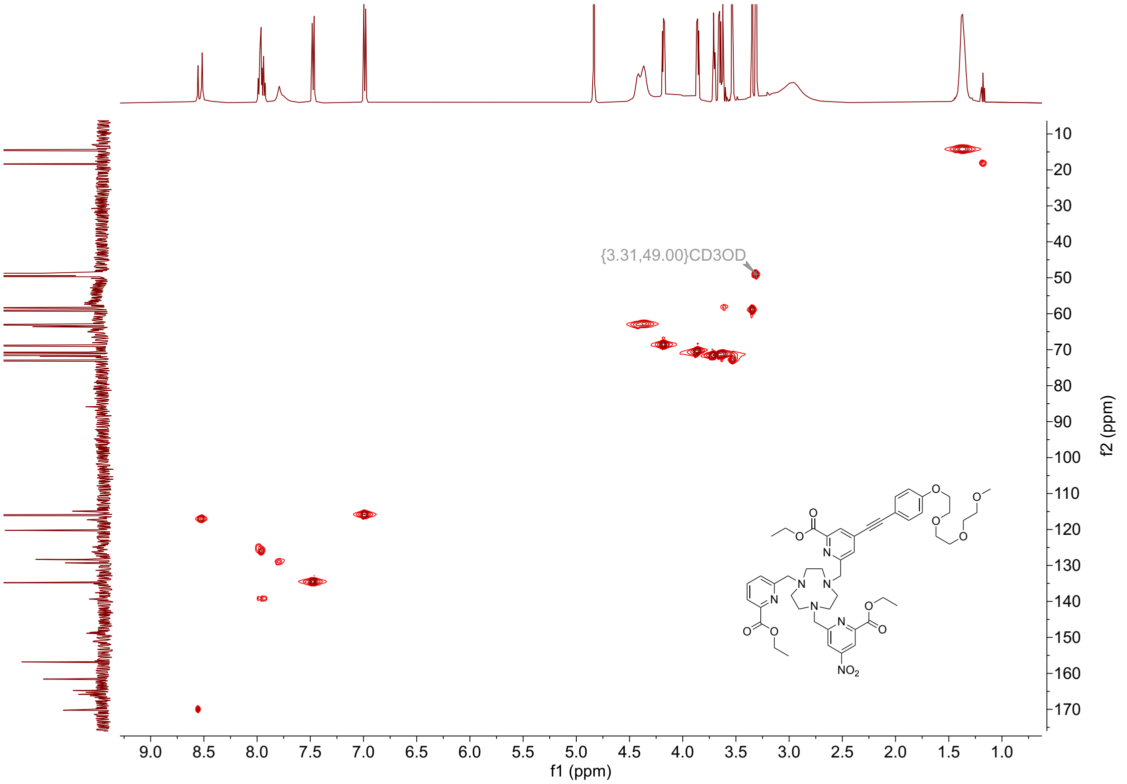

Figure S103. ^{1}H-^{13}C HSQC NMR spectrum of **11** (500 MHz, MeOD-d_4).

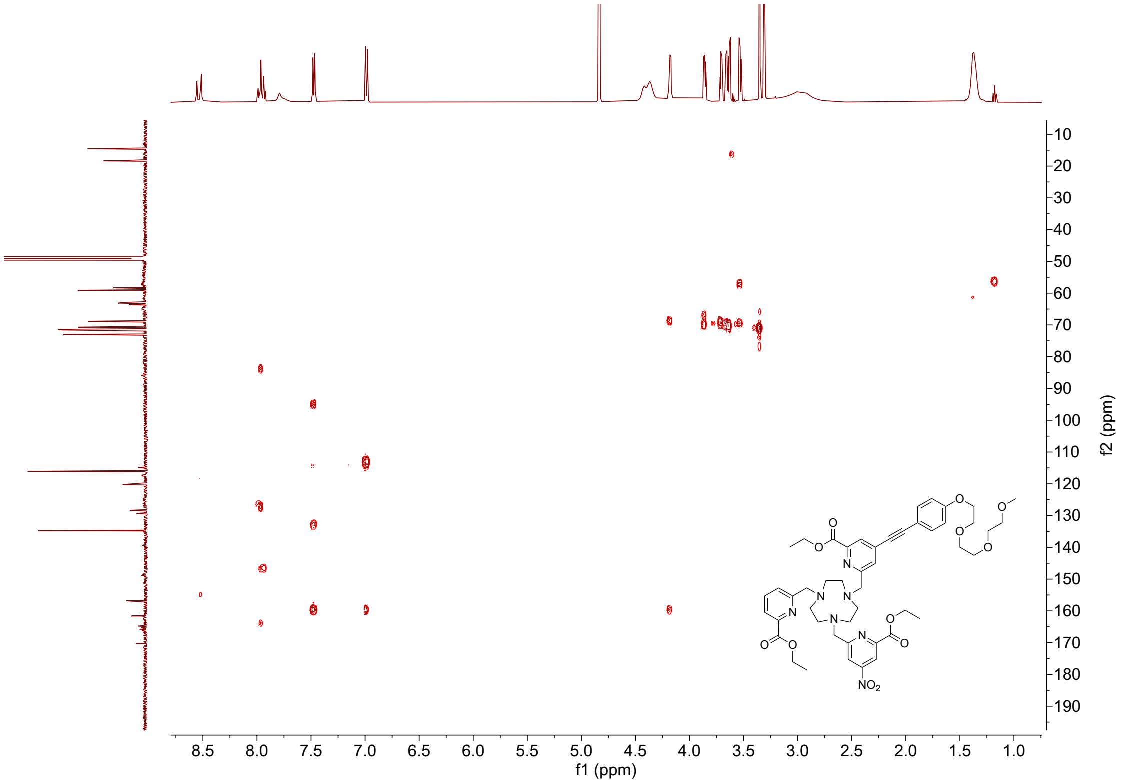

Figure S104. ^{1}H-^{13}C HMBC NMR spectrum of **11** (500 MHz, MeOD-d_4).

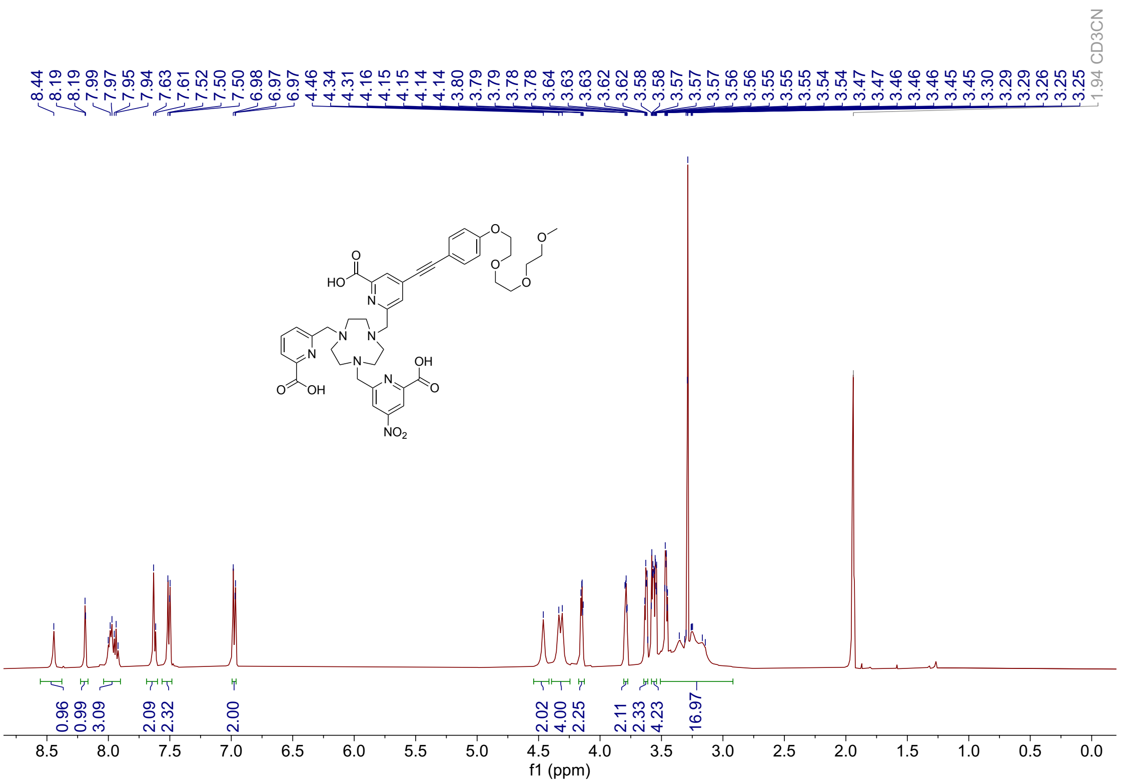

Figure S105. ^{1}H NMR spectrum of **12** (500 MHz, CD_3CN).

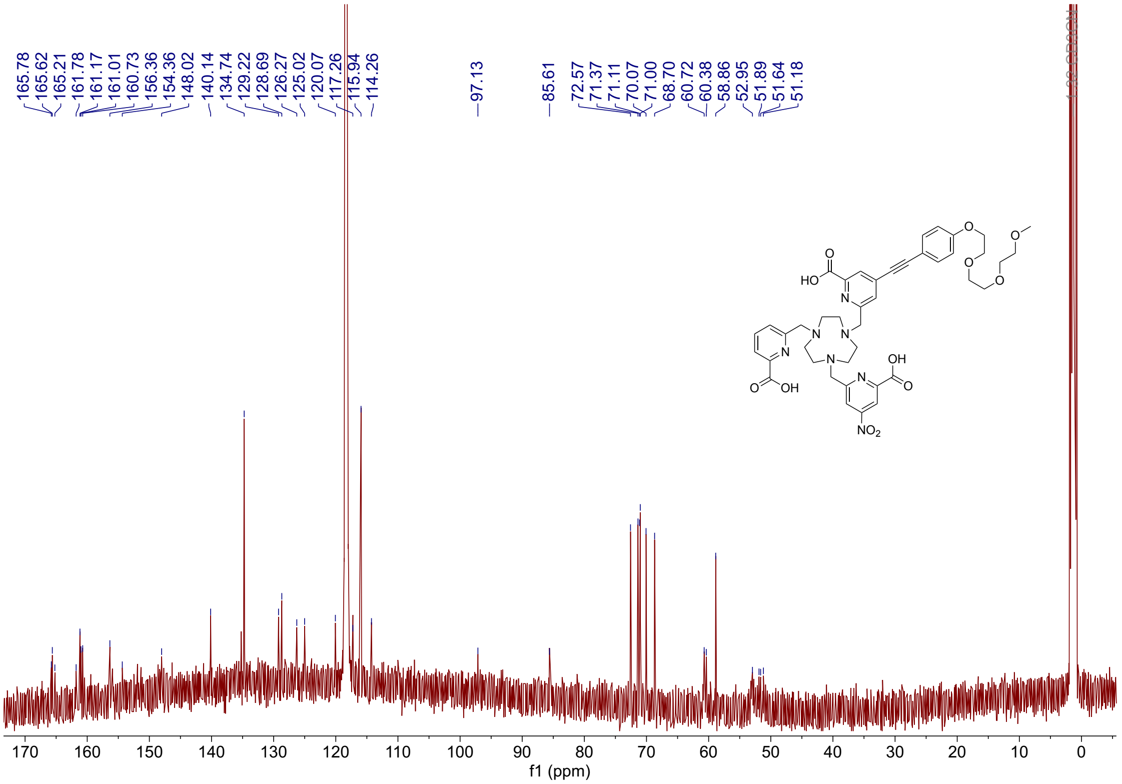

Figure S106. ^{13}C NMR spectrum of **12** (500 MHz, CD_3CN).

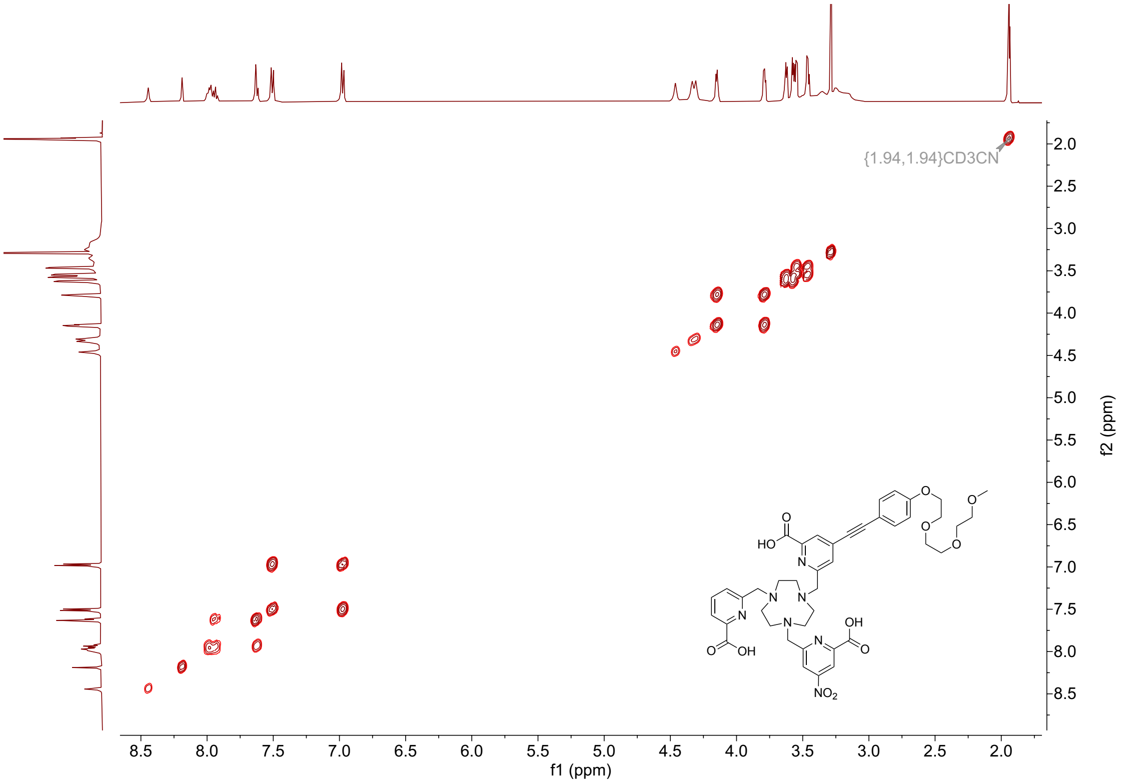

Figure S107. ^{1}H-^{1}H COSY NMR spectrum of **12** (500 MHz, CD_3CN).

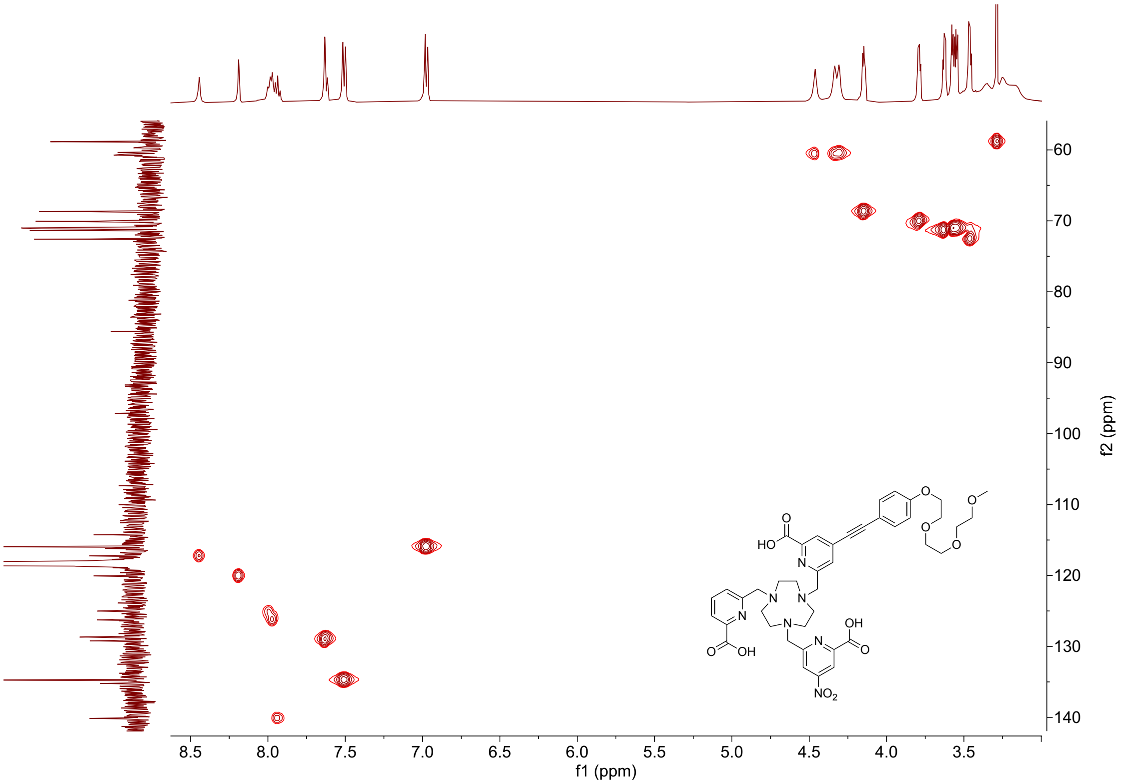

Figure S108. ^{1}H-^{13}C HSQC NMR spectrum of **12** (500 MHz, CD_3CN).

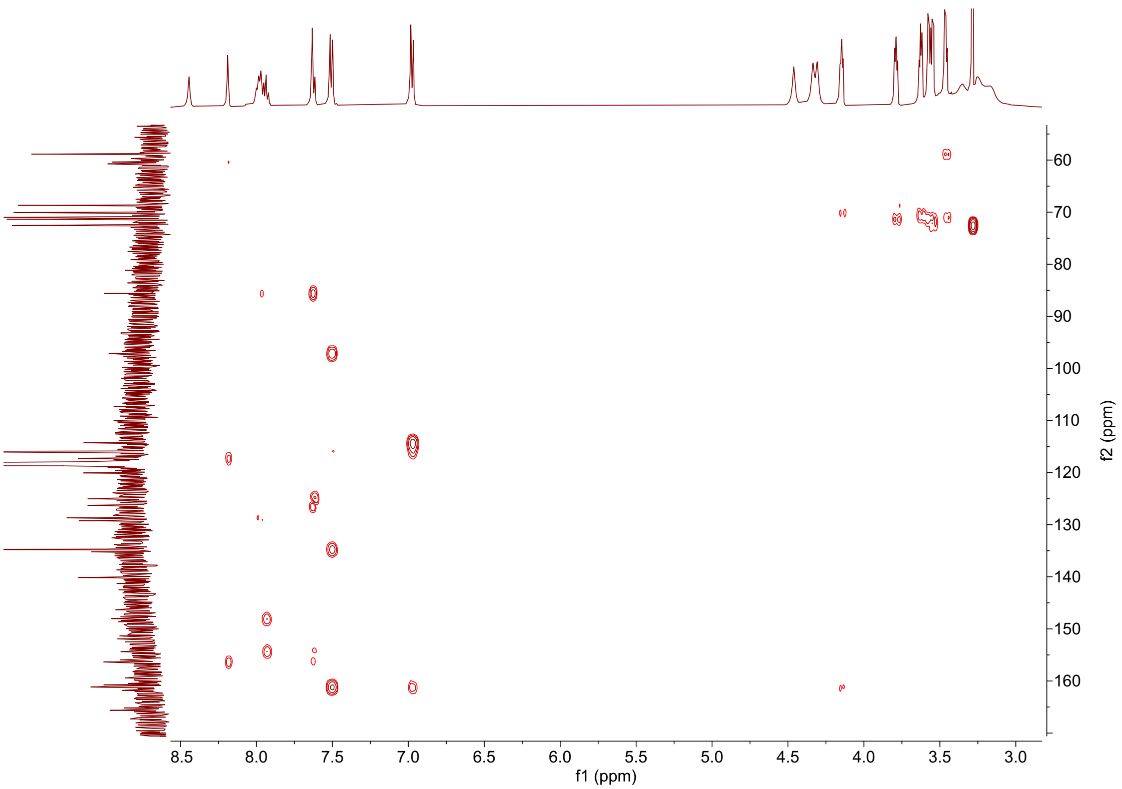

Figure S109. ^{1}H-^{13}C HMBC NMR spectrum of **12** (500 MHz, CD_3CN).

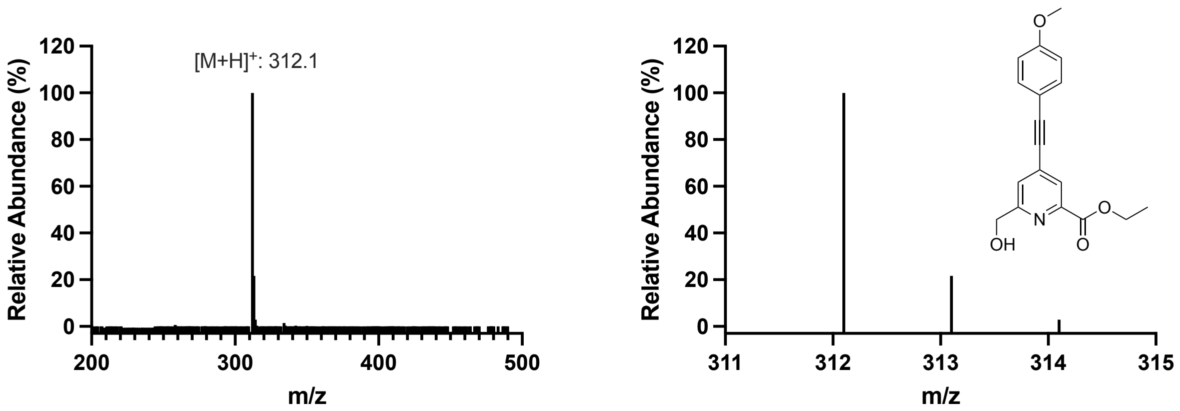

Figure S110. ESI-MS of **2.** Calc m/z for $C_{18}H_{18}NO_4$ $[M+H]^+$: 312.1. Found: 312.1.

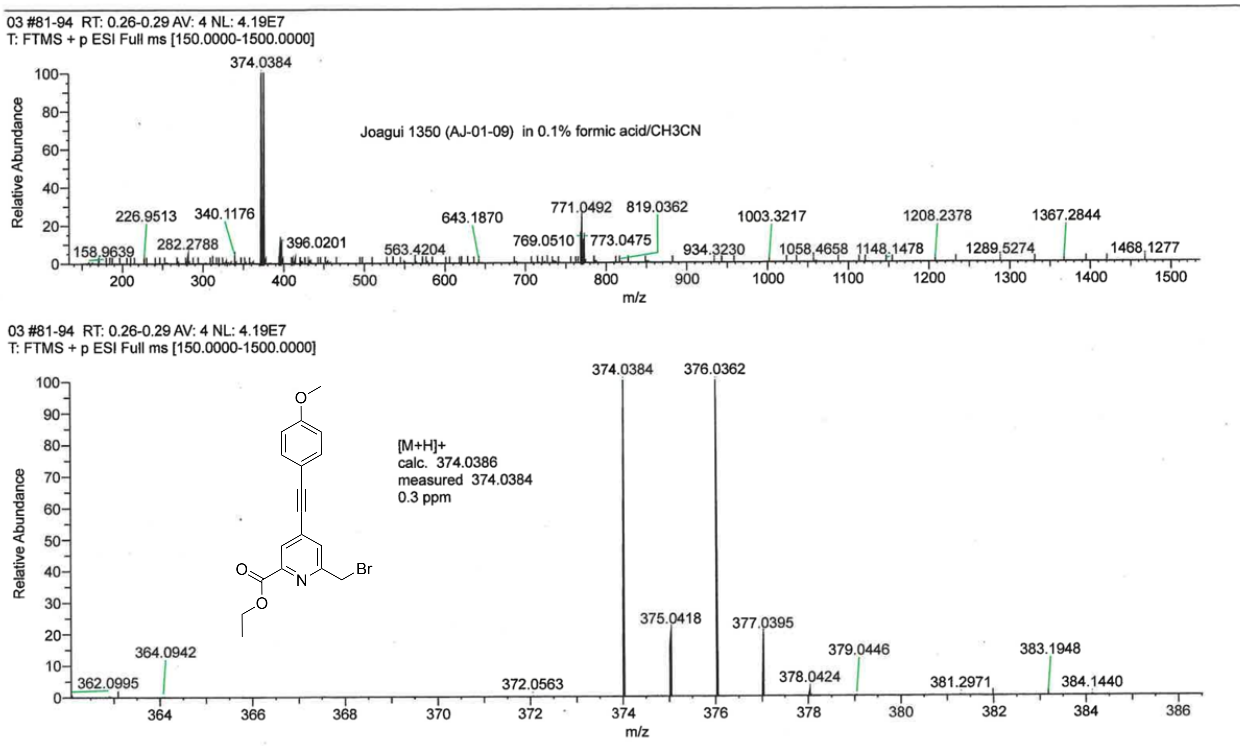

Figure S111. ESI-HRMS of **3**. Calc m/z for $C_{18}H_{17}BrNO_3$ $[M+H]^+$: 374.0386. Found: 374.0384.

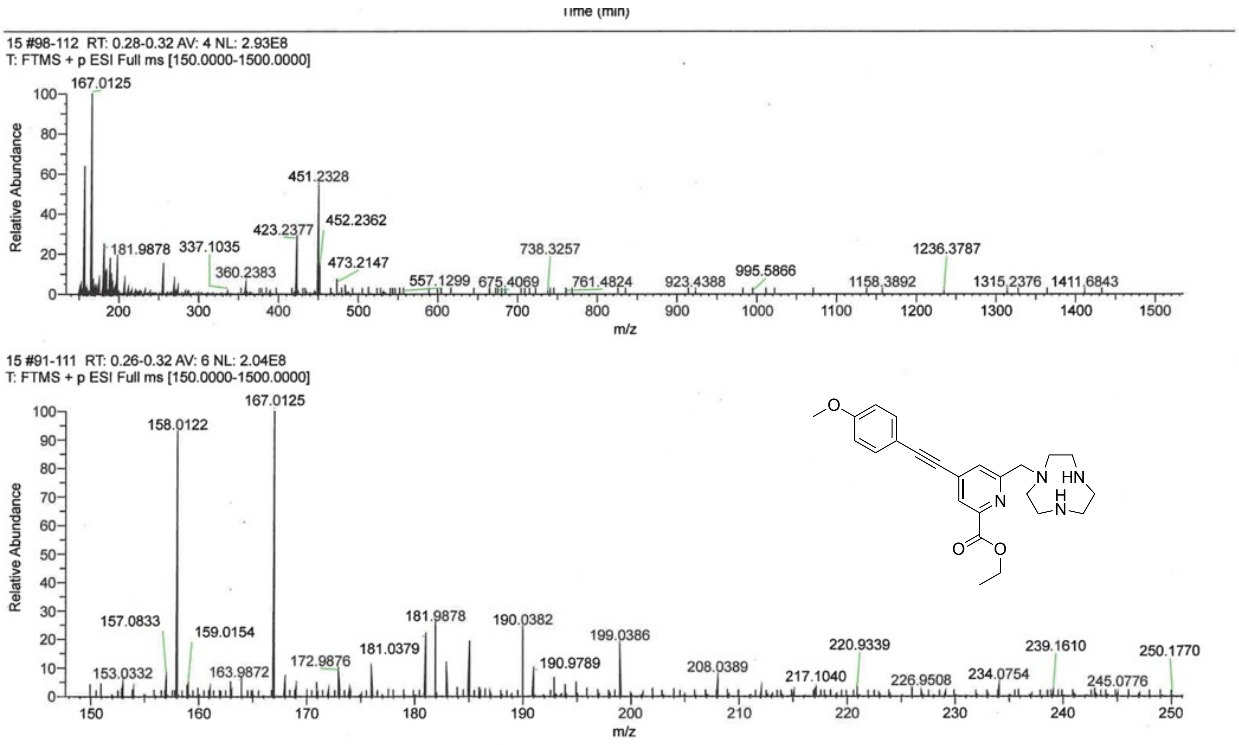

Figure S112. ESI-HRMS of **4**. Calc m/z for $C_{24}H_{31}N_4O_3$ $[M+H]^+$: 423.2396. Found: 423.2377.

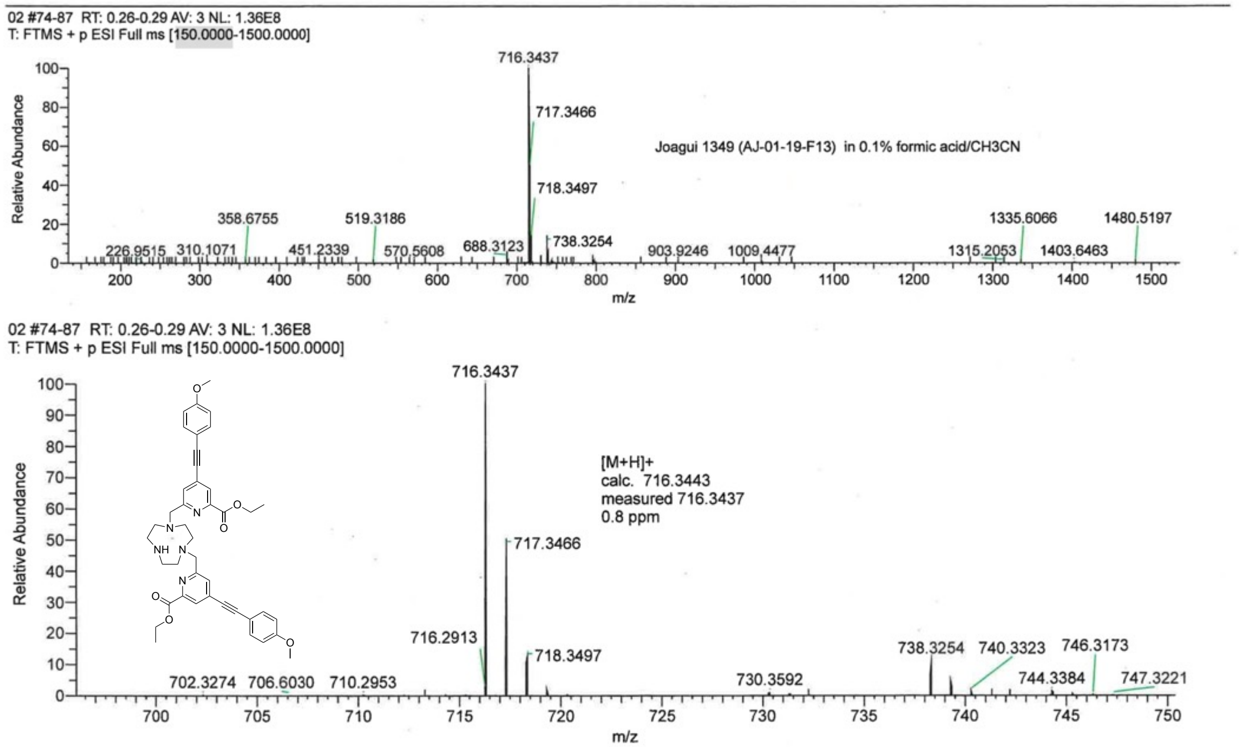

Figure S113. ESI-HRMS of **5**. Calc m/z for $C_{42}H_{46}N_5O_6$ $[M+H]^+$: 716.3443. Found: 716.3447.

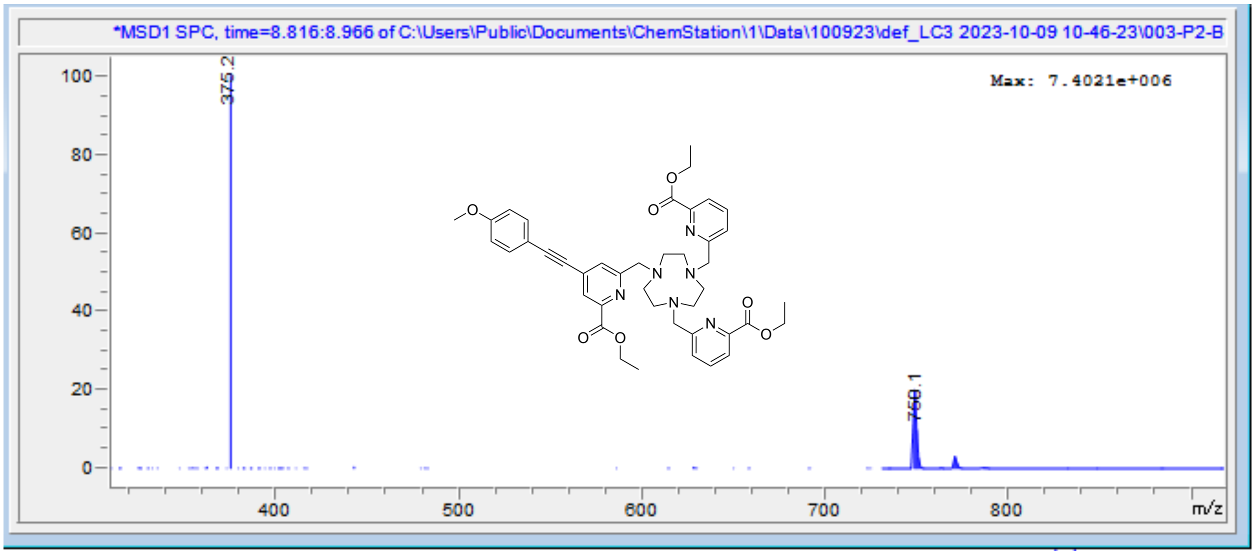

Figure S114. ESI-MS of **6**. Calc m/z for $C_{42}H_{49}N_6O_7$ $[M+H]^+$: 749.4. Found: 749.1.4. Calc for $C_{42}H_{50}N_6O_7$ $[M+2H]^{2+}$: 375.2. Found: 375.2.

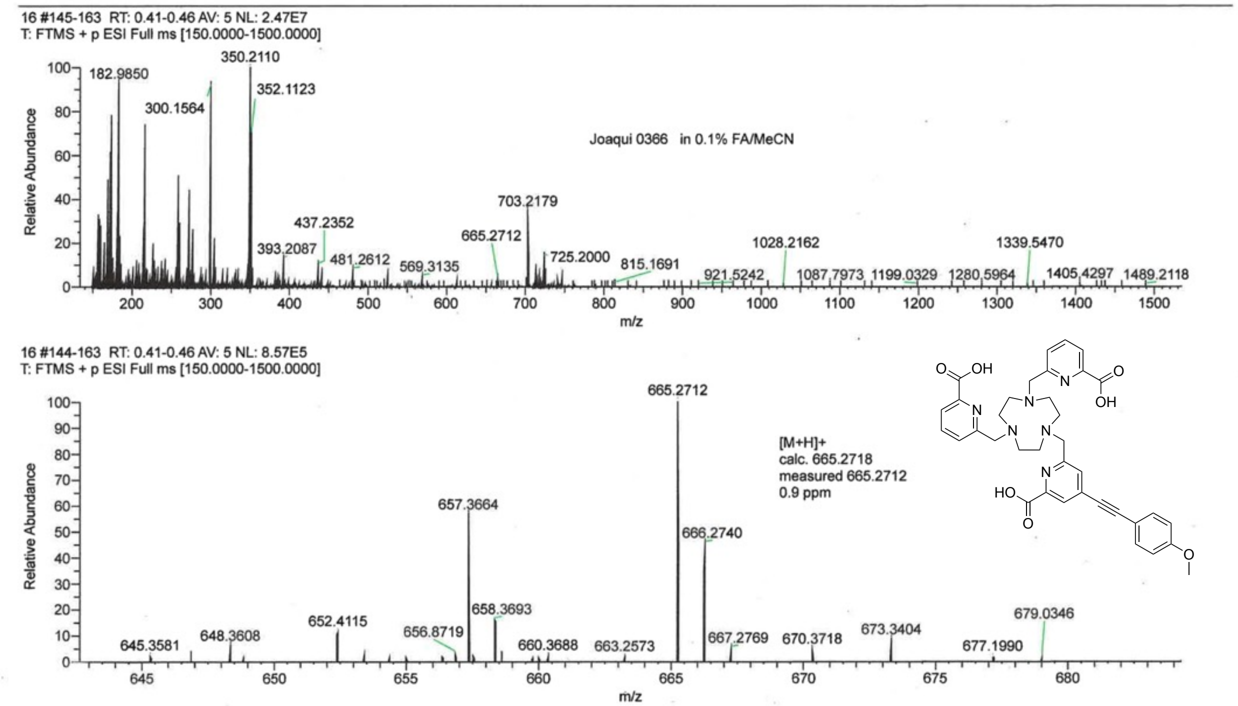

Figure S115. ESI-HRMS of **mepa-pic$_2$**. Calc m/z for $C_{36}H_{37}N_6O_7$ $[M+H]^+$: 665.2718. Found: 665.2712.

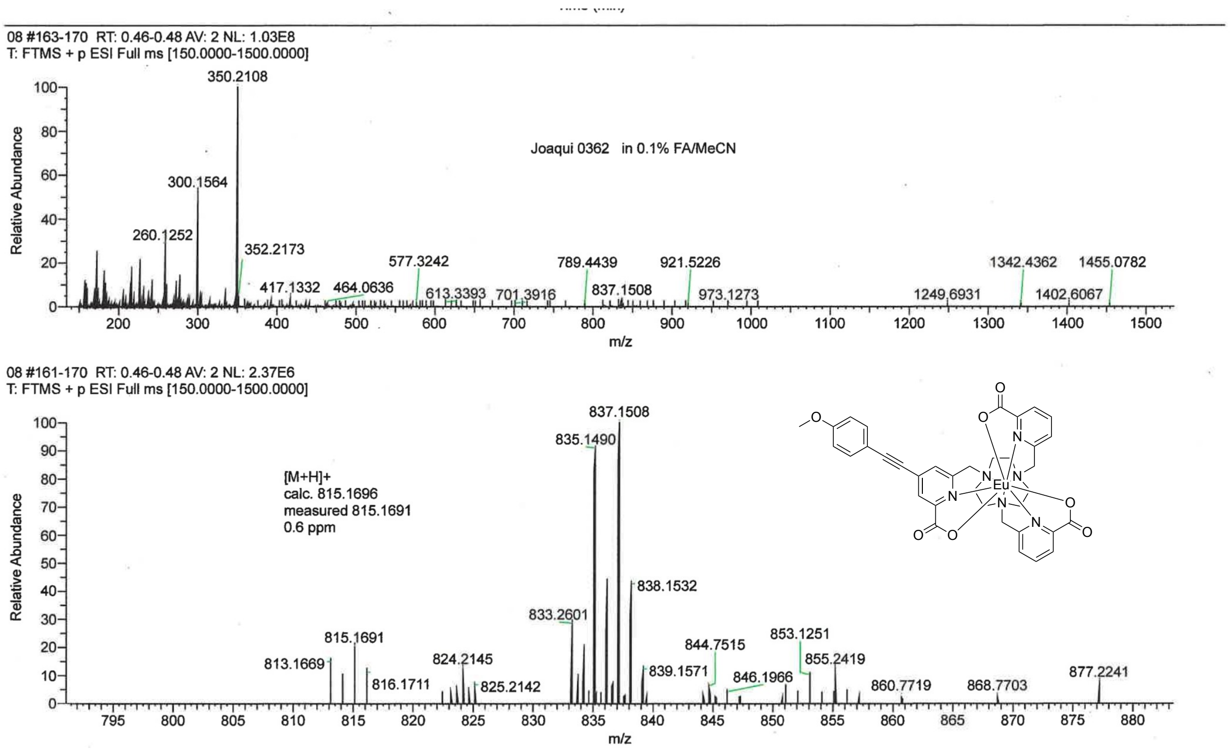

Figure S116. ESI-HRMS of **^{nat}Eu-mepa-pic$_2$**. Calc m/z for $C_{36}H_{34}EuN_6O_7$ [M+H]$^+$: 815.1696. Found: 815.1696. Calc for $C_{36}H_{33}EuN_6NaO_7$ [M+Na]$^+$: 837.1521. Found: 815.1508.

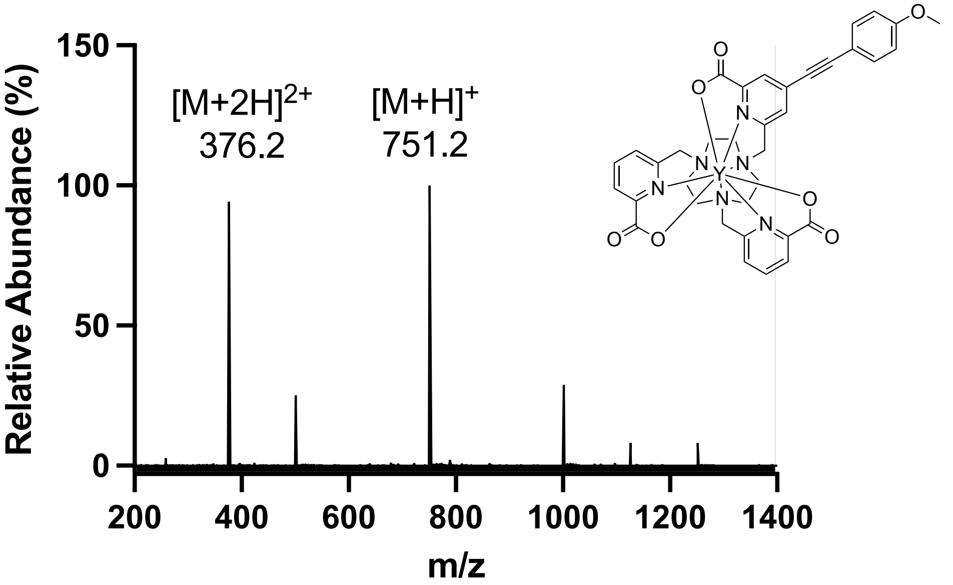

Figure S117. ESI-MS of **^{nat}Y-mepa-pic$_2$**. Calc m/z for $C_{36}H_{34}N_6O_7Y$ [M+H]$^+$: 751.2. Found: 751.2. Calc for $C_{36}H_{34}N_6O_7Y$ [M+2H]$^{2+}$: 376.1. Found: 376.2.

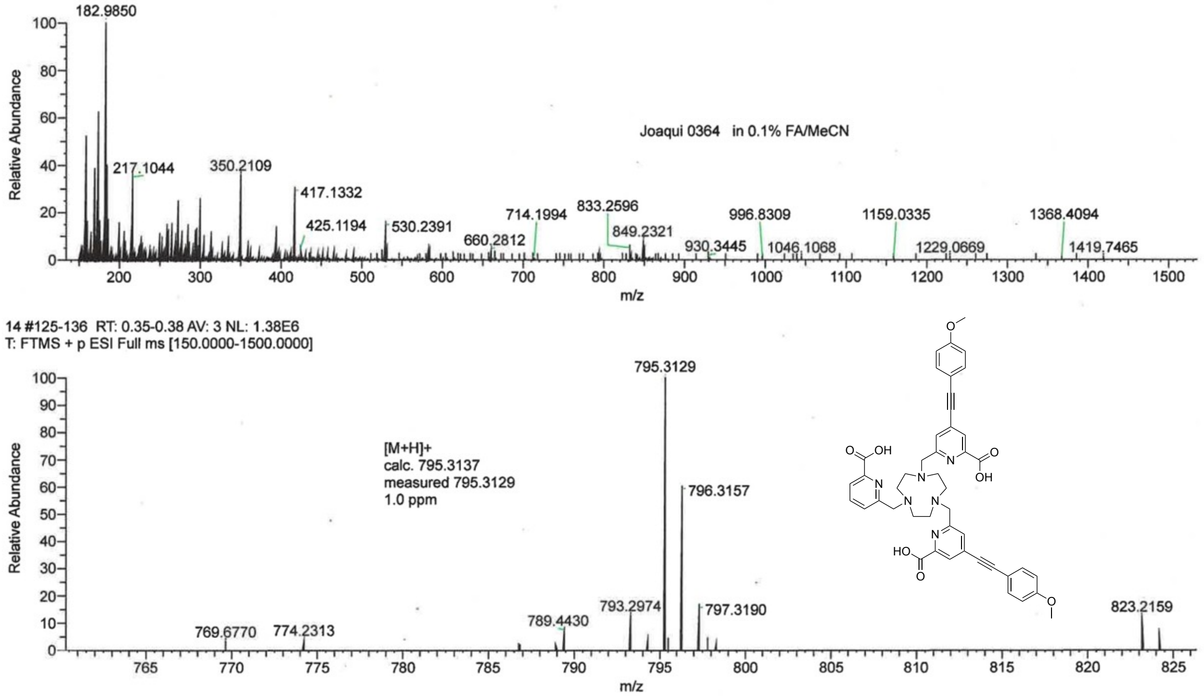

Figure S118. ESI-HRMS of **mepa$_2$-pic**. Calc for $C_{45}H_{43}N_6O_8$ $[M+H]^+$: 795.3137. Found: 795.3129.

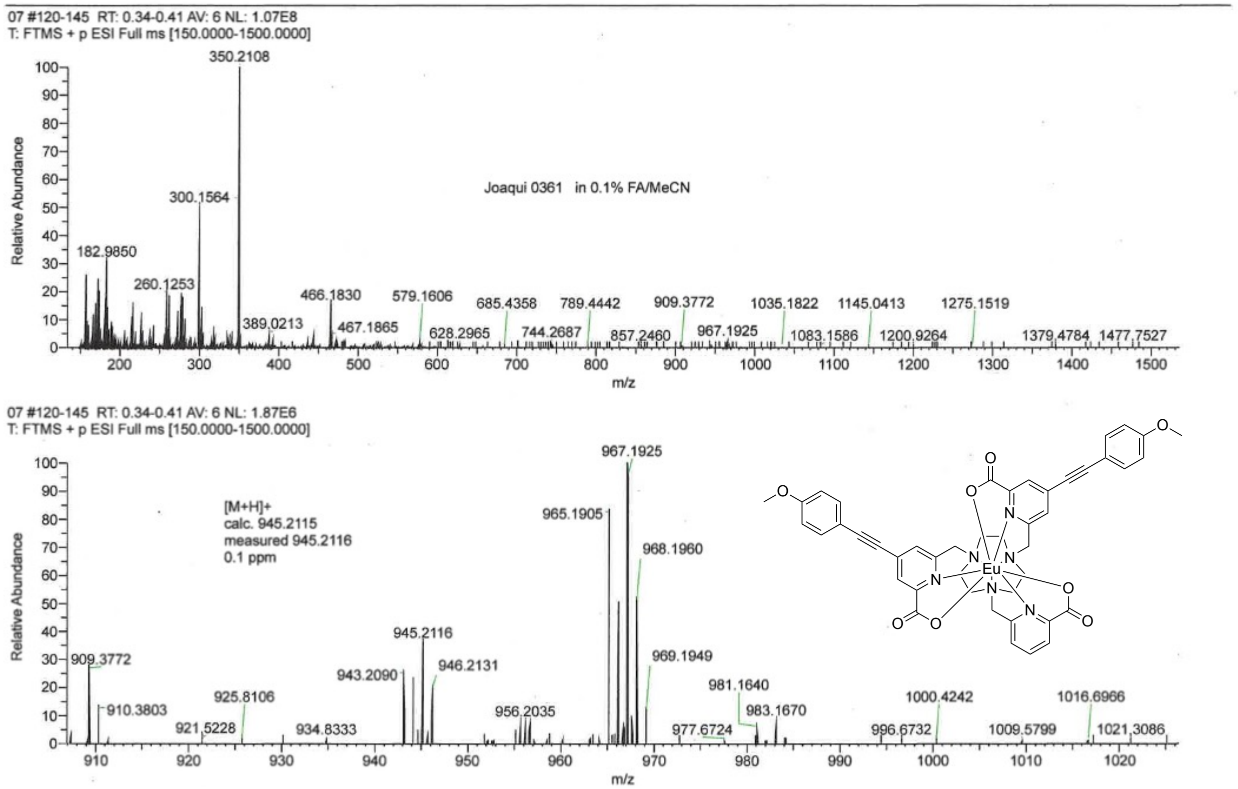

Figure S119. ESI-HRMS of **^{nat}Eu-mepa$_2$-pic**. Calc m/z for $C_{45}H_{40}EuN_6O_8$ $[M+H]^+$: 945.2115. Found: 945.2116. Calc m/z for $C_{45}H_{39}EuN_6NaO_8$ 967.1939 $[M+Na]^+$. Found 967.1925.

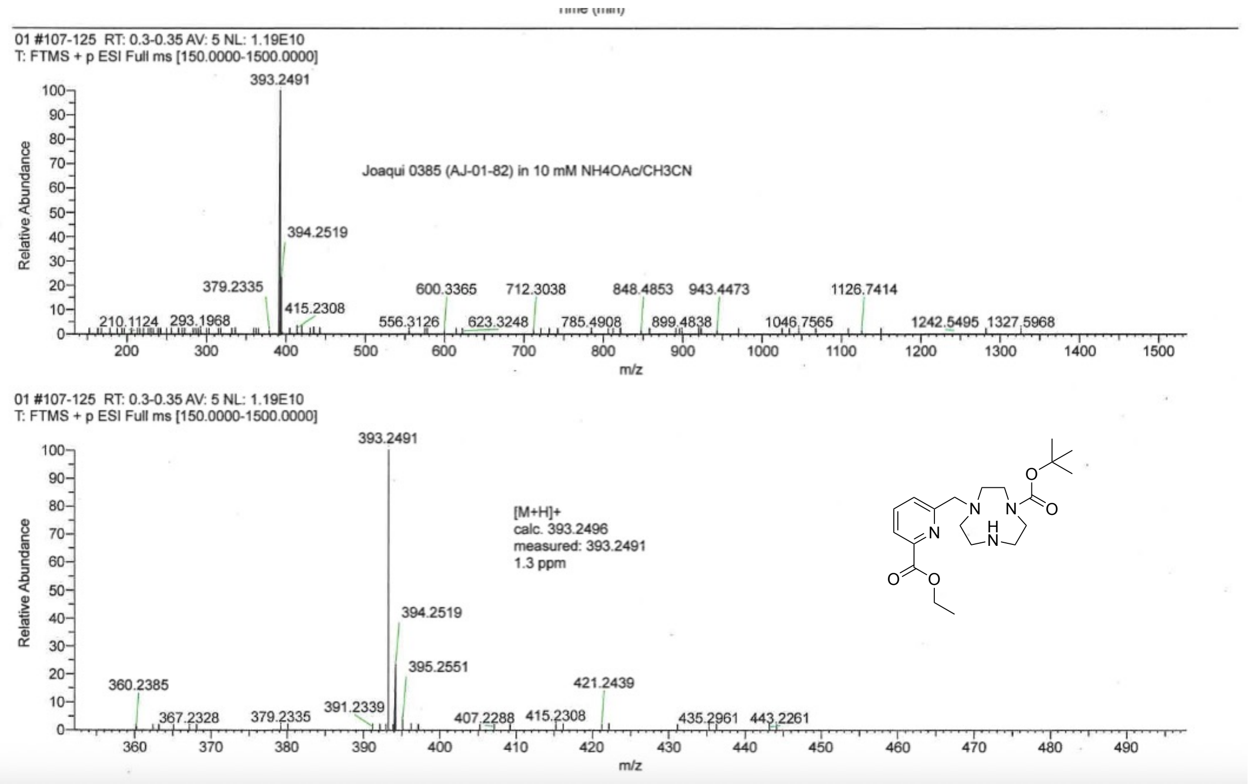

Figure S120. ESI-HRMS of **7**. Calc m/z for $C_{20}H_{33}N_4O_4$ $[M+H]^+$: 393.2496. Found: 393.2491

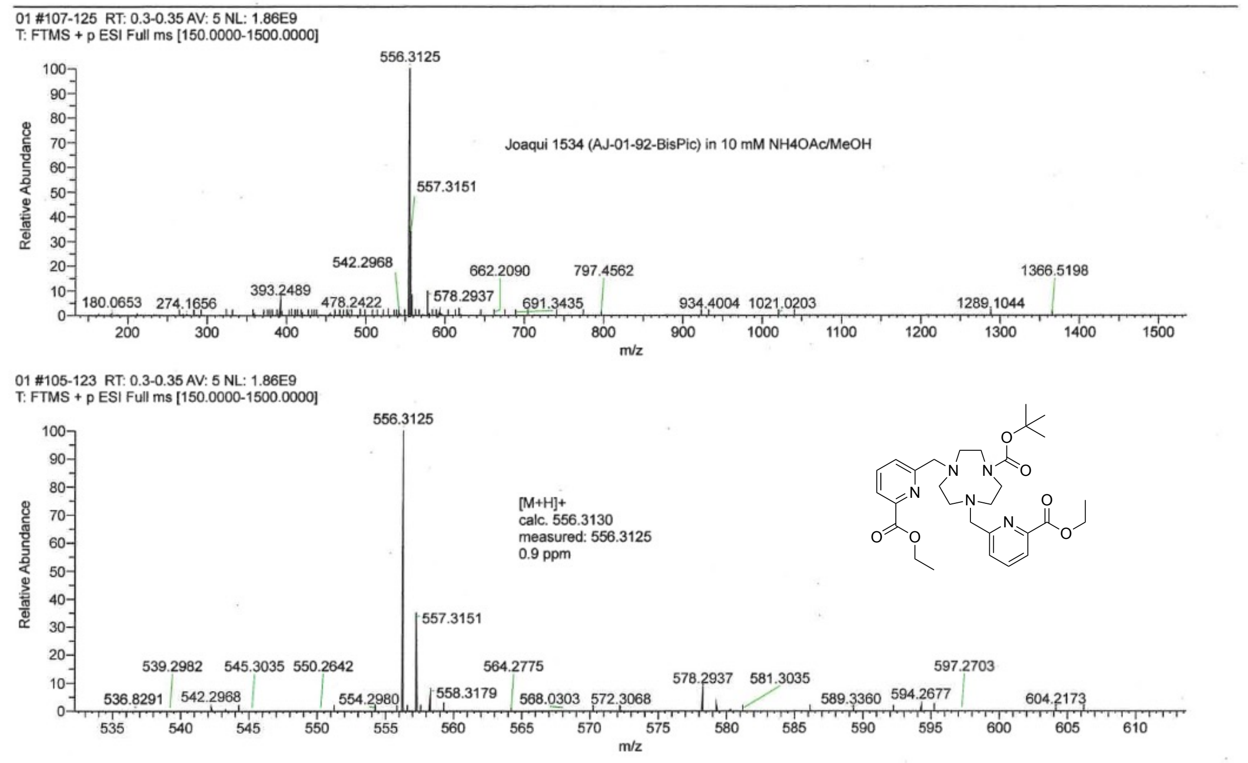

Figure S121. ESI-MS of **8**. Calc m/z for $C_{29}H_{42}N_5O_6$ $[M+H]^+$: 556.3130. Found: 556.3125.

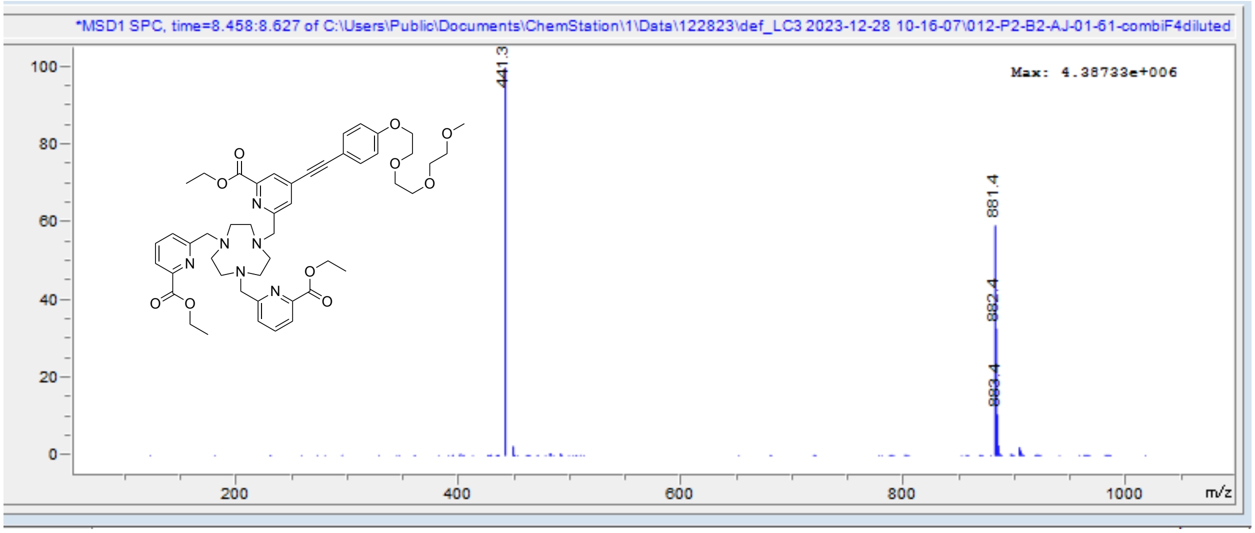

Figure S122. ESI-MS of **9**. Calc m/z for $C_{48}H_{61}N_6O_{10}$ $[M+H]^+$: 881.4. Found: 881.4. Calc m/z for $C_{48}H_{62}N_6O_{10}$ $[M+2H]^{2+}$: 441.2. Found: 441.3.

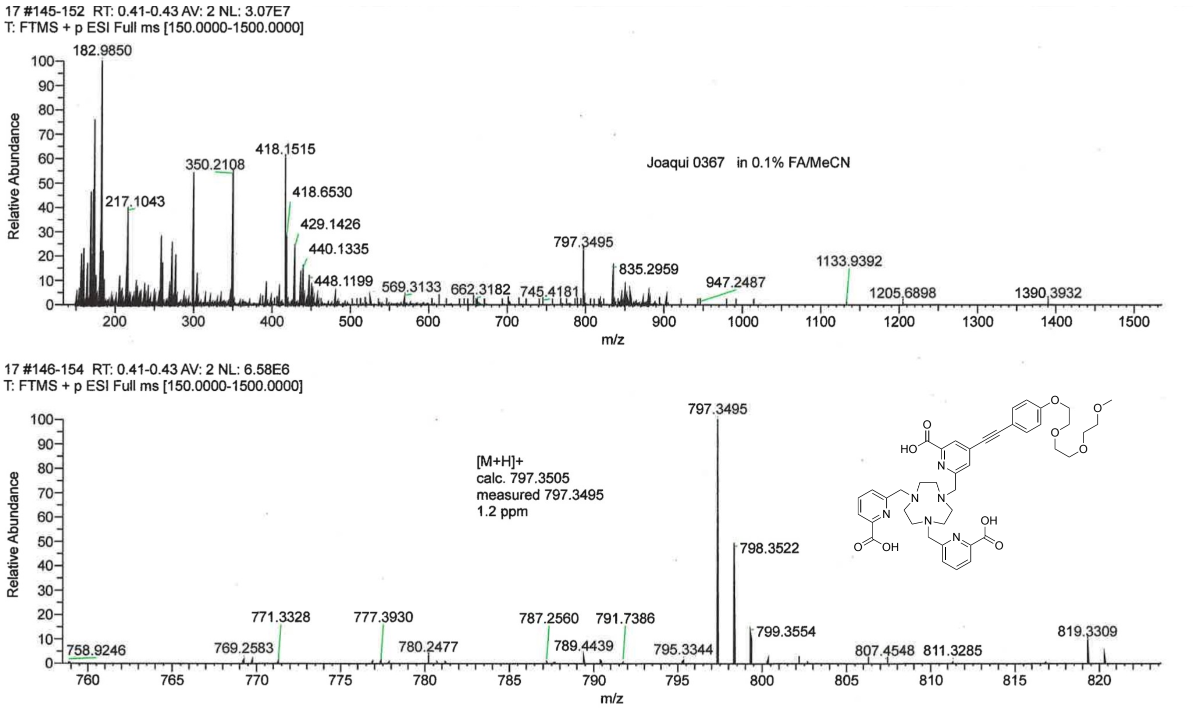

Figure S123. ESI-HRMS of **pepa-pic$_2$**. Calc m/z for $C_{42}H_{49}N_6O_{10}$ $[M+H]^+$: 797.3505. Found: 797.3495

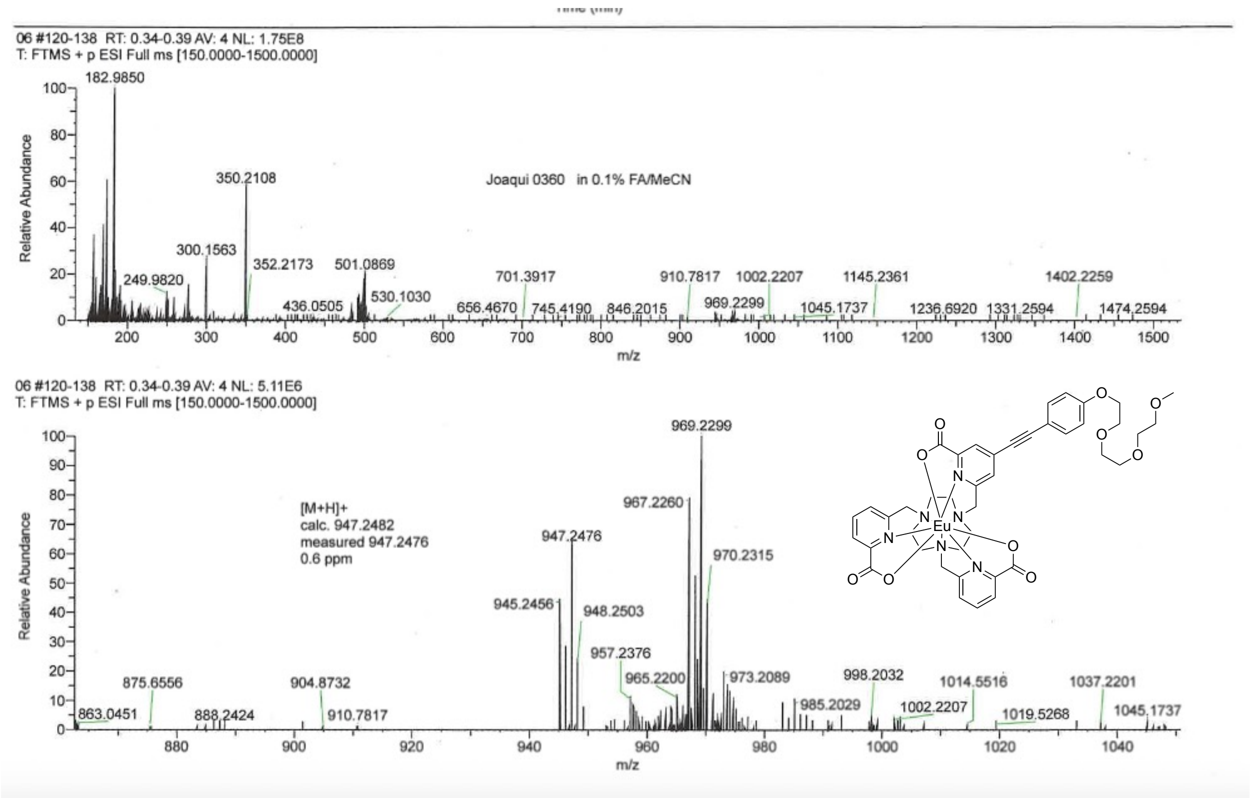

Figure S124. ESI-HRMS of **^{nat}Eu-pepa-pic$_2$**. Calc m/z for $C_{42}H_{46}EuN_6O_{10}$ $[M+H]^+$: 947.2482. Found: 947.2476. Calc for $C_{42}H_{45}EuN_6NaO_{10}$ $[M+H]^+$: 969.2307. Found: 969.2299.

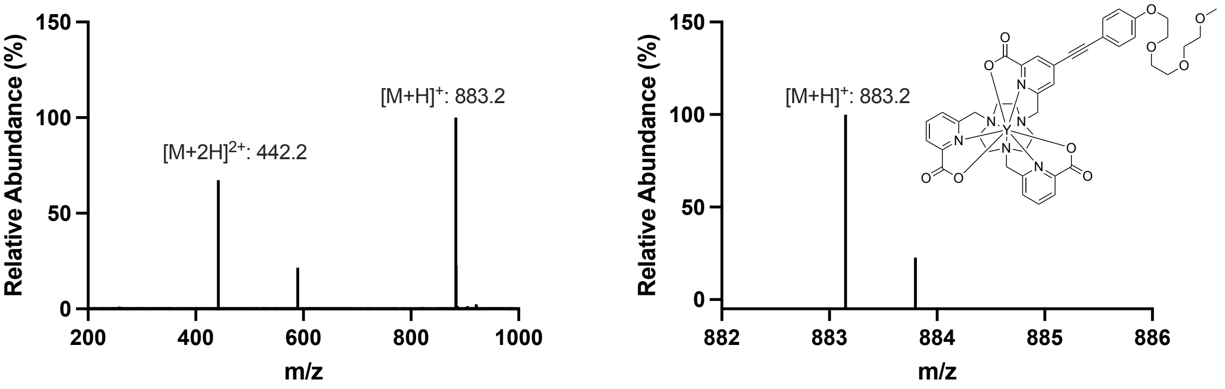

Figure S125. ESI-MS of **^{nat}Y-pepa-pic$_2$**. Calc m/z for $C_{42}H_{46}N_6O_{10}Y$ $[M+H]^+$: 883.2. Found: 883.2. Calc m/z for $C_{42}H_{47}N_6O_{10}Y$ $[M+2H]^{2+}$: 442.1. Found: 442.2.

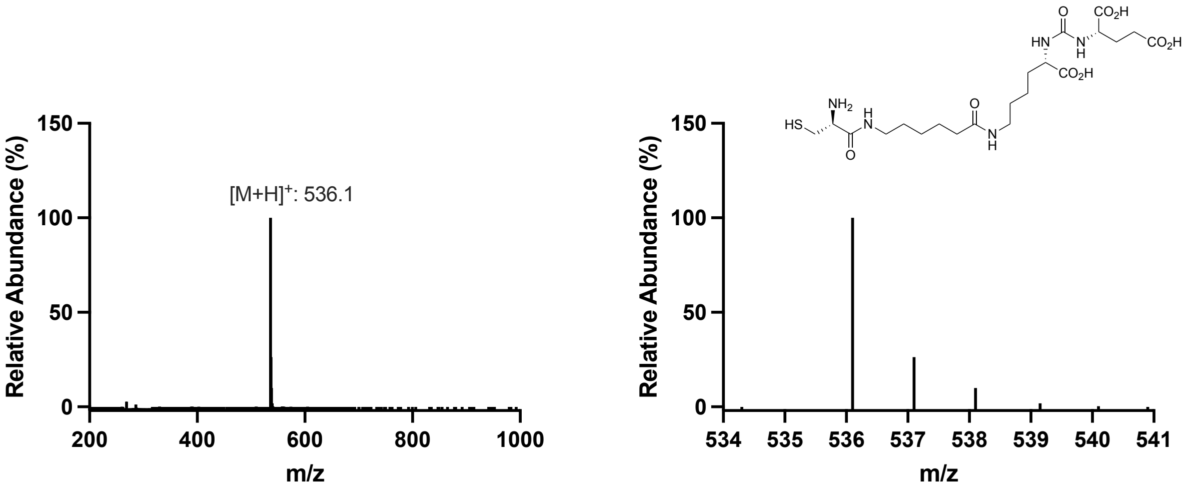

Figure S126. ESI-MS of **C-Hex-KuE**. Calc m/z for $C_{21}H_{38}N_5O_9S$ $[M+H]^+$: 536.2. Found: 536.1.

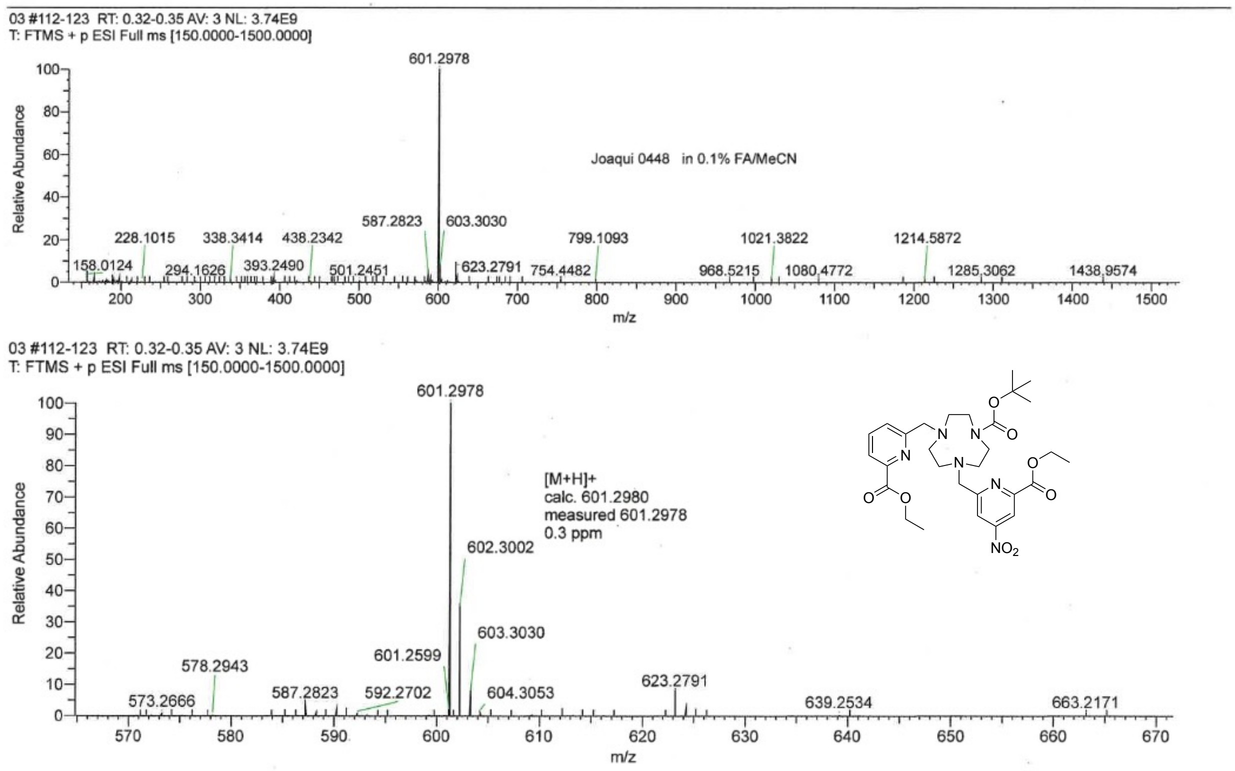

Figure S127. ESI-HRMS of **10**. Calc m/z for $C_{29}H_{41}N_6O_8$ $[M+H]^+$: 601.2980. Found: 601.2978.

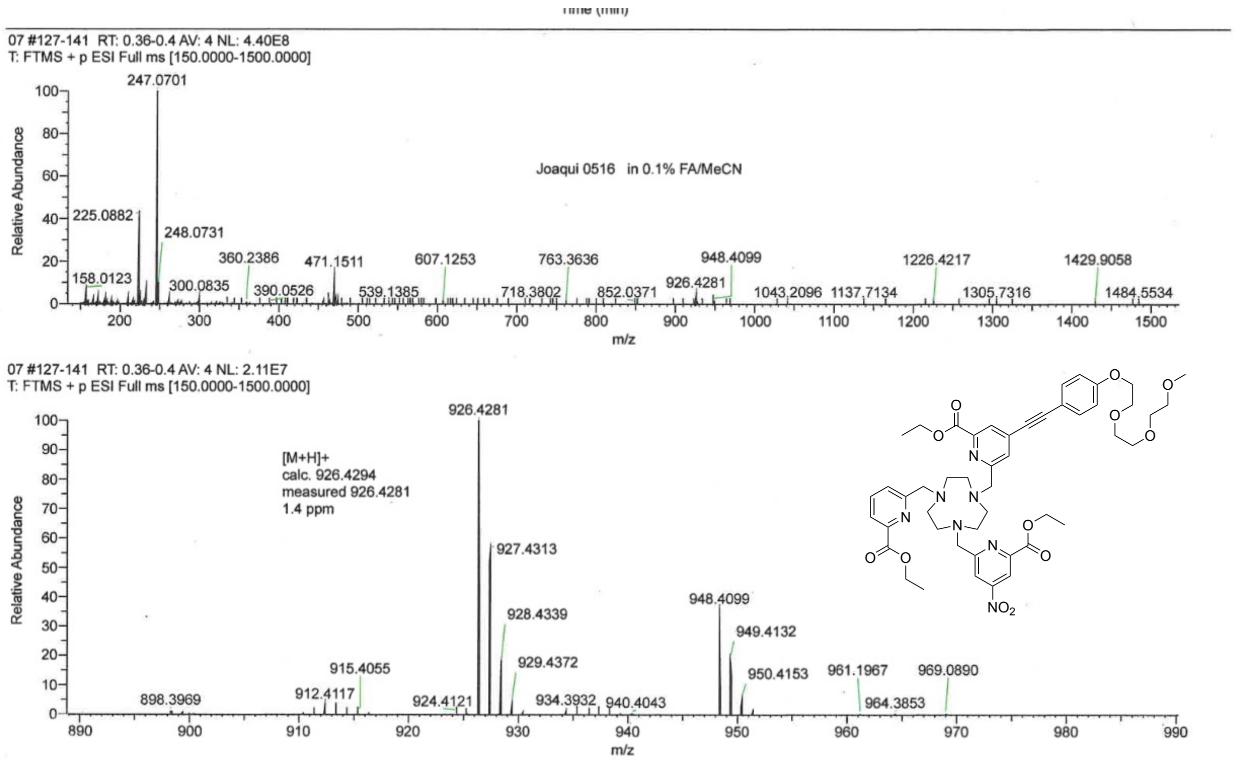

Figure S128. ESI-HRMS of **11**. Calc m/z for $C_{48}H_{60}N_7O_{12}$ $[M+H]^+$: 926.4294. Found: 926.4281. Calc m/z for $C_{48}H_{59}N_7NaO_{12}$ $[M+Na]^+$: 948.4119. Found: 948.4099.

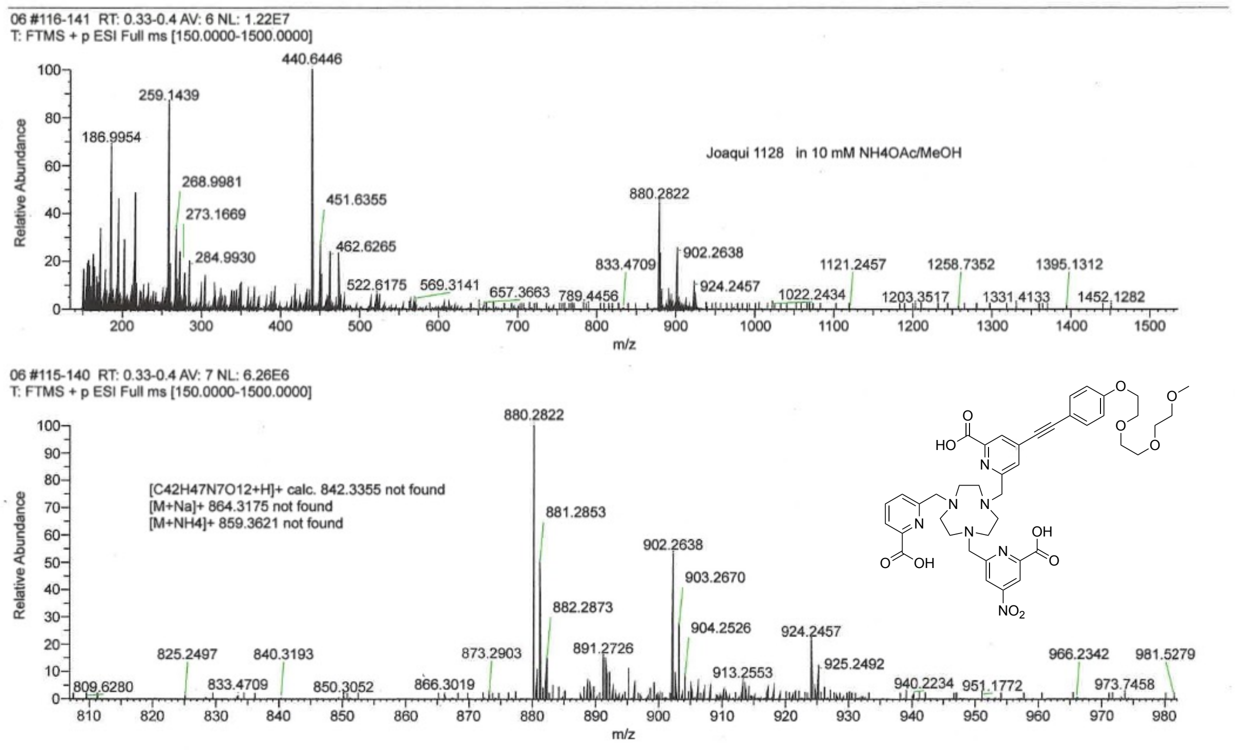

Figure S129. ESI-HRMS of **12**. Calc m/z for $C_{42}H_{47}KN_7O_{12}$ $[M+K]^+$: 880.2920. Found: 880.2822.

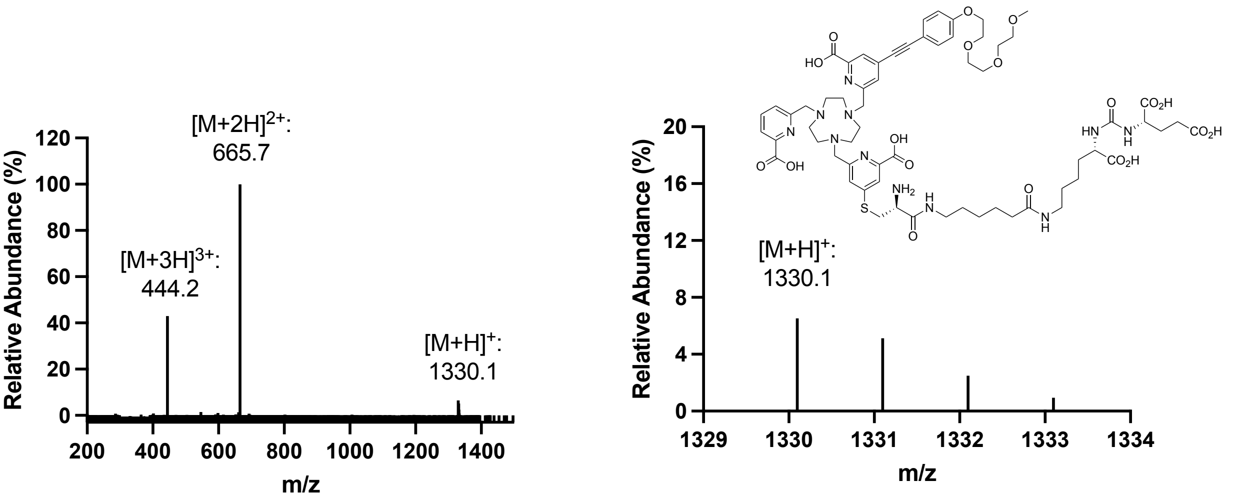

Figure S130. ESI-MS of **pepa-pic$_2$-C-Hex-KuE**. Calc m/z for $C_{63}H_{84}N_{11}O_{19}S$ $[M+H]^+$: 1330.6. Found 1330.1. Calc m/z for $C_{63}H_{85}N_{11}O_{19}S$ $[M+2H]^{2+}$: 665.8. Found 665.7. Calc m/z for $C_{63}H_{86}N_{11}O_{19}S$ $[M+3H]^{3+}$: 444.2. Found 444.2.

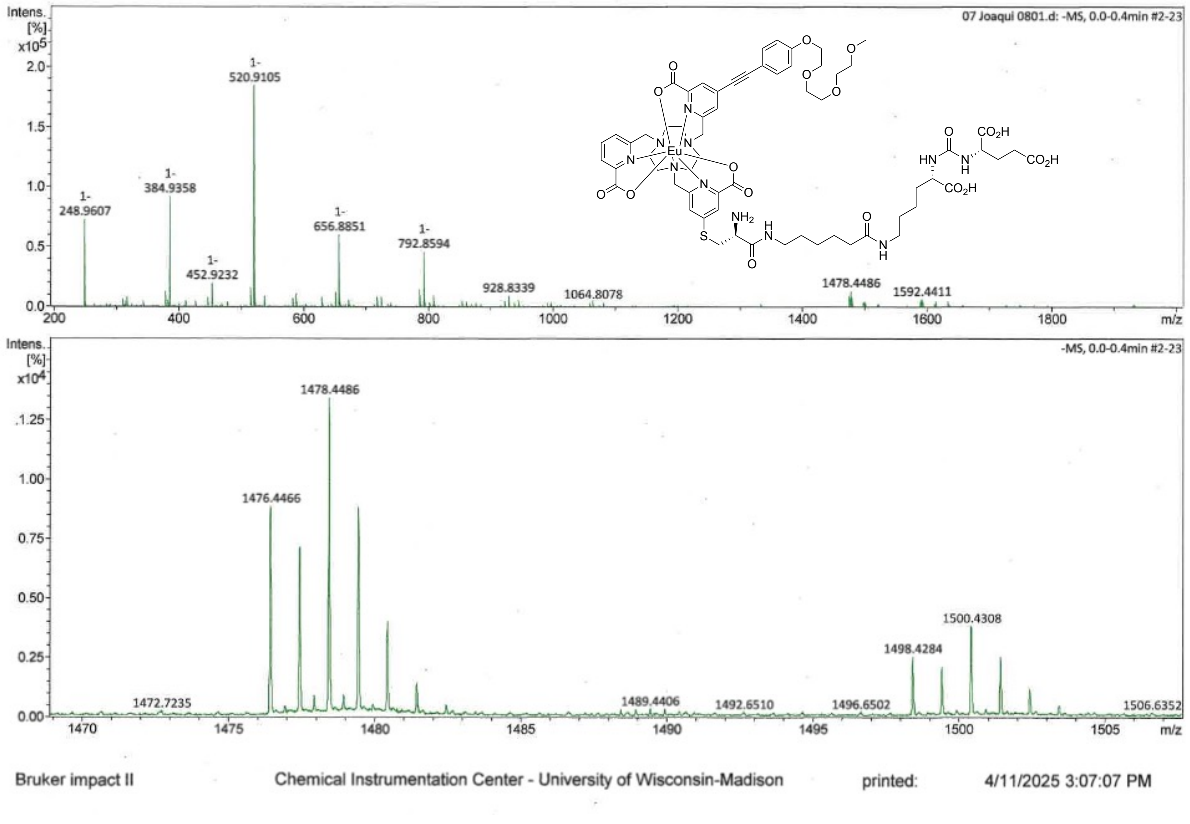

Figure S131. ESI-HRMS of ^{nat}**Eu-pepa-pic$_2$-C-Hex-KuE**. Calc m/z for $C_{63}H_{79}EuN_{11}O_{19}S$ $[M-H]^-$: 1478.4487. Found: 1478.4486.

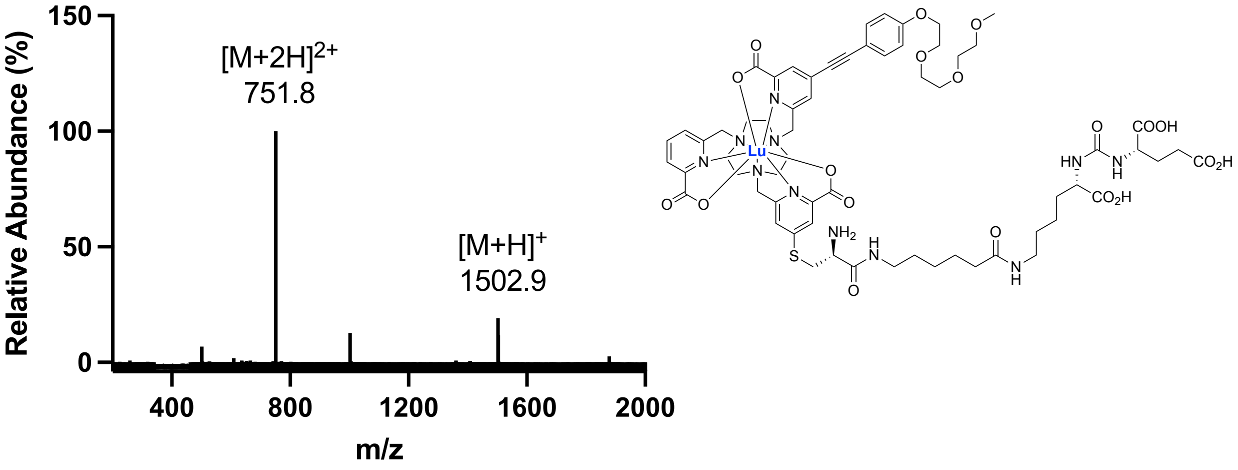

Figure S132. ESI-MS of **^{nat}Lu-pepa-pic$_2$-C-Hex-KuE**. ESI-MS: Calc m/z for $C_{63}H_{81}LuN_{11}O_{19}S$ $[M+H]^+$: 1502.5. Found: 1502.9. Calc m/z for $C_{63}H_{82}LuN_{11}O_{19}S$ $[M+2H]^{2+}$: 751.7. Found: 751.8.

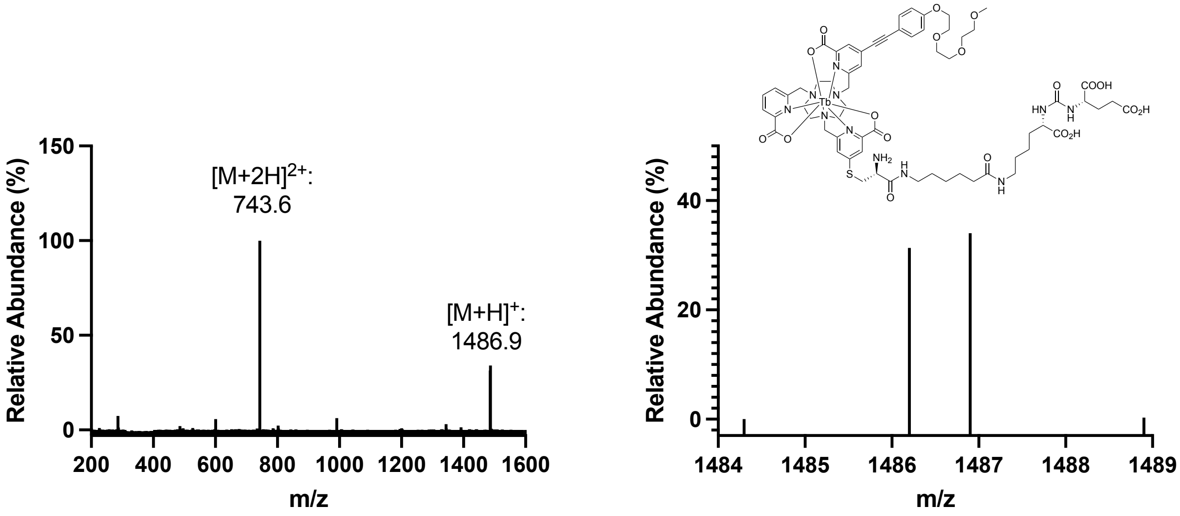

Figure S133. ESI-MS of **^{nat}Tb-pepa-pic$_2$-C-Hex-KuE**. Calc m/z for $C_{63}H_{81}N_{11}O_{19}STb$ $[M+H]^+$: 1486.5. Found: 1486.9. Calc m/z for $C_{63}H_{82}N_{11}O_{19}STb$ $[M+2H]^{2+}$: 743.7. Found: 743.6.

6 References

(1) Gai, Y.; Sun, L.; Lan, X.; Zeng, D.; Xiang, G.; Ma, X. Synthesis and Evaluation of New Bifunctional Chelators with Phosphonic Acid Arms for Gallium-68 Based PET Imaging in Melanoma. *Bioconjug Chem.* **2018**, *29* (10), 3483-3494.

(2) Śmiłowicz, D.; Schlyer, D.; Boros, E.; Meimetis, L. Evaluation of a Radio-IMmunoStimulant (RIMS) in a Syngeneic Model of Murine Prostate Cancer and ImmunoPET Analysis of T-cell Distribution. *Mol Pharm* **2022**, *19* (9), 3217-3227.

(3) Kelderman, C. A. A.; Glaser, O. M.; Whetter, J. N.; Aluicio-Sarduy, E.; Mixdorf, J. C.; Sanders, K. M.; Guzei, I. A.; Barnhart, T. E.; Engle, J. W.; Boros, E. Charting the coordinative landscape of the 18F–Sc/44Sc/177Lu triad with the tri-aza-cyclononane (tacn) scaffold. *Chem. Sci.* **2024**, *15* (43), 17927-17936.
(4) Lengacher, R.; Cosby, A. G.; Śmiłowicz, D.; Boros, E. Validation of a post-radiolabeling bioconjugation strategy for radioactive rare earth complexes with minimal structural footprint. *Chem. Commun.* **2022**, *58* (99), 13728-13730.
(5) Lengacher, R.; Martin, K. E.; Śmiłowicz, D.; Esseln, H.; Lotlikar, P.; Grichine, A.; Maury, O.; Boros, E. Targeted, Molecular Europium(III) Probes Enable Luminescence-Guided Surgery and 1 Photon Post-Surgical Luminescence Microscopy of Solid Tumors. *J. Am. Chem. Soc.* **2023**, *145* (44), 24358-24366.
(6) Supkowski, R. M.; Horrocks, W. D. On the determination of the number of water molecules, q, coordinated to europium(III) ions in solution from luminescence decay lifetimes. *Inorg. Chim. Acta* **2002**, *340*, 44-48.
(7) Beeby, A.; M. Clarkson, I.; S. Dickins, R.; Faulkner, S.; Parker, D.; Royle, L.; S. de Sousa, A.; A. Gareth Williams, J.; Woods, M. Non-radiative deactivation of the excited states of europium, terbium and ytterbium complexes by proximate energy-matched OH, NH and CH oscillators: an improved luminescence method for establishing solution hydration states. *J. Chem. Soc., Perkin Trans. 2* **1999**, (3), 493-504.
(8) Kovacs, D.; Kiraev, S. R.; Phipps, D.; Orthaber, A.; Borbas, K. E. Eu(III) and Tb(III) Complexes of Octa- and Nonadentate Macrocyclic Ligands Carrying Azide, Alkyne, and Ester Reactive Groups. *Inorg. Chem.* **2020**, *59* (1), 106-117.
(9) Antaris, A. L.; Chen, H.; Diao, S.; Ma, Z.; Zhang, Z.; Zhu, S.; Wang, J.; Lozano, A. X.; Fan, Q.; Chew, L.; Zhu, M.; Cheng, K.; Hong, X.; Dai, H.; Cheng, Z. A high quantum yield molecule-protein complex fluorophore for near-infrared II imaging. *Nat. Commun.* **2017**, *8* (1), 15269.
(10) Scott, T. A., Jr. Refractive Index of Ethanol–Water Mixtures and Density and Refractive Index of Ethanol–Water–Ethyl Ether Mixtures. *The Journal of Physical Chemistry* **1946**, *50* (5), 406-412.
(11) Vaughn, B. A.; Loveless, C. S.; Cingoranelli, S. J.; Schlyer, D.; Lapi, S. E.; Boros, E. Evaluation of 177Lu and 47Sc Picaga-Linked, Prostate-Specific Membrane Antigen-Targeting Constructs for Their Radiotherapeutic Efficacy and Dosimetry. *Mol Pharm* **2021**, *18* (12), 4511-4519.
